# Halide Abstraction Competes with Oxidative Addition in the Reactions of Aryl Halides with [Ni(PMe_n_Ph_(3−*n*)_)_4_]

**DOI:** 10.1002/chem.201702331

**Published:** 2017-11-20

**Authors:** Ignacio Funes‐Ardoiz, David J. Nelson, Feliu Maseras

**Affiliations:** ^1^ Institute of Chemical Research of Catalonia The Barcelona Institute of Science and Technology Avgda. Països Catalans 16 43007 Tarragona Spain; ^2^ WestCHEM Department of Pure and Applied Chemistry University of Strathclyde 295 Cathedral Street Glasgow G1 1XL UK; ^3^ Departament de Química Universitat Autònoma de Barcelona 08193 Bellaterra, Catalonia Spain).

**Keywords:** density functional calculations, electron transfer, homogeneous catalysis, ligand effects, nickel

## Abstract

Density functional theory (DFT) calculations have been used to study the oxidative addition of aryl halides to complexes of the type [Ni(PMe_n_Ph_(3−*n*)_)_4_], revealing the crucial role of an open‐shell singlet transition state for halide abstraction. The formation of Ni^I^ versus Ni^II^ has been rationalised through the study of three different pathways: (i) halide abstraction by [Ni(PMe_n_Ph_(3−*n*)_)_3_], via an open‐shell singlet transition state; (ii) S_N_2‐type oxidative addition to [Ni(PMe_n_Ph_(3−*n*)_)_3_], followed by phosphine dissociation; and (iii) oxidative addition to [Ni(PMe_n_Ph_(3−*n*)_)_2_]. For the overall reaction between [Ni(PMe_3_)_4_], PhCl, and PhI, a microkinetic model was used to show that our results are consistent with the experimentally observed ratios of Ni^I^ and Ni^II^ when the PEt_3_ complex is used. Importantly, [Ni(PMe_n_Ph_(3−*n*)_)_2_] complexes often have little, if any, role in oxidative addition reactions because they are relatively high in energy. The behaviour of [Ni(PR_3_)_4_] complexes in catalysis is therefore likely to differ considerably from those based on diphosphine ligands in which two coordinate Ni^0^ complexes are the key species undergoing oxidative addition.

## Introduction

The development of catalytic methods that use abundant, sustainable, and less expensive elements is an area of recent and intense focus. Nickel is one such element that has recently received increased attention.^1^ Nickel can catalyse a range of reactions, including: cross‐coupling reactions of halide and phenol‐derived substrates,^2^ rearrangement reactions of unsaturated aliphatic substrates,^3^ tandem photocatalysis/cross‐coupling reactions,^4^, ^5^ and reductive cross‐coupling reactions.^6^ To fully exploit the catalytic potential of nickel, it is essential to understand the mechanistic aspects of these reactions. The accessibility of oxidation states +I and +III complicates mechanistic understanding. A number of studies identify Ni^I^ intermediates or products from catalytic reactions,^7^, ^8^, ^9^, ^10^, ^11^ but others suggest that they are often not involved in catalysis.^12^, ^13^ It has been shown that ligand^14^ and substrate^15^ structure are both crucial in determining the role, if any, of Ni^I^ in catalysis. Further investigation is essential to fully understand when and why Ni^I^ arises, and what its role is in catalysis, because this will have an impact upon the development of nickel‐catalysed reactions.

**Figure 1:**
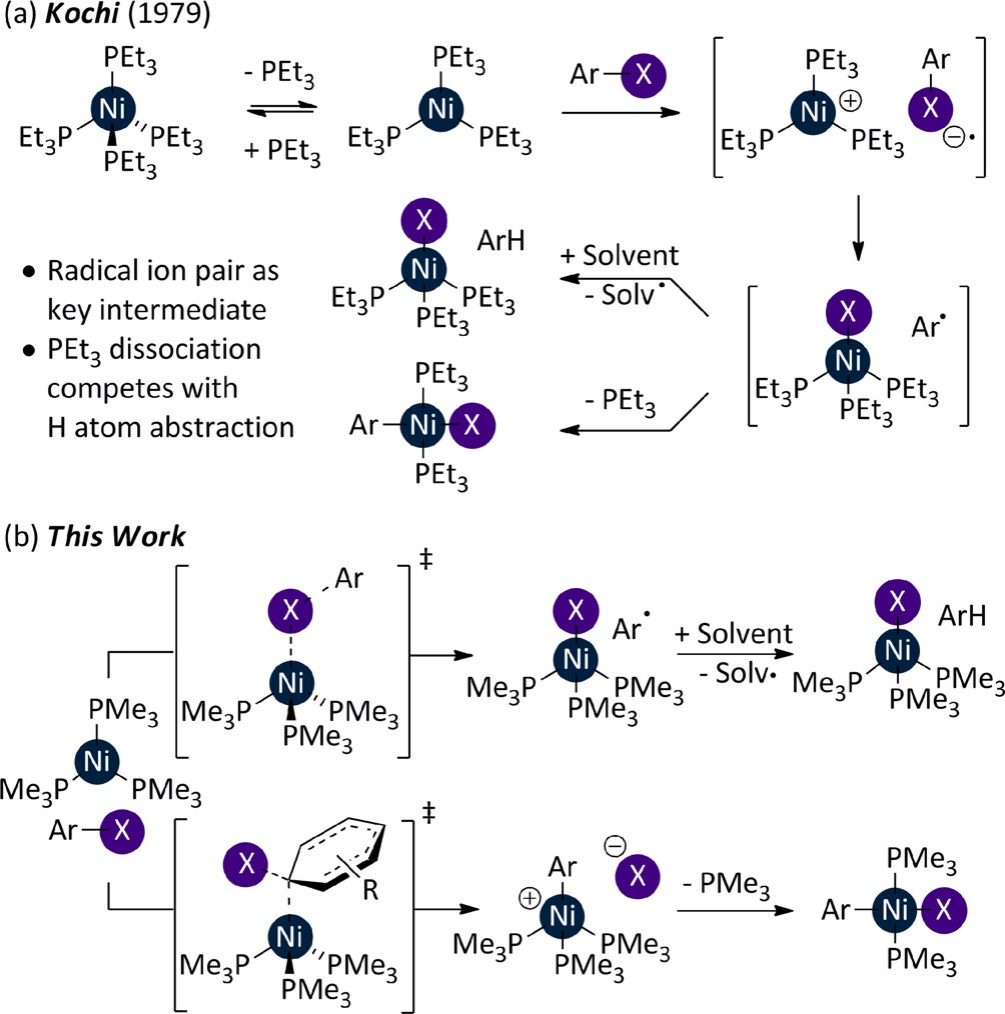
Oxidative addition of aryl halides to [Ni(PEt_3_)_4_]: (a) Kochi's mechanistic proposal and, (b) our mechanistic proposal.

In the seminal experimental study of oxidative addition to Ni^0^ using [Ni(PEt_3_)_4_], it was proposed that electron transfer from [Ni(PEt_3_)_3_] to the aryl halide forms a solvent‐caged radical ion pair;^16^ the nickel centre could then trap the aryl radical, or the radical could escape the cage and react with the solvent (Figure 1 (a)). Comproportionation was ruled out as a possible pathway to Ni^I^ from Ni^II^. This mechanism differs from that established for [Pd(PPh_3_)_4_], which proceeds via [Pd(PPh_3_)_2_] and a concerted three‐centre transition state.^17^ Concerted three‐centre transition states have been computationally characterised for oxidative addition to Ni^0^ complexes bearing bidentate phosphine ligands.^9^, ^12^, ^14^, ^18^, ^19^, ^20^ We present evidence from computational studies that Ni^I^ and Ni^II^ products both do indeed arise via [Ni(PEt_3_)_3_]; Ni^I^ is obtained via an open‐shell singlet transition state and Ni^II^ is formed from an S_N_2‐type oxidative addition event (Figure 1 (b)). Furthermore, we have investigated how this behaviour changes as the structure of the phosphine is varied, in order to lay the groundwork for future catalyst and reaction design.

## Results and Discussion

As a basis for developing a model for understanding structure/activity relationships in nickel catalysis, the oxidative addition of aryl halides to a model Ni^0^ complex was studied using density functional theory; free energies and enthalpies are reported using the B3LYP functional with Grimme's D3 corrections in THF solution (SMD) unless otherwise stated. Please see the Computational Methods for further details, and the Supporting Information for details of benchmarking. [Ni(PMe_3_)_4_] was used as a model for [Ni(PEt_3_)_4_] to reduce the computational cost and to prevent issues arising from the conformational complexity of PEt_3_; PMe_3_ has sufficiently similar properties to PMe_3_ to be an appropriate model.^21^, ^22^

In solution, [Ni(PEt_3_)_4_] spontaneously dissociates PEt_3_ to form [Ni(PEt_3_)_3_] (*K*_eq_≈10^−3^ mol L^−1^), with further PEt_3_ dissociation to form [Ni(PEt_3_)_2_] being possible (*K*_eq_<10^−6^ mol L^−1^).^16^, ^22^ DFT calculations yield free energy differences of 7.8 and 12.7 kcal mol^−1^ for the first and second phosphine dissociation events, respectively, which is consistent with experiment (Δ*H*=23.8 and 25.0 kcal mol^−1^, respectively). A relaxed scan of the potential energy as the Ni−P distance was increased to 4.0 Å identified no transition states for these dissociative reactions, but only a gradual increase in energy (see Figure S1 in the Supporting Information).

**Figure 2:**
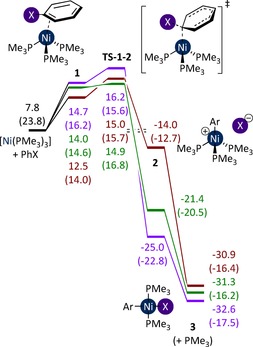
Oxidative addition of PhI (purple), PhBr (brown), and PhCl (green) to [Ni(PMe_3_)_3_]. Energies are free energies, in kcal mol^−1^ relative to [Ni(PMe_3_)_4_] plus aryl halide, in THF solvent. Enthalpies are provided in parentheses.

It is known from studies of oxidative addition to Pd^0^ that the number of phosphine ligands coordinated to the metal centre has a profound effect on reactivity and selectivity.^23^, ^24^, ^25^, ^26^, ^27^ [Ni(PMe_3_)_2_] and [Ni(PMe_3_)_3_] can each undergo oxidative addition to PhX (X=Cl, Br, I). Barriers for the oxidative addition to [Ni(PMe_3_)_2_] are lower in all three cases (Figure S2), but the dissociation of PMe_3_ from [Ni(PMe_3_)_3_] is sufficiently endergonic to place these transition states higher on the free energy surface than those proceeding via [Ni(PMe_3_)_3_]. The most energetically favourable oxidative addition pathway is therefore the addition of PhX to [Ni(PMe_3_)_3_] (Figure 2). This reaction proceeds via the endergonic coordination of PhX to [Ni(PMe_3_)_3_] to form η^2^‐complexes **1**. These readily undergo S_N_2‐type oxidative addition with very similar barriers (1.4, 2.4, 0.9 kcal mol^−1^ for I, Br, Cl, respectively). The barrier is not so dependent on halide identity, but the overall barrier for the process results from the decrease in entropy due to coordination of the aryl halide to the Ni^0^ centre.

**Figure 3:**
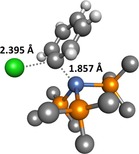
Transition state **TS‐1**–**2‐Cl** for the oxidative addition of chlorobenzene to [Ni(PMe_3_)_3_]. Some hydrogen atoms have been omitted for clarity.

Transition state **TS‐1**–**2‐Cl** is depicted in Figure 3. The Ni^0^ centre acts as the nucleophile, attacking the *ipso*‐carbon in an S_N_2‐like transition state, rather than a concerted three‐centre transition state. The overall barriers for this process, with respect to [Ni(PMe_3_)_4_] and aryl halide, are 14.9, 15.0, and 16.2 kcal mol^−1^ for PhCl, PhBr, and PhI, respectively. While this is not the order of reactivity that might be expected, the energy differences are very small. Finally, **2** evolves to *trans* square planar complex **3** by dissociating a phosphine ligand and capturing the halide.

[NiBr(PEt_3_)_3_] and [NiI(PEt_3_)_3_] were both isolated by Kochi,^16^ therefore a pathway was sought that connects [Ni(PMe_3_)_3_] with the Ni^I^ product. The reaction proceeds via [Ni(PMe_3_)_3_(XPh)] (**4**). For PhI and PhBr, the aryl halide ligates the nickel via an electron pair on the halide (*G*_rel_=8.5, 12.6 kcal mol^−1^, respectively) while for PhCl an η^2^‐complex is formed in which the *ortho*/*meta* edge of the arene coordinates the nickel (*G*_rel_=10.8 kcal mol^−1^).

An electron transfer mechanism was proposed by Kochi, so this was investigated first. One possibility is that there is a spin change within complex **4** from singlet to triplet, leading to the cleavage of the C−X bond and formation of a Ni^I^ complex and an aryl radical. The minimum energy crossing point (MECP), the geometry at which the singlet and triplet states of **4** have the same energy, was located using Harvey's software.^28^, ^29^ However, the barrier to this spin change is too high to be a plausible route to the Ni^I^ product (*G*_rel_=ca. 15 and 22 kcal mol^−1^ for PhI and PhBr, respectively; see the Supporting Information). In particular, the barrier for PhBr is 7 kcal mol^−1^ higher than the highest point on the oxidative addition pathway in Figure 2, and therefore no Ni^I^ would be expected. However, experimentally, [NiBr(PEt)_3_] is obtained from the oxidative addition of PhBr to [Ni(PEt)_4_]. Similarly, an outer‐sphere electron transfer process was investigated using a Marcus theory treatment, but the barriers involved are far too high to be a plausible explanation for the observed reactivity (details can be found in the Supporting Information).

**Figure 4:**
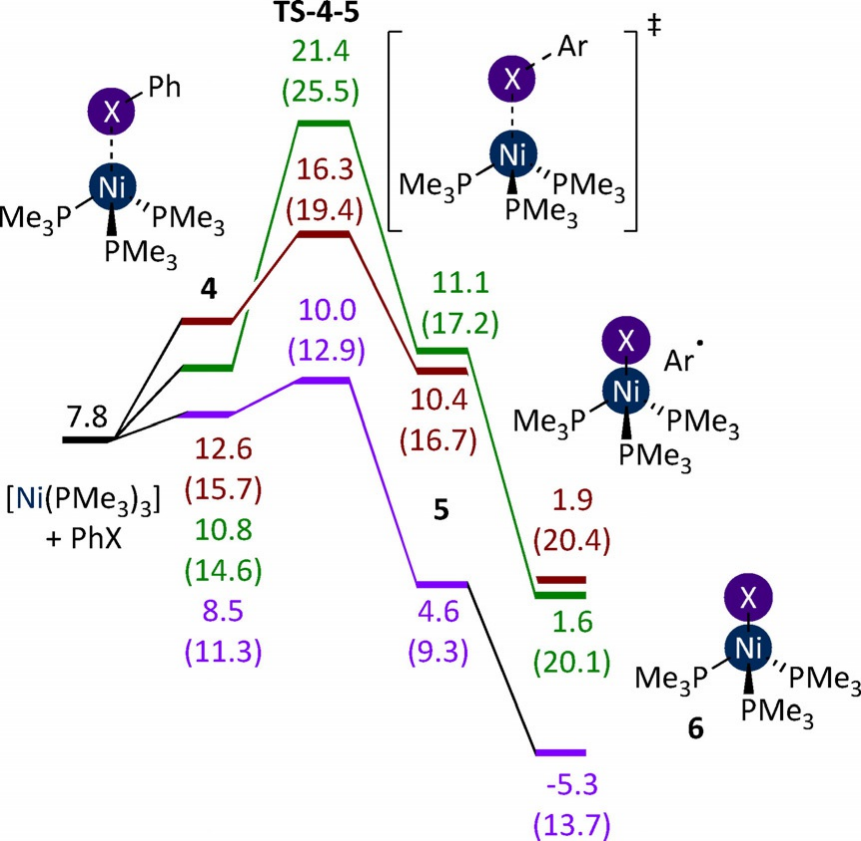
Halogen abstraction from PhI (purple), PhBr (orange), and PhCl (green) by [Ni(PMe_3_)_3_]. Energies are free energies, in kcal mol^−1^ relative to [Ni(PMe_3_)_4_] plus PhX, in THF solvent. Enthalpies are provided in parentheses.

**Figure 5:**
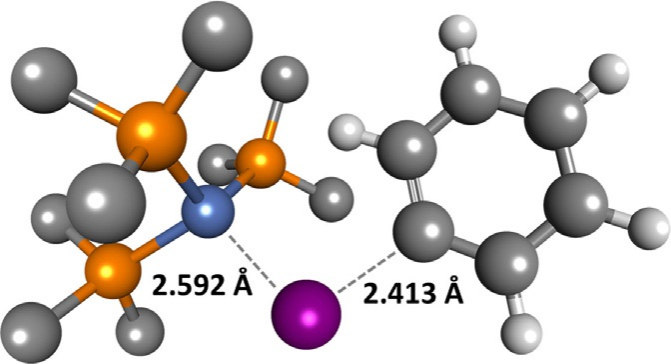
Transition state **TS‐4**–**5‐I** for the abstraction of iodine from PhI by [Ni(PMe_3_)_3_]. Some hydrogen atoms have been omitted for clarity.

The alternative is an open‐shell singlet transition state for halogen abstraction, with concomitant oxidation of Ni^0^ to Ni^I^ (Figure 4). Transition states were located for PhI, PhBr, and PhCl (**TS‐4**–**5**). The nickel has a partially open‐shell, allowing the transfer of an electron from the (full) *d*‐orbitals to the interacting σ^*^_CX_ orbital, simultaneously breaking the C−X bond. Finally, **5** dissociates phenyl radical to yield the experimentally observed [NiX(PMe_3_)_3_] complex (**6**). **TS‐4**–**5‐I** is shown in Figure 5.

For PhI, the barrier is 10.0 kcal mol^−1^ and so this pathway will be preferred over the oxidative addition mechanism described above. For PhBr, the barrier is 16.3 kcal mol^−1^, which is close to the energy of **TS‐1**–**2‐Br** (15.0 kcal mol^−1^). For PhCl, the barrier is much higher than the alternative oxidative addition transition state (21.4 versus 16.2 kcal mol^−1^). These results are consistent with experimental observations; PhI leads primarily to Ni^I^ products, PhBr yields mostly Ni^II^ but some Ni^I^, and PhCl yields exclusively Ni^II^ products.^16^ The free energy change between the coordinated aryl halide (Δ*G*=1.5, 3.7, 10.6 kcal mol^−1^ for PhI, PhBr, PhCl, respectively) is almost entirely due to a corresponding enthalpy change as the C‐X bond is broken (Δ*H*=1.6, 3.6, 10.9 kcal mol^−1^ for PhI, PhBr, PhCl, respectively).

**Figure 6:**
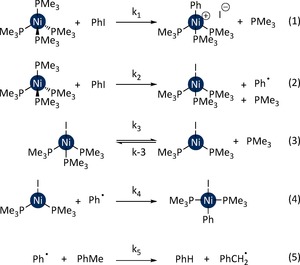
Microkinetic model for the competition between Ni^I^ and Ni^II^ formation.

Halogen abstraction is very sensitive to the aryl halide, with a difference of 11.4 kcal mol‐1 between **TS‐4**–**5‐I** and **TS‐4**–**5‐Cl**. This can be explained by the significant differences in the C−X bond dissociation energies (BDE), which are 66.9, 82.7, and 97.3 kcal mol^−1^ for PhI, PhBr, and PhCl, respectively.^30^ These BDE are proportional to the halogen abstraction barrier and this can be associated with the energy of the σ* orbital which receives an electron during the reaction. To enable a good interaction, this orbital should be low in energy, and therefore the reaction barrier decreases in the order PhCl>PhBr>PhI. The relative barriers for oxidative addition and halogen abstraction (**TS‐1**–**2** versus **TS‐4**–**5**) provide a good qualitative explanation for the behaviour of the three halobenzenes, but the quantitative agreement is limited. The application of a Boltzmann distribution to the relative barriers in Figures 2 and 4 leads to a predicted Ni^I^ : Ni^II^ selectivity of 0:100, 10:90, and 100:0 for PhCl, PhBr, and PhI, respectively. The available experimental values are, respectively, 0:100, 19:81, and 80:20. For the particular case of PhI, the experimental value is reported in toluene solution, but repeating the calculations in toluene did not yield a significant difference (see the Supporting Information). Better quantitative agreement between calculation and experiment can be obtained by taking into account the fate of the phenyl radical that is generated as a by‐product of Ni^I^ formation (Figure 4). This radical may react with the Ni^I^ complex to form the Ni^II^ species, or be lost by reaction with the solvent molecules. As the solvent and radical are present in very different concentrations, a microkinetic model was constructed. This type of approach has been used previously to understand competing processes that have been modelled using DFT calculations.^31^, ^32^, ^33^, ^34^, ^35^ The reaction between [Ni(PMe_3_)_4_] and PhI in toluene was simulated initially (Figure 6); the chemical reactions included in the kinetic model are presented below: oxidative addition (Eq. (1)), halogen abstraction (Eq. (2)), phosphine dissociation from **6** (Eq. (3)), phenyl radical trapping by the Ni^I^ complex (Eq. (4)), and the reaction of phenyl radical with the solvent (Eq. (5)). The barriers for Eq. (1) and Eq. (2) are taken from the calculations using toluene as the solvent, those for Eq. (3) and Eq. (4) are taken from calculated diffusion barriers, and the barrier for Eq. (5) was computed to be 10.6 kcal mol^−1^. The application of this microkinetic model yields a predicted Ni^I^:Ni^II^ ratio of 83:17, which is in significantly better agreement with the value of 80:20 determined experimentally. Applying the same methodology to the reaction of PhBr gave a ratio of 8:92 (versus 19:81 experimentally).

The explicit introduction of a solvent molecule in the microkinetic model has the additional advantage of rationalising selectivity. The barrier for hydrogen abstraction is a function of solvent, with values that can be determined computationally: 9.5 kcal mol^−1^ for THF, 10.6 kcal mol^−1^ for toluene, and 10.8 kcal mol^−1^ for *n*‐hexane. This correlates well with the observation that the Ni^I^:Ni^II^ ratio increases in the order *n*‐hexane< toluene<THF.

While this study reveals the mechanistic details underpinning the observed halogen and solvent effects disclosed in Kochi's seminal study of oxidative addition to [Ni(PEt_3_)_4_], it should be noted that this type of complex is rarely applied in modern catalysis. The reactivity with PhI of a series of analogous complexes, bearing PMe_2_Ph, PMePh_2_, and PPh_3_ ligands was therefore evaluated using the same methods.

The reactions of [Ni(PPh_3_)_4_] with aryl halides have been investigated previously by Cassar, who reported the rates of the reactions and their selectivities, but little analysis of the reaction products.^36^ Recently, Baird and Budzelaar have shown that [Ni(PPh_3_)_4_] reacts with PhCl, PhBr, and PhI to form [Ni(Ph)X(PPh_3_)_2_] and [NiX(PPh_3_)_3_] in varying proportions.^11^ Analogously to Kochi's study, the propensity to form Ni^I^ decreases in the order PhI>PhBr>PhCl. Interestingly, experimental evidence and computational studies showed that *trans*‐[NiX(Ph)(PPh_3_)_2_] decomposes rapidly to [NiX(PPh_3_)_3_] in the absence of excess PPh_3_.

**Figure 7:**
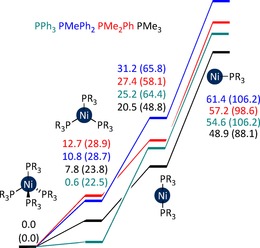
Free energies (kcal mol^−1^ versus [Ni(PR_3_)_4_]) for [Ni(PR_3_)_*n*_] complexes (PR_3_=PMe_3_, black; PMe_2_Ph, red; PMePh_2_, blue; or PPh_3_, teal) in THF solvent. Enthalpies are provided in parentheses.

The equilibria for phosphine dissociation differ somewhat across the four ligand systems, but it is clear that in all cases [Ni(PR_3_)] is far too high in energy to play a role in the reaction (Figure 7; PMe_3_ is included for comparison). However, [Ni(PR_3_)_3_] is readily formed in each case, particularly for PPh_3_ (*G*_rel_=0.6 kcal mol^−1^; *H*_rel_=22.5 kcal mol^−1^). Three possible pathways were then considered for the reaction of these complexes with PhI: (i) halogen abstraction by [Ni(PR_3_)_3_], (ii) oxidative addition to [Ni(PR_3_)_3_], and (iii) oxidative addition to [Ni(PR_3_)_2_].

**Figure 8:**
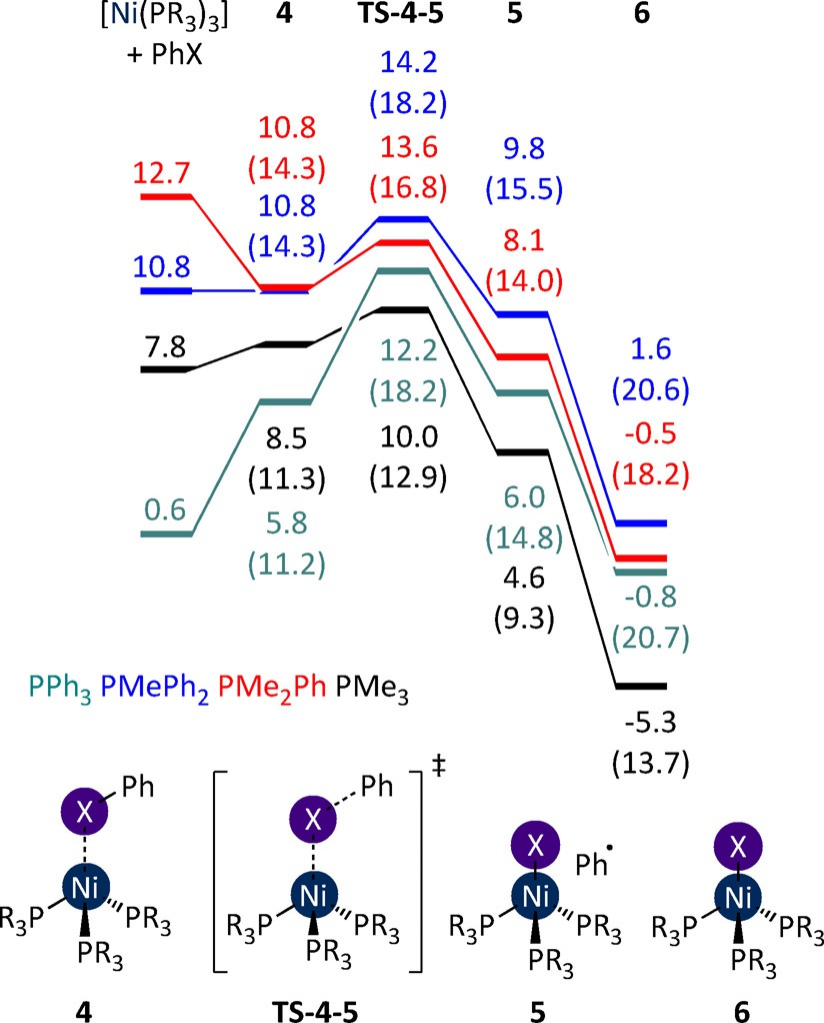
Halogen abstraction from PhI by [Ni(PR_3_)_3_]. Energies are free energies, in kcal mol^−1^ relative to [Ni(PMe_3_)_4_] plus PhX, in THF solvent (PR_3_=PMe_3_, black; PMe_2_Ph, red; PMePh_2_, blue; or PPh_3_, teal). Enthalpies are in parentheses.

The formation of **4** bears no significant energetic penalty, and is exergonic for PMe_2_Ph. Open‐shell singlet transition states link **4** to [NiI(PR_3_)_3_] and phenyl radical (Figure 8). The energies of **TS‐4**–**5** suggest that the reaction is facile in each case, even for less electron‐rich ligands.

**Figure 9:**
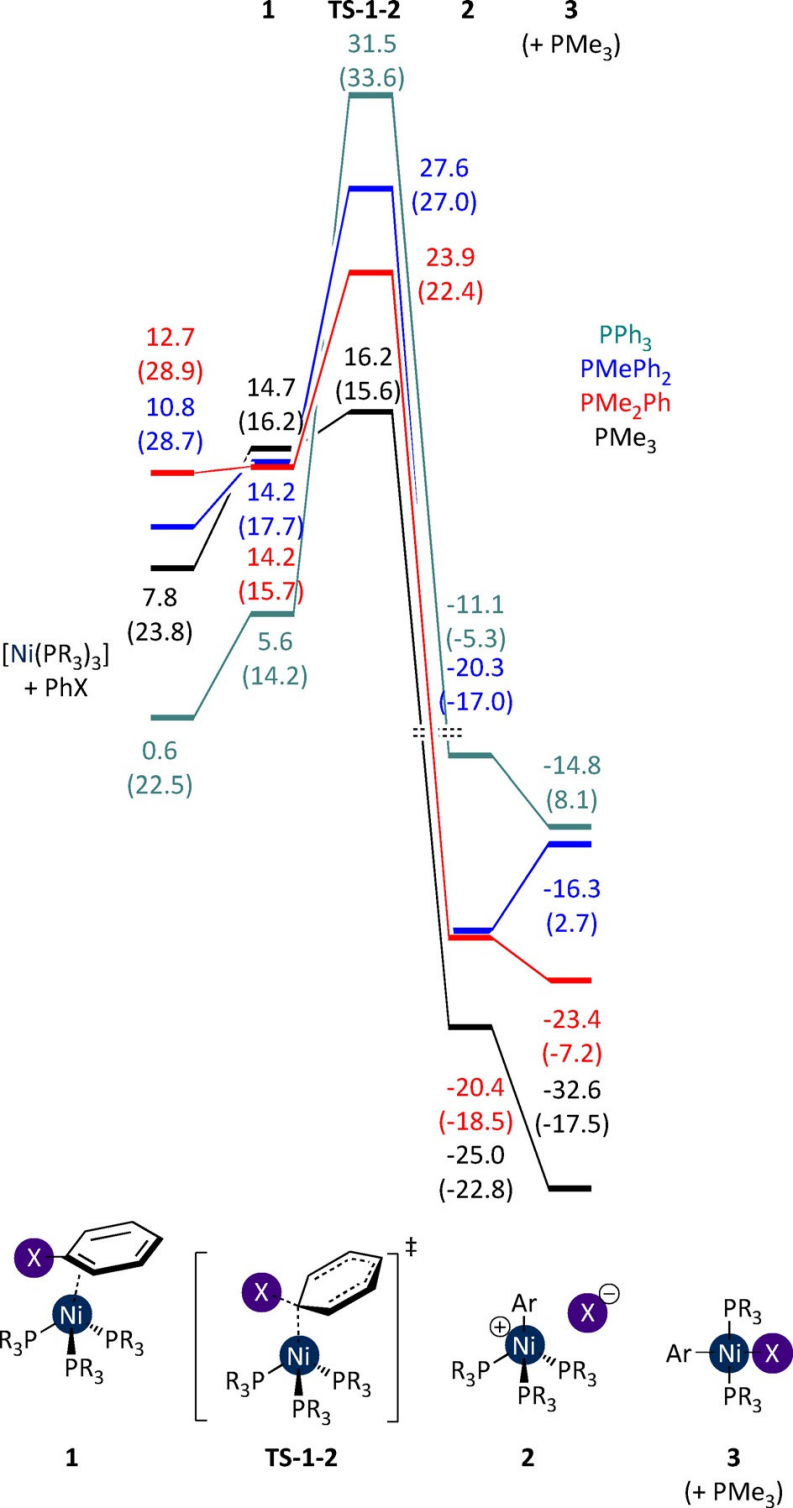
Oxidative addition of PhI to [Ni(PR_3_)_3_]. Energies are free energies, in kcal mol^−1^ relative to [Ni(PMe_3_)_4_] plus aryl halide, in THF solvent (PR_3_=PMe_3_, black; PMe_2_Ph, red; PMePh_2_, blue; or PPh_3_, teal). Enthalpies are provided in parentheses.

In each case the energy of this transition state (*G*_rel_=10.0–14.2 kcal mol^−1^) is considerably lower than that of the corresponding [Ni(PR_3_)_2_] complex (*G*_rel_=20.5–31.2 kcal mol^−1^). Oxidative addition via [Ni(PR_3_)_2_] cannot therefore be completive with this pathway. Instead, the free energy profiles for halide abstraction must be compared with those for oxidative addition via the [Ni(PR_3_)_3_] complex (Figure 9). However, the oxidative addition transition states involving [Ni(PR_3_)_3_] are high in energy (*G*_rel_=ca. 24–28 kcal mol^−1^), and much higher in energy than the halogen abstraction transition state. With the exception of the PMe_3_ system, the oxidative addition of PhI to [Ni(PR_3_)_3_] cannot compete with the halogen abstraction pathway, and therefore this reaction will occur exclusively via the latter pathway for PMe_2_Ph, PMePh_2_, and PPh_3_ complexes.

## Conclusion

This study provides new insight into the mechanisms of oxidative addition of aryl halides to nickel complexes bearing monodentate phosphine ligands. Importantly, the key pathways—S_N_2‐type oxidative addition and halide abstraction—both proceed via the [Ni(PR_3_)_3_] complex. The [Ni(PR_3_)_2_] complex plays little, if any, role in the oxidative addition reaction when PMe_3_ is involved. In the specific case of iodobenzene, this holds true not only for PMe_3_, but for more commonly employed ligands such as PPh_3_.

The open‐shell singlet transition state for the direct formation of Ni^I^ from Ni^0^ had not previously been reported, yet it clearly accounts for the major product in the reactions of aryl iodides with [Ni(PR_3_)_4_] complexes. Similar transition states have been shown to play a role in nickel‐mediated C−H functionalisation reactions where substrates have relatively low bond dissociation energies.^15^

The pathway proceeding via S_N_2‐type oxidative addition of the aryl halide (via **TS‐1**–**2**) is influenced only slightly by the identity of the halide, while the open‐shell singlet transition state for halogen abstraction presents a more variable barrier that depends on the energy of the σ^*^_CX_ orbital. The selectivity for Ni^I^ versus Ni^II^ is determined by the competition between both transition states and also by the reactions of the aryl radical with Ni^I^ or the solvent. From a computational perspective, the importance of considering open‐shell broken‐symmetry transition states and microkinetic models is demonstrated. The coordination number of the Ni^0^ intermediate is crucial; in contrast, most reactions of bisphosphine‐Ni^0^ complexes will proceed via complexes with only two phosphorus moieties attached, and will show rather different reactivity. Recent studies have shown that Ni^I^ complexes with bidentate phosphine ligands most likely arise as a result of comproportionation, rather than via electron transfer or halide abstraction.^7^, ^9^, ^10^, ^12^, ^14^ Here, the special role of the tris(phosphine) complex is another example of the delicate interplay between ligand choice and reactivity. This balance must be considered carefully in the design and study of nickel‐catalysed cross‐coupling method.

## Computational Methods

All DFT calculations were carried out using Gaussian 09 (Rev D.01).^37^ The B3LYP functional,^38^, ^39^, ^40^ was employed using Grimme's D3 corrections to account for dispersion interactions.^41^ Solvation (THF, toluene, *n*‐hexane) was accounted for using the SMD implicit solvation model.^42^ For open‐shell singlet structures, the potential energy was corrected using Yamaguchi's equation to account for spin contamination.^43^, ^44^, ^45^, ^46^, ^47^ Optimization and frequency calculations were carried out with the following basis set: LANL2TZ(f) on Ni; LANL2DZ(dp) on Br, I; 6‐31G(d) on all other atoms. Potential energies were then refined using the larger basis set: LANL2TZ(f) on Ni; LANL2DZ(dp) on Br, I; 6–311+G(d,p) on all other atoms. All calculations were carried out in THF solution unless stated otherwise. A data set collection of computational results is available in the ioChem‐BD repository^48^ (these can be accessed via https://doi.org/10.19061/iochem‐bd‐1‐55). Kinetic simulations were carried out using the COPASI software package.^49^ Full details of the computational methodology, benchmarking studies, and Cartesian coordinates for each structure, can be found in the Supporting Information.

## Conflict of interest

The authors declare no conflict of interest.

## Supporting information

As a service to our authors and readers, this journal provides supporting information supplied by the authors. Such materials are peer reviewed and may be re‐organized for online delivery, but are not copy‐edited or typeset. Technical support issues arising from supporting information (other than missing files) should be addressed to the authors.

Supplementary
