# Supplementary material for: Halide Abstraction Competes with Oxidative Addition in the Reactions of Aryl Halides with [Ni(PMenPh(3−n))4]
Source: Chemistry. 2017 Nov 20;23(66):16728–33. doi: 10.1002/chem.201702331 (PMC5725734; doi:10.1002/chem.201702331)
Supplement: Supplementary file 1 — Supplementary [file CHEM-23-16728-s001.pdf]

CHEMISTRY
A European Journal

Supporting Information

# Halide Abstraction Competes with Oxidative Addition in the Reactions of Aryl Halides with $[Ni(PMe_nPh_{(3-n)})_4]$

Ignacio Funes-Ardoiz,[a] David J. Nelson,*[b] and Feliu Maseras*[a, c]

chem_201702331_sm_miscellaneous_information.pdf

# Contents


COMPUTATIONAL DETAILS ........ S2

BENCHMARK OF FUNCTIONALS ........ S3

PHOSPHINE DISSOCIATION POTENTIAL ENERGY RELAXED SCAN FROM Ni($PMe_3$)$_4$ ........ S4

ALTERNATIVE MECHANISMS........ S5

(A) Outer-sphere electron transfer from [Ni($PMe_3$)$_4$] to ArX ........ S5

(B) Inner-sphere electron transfer from [Ni($PMe_3$)$_3$] to ArX ........ S6

(C) Oxidative Addition Pathway from [Ni($PMe_3$)$_2$] ........ S7

MICROKINETIC MODEL ........ S8

(A) Reaction between [Ni($PMe_3$)$_4$] to PhI in toluene ........ S8

(B) Reaction between [Ni($PMe_3$)$_4$] to PhBr in THF ........ S10

COORDINATES AND ENERGIES FOR MINIMA AND TRANSITION STATES ........ S12

REFERENCES ........ S74

# COMPUTATIONAL DETAILS

Calculations were performed using Gaussian09 (Rev. D01)[1] at the B3LYP level of theory[2,3,4] with Grimme's D3 dispersion correction.[5] This correction renders B3LYP appropriate for studying organometallic chemistry.[6] For optimizations and frequency calculations, the 6-31G(d) basis set[7,8,9] was used for H, C, N, O, P, and Cl atoms, and the LANL2DZdp basis set/ECP[10] used for Br and I, and LANL2LTZ(f) [11,12,13] for Ni. The potential energies were refined using the same basis set for Br, I and Ni, and increasing the basis set for H, C, N, O, P and Cl, using 6-311+G(d,p).[14] The solvent is experimentally significant, so all calculations were carried out in solvent (using the SMD method for implicit solvation), unless otherwise stated. Unless otherwise stated all calculations were carried out on THF. Toluene and *n*-hexane were used in specific cases (see the manuscript text for discussion of solvent effects).

The nature of each stationary point was verified using frequency analyses, and IRC calculations were used to verify that transition states linked the relevant minima. Free energy corrections were computed at 298 K and 1 atm. All energies quoted are Gibbs free energies in solution, in kcal mol$^{-1}$. Enthalpies are given in the schemes, in brackets, in kcal mol$^{-1}$.

In the case of open-shell singlet structures, where $S^2$ is not zero, we corrected the energy by applying the Yamaguchi's equation,[15,16,17,18] in the form:[19]

$$E_{singlet} = \frac{2E_{OSS} - E_{triplet}\langle S^2\rangle_{OSS}}{2 - \langle S^2\rangle_{OSS}}$$

where $E_{OSS}$ is the energy of open-shell singlet calculation, $E_{triplet}$ is the energy of the triplet state at the open-shell singlet geometry and $\langle S^2\rangle_{OSS}$ is the $S^2$ value of the open-shell singlet calculation.

$PEt_3$ has a number of possible conformations, so the ligands were truncated to $PMe_3$, which has similar properties (Tolman electronic parameter = 2064.1 cm$^{-1}$ for $PMe_3$, 2061.7 cm$^{-1}$ for $PEt_3$; cone angle = 118° for $PMe_3$, 132° for $PEt_3$).[20] Tolman determined that both ligands undergo fast ligand exchange at a $Ni^0$ centre in benzene or toluene solution, although $PEt_3$ has a slightly greater propensity for dissociation.[21]

## BENCHMARK OF FUNCTIONALS

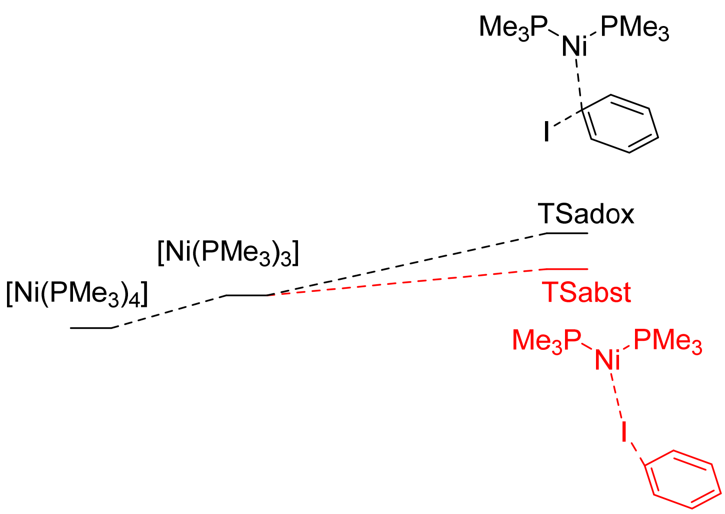


**Table S1.** Comparison of four different functionals on the key step of Ni(0) oxidation with PhI. Free energies in kcal/mol.

| Method* | $Ni(PMe_3)_4$ | $Ni(PMe_3)_3$ | TSadox | TSabst |
|---|---|---|---|---|
| B3LYP-D3 | 0.0 | 7.8 | 16.2 | 10.0 |
| TPSSh-D3 | 0.0 | 13.4 | 16.2 | 14.8 |
| ωB97X-D | 0.0 | 10.1 | 21.5 | 11.4 |
| PBE1PBE-D3 | 0.0 | 12.2 | 19.4 | 11.4 |

* Single point energies on B3LYP-D3 geometries using the LANL2DZdp basis set/ECP for I, LANL2LTZ(f) for Ni and 6-311+G(d,p) for the rest of atoms, with free energy corrections from B3LYP-D3 calculations.

The evaluation of different functionals in the key step with PhI and $PMe_3$ as ligand shows minor differences among all of them. The open-shell single electronic structure for the halogen abstraction transition state was found to be the most stable electronic structure in all the cases (using the wavefunction stability evaluation of g09). We selected B3LYP-D3 as the functional because the dissociation energy of the phosphine was the most consistent with experimental data and the evaluation of the product ratio provides a full agreement with the experimental observations.

## PHOSPHINE DISSOCIATION POTENTIAL ENERGY RELAXED SCAN FROM $Ni(PMe_3)_4$

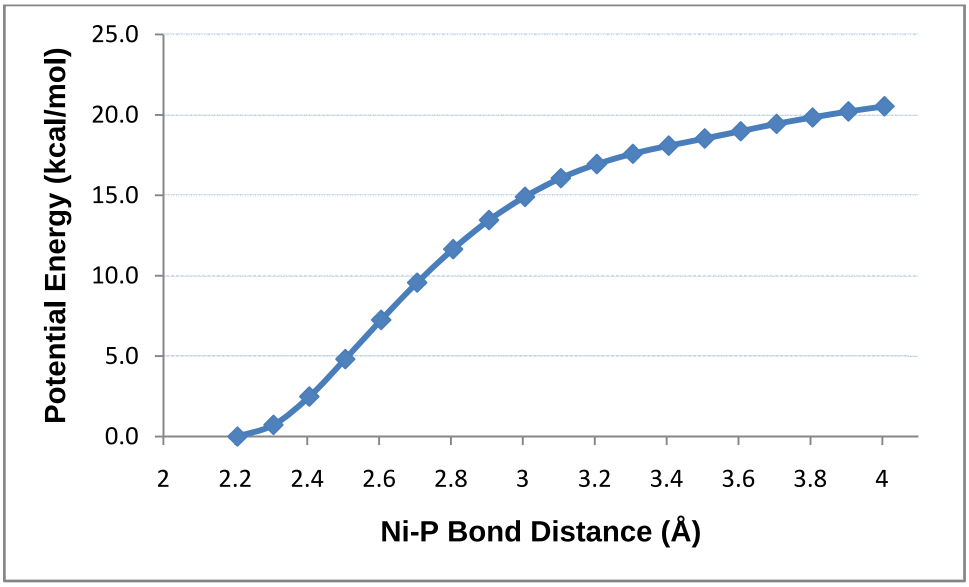


**Figure S1.** Potential energy relaxed scan for the phosphine dissociation in $Ni(PMe_3)_4$ complex.

According to the scan, there is no activation barrier for the phosphine dissociation in $Ni(PMe_3)_4$ complex.

# ALTERNATIVE MECHANISMS

## *(A) Outer-sphere electron transfer from [Ni($PMe_3$)$_4$] to ArX*

Electron transfer from [Ni($PMe_3$)$_4$] to ArX could in theory occur *via* an outer sphere mechanism, to form [Ni($PMe_3$)$_4$]$^+$ and the radical anion of ArX; the latter would then spontaneously dissociate $X^-$ to form an aryl radical. This was explored using Marcus-Hush theory, where the barrier to electron transfer can be estimated using equations S1-S3. This assumes that the major energetic barrier is the rearrangement of the products immediately following the electron transfer event. This has been successfully used to describe the reactions of aryl halides with 'super electron donors'.[22]

$$\Delta G^{\ddagger} \approx (\lambda_i/4) \cdot (1 + \Delta G/\lambda_i)^2 \quad \text{(S1)}$$

$$\lambda_i = (1/2) \cdot (\lambda_i(\text{NiP4+}) + \lambda_i(\text{ArX})) \quad \text{(S2)}$$

$$\lambda_i\ (\text{species}) = (E_N(R_C) - E_N(R_N)) + (E_C(R_N) - E_C(R_C)) \quad \text{(S3)}$$

- $\lambda_i$ is the internal reorganisation energy; $\lambda_i$(NiP4$^+$) is the internal reorganisation energy of [Ni($PMe_3$)$_4$]$^+$; and $\lambda_i$(ArX) is the internal reorganisation energy of ArX radical anion
- $\Delta G$ is the free energy change of the reaction
- $E_N(R_N)$ and $E_C(R_C)$ are the energies of the neutral and charged species in their respective geometries; $E_C(R_N)$ and $E_N(R_C)$ are the single point energies of the neutral and charged geometries as charged and neutral species, respectively.

While the initial result with [Ni($PMe_3$)]$_4$ and PhI in THF was sensible ($\Delta G^{\ddagger}$ = 15.6 kcal mol$^{-1}$), barriers for PhBr and in toluene and hexane were far too high to account for the $Ni^I$ products observed, albeit in reduced quantities, in these reactions (see Table S2). The major contributor to these energies was the very large value of $\Delta G$ for this process in non-polar solvents.

**Table S2.** Calculated energies for outer-sphere electron transfer from [Ni($PMe_3$)$_4$] to ArX.

| **Solvent** | **Aryl Halide** | **ΔG** (kcal/mol) | **$\lambda_i$** (kcal/mol) | **$\Delta G^{\ddagger}$** (kcal/mol) |
|---|---|---|---|---|
| THF | PhI | 1.2 | 59.9 | 15.6 |
| THF | PhBr | 11.8 | 63.6 | 22.3 |
| Toluene | PhI | 29.8 | 56.8 | 33.0 |
| Hexane | PhI | 38.3 | 61.6 | 40.5 |

### *(B) Inner-sphere electron transfer from [Ni($PMe_3$)$_3$] to ArX*

Inner-sphere electron transfer from [Ni($PMe_3$)$_3$] to ArX could occur *via* complex [Ni($PMe_3$)$_3$(XAr)], with a concomitant change in multiplicity from singlet to triplet. This cannot be described by a simple transition state, and instead requires the geometry at which the singlet and triplet have the same energy to be located, termed the minimum energy crossing point (MECP).

The software tool published by Harvey was used, and the MECP could be located for a number of examples. However, the energies of MECPs for aryl bromides were too high to compete with oxidative addition to [Ni($PMe_3$)$_2$] (see Table S3). Energies are quoted as a range, because these structures typically have imaginary frequencies when either or both of the singlet and triplet frequency calculations are performed; there is a small discrepancy in their free energies, because the MECP calculation seeks the geometry at which the *potential energy E* is the same for both singlet and triplet.

**Table S3.** Calculated minimum energy crossing points for inner-sphere electron transfer from [Ni($PMe_3$)$_4$] to ArX.

| **Solvent** | **Aryl Halide** | **MECP $G_{rel}$** (kcal/mol) |
|---|---|---|
| THF | PhI | 15.0 – 15.2 |
| THF | PhBr | 22.5 – 22.6 |
| Toluene | PhI | 16.5 – 16.8 |
| Toluene | PhBr | 23.9 – 24.6 |
| Hexane | PhI | 16.7 – 17.1 |
| Hexane | PhBr | 24.7 – 26.0 |

### *(C) Oxidative Addition Pathway from [Ni(PMe$_3$)$_2$]*

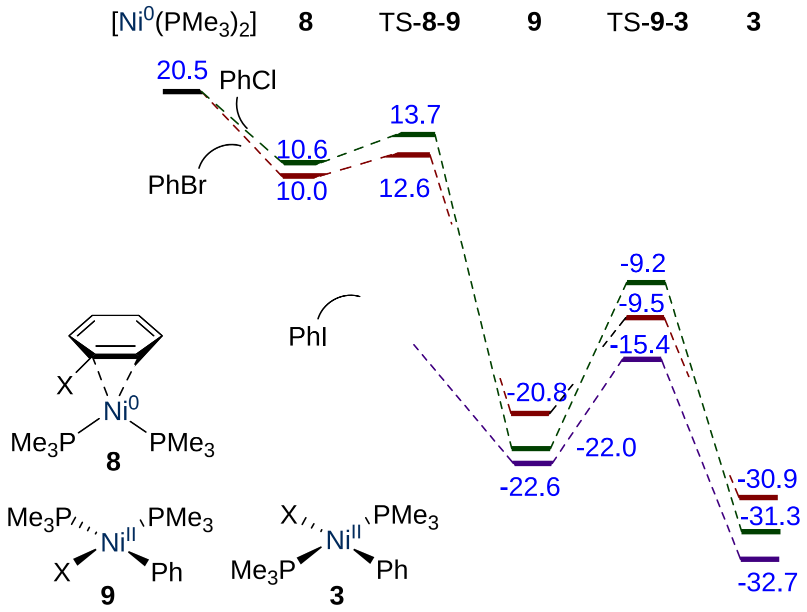


**Figure S2.** Oxidative addition of PhI (purple), PhBr (brown), and PhCl (green) to [Ni(PMe$_3$)$_2$]. Energies are in kcal mol$^{-1}$.

The dissociation energy of the second phosphine is always higher that the oxidative addition transition state from [Ni(PMe$_3$)$_3$], so this mechanism is unfavourable in the three cases. We could not find the barrier for PhI oxidative addition, due to the high reactivity of this species with Ni(P)$_2$, which forms the Ni(II) product **9** directly.

# MICROKINETIC MODEL

## *(A) Reaction between [Ni(PMe$_3$)$_4$] to PhI in toluene*

We used the COPASI program package to run the reactions used in the microkinetic model, with the following equations:

$$\underset{\mathbf{A}}{[Ni^0(PMe_3)_4]} + \underset{\mathbf{B}}{PhI} \xrightarrow{k_1} \underset{\mathbf{C}}{[Ni^{II}(PMe_3)_3Ph]^{+}I^{-}} + \underset{\mathbf{D}}{PMe_3} \quad (1)$$

$$\underset{\mathbf{A}}{[Ni^0(PMe_3)_4]} + \underset{\mathbf{B}}{PhI} \xrightarrow{k_2} \underset{\mathbf{E}}{[Ni^{I}(PMe_3)_3I]} + \underset{\mathbf{F}}{Ph^{\cdot}} + \underset{\mathbf{D}}{PMe_3} \quad (2)$$

$$\underset{\mathbf{E}}{[Ni^{I}(PMe_3)_3I]} \underset{k_{-3}}{\overset{k_3}{\rightleftharpoons}} \underset{\mathbf{G}}{[Ni^{I}(PMe_3)_2I]} + \underset{\mathbf{D}}{PMe_3} \quad (3)$$

$$\underset{\mathbf{G}}{[Ni^{I}(PMe_3)_2I]} + \underset{\mathbf{F}}{Ph^{\cdot}} \xrightarrow{k_4} \underset{\mathbf{H}}{[Ni^{II}(PMe_3)_2I(Ph)]} \quad (4)$$

$$\underset{\mathbf{F}}{Ph^{\cdot}} + \underset{\mathbf{I}}{Ph\text{-}CH_3} \xrightarrow{k_5} \underset{\mathbf{J}}{PhH} + \underset{\mathbf{K}}{PhCH_2^{\cdot}} \quad (5)$$

The activation energies and the associated kinetic constants are in Table 4

**Table S4.** Calculated activation free energies and kinetic constants of kinetic model of the reaction between [Ni(PMe$_3$)$_4$] and PhI in toluene.

| Reaction | $\Delta G^{\ddagger}$ (kcal/mol) | Kinetic constant ($M^{-1}\cdot s$) |
|---|---|---|
| **1** | 16.7 | 3.514 |
| **2** | 9.4 | 7.932e+5 |
| **3** | 10.0 | 2.784e+5 |
| **3($k_{-3}$)** | 3.7 | 1.18e+10 |
| **4**[1] | 3.7 | 1.18e+10 |
| **5** | 10.6 | 1.046e+5 |

The reactions 3 and 4 are barrierless. For this reason, we considered them as diffusion-controlled, with the rate constant given by the equation $k_{diff} = 8k_bT/(3\eta)\cdot 1000\cdot N_a$, in $M^{-1}\cdot s$.[23] The viscosity of toluene at 298 K is 0.560 mPa·s.[24] The diffusion rate constant would correspond to an associated activation free energy of 3.7 kcal/mol, calculated from Eyring equation.

The conditions used to analyze the time course of the reaction were 0.01s in 20 steps, obtaining the results in Table S5. The initial concentrations were taken from the experiments (0.005 M for species

A and B and solvent concentration calculated from the density at 25ºC (12.2 M for THF). The numbers represent the concentration of labelled species in mol per litre (Table S5).

**Table S5.** Concentrations of different species in solution during reaction time in mol/L for the reaction between [Ni(PMe$_3$)$_4$] and PhI in toluene.

| Time (s) | A | B | C | D | E | F | G | H | I | J | K |
|---|---|---|---|---|---|---|---|---|---|---|---|
| 0 | 0.005 | 0.005 | 0 | 0 | 0 | 0 | 0 | 0 | 9.39 | 0 | 0 |
| 0.0005 | 1.68E-03 | 1.68E-03 | 1.47E-08 | 3.88E-03 | 2.76E-03 | 1.89E-06 | 1.68E-05 | 5.44E-04 | 9.39E+00 | 2.78E-03 | 2.78E-03 |
| 0.001 | 1.01E-03 | 1.01E-03 | 1.77E-08 | 4.67E-03 | 3.32E-03 | 6.83E-07 | 1.68E-05 | 6.57E-04 | 9.39E+00 | 3.34E-03 | 3.34E-03 |
| 0.0015 | 7.20E-04 | 7.20E-04 | 1.90E-08 | 5.00E-03 | 3.56E-03 | 3.49E-07 | 1.68E-05 | 7.05E-04 | 9.39E+00 | 3.58E-03 | 3.58E-03 |
| 0.002 | 5.60E-04 | 5.60E-04 | 1.97E-08 | 5.19E-03 | 3.69E-03 | 2.11E-07 | 1.68E-05 | 7.32E-04 | 9.39E+00 | 3.71E-03 | 3.71E-03 |
| 0.0025 | 4.58E-04 | 4.58E-04 | 2.01E-08 | 5.31E-03 | 3.78E-03 | 1.41E-07 | 1.68E-05 | 7.49E-04 | 9.39E+00 | 3.79E-03 | 3.79E-03 |
| 0.003 | 3.88E-04 | 3.88E-04 | 2.04E-08 | 5.39E-03 | 3.83E-03 | 1.01E-07 | 1.68E-05 | 7.61E-04 | 9.39E+00 | 3.85E-03 | 3.85E-03 |
| 0.0035 | 3.36E-04 | 3.36E-04 | 2.07E-08 | 5.45E-03 | 3.88E-03 | 7.60E-08 | 1.68E-05 | 7.69E-04 | 9.39E+00 | 3.89E-03 | 3.89E-03 |
| 0.004 | 2.96E-04 | 2.96E-04 | 2.08E-08 | 5.50E-03 | 3.91E-03 | 5.91E-08 | 1.68E-05 | 7.76E-04 | 9.39E+00 | 3.93E-03 | 3.93E-03 |
| 0.0045 | 2.65E-04 | 2.65E-04 | 2.10E-08 | 5.53E-03 | 3.94E-03 | 4.74E-08 | 1.68E-05 | 7.81E-04 | 9.39E+00 | 3.95E-03 | 3.95E-03 |
| 0.005 | 2.40E-04 | 2.40E-04 | 2.11E-08 | 5.56E-03 | 3.96E-03 | 3.88E-08 | 1.68E-05 | 7.86E-04 | 9.39E+00 | 3.97E-03 | 3.97E-03 |
| 0.0055 | 2.19E-04 | 2.19E-04 | 2.12E-08 | 5.59E-03 | 3.97E-03 | 3.23E-08 | 1.68E-05 | 7.89E-04 | 9.39E+00 | 3.99E-03 | 3.99E-03 |
| 0.006 | 2.02E-04 | 2.02E-04 | 2.13E-08 | 5.61E-03 | 3.99E-03 | 2.74E-08 | 1.68E-05 | 7.92E-04 | 9.39E+00 | 4.01E-03 | 4.01E-03 |
| 0.0065 | 1.87E-04 | 1.87E-04 | 2.13E-08 | 5.62E-03 | 4.00E-03 | 2.35E-08 | 1.68E-05 | 7.95E-04 | 9.39E+00 | 4.02E-03 | 4.02E-03 |
| 0.007 | 1.74E-04 | 1.74E-04 | 2.14E-08 | 5.64E-03 | 4.01E-03 | 2.03E-08 | 1.68E-05 | 7.97E-04 | 9.39E+00 | 4.03E-03 | 4.03E-03 |
| 0.0075 | 1.63E-04 | 1.63E-04 | 2.14E-08 | 5.65E-03 | 4.02E-03 | 1.78E-08 | 1.68E-05 | 7.99E-04 | 9.39E+00 | 4.04E-03 | 4.04E-03 |
| 0.008 | 1.53E-04 | 1.53E-04 | 2.15E-08 | 5.66E-03 | 4.03E-03 | 1.57E-08 | 1.68E-05 | 8.00E-04 | 9.39E+00 | 4.05E-03 | 4.05E-03 |
| 0.0085 | 1.44E-04 | 1.44E-04 | 2.15E-08 | 5.67E-03 | 4.04E-03 | 1.40E-08 | 1.68E-05 | 8.02E-04 | 9.39E+00 | 4.05E-03 | 4.05E-03 |
| 0.009 | 1.36E-04 | 1.36E-04 | 2.15E-08 | 5.68E-03 | 4.04E-03 | 1.25E-08 | 1.68E-05 | 8.03E-04 | 9.39E+00 | 4.06E-03 | 4.06E-03 |
| 0.0095 | 1.29E-04 | 1.29E-04 | 2.16E-08 | 5.69E-03 | 4.05E-03 | 1.12E-08 | 1.68E-05 | 8.04E-04 | 9.39E+00 | 4.07E-03 | 4.07E-03 |
| 0.01 | 1.23E-04 | 1.23E-04 | 2.16E-08 | 5.70E-03 | 4.05E-03 | 1.02E-08 | 1.68E-05 | 8.05E-04 | 9.39E+00 | 4.07E-03 | 4.07E-03 |

The ratio of Ni(I) vs Ni(II) (83:17 in this case) was obtained using the following formula:

$$\frac{[Ni(I)]}{[Ni(II)]} = \frac{[PhH]}{[[Ni(II)(PMe_3)_3Ph]^+I^-] + [Ni(II)(PMe_3)_2(Ph)I]]}$$

## *(B) Reaction between [Ni(PMe$_3$)$_4$] to PhBr in THF*

We ran the same microkinetic model for the reaction between Ni complex and PhBr in THF. The reactions were:

$$\underset{\mathbf{A}}{[Ni^0(PMe_3)_4]} + \underset{\mathbf{B}}{PhBr} \xrightarrow{k_1} \underset{\mathbf{C}}{[Ni^{II}(PMe_3)_3Ph]^+Br^-} + \underset{\mathbf{D}}{PMe_3} \qquad (1)$$

$$\underset{\mathbf{A}}{[Ni^0(PMe_3)_4]} + \underset{\mathbf{B}}{PhBr} \xrightarrow{k_2} \underset{\mathbf{E}}{[Ni^{I}(PMe_3)_3Br]} + \underset{\mathbf{F}}{Ph^{\cdot}} + \underset{\mathbf{D}}{PMe_3} \qquad (2)$$

$$\underset{\mathbf{E}}{[Ni^{I}(PMe_3)_3Br]} \underset{k_{-3}}{\overset{k_3}{\rightleftharpoons}} \underset{\mathbf{G}}{[Ni^{I}(PMe_3)_2Br]} + \underset{\mathbf{D}}{PMe_3} \qquad (3)$$

$$\underset{\mathbf{G}}{[Ni^{I}(PMe_3)_2Br]} + \underset{\mathbf{F}}{Ph^{\cdot}} \xrightarrow{k_4} \underset{\mathbf{H}}{[Ni^{II}(PMe_3)_2I(Ph)]} \qquad (4)$$

$$\underset{\mathbf{F}}{Ph^{\cdot}} + \underset{\mathbf{I}}{Ph\text{-}CH_3} \xrightarrow{k_5} \underset{\mathbf{J}}{PhH} + \underset{\mathbf{K}}{PhCH_2^{\cdot}} \qquad (5)$$

The activation energies and the associated kinetic constants are in Table S6:

**Table S6.** Calculated activation free energies and kinetic constants of kinetic model of the reaction between [Ni(PMe$_3$)$_4$] and PhBr in THF.

| Reaction | $\Delta G^{\ddagger}$ (kcal/mol) | Kinetic constant ($M^{-1}\cdot s$) |
|---|---|---|
| **1** | 15.0 | 62.015 |
| **2** | 16.3 | 6.904 |
| **3** | 6.0 | 2.471e+8 |
| **3($k_{-3}$)** | 3.6 | 1.45e+10 |
| **4**[1] | 3.6 | 1.45e+10 |
| **5** | 9.5 | 6.700e+5 |

The reactions 3 and 4 are also barrierless. We applied the same methodology that above, using the experimental value of 0.456 mPa·s for the viscosity of THF.[25] The associated activation free energy was calculated from Eyring equation and is 3.6 kcal/mol.

**Table S7.** Concentrations of different species in solution along reaction time in mol/L for the reaction between [$Ni(PMe_3)_4$] and PhBr in THF.

| Time (s) | A | B | C | D | E | F | G | H | I | J | K | |
|---|---|---|---|---|---|---|---|---|---|---|---|---|
| 0 | 0.005 | 0.005 | 0 | 0 | 0 | 0 | 0 | 0 | 12.22 | 0 | 0 | |
| 9 | 1.22E-03 | 1.22E-03 | 3.40E-03 | 4.10E-03 | 5.94E-05 | 8.72E-13 | 2.47E-04 | 7.23E-05 | 1.22E+01 | 3.06E-04 | 3.06E-04 | |
| 18 | 6.94E-04 | 6.94E-04 | 3.87E-03 | 4.66E-03 | 7.36E-05 | 2.75E-13 | 2.69E-04 | 8.88E-05 | 1.22E+01 | 3.43E-04 | 3.43E-04 | |
| 27 | 4.85E-04 | 4.85E-04 | 4.06E-03 | 4.89E-03 | 7.95E-05 | 1.33E-13 | 2.77E-04 | 9.56E-05 | 1.22E+01 | 3.57E-04 | 3.57E-04 | |
| 36 | 3.73E-04 | 3.73E-04 | 4.16E-03 | 5.01E-03 | 8.27E-05 | 7.83E-14 | 2.81E-04 | 9.93E-05 | 1.22E+01 | 3.64E-04 | 3.64E-04 | |
| 45 | 3.03E-04 | 3.03E-04 | 4.23E-03 | 5.08E-03 | 8.47E-05 | 5.15E-14 | 2.84E-04 | 1.02E-04 | 1.22E+01 | 3.69E-04 | 3.69E-04 | |
| 54 | 2.55E-04 | 2.55E-04 | 4.27E-03 | 5.13E-03 | 8.61E-05 | 3.64E-14 | 2.86E-04 | 1.03E-04 | 1.22E+01 | 3.72E-04 | 3.72E-04 | |
| 63 | 2.20E-04 | 2.20E-04 | 4.30E-03 | 5.17E-03 | 8.72E-05 | 2.71E-14 | 2.87E-04 | 1.04E-04 | 1.22E+01 | 3.74E-04 | 3.74E-04 | |
| 72 | 1.94E-04 | 1.94E-04 | 4.32E-03 | 5.20E-03 | 8.79E-05 | 2.10E-14 | 2.88E-04 | 1.05E-04 | 1.22E+01 | 3.76E-04 | 3.76E-04 | |
| 81 | 1.73E-04 | 1.73E-04 | 4.34E-03 | 5.22E-03 | 8.85E-05 | 1.67E-14 | 2.89E-04 | 1.06E-04 | 1.22E+01 | 3.78E-04 | 3.78E-04 | |
| 90 | 1.56E-04 | 1.56E-04 | 4.36E-03 | 5.24E-03 | 8.90E-05 | 1.36E-14 | 2.90E-04 | 1.07E-04 | 1.22E+01 | 3.79E-04 | 3.79E-04 | |
| 99 | 1.42E-04 | 1.42E-04 | 4.37E-03 | 5.25E-03 | 8.94E-05 | 1.13E-14 | 2.90E-04 | 1.07E-04 | 1.22E+01 | 3.80E-04 | 3.80E-04 | |
| 108 | 1.31E-04 | 1.31E-04 | 4.38E-03 | 5.27E-03 | 8.98E-05 | 9.53E-15 | 2.91E-04 | 1.07E-04 | 1.22E+01 | 3.80E-04 | 3.80E-04 | |
| 117 | 1.21E-04 | 1.21E-04 | 4.39E-03 | 5.28E-03 | 9.01E-05 | 8.15E-15 | 2.91E-04 | 1.08E-04 | 1.22E+01 | 3.81E-04 | 3.81E-04 | |
| 126 | 1.13E-04 | 1.13E-04 | 4.40E-03 | 5.29E-03 | 9.03E-05 | 7.05E-15 | 2.91E-04 | 1.08E-04 | 1.22E+01 | 3.81E-04 | 3.81E-04 | |
| 135 | 1.05E-04 | 1.05E-04 | 4.40E-03 | 5.29E-03 | 9.05E-05 | 6.16E-15 | 2.91E-04 | 1.08E-04 | 1.22E+01 | 3.82E-04 | 3.82E-04 | |
| 144 | 9.88E-05 | 9.88E-05 | 4.41E-03 | 5.30E-03 | 9.07E-05 | 5.43E-15 | 2.92E-04 | 1.09E-04 | 1.22E+01 | 3.82E-04 | 3.82E-04 | |
| 153 | 9.31E-05 | 9.31E-05 | 4.42E-03 | 5.31E-03 | 9.09E-05 | 4.82E-15 | 2.92E-04 | 1.09E-04 | 1.22E+01 | 3.83E-04 | 3.83E-04 | |
| 162 | 8.80E-05 | 8.80E-05 | 4.42E-03 | 5.31E-03 | 9.11E-05 | 4.30E-15 | 2.92E-04 | 1.09E-04 | 1.22E+01 | 3.83E-04 | 3.83E-04 | |
| 171 | 8.34E-05 | 8.34E-05 | 4.42E-03 | 5.32E-03 | 9.12E-05 | 3.87E-15 | 2.92E-04 | 1.09E-04 | 1.22E+01 | 3.83E-04 | 3.83E-04 | |
| 180 | 7.93E-05 | 7.93E-05 | 4.43E-03 | 5.32E-03 | 9.13E-05 | 3.50E-15 | 2.92E-04 | 1.09E-04 | 1.22E+01 | 3.84E-04 | 3.84E-04 | |

The conditions that are used to analyze the time course of the reaction were 180 s in 20 steps, obtaining the Table S7. The initial concentrations were taken from the experiments (0.005 M for species A and B and solvent concentration calculated from the density at 25ºC (12.2 M for THF). The numbers represent the concentration of labelled species in mol per litre.

The ratio of Ni(I) vs Ni(II) (8:92 in this case) was obtained using the following formula:

$$\frac{[Ni(I)]}{[Ni(II)]} = \frac{[PhH]}{[[Ni(II)(PMe_3)_3Ph]^{+}Br^{-}] + [Ni(II)(PMe_3)_2(Ph)Br]]}$$

# COORDINATES AND ENERGIES FOR MINIMA AND TRANSITION STATES

*Energies are given in Hartrees and correspond to the potential energy with the large basis set (first line) and to the free energy correction (second line). The energies used to compute all the profiles were calculated by the sum of E + $G_{corr}$. In the case of open-shell singlet structures, the values of the potential energy at triplet electronic structure and the $S^2$ of the open-shell singlet are also provided.*

*Coordinates are Cartesian coordinates in Å.*

## In Tetrahydrofuran

### Ni(PMe$_3$)$_4$

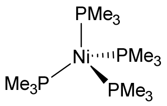


E = -2014.13817957

$G_{corr}$ = 0.403583

| | | | |
|---|---|---|---|
| Ni | 2.97381100 | 7.49675000 | 11.85409900 |
| P | 1.37271500 | 8.84467500 | 11.13266300 |
| P | 2.20232100 | 6.27155600 | 13.51844500 |
| C | 0.89366400 | 10.25111400 | 12.25430200 |
| C | -0.32028500 | 8.16982600 | 10.75440800 |
| C | 1.64237000 | 9.81235900 | 9.56509700 |
| C | 2.55471800 | 6.82320300 | 15.26175000 |
| C | 0.36359500 | 6.01879000 | 13.68224400 |
| C | 2.73487100 | 4.49313900 | 13.67924800 |
| H | 0.06919600 | 10.85075300 | 11.84660400 |
| H | 1.76215600 | 10.90192100 | 12.40339800 |
| H | -0.99638400 | 8.94063300 | 10.36095200 |
| H | -0.76101600 | 7.74569400 | 11.66173000 |
| H | 1.69654200 | 9.12553800 | 8.71509500 |
| H | 2.59494300 | 10.35007200 | 9.62310200 |
| H | 2.17994800 | 7.84212700 | 15.40599100 |
| H | 3.63677800 | 6.83117300 | 15.43343700 |
| H | -0.12463500 | 6.98511600 | 13.85086800 |
| H | 0.11165200 | 5.34883700 | 14.51507000 |
| H | 2.35690800 | 3.92165300 | 12.82559900 |
| H | 2.36069900 | 4.03111600 | 14.60259900 |
| P | 4.57490600 | 8.84467500 | 12.57553600 |
| P | 3.74530100 | 6.27155500 | 10.18975400 |
| C | 5.05395800 | 10.25111300 | 11.45389700 |
| C | 6.26790600 | 8.16982500 | 12.95379100 |
| C | 4.30525200 | 9.81235900 | 14.14310200 |
| C | 3.39290300 | 6.82320300 | 8.44644900 |
| C | 5.58402600 | 6.01879000 | 10.02595500 |
| C | 3.21275000 | 4.49313900 | 10.02895100 |
| H | 5.87842600 | 10.85075200 | 11.86159500 |
| H | 4.18546600 | 10.90192100 | 11.30480100 |
| H | 6.94400500 | 8.94063200 | 13.34724800 |
| H | 6.70863700 | 7.74569400 | 12.04646900 |
| H | 4.25107900 | 9.12553800 | 14.99310400 |
| H | 3.35267900 | 10.35007200 | 14.08509700 |
| H | 3.76767300 | 7.84212700 | 8.30220800 |
| H | 2.31084300 | 6.83117300 | 8.27476200 |
| H | 6.07225600 | 6.98511600 | 9.85733000 |
| H | 5.83596900 | 5.34883700 | 9.19312900 |
| H | 3.59071400 | 3.92165300 | 10.88260000 |
| H | 3.58692200 | 4.03111600 | 9.10560000 |
| H | 0.59726200 | 9.85542400 | 13.23208300 |
| H | -0.23262500 | 7.36424900 | 10.01614700 |
| H | 0.83498600 | 10.53369400 | 9.38176000 |
| H | 3.86048500 | 6.16419300 | 7.70282700 |
| H | 5.97907600 | 5.59522600 | 10.95569700 |
| H | 2.11850700 | 4.43465200 | 10.03648300 |
| H | 3.82911400 | 4.43465200 | 13.67171600 |
| H | -0.03145400 | 5.59522600 | 12.75250200 |
| H | 2.08713600 | 6.16419300 | 16.00537200 |
| H | 5.11263600 | 10.53369400 | 14.32643800 |
| H | 5.35036000 | 9.85542400 | 10.47611600 |
| H | 6.18024600 | 7.36424900 | 13.69205200 |

### Ni(PMe$_3$)$_3$

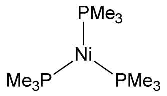


E = -1552.92475226

$G_{corr}$ = 0.291517

| | | | |
|---|---|---|---|
| Ni | 2.96964300 | 7.43814400 | 11.73948900 |
| P | 1.78588200 | 9.17165100 | 11.21717900 |
| P | 2.68132400 | 6.67241600 | 13.74450700 |
| C | 1.79598400 | 10.58439200 | 12.42628900 |
| C | -0.04181500 | 8.86861400 | 11.07777300 |
| C | 2.06963900 | 10.11393500 | 9.63767100 |
| C | 3.57495300 | 7.68797300 | 15.01798000 |
| C | 0.97902400 | 6.63470300 | 14.49173100 |
| C | 3.25422900 | 4.96917000 | 14.22969100 |
| H | 1.15551600 | 11.41351500 | 12.09851300 |
| H | 2.82005700 | 10.95505800 | 12.54912700 |
| H | -0.59829000 | 9.79323900 | 10.87757200 |
| H | -0.40559000 | 8.42471400 | 12.01072500 |
| H | 1.96570000 | 9.43010700 | 8.78771700 |
| H | 3.09258400 | 10.50757700 | 9.63084000 |

| | | | |
|---|---|---|---|
| H | 3.22238500 | 8.72419700 | 14.96670900 |
| H | 4.64846300 | 7.68635400 | 14.79843400 |
| H | 0.55792400 | 7.64612100 | 14.49004800 |
| H | 0.98845100 | 6.25528300 | 15.52202300 |
| H | 2.72224900 | 4.22273200 | 13.62852200 |
| H | 3.08263200 | 4.75512200 | 15.29300900 |
| P | 4.45383700 | 6.54153700 | 10.44821300 |
| C | 4.60327800 | 7.07980900 | 8.67297700 |
| C | 6.21303400 | 6.77898100 | 10.99831900 |
| C | 4.42707200 | 4.69818700 | 10.21154500 |
| H | 4.80172900 | 8.15715900 | 8.64422400 |
| H | 3.65177000 | 6.90058000 | 8.15895700 |
| H | 6.43056300 | 7.85029900 | 11.07367200 |
| H | 6.92678200 | 6.32004700 | 10.30218900 |
| H | 4.50401700 | 4.20697500 | 11.18799600 |
| H | 5.24597500 | 4.35075900 | 9.56835400 |
| H | 1.44614000 | 10.22958800 | 13.40207200 |
| H | -0.23565700 | 8.15585000 | 10.26833200 |
| H | 1.36788200 | 10.94931000 | 9.51041000 |
| H | 5.40333300 | 6.55470500 | 8.13409900 |
| H | 6.34660700 | 6.33642200 | 11.99147800 |
| H | 3.47314100 | 4.40221900 | 9.76000300 |
| H | 4.32488100 | 4.87366400 | 14.01640900 |
| H | 0.32692000 | 5.99561100 | 13.88540800 |
| H | 3.41884200 | 7.30404700 | 16.03434000 |

**Ni(PMe$_3$)$_2$**

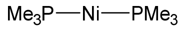


E = -1091.70993065

$G_{corr}$ = 0.185935

| | | | |
|---|---|---|---|
| Ni | 2.97940200 | 7.45613300 | 11.84238900 |
| P | 1.82760700 | 9.14688900 | 11.21331900 |
| P | 4.14634600 | 5.79697500 | 12.52380600 |
| C | 1.88491500 | 10.63783100 | 12.31406000 |
| C | -0.00276200 | 8.91183500 | 11.03663200 |
| C | 2.24526000 | 9.90515700 | 9.57415300 |
| C | 5.40931900 | 6.15031900 | 13.83403500 |
| C | 3.23801200 | 4.37491100 | 13.29076200 |
| C | 5.18415800 | 4.91079900 | 11.26996800 |
| H | 1.26026200 | 11.45018600 | 11.92222000 |
| H | 2.91861800 | 10.99001700 | 12.39950100 |
| H | -0.49668400 | 9.83947600 | 10.72147200 |
| H | -0.42422300 | 8.58776900 | 11.99427500 |
| H | 2.14679100 | 9.15199800 | 8.78486600 |
| H | 3.28488800 | 10.25016200 | 9.58631900 |
| H | 4.91251800 | 6.56083300 | 14.71975700 |
| H | 6.12164400 | 6.89689400 | 13.46665300 |
| H | 2.65731000 | 4.73180200 | 14.14814100 |
| H | 3.92965900 | 3.59271500 | 13.62725900 |
| H | 4.54245400 | 4.51572800 | 10.47505300 |
| H | 5.74143700 | 4.08265400 | 11.72527000 |
| H | 1.53426800 | 10.36600100 | 13.31554000 |
| H | -0.20289600 | 8.13003700 | 10.29592800 |
| H | 1.58780900 | 10.75335400 | 9.34658600 |
| H | 5.89276500 | 5.61284900 | 10.81737700 |
| H | 2.54108800 | 3.94792700 | 12.56145500 |
| H | 5.95561300 | 5.24216700 | 14.11834500 |

**Ni(PMe$_3$)**

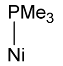


E = -630.471631181

$G_{corr}$ = 0.081831

| | | | |
|---|---|---|---|
| Ni | 3.06794400 | 7.21941500 | 11.85670300 |
| P | 4.21742000 | 5.68285000 | 12.55093100 |
| C | 5.63580600 | 6.13312300 | 13.65447800 |
| C | 3.35958800 | 4.39542600 | 13.56937000 |
| C | 5.06362100 | 4.63561000 | 11.27789100 |
| H | 5.25920200 | 6.65545900 | 14.54008500 |
| H | 6.31509500 | 6.80683700 | 13.12206300 |
| H | 2.90370800 | 4.86361200 | 14.44790400 |
| H | 4.06244000 | 3.61987800 | 13.89765000 |
| H | 4.31558300 | 4.19467400 | 10.61075800 |
| H | 5.64465900 | 3.83317600 | 11.74898300 |
| H | 5.73209000 | 5.25981700 | 10.67574400 |
| H | 2.56361100 | 3.93212800 | 12.97710200 |
| H | 6.18841900 | 5.23976400 | 13.97105400 |

**PMe$_3$**

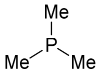


E = -461.173291179

$G_{corr}$ = 0.084310

| | | | |
|---|---|---|---|
| P | 1.68046600 | -0.11497800 | -0.01298200 |
| C | 2.55738000 | 0.74229000 | -1.41387900 |
| H | 2.39880900 | 0.18731300 | -2.34576300 |
| H | 2.14691600 | 1.74954600 | -1.54897100 |
| H | 3.63703800 | 0.82342500 | -1.23338400 |
| C | 2.37105300 | 0.85390800 | 1.41780600 |
| H | 1.97873600 | 1.87728700 | 1.39604500 |
| H | 2.05802300 | 0.39463300 | 2.36254700 |
| H | 3.46755700 | 0.89693200 | 1.39420500 |
| C | 2.73435100 | -1.64354700 | 0.11651900 |
| H | 2.46686700 | -2.20396100 | 1.01978800 |
| H | 2.55202200 | -2.29355600 | -0.74702300 |
| H | 3.80469200 | -1.40360800 | 0.15536100 |

**Ni(PMe$_2$Ph)$_4$**

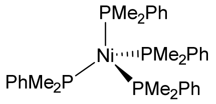


E = -2781.32169362

$G_{corr}$ = 0.601644

| | | | |
|---|---|---|---|
| Ni | -0.49706400 | 0.85226400 | -0.02368300 |
| P | 0.01251600 | 0.52395400 | -2.15370000 |
| P | -1.49145900 | -0.97581000 | 0.70533100 |
| P | 1.42367600 | 1.04561800 | 1.05462400 |
| P | -1.80334900 | 2.60498900 | 0.24991400 |
| C | 1.44599200 | 1.50065700 | -2.82277500 |
| C | 1.58777700 | 2.82186900 | -2.36496000 |
| H | 0.87764400 | 3.20673000 | -1.64199200 |
| C | 2.63543100 | 3.63129200 | -2.80649600 |
| H | 2.71366800 | 4.65272600 | -2.44247100 |
| C | 3.58266900 | 3.12311500 | -3.70047800 |
| H | 4.40850900 | 3.74499500 | -4.03692100 |
| C | 3.46370500 | 1.80709300 | -4.15414400 |
| H | 4.19855600 | 1.40170300 | -4.84581900 |
| C | 2.40074000 | 1.00476600 | -3.72544200 |
| H | 2.33039800 | -0.01259800 | -4.09841600 |
| C | -1.25265400 | 0.87681700 | -3.47378900 |
| H | -2.14416600 | 0.27210800 | -3.27470100 |
| H | -0.87875100 | 0.64356400 | -4.47868900 |
| H | -1.53122900 | 1.93466600 | -3.43637800 |
| C | 0.45981000 | -1.21197800 | -2.63527700 |
| H | -0.37075600 | -1.86256600 | -2.34484200 |
| H | 1.35010000 | -1.52974500 | -2.08220500 |
| H | 0.63660500 | -1.33981400 | -3.70983400 |
| C | -2.50142700 | -1.91250700 | -0.54081700 |
| C | -2.31480100 | -3.26828100 | -0.85570700 |
| H | -1.57269000 | -3.85922200 | -0.32841100 |
| C | -3.07014800 | -3.88223000 | -1.86133600 |
| H | -2.90079400 | -4.93073300 | -2.09493400 |
| C | -4.03654600 | -3.15737200 | -2.56063700 |
| H | -4.62264800 | -3.63567300 | -3.34112900 |
| C | -4.23998400 | -1.80810100 | -2.25135500 |
| H | -4.98763600 | -1.23121700 | -2.79067800 |
| C | -3.47235500 | -1.19386600 | -1.26174200 |
| H | -3.61022300 | -0.13670100 | -1.05729600 |
| C | -2.75428100 | -0.82552000 | 2.06837600 |
| H | -3.51881900 | -0.09823100 | 1.77663000 |
| H | -2.25326300 | -0.45766700 | 2.97021900 |
| H | -3.23954100 | -1.78240600 | 2.29665000 |
| C | -0.43652600 | -2.32011200 | 1.43071300 |
| H | 0.12415400 | -1.89283200 | 2.26718100 |
| H | 0.28595900 | -2.66586800 | 0.68385200 |
| H | -1.01630100 | -3.17576000 | 1.79752000 |
| C | 1.44013600 | 0.45618400 | 2.81741300 |
| C | 0.30475800 | 0.73595300 | 3.59775500 |
| H | -0.52847300 | 1.25974500 | 3.13913100 |
| C | 0.22032500 | 0.32062800 | 4.92731500 |
| H | -0.66813800 | 0.55082500 | 5.51070500 |
| C | 1.26816400 | -0.40728100 | 5.50010800 |
| H | 1.20116200 | -0.74593800 | 6.53083900 |
| C | 2.39923900 | -0.70153700 | 4.73529000 |
| H | 3.21762700 | -1.26989600 | 5.17095000 |
| C | 2.48802200 | -0.26730000 | 3.40832700 |
| H | 3.38110100 | -0.50650400 | 2.83910300 |
| C | 2.87279200 | 0.14066400 | 0.32591400 |
| H | 2.67036100 | -0.93561000 | 0.32112100 |
| H | 3.81634100 | 0.32944400 | 0.85153700 |
| H | 2.98546300 | 0.47259700 | -0.71091700 |
| C | 2.22105300 | 2.71353100 | 1.30194400 |
| H | 1.56058100 | 3.34809800 | 1.90097300 |
| H | 2.36848500 | 3.18911900 | 0.32705200 |
| H | 3.18804700 | 2.62963300 | 1.81337100 |
| C | -1.27360600 | 4.17381100 | -0.58110800 |
| C | -1.68428800 | 4.47886500 | -1.89111400 |
| H | -2.41726800 | 3.85324000 | -2.39192000 |
| C | -1.15463300 | 5.57511400 | -2.57544900 |
| H | -1.48480700 | 5.78616900 | -3.58968800 |
| C | -0.20459800 | 6.39671000 | -1.96340500 |
| H | 0.21126500 | 7.24728800 | -2.49691000 |
| C | 0.20256300 | 6.11578200 | -0.65577600 |
| H | 0.93473800 | 6.75183500 | -0.16397400 |
| C | -0.32601500 | 5.01744000 | 0.02559000 |
| H | 0.01588100 | 4.81218700 | 1.03524600 |
| C | -2.13437100 | 3.22646400 | 1.97428100 |
| H | -2.63347800 | 2.42605600 | 2.53154900 |
| H | -2.77333900 | 4.11784700 | 1.98271800 |
| H | -1.19547800 | 3.45594400 | 2.48616600 |
| C | -3.57308600 | 2.49774500 | -0.32230300 |
| H | -4.07770900 | 1.73805200 | 0.28448000 |
| H | -3.61967100 | 2.17864900 | -1.36763600 |
| H | -4.10777600 | 3.44907700 | -0.21241800 |

**$Ni(PMe_2Ph)_3$**

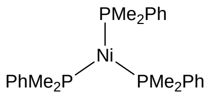


E = -2128.30803416

$G_{corr}$ = 0.439835

| | | | |
|---|---|---|---|
| Ni | 4.19121200 | 6.76005000 | 4.08091800 |
| P | 5.21356600 | 6.50865700 | 2.18812400 |
| P | 3.70034400 | 4.96200500 | 5.16838900 |
| P | 3.31464100 | 8.64088100 | 4.66770700 |
| C | 6.68591700 | 7.57572700 | 1.85295000 |
| C | 6.49986900 | 8.95486200 | 1.64500700 |
| H | 5.49876400 | 9.37361800 | 1.63613700 |
| C | 7.59107200 | 9.80313500 | 1.45629400 |
| H | 7.41970500 | 10.86406400 | 1.29357800 |
| C | 8.89359000 | 9.29463600 | 1.48605800 |
| H | 9.74396400 | 9.95655200 | 1.34369600 |
| C | 9.09260500 | 7.92948300 | 1.70288900 |
| H | 10.10067900 | 7.52261500 | 1.72875300 |
| C | 7.99902900 | 7.07694100 | 1.88293000 |
| H | 8.17977200 | 6.01839900 | 2.04575100 |
| C | 4.12410700 | 6.89228000 | 0.73193300 |
| H | 3.25517000 | 6.22592500 | 0.77775400 |
| H | 4.64020300 | 6.75115700 | -0.22508300 |
| H | 3.76255500 | 7.92237500 | 0.80240100 |

| | | | |
|---|---|---|---|
| C | 5.81532500 | 4.82768500 | 1.67522000 |
| H | 4.94587500 | 4.16063700 | 1.66110100 |
| H | 6.52223500 | 4.43315300 | 2.41211200 |
| H | 6.28582100 | 4.82312800 | 0.68440700 |
| C | 2.50137300 | 4.12490600 | 4.04086200 |
| C | 2.73753100 | 2.88131400 | 3.43361500 |
| H | 3.62787700 | 2.31038700 | 3.68055900 |
| C | 1.83537700 | 2.36088600 | 2.49967600 |
| H | 2.03598600 | 1.39666800 | 2.03877000 |
| C | 0.68182700 | 3.07127400 | 2.16035900 |
| H | -0.01761400 | 2.66506000 | 1.43438200 |
| C | 0.43354400 | 4.31049900 | 2.76076700 |
| H | -0.46152100 | 4.87194800 | 2.50382100 |
| C | 1.33703100 | 4.83351600 | 3.68571200 |
| H | 1.14903700 | 5.80738000 | 4.13095800 |
| C | 2.74468600 | 4.98610600 | 6.76604500 |
| H | 1.86803700 | 5.63111800 | 6.64512000 |
| H | 3.37865000 | 5.41874900 | 7.54881800 |
| H | 2.41552000 | 3.98804300 | 7.08093200 |
| C | 4.93349100 | 3.63721800 | 5.56454500 |
| H | 5.61014300 | 4.02794900 | 6.33300400 |
| H | 5.53219200 | 3.41112200 | 4.67655600 |
| H | 4.46763000 | 2.71773700 | 5.93874700 |
| C | 3.70642200 | 10.14398700 | 3.65906700 |
| C | 3.04912200 | 10.35546900 | 2.43268700 |
| H | 2.24552000 | 9.69080800 | 2.12557300 |
| C | 3.42355200 | 11.40147100 | 1.58756400 |
| H | 2.89956600 | 11.54712800 | 0.64595700 |
| C | 4.46988600 | 12.25759600 | 1.94755400 |
| H | 4.76493200 | 13.06996900 | 1.28863800 |
| C | 5.13203600 | 12.05764900 | 3.16120800 |
| H | 5.94820600 | 12.71491500 | 3.45114400 |
| C | 4.75423800 | 11.01173200 | 4.00775800 |
| H | 5.29363000 | 10.86913900 | 4.94007700 |
| C | 3.64599800 | 9.24085100 | 6.39382600 |
| H | 3.21338400 | 8.50797000 | 7.08415100 |
| H | 3.20760800 | 10.22661600 | 6.58971000 |
| H | 4.72283900 | 9.27883200 | 6.58541300 |
| C | 1.45496100 | 8.69570800 | 4.67491100 |
| H | 1.09255400 | 7.96880000 | 5.40985300 |
| H | 1.06069200 | 8.40972200 | 3.69546300 |
| H | 1.07622300 | 9.68997700 | 4.93916500 |

**Ni(PMe$_2$Ph)$_2$**

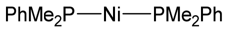


E = -1475.29545173

$G_{corr}$ = 0.28227

| | | | |
|---|---|---|---|
| Ni | 2.92241100 | 7.11859900 | 5.19863800 |
| P | 3.47235300 | 5.05218800 | 5.30225500 |
| P | 2.40999600 | 9.18799300 | 5.00635800 |
| C | 2.11340800 | 3.83268800 | 5.01141200 |
| C | 2.32833800 | 2.44477700 | 5.01571100 |
| H | 3.32405800 | 2.04670600 | 5.19499900 |
| C | 1.27041100 | 1.56232400 | 4.79070400 |
| H | 1.44994400 | 0.49012600 | 4.79674500 |
| C | -0.01768100 | 2.05566300 | 4.55728500 |
| H | -0.84021400 | 1.36703600 | 4.38190200 |
| C | -0.24309100 | 3.43400300 | 4.54980300 |
| H | -1.24185700 | 3.82310600 | 4.36866100 |
| C | 0.81737400 | 4.31589400 | 4.77552800 |
| H | 0.65178300 | 5.39162200 | 4.77119100 |
| C | 4.21624900 | 4.43587200 | 6.88614000 |
| H | 3.49925300 | 4.56974200 | 7.70273900 |
| H | 5.10810500 | 5.03064700 | 7.11204500 |
| H | 4.49844100 | 3.37864200 | 6.82494500 |
| C | 4.76298600 | 4.49912500 | 4.08951200 |
| H | 5.66612400 | 5.10065000 | 4.24023900 |
| H | 4.40248200 | 4.67022500 | 3.07013300 |
| H | 5.01517600 | 3.43931400 | 4.20944800 |
| C | 3.83168800 | 10.35892800 | 4.84879700 |
| C | 3.65586400 | 11.74603700 | 4.71739000 |
| H | 2.65526400 | 12.17093400 | 4.70650000 |
| C | 4.75923800 | 12.59286600 | 4.59918200 |
| H | 4.61005900 | 13.66494600 | 4.49808800 |
| C | 6.05430600 | 12.06388800 | 4.61090400 |
| H | 6.91236200 | 12.72481400 | 4.51901000 |
| C | 6.24110700 | 10.68584400 | 4.74103300 |
| H | 7.24509300 | 10.26925700 | 4.75062900 |
| C | 5.13512600 | 9.83966600 | 4.85908900 |
| H | 5.26999500 | 8.76438200 | 4.96032200 |
| C | 1.41358700 | 9.95775500 | 6.36900800 |
| H | 0.47799100 | 9.39765500 | 6.47613900 |
| H | 1.17894100 | 11.00892900 | 6.16680900 |
| H | 1.96654800 | 9.88717000 | 7.31131800 |
| C | 1.37048500 | 9.64012100 | 3.53769000 |
| H | 0.43129400 | 9.07790900 | 3.58412100 |
| H | 1.89456300 | 9.35605500 | 2.61936600 |
| H | 1.14435600 | 10.71207600 | 3.50680400 |

**Ni(PMe$_2$Ph)**

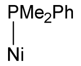


E = -822.264490723

$G_{corr}$ = 0.130288

| | | | |
|---|---|---|---|
| Ni | 3.64952100 | 7.12531700 | 5.00244100 |
| P | 3.41747500 | 5.11515900 | 5.25861700 |
| C | 2.50041500 | 4.21075400 | 3.92761400 |
| C | 2.24237300 | 2.83206200 | 4.00590600 |
| H | 2.58070100 | 2.26020700 | 4.86635100 |
| C | 1.55079100 | 2.18047800 | 2.98316600 |
| H | 1.35592300 | 1.11348400 | 3.05667000 |
| C | 1.10918500 | 2.89760500 | 1.86640400 |
| H | 0.57117600 | 2.38864500 | 1.07077800 |
| C | 1.36195300 | 4.26833000 | 1.77702400 |
| H | 1.02235200 | 4.83123900 | 0.91130500 |
| C | 2.05274500 | 4.91888500 | 2.80286100 |

| | | | |
|---|---|---|---|
| H | 2.25421400 | 5.98640500 | 2.74311300 |
| C | 2.51599100 | 4.58407600 | 6.79081300 |
| H | 1.50187400 | 4.99570400 | 6.77850700 |
| H | 3.04140400 | 4.98493300 | 7.66408300 |
| H | 2.46104400 | 3.49294700 | 6.87634200 |
| C | 4.97271200 | 4.11402600 | 5.40575700 |
| H | 5.55908800 | 4.49693900 | 6.24767900 |
| H | 5.56528100 | 4.22584500 | 4.49240400 |
| H | 4.75977500 | 3.05136700 | 5.56956000 |

**$PMe_2Ph$**

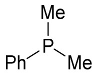


E = -652.964031445

$G_{corr}$ = 0.132457

| | | | |
|---|---|---|---|
| P | -1.30905200 | -0.28950500 | -0.03156300 |
| C | -0.53198400 | 0.56311500 | -1.48545100 |
| C | -1.37168700 | 1.16607600 | -2.43429200 |
| C | 0.85804100 | 0.63792200 | -1.67860600 |
| C | -0.84085600 | 1.82725300 | -3.54607400 |
| H | -2.45046600 | 1.11860000 | -2.30335400 |
| C | 1.39128300 | 1.29448000 | -2.78824400 |
| H | 1.53357900 | 0.18124100 | -0.95957900 |
| C | 0.54176800 | 1.89158400 | -3.72535300 |
| H | -1.50740400 | 2.28884400 | -4.27021800 |
| H | 2.46912700 | 1.34100900 | -2.92329400 |
| H | 0.95767700 | 2.40268700 | -4.58987700 |
| C | -0.37379700 | -1.90028000 | -0.01465200 |
| H | 0.71302900 | -1.75927200 | 0.00532400 |
| H | -0.66727200 | -2.47036700 | 0.87465000 |
| H | -0.63735500 | -2.49165700 | -0.89839400 |
| C | -0.44285900 | 0.57133100 | 1.37596200 |
| H | -0.77915700 | 1.61227200 | 1.43393200 |
| H | -0.70926800 | 0.07564300 | 2.31680100 |
| H | 0.64819800 | 0.55826200 | 1.26825500 |

**$Ni(PMePh_2)_4$**

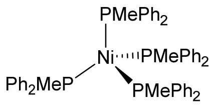


E = -3548.5036579

$G_{corr}$ = 0.801946

| | | | |
|---|---|---|---|
| Ni | 9.59031000 | 10.54489800 | 4.13449400 |
| P | 9.87146800 | 10.62134100 | 1.92237500 |
| C | 8.65761800 | 9.75601500 | 0.81128400 |
| C | 7.52397300 | 10.41534300 | 0.30694100 |
| H | 7.39665700 | 11.48197600 | 0.46109400 |
| C | 6.54045100 | 9.71644600 | -0.39894900 |
| H | 5.67123900 | 10.24924900 | -0.77695700 |
| C | 6.67334100 | 8.34394900 | -0.62267500 |
| H | 5.90719900 | 7.80005300 | -1.16891100 |
| C | 7.80715500 | 7.67943900 | -0.14481600 |
| H | 7.93011500 | 6.61357300 | -0.32113000 |
| C | 8.78660800 | 8.37778900 | 0.56176000 |
| H | 9.65572900 | 7.84194500 | 0.92929300 |
| C | 11.45461400 | 9.91898400 | 1.25612800 |
| C | 11.99216300 | 8.79464100 | 1.90215900 |
| H | 11.47276900 | 8.37774000 | 2.75750000 |
| C | 13.19301600 | 8.22396800 | 1.47436100 |
| H | 13.58519900 | 7.35070600 | 1.98908300 |
| C | 13.88904600 | 8.78522000 | 0.40025000 |
| H | 14.83204400 | 8.35480700 | 0.07299600 |
| C | 13.36400500 | 9.90448100 | -0.25229200 |
| H | 13.89823600 | 10.34717500 | -1.08947700 |
| C | 12.15250700 | 10.46113800 | 0.16566500 |
| H | 11.76819500 | 11.33019100 | -0.35832100 |
| C | 9.87591400 | 12.32571300 | 1.18371100 |
| H | 9.86814100 | 12.32920600 | 0.08888700 |
| H | 10.76620600 | 12.85065800 | 1.54408300 |
| H | 8.99973600 | 12.86941800 | 1.54666400 |
| P | 7.81248900 | 11.83305100 | 4.60153700 |
| C | 6.88495300 | 11.55674000 | 6.18703700 |
| C | 7.19289300 | 12.27375300 | 7.35741600 |
| H | 7.92532000 | 13.07396800 | 7.32413600 |
| C | 6.56370500 | 11.97935300 | 8.56869200 |
| H | 6.81621100 | 12.55309200 | 9.45726000 |
| C | 5.60997500 | 10.95958800 | 8.63816400 |
| H | 5.12125600 | 10.72846600 | 9.58112400 |
| C | 5.28340200 | 10.24876700 | 7.48082200 |
| H | 4.53801400 | 9.45812100 | 7.51504600 |
| C | 5.91044800 | 10.54639600 | 6.26830500 |
| H | 5.63723200 | 9.97716700 | 5.38682800 |
| C | 8.02798800 | 13.67866900 | 4.61893600 |
| C | 7.02729700 | 14.57342300 | 5.03608000 |
| H | 6.09018700 | 14.19366700 | 5.43490000 |
| C | 7.23122300 | 15.95188400 | 4.95652200 |
| H | 6.45167800 | 16.63312600 | 5.28888200 |
| C | 8.43425700 | 16.45814100 | 4.45006800 |
| H | 8.59039500 | 17.53230500 | 4.38983200 |
| C | 9.43076800 | 15.57788100 | 4.02533200 |
| H | 10.37000400 | 15.95608300 | 3.62978700 |
| C | 9.22413000 | 14.19833200 | 4.11366200 |
| H | 9.99025800 | 13.50553100 | 3.78634700 |
| C | 6.39111700 | 11.77595200 | 3.39594600 |
| H | 5.53313300 | 12.35332200 | 3.75680000 |
| H | 6.08146600 | 10.74645900 | 3.20907100 |
| H | 6.73454400 | 12.20670800 | 2.45231900 |
| P | 9.09892300 | 8.55186700 | 5.01251600 |
| C | 10.06402400 | 7.05019800 | 4.49069000 |
| C | 11.24938400 | 6.68408200 | 5.15014200 |
| H | 11.55819300 | 7.20679500 | 6.04895900 |
| C | 12.05244000 | 5.64844000 | 4.66490800 |
| H | 12.96562400 | 5.38580600 | 5.19356100 |
| C | 11.68416900 | 4.95173200 | 3.51131900 |
| H | 12.30995100 | 4.14830900 | 3.13180100 |
| C | 10.49682600 | 5.29246000 | 2.85611000 |

| | | | |
|---|---|---|---|
| H | 10.19120500 | 4.75110900 | 1.96406000 |
| C | 9.69638400 | 6.32742500 | 3.34157400 |
| H | 8.78020600 | 6.57759800 | 2.81638900 |
| C | 7.37130700 | 7.92378600 | 4.75649200 |
| C | 6.74620800 | 8.22139100 | 3.53554100 |
| H | 7.28528100 | 8.79667500 | 2.79217900 |
| C | 5.43729900 | 7.80930800 | 3.27552100 |
| H | 4.97962200 | 8.04980500 | 2.31949200 |
| C | 4.72093300 | 7.10699600 | 4.24827500 |
| H | 3.69593500 | 6.79894400 | 4.05855400 |
| C | 5.33204700 | 6.80239200 | 5.46829200 |
| H | 4.78276400 | 6.25509400 | 6.23054800 |
| C | 6.64900800 | 7.19792200 | 5.71667700 |
| H | 7.09813700 | 6.94726200 | 6.67200100 |
| C | 9.26291100 | 8.44852400 | 6.86142800 |
| H | 9.16184800 | 7.43125400 | 7.25372100 |
| H | 8.49656500 | 9.09003600 | 7.30794900 |
| H | 10.23912900 | 8.84253600 | 7.15600500 |
| P | 11.56535900 | 11.16998300 | 4.99441200 |
| C | 11.61672700 | 11.87597000 | 6.71185900 |
| C | 10.46879900 | 11.75001400 | 7.50153000 |
| H | 9.58387300 | 11.30399300 | 7.06262700 |
| C | 10.45666300 | 12.18307400 | 8.83048600 |
| H | 9.55089000 | 12.07542300 | 9.42177800 |
| C | 11.60269700 | 12.75465100 | 9.38569700 |
| H | 11.59926300 | 13.09771700 | 10.41721900 |
| C | 12.75882400 | 12.88587300 | 8.60726400 |
| H | 13.65445800 | 13.33032000 | 9.03463200 |
| C | 12.76776100 | 12.44598000 | 7.28269100 |
| H | 13.67120000 | 12.55640500 | 6.68876800 |
| C | 12.60161700 | 12.39813600 | 4.06076600 |
| C | 12.51110800 | 13.78019000 | 4.30693300 |
| H | 11.89436100 | 14.14454900 | 5.12211700 |
| C | 13.21153500 | 14.69886700 | 3.52269400 |
| H | 13.12771500 | 15.76143900 | 3.73811700 |
| C | 14.02209200 | 14.25612000 | 2.47320400 |
| H | 14.56551800 | 14.97067100 | 1.86037800 |
| C | 14.13448900 | 12.88573200 | 2.22729800 |
| H | 14.76585300 | 12.52374800 | 1.41976400 |
| C | 13.43555800 | 11.96676600 | 3.01414800 |
| H | 13.54045600 | 10.90950000 | 2.79758600 |
| C | 12.84305600 | 9.83482700 | 5.26077300 |
| H | 13.81909900 | 10.25909200 | 5.52054100 |
| H | 12.94299000 | 9.21083600 | 4.37131400 |
| H | 12.50602700 | 9.20455500 | 6.08760400 |

**Ni(PMePh$_2$)$_3$**

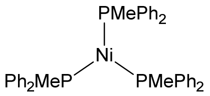


E = -1858.88033969

$G_{corr}$ = 0.588582

| | | | |
|---|---|---|---|
| Ni | 8.95690100 | 10.55166600 | 3.71532900 |
| P | 9.90260700 | 10.75027500 | 1.79241700 |
| C | 8.66182700 | 9.90016100 | 0.71748400 |
| C | 7.48228000 | 10.57100500 | 0.34924700 |
| H | 7.36049700 | 11.62453800 | 0.58287400 |
| C | 6.44941500 | 9.89400100 | -0.30294400 |
| H | 5.54456100 | 10.43042600 | -0.57788100 |
| C | 6.57503600 | 8.53276600 | -0.59668600 |
| H | 5.76898000 | 8.00456500 | -1.09915400 |
| C | 7.74619100 | 7.85739700 | -0.24124900 |
| H | 7.85512300 | 6.79936000 | -0.46658000 |
| C | 8.77969100 | 8.53376500 | 0.41010100 |
| H | 9.67773100 | 7.99322900 | 0.69208600 |
| C | 11.47935000 | 9.87655900 | 1.37160200 |
| C | 12.21113600 | 9.29576800 | 2.41582700 |
| H | 11.83158100 | 9.37301600 | 3.43045100 |
| C | 13.39747300 | 8.60295200 | 2.15802000 |
| H | 13.94583800 | 8.14913100 | 2.97869200 |
| C | 13.86204700 | 8.48097900 | 0.84716000 |
| H | 14.78091200 | 7.93737400 | 0.64209100 |
| C | 13.13851600 | 9.05455500 | -0.20481400 |
| H | 13.49469800 | 8.95733600 | -1.22747500 |
| C | 11.95593300 | 9.74794900 | 0.05585500 |
| H | 11.39354100 | 10.17560400 | -0.77049200 |
| C | 10.16056100 | 12.36692800 | 0.90612000 |
| H | 10.40528300 | 12.24007300 | -0.15405300 |
| H | 10.97971400 | 12.90273100 | 1.39839900 |
| H | 9.25685400 | 12.97720400 | 0.99710400 |
| P | 7.44597800 | 11.91802300 | 4.42499000 |
| C | 6.76683200 | 11.55555900 | 6.10786900 |
| C | 7.35127200 | 12.11038900 | 7.25913200 |
| H | 8.12332900 | 12.86931100 | 7.16089400 |
| C | 6.95253600 | 11.69920800 | 8.53249300 |
| H | 7.41520400 | 12.14345700 | 9.41053200 |
| C | 5.96432000 | 10.72094600 | 8.68038800 |
| H | 5.65685700 | 10.39839200 | 9.67174300 |
| C | 5.37617500 | 10.16114100 | 7.54278100 |
| H | 4.61072800 | 9.39559400 | 7.64282300 |
| C | 5.77390300 | 10.57321900 | 6.26901500 |
| H | 5.31953500 | 10.10683400 | 5.40008900 |
| C | 7.83900700 | 13.72130500 | 4.54925900 |
| C | 6.91053600 | 14.67693800 | 4.99371900 |
| H | 5.92093800 | 14.36242800 | 5.31610700 |
| C | 7.25247000 | 16.02923900 | 5.03573800 |
| H | 6.52564200 | 16.76115200 | 5.37935600 |
| C | 8.52942300 | 16.44295400 | 4.63891600 |
| H | 8.79381700 | 17.49682700 | 4.67261600 |
| C | 9.46252400 | 15.49972100 | 4.20289500 |
| H | 10.45694300 | 15.81534500 | 3.89715400 |
| C | 9.11674800 | 14.14576000 | 4.15891800 |
| H | 9.83290000 | 13.40115500 | 3.81757500 |
| C | 5.89616100 | 11.95461400 | 3.39852100 |
| H | 5.08737300 | 12.51462400 | 3.88039200 |
| H | 5.56254600 | 10.93203700 | 3.20148800 |
| H | 6.13782000 | 12.42280100 | 2.43985500 |
| P | 8.94024800 | 8.69131700 | 4.83412000 |

| | | | |
|---|---|---|---|
| C | 10.10321700 | 7.31188900 | 4.41387100 |
| C | 11.28554100 | 7.09996000 | 5.14207000 |
| H | 11.48730900 | 7.67734800 | 6.04026500 |
| C | 12.22549700 | 6.15492900 | 4.71960900 |
| H | 13.13459900 | 6.00577600 | 5.29738100 |
| C | 12.00066700 | 5.40602300 | 3.56232700 |
| H | 12.73358000 | 4.67497400 | 3.23132900 |
| C | 10.82420900 | 5.60488900 | 2.83266600 |
| H | 10.63631000 | 5.02475200 | 1.93237100 |
| C | 9.88734900 | 6.54852500 | 3.25349800 |
| H | 8.98219900 | 6.69858400 | 2.67199000 |
| C | 7.29046700 | 7.87855700 | 4.60966700 |
| C | 6.58159900 | 8.14374200 | 3.42548800 |
| H | 7.02261100 | 8.79272700 | 2.67453500 |
| C | 5.31334600 | 7.59783400 | 3.21473300 |
| H | 4.78420800 | 7.81024400 | 2.28928600 |
| C | 4.72760900 | 6.79189400 | 4.19519100 |
| H | 3.73591000 | 6.37475000 | 4.03957700 |
| C | 5.42237200 | 6.52489200 | 5.37869100 |
| H | 4.97176200 | 5.89936600 | 6.14533100 |
| C | 6.69741300 | 7.05902500 | 5.58190700 |
| H | 7.22117200 | 6.84029300 | 6.50786700 |
| C | 9.10770000 | 8.78321200 | 6.67987500 |
| H | 9.21766700 | 7.80301200 | 7.15535800 |
| H | 8.22106600 | 9.28307900 | 7.07873700 |
| H | 9.97866000 | 9.39785100 | 6.92725200 |

**Ni(PMePh$_2$)$_2$**

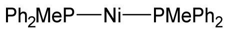


E = -1858.88033969

$G_{corr}$ = 0.378772

| | | | |
|---|---|---|---|
| Ni | -1.41586700 | 1.01000500 | -0.08372400 |
| P | -0.81611000 | 2.12220600 | -1.80758100 |
| C | -0.98230400 | 3.95372700 | -1.63782700 |
| C | -0.13827000 | 4.88889500 | -2.25671500 |
| H | 0.68689900 | 4.55915400 | -2.88069700 |
| C | -0.34634200 | 6.25947000 | -2.07586900 |
| H | 0.31677900 | 6.97276700 | -2.55888100 |
| C | -1.40037900 | 6.71223300 | -1.27878100 |
| H | -1.55958500 | 7.77837900 | -1.13957500 |
| C | -2.24431400 | 5.78901700 | -0.65378100 |
| H | -3.06092100 | 6.13381700 | -0.02466000 |
| C | -2.03092500 | 4.42109400 | -0.82679300 |
| H | -2.67032400 | 3.69751600 | -0.32430600 |
| C | -1.77848200 | 1.74749400 | -3.34441400 |
| C | -2.49248400 | 0.54034900 | -3.39614000 |
| H | -2.48598400 | -0.11209800 | -2.52493100 |
| C | -3.20412300 | 0.18436200 | -4.54468500 |
| H | -3.75252000 | -0.75384400 | -4.57229500 |
| C | -3.21602500 | 1.03761400 | -5.65086500 |
| H | -3.77473700 | 0.76655600 | -6.54298100 |
| C | -2.51245700 | 2.24569200 | -5.60583500 |
| H | -2.52325400 | 2.91437600 | -6.46293300 |
| C | -1.79628400 | 2.59812300 | -4.46043300 |
| H | -1.25764900 | 3.54188800 | -4.43366800 |
| C | 0.91831900 | 1.88965600 | -2.41947500 |
| H | 1.10625600 | 2.40968700 | -3.36447600 |
| H | 1.07329600 | 0.81703000 | -2.57483000 |
| H | 1.62747400 | 2.23525500 | -1.66000100 |
| P | -1.95177400 | -0.18643300 | 1.60445500 |
| C | -1.47310700 | 0.54269700 | 3.23203500 |
| C | -2.19297600 | 0.36951500 | 4.42430000 |
| H | -3.10710200 | -0.21642000 | 4.43700000 |
| C | -1.74285300 | 0.95040300 | 5.61383600 |
| H | -2.31071900 | 0.80920600 | 6.53002300 |
| C | -0.56882300 | 1.70716100 | 5.62787600 |
| H | -0.22096000 | 2.15642100 | 6.55442200 |
| C | 0.15204700 | 1.89196400 | 4.44404200 |
| H | 1.06115700 | 2.48782900 | 4.44513300 |
| C | -0.30222100 | 1.32045000 | 3.25529400 |
| H | 0.24186600 | 1.47969800 | 2.32611100 |
| C | -1.14655700 | -1.85454600 | 1.63149400 |
| C | -0.66773100 | -2.37221900 | 0.41779700 |
| H | -0.75350700 | -1.76657400 | -0.48237600 |
| C | -0.08517000 | -3.64104700 | 0.36645900 |
| H | 0.28097300 | -4.03043200 | -0.58014200 |
| C | 0.03375100 | -4.40189000 | 1.53215300 |
| H | 0.49304000 | -5.38640000 | 1.49665800 |
| C | -0.43439100 | -3.89149400 | 2.74729100 |
| H | -0.33871900 | -4.47849300 | 3.65730800 |
| C | -1.02240500 | -2.62606600 | 2.79727300 |
| H | -1.37613200 | -2.23689100 | 3.74847700 |
| C | -3.73027700 | -0.65480000 | 1.83537800 |
| H | -3.88189600 | -1.35099100 | 2.66685800 |
| H | -4.06875600 | -1.13597800 | 0.91195800 |
| H | -4.33073000 | 0.24665000 | 1.99621500 |

**Ni(PMePh$_2$)**

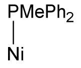


E = -844.755968904

$G_{corr}$ = 0.178995

| | | | |
|---|---|---|---|
| Ni | 9.31802800 | 9.48396300 | 3.82312600 |
| P | 9.72794500 | 10.33446600 | 2.02123500 |
| C | 8.42705100 | 10.05837000 | 0.73423500 |
| C | 7.94132500 | 11.06150900 | -0.11810100 |
| H | 8.31932700 | 12.07672600 | -0.04968100 |
| C | 6.95838200 | 10.76881400 | -1.06938600 |
| H | 6.58988100 | 11.55719200 | -1.72090700 |
| C | 6.45325900 | 9.47247100 | -1.18429300 |
| H | 5.69007500 | 9.24760500 | -1.92475200 |
| C | 6.92830900 | 8.46587800 | -0.33665000 |
| H | 6.53529300 | 7.45547100 | -0.41527900 |
| C | 7.90023900 | 8.75998000 | 0.61876400 |
| H | 8.25500800 | 7.98338200 | 1.29309200 |
| C | 11.26492300 | 9.74579400 | 1.16536100 |

| | | | |
|---|---|---|---|
| C | 12.23804500 | 9.06480600 | 1.91103100 |
| H | 12.05139900 | 8.86885000 | 2.96491300 |
| C | 13.42679200 | 8.64118700 | 1.31102300 |
| H | 14.17296300 | 8.11468700 | 1.90070400 |
| C | 13.65071500 | 8.88889900 | -0.04533100 |
| H | 14.57196500 | 8.55529400 | -0.51590600 |
| C | 12.68391500 | 9.56301600 | -0.79862300 |
| H | 12.85300700 | 9.75423800 | -1.85534100 |
| C | 11.49832500 | 9.98957200 | -0.19765400 |
| H | 10.75046900 | 10.50554800 | -0.79465300 |
| C | 9.99733500 | 12.16858900 | 1.98247300 |
| H | 10.28291100 | 12.52489200 | 0.98698300 |
| H | 10.80643000 | 12.39927600 | 2.68235700 |
| H | 9.09249300 | 12.68575700 | 2.31715400 |

**$PMePh_2$**

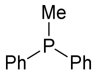


E = -844.755968904

$G_{corr}$ = 0.18076

| | | | |
|---|---|---|---|
| P | -1.19882300 | -0.44166600 | 0.01868500 |
| C | -0.58479000 | 0.57761700 | -1.40342600 |
| C | -1.47307300 | 0.88000500 | -2.44556800 |
| C | 0.74095200 | 1.03645900 | -1.49012200 |
| C | -1.04780700 | 1.61868600 | -3.55421100 |
| H | -2.50452300 | 0.53933200 | -2.39059700 |
| C | 1.16584100 | 1.77564100 | -2.59375900 |
| H | 1.43995200 | 0.82193600 | -0.68572200 |
| C | 0.27155500 | 2.06731000 | -3.62953000 |
| H | -1.74831300 | 1.84574300 | -4.35376400 |
| H | 2.19379100 | 2.12549900 | -2.64718800 |
| H | 0.60313200 | 2.64495300 | -4.48856200 |
| C | -0.11094700 | -1.94065500 | -0.16172500 |
| H | 0.95659900 | -1.69504300 | -0.15159400 |
| H | -0.32582500 | -2.65911500 | 0.63622400 |
| H | -0.35467700 | -2.41836200 | -1.11669300 |
| C | -0.41203600 | 0.43923800 | 1.44440300 |
| C | -0.68192000 | 1.80931500 | 1.61881300 |
| C | 0.37817000 | -0.21224400 | 2.40447600 |
| C | -0.16435100 | 2.50833900 | 2.70825600 |
| H | -1.29061100 | 2.33641400 | 0.88747400 |
| C | 0.89057200 | 0.48787900 | 3.50132300 |
| H | 0.60612700 | -1.26938100 | 2.30535400 |
| C | 0.62499100 | 1.84896900 | 3.65600200 |
| H | -0.37697400 | 3.56885400 | 2.81787500 |
| H | 1.50352000 | -0.03450100 | 4.23175200 |
| H | 1.02759600 | 2.39243100 | 4.50672700 |

**$Ni(PPh_3)_4$**

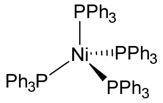


E = -4315.67341777

$G_{corr}$ = 1.003185

| | | | |
|---|---|---|---|
| Ni | 9.57524700 | 10.55790800 | 4.18916900 |
| P | 9.74169400 | 10.45242400 | 1.90715400 |
| C | 8.53392100 | 9.40000600 | 0.95056500 |
| C | 7.20132900 | 9.82337500 | 0.79988600 |
| H | 6.89640200 | 10.78685300 | 1.18440400 |
| C | 6.25769000 | 9.02256900 | 0.15706600 |
| H | 5.23780900 | 9.38327400 | 0.05198800 |
| C | 6.62110800 | 7.76800900 | -0.34081200 |
| H | 5.88574300 | 7.13831900 | -0.83494300 |
| C | 7.94071300 | 7.33516500 | -0.20063300 |
| H | 8.24142800 | 6.36431300 | -0.58689500 |
| C | 8.88900800 | 8.14462200 | 0.43205600 |
| H | 9.90641300 | 7.78320700 | 0.52467500 |
| C | 11.31920400 | 9.80690200 | 1.15607700 |
| C | 12.01138800 | 8.80919800 | 1.85456500 |
| H | 11.63893000 | 8.48367200 | 2.81665400 |
| C | 13.18271600 | 8.25121600 | 1.33802400 |
| H | 13.69565400 | 7.47460600 | 1.89848700 |
| C | 13.69371800 | 8.70529500 | 0.12048500 |
| H | 14.61294100 | 8.28491400 | -0.27949400 |
| C | 13.01786800 | 9.70955000 | -0.58044200 |
| H | 13.41079600 | 10.07142800 | -1.52749400 |
| C | 11.83410600 | 10.24965300 | -0.07302000 |
| H | 11.31931900 | 11.02273100 | -0.63442700 |
| P | 7.90869500 | 12.08454900 | 4.60375300 |
| C | 6.97656700 | 11.91024700 | 6.22232200 |
| C | 6.88228000 | 12.93310200 | 7.18269500 |
| H | 7.31152800 | 13.90944800 | 6.99003300 |
| C | 6.25293900 | 12.71091800 | 8.41127100 |
| H | 6.20299300 | 13.51871200 | 9.13745000 |
| C | 5.69101900 | 11.46719100 | 8.70628200 |
| H | 5.20030800 | 11.29806500 | 9.66121000 |
| C | 5.77500000 | 10.44155000 | 7.76225400 |
| H | 5.35677800 | 9.46056600 | 7.97308000 |
| C | 6.42212000 | 10.66020000 | 6.54598900 |
| H | 6.51146000 | 9.83628500 | 5.85189400 |
| C | 8.32641100 | 13.90442000 | 4.67465900 |
| C | 7.34002800 | 14.90812400 | 4.64396400 |
| H | 6.29121900 | 14.63589600 | 4.57576000 |
| C | 7.69125200 | 16.25713400 | 4.69820000 |
| H | 6.91390600 | 17.01658900 | 4.66932800 |
| C | 9.03637500 | 16.63238700 | 4.78842300 |
| H | 9.30839800 | 17.68423300 | 4.82330300 |
| C | 10.02332200 | 15.64725600 | 4.83195300 |
| H | 11.07418100 | 15.91689600 | 4.89522900 |
| C | 9.66225200 | 14.29892500 | 4.77700300 |
| H | 10.43170300 | 13.54238500 | 4.79555600 |

| | | | |
|---|---|---|---|
| P | 9.10187700 | 8.45462600 | 4.94328600 |
| C | 9.97374500 | 7.02050600 | 4.13351700 |
| C | 11.29676700 | 6.69449900 | 4.48063700 |
| H | 11.78736300 | 7.22239400 | 5.28677400 |
| C | 11.99370800 | 5.69473800 | 3.80103500 |
| H | 13.01383200 | 5.46341200 | 4.09634500 |
| C | 11.38744000 | 5.00530500 | 2.74726100 |
| H | 11.93141100 | 4.23191500 | 2.21113600 |
| C | 10.07393500 | 5.31864000 | 2.39286200 |
| H | 9.58689700 | 4.79020600 | 1.57700700 |
| C | 9.37210600 | 6.31123100 | 3.08184400 |
| H | 8.35406600 | 6.53522500 | 2.78507200 |
| C | 7.35442200 | 7.82605500 | 4.79261100 |
| C | 6.56590400 | 8.35981700 | 3.76640200 |
| H | 6.97809400 | 9.14523600 | 3.14652200 |
| C | 5.26033200 | 7.91190500 | 3.55412700 |
| H | 4.67316800 | 8.34023900 | 2.74676800 |
| C | 4.71535900 | 6.93488700 | 4.38907000 |
| H | 3.69509800 | 6.59157500 | 4.23789500 |
| C | 5.48825400 | 6.40381100 | 5.42733500 |
| H | 5.06929500 | 5.64623500 | 6.08514700 |
| C | 6.80075000 | 6.83822300 | 5.62322200 |
| H | 7.38609000 | 6.40840700 | 6.42979600 |
| P | 11.56165600 | 11.11721200 | 5.21215000 |
| C | 11.52704000 | 11.88361800 | 6.91666400 |
| C | 10.32341100 | 12.37309000 | 7.42892500 |
| H | 9.42534600 | 12.28275000 | 6.83693400 |
| C | 10.25259300 | 12.95785500 | 8.69588900 |
| H | 9.29559600 | 13.31534500 | 9.06655500 |
| C | 11.40401800 | 13.06460100 | 9.47616200 |
| H | 11.35749600 | 13.51271700 | 10.46539100 |
| C | 12.62186100 | 12.58935700 | 8.97656200 |
| H | 13.52615800 | 12.67256100 | 9.57408400 |
| C | 12.68301500 | 12.00708700 | 7.71052800 |
| H | 13.63676100 | 11.64539500 | 7.33847100 |
| C | 12.66811500 | 12.35812200 | 4.34003800 |
| C | 13.06095000 | 13.58249100 | 4.91042000 |
| H | 12.78088300 | 13.82963500 | 5.92754100 |
| C | 13.79818100 | 14.51607000 | 4.17588100 |
| H | 14.07985100 | 15.45699100 | 4.64231800 |
| C | 14.17153800 | 14.24704500 | 2.85766100 |
| H | 14.74458300 | 14.97470400 | 2.28901000 |
| C | 13.79044500 | 13.03563700 | 2.27702600 |
| H | 14.05691600 | 12.80788800 | 1.24815700 |
| C | 13.03894800 | 12.11417200 | 3.00653800 |
| H | 12.71691100 | 11.20727900 | 2.51521500 |
| C | 12.77470400 | 9.73859800 | 5.57844900 |
| C | 12.67234200 | 9.02908000 | 6.79089700 |
| C | 13.75503100 | 9.32878900 | 4.66145700 |
| C | 13.51946400 | 7.95640400 | 7.07364600 |
| H | 11.93121800 | 9.31088900 | 7.52856400 |
| C | 14.59671400 | 8.24846800 | 4.93965300 |
| H | 13.88064100 | 9.84720100 | 3.72003100 |
| C | 14.48520700 | 7.55562100 | 6.14672900 |
| H | 13.41195800 | 7.43014800 | 8.01849700 |
| H | 15.34521500 | 7.95533000 | 4.20773800 |
| H | 15.14255800 | 6.71773000 | 6.36374200 |
| C | 9.42454400 | 8.14170800 | 6.74553100 |
| C | 9.81648700 | 6.91297100 | 7.30148200 |
| C | 9.25062700 | 9.23622200 | 7.60303200 |
| C | 10.05284500 | 6.79781600 | 8.67384600 |
| H | 9.95408600 | 6.04395800 | 6.66655900 |
| C | 9.47351700 | 9.12357200 | 8.97579100 |
| H | 8.96115300 | 10.18715200 | 7.17898200 |
| C | 9.88614500 | 7.90244800 | 9.51498000 |
| H | 10.36998200 | 5.84266000 | 9.08503900 |
| H | 9.34460200 | 9.99467800 | 9.61259800 |
| H | 10.07844000 | 7.81088800 | 10.58091500 |
| C | 6.48936900 | 12.19593500 | 3.38722700 |
| C | 6.61037100 | 13.02592700 | 2.25580600 |
| C | 5.32206000 | 11.42690900 | 3.51417200 |
| C | 5.60321100 | 13.08143200 | 1.29112500 |
| H | 7.49205900 | 13.64053000 | 2.12127300 |
| C | 4.31812100 | 11.47405100 | 2.54302800 |
| H | 5.17790400 | 10.78253500 | 4.37190800 |
| C | 4.45220500 | 12.30056100 | 1.42550500 |
| H | 5.72903500 | 13.73136000 | 0.42922900 |
| H | 3.42636100 | 10.86521600 | 2.66981000 |
| H | 3.66982700 | 12.33878100 | 0.67203500 |
| C | 9.59224400 | 12.08407300 | 1.03130000 |
| C | 10.12815600 | 13.19390900 | 1.69870100 |
| C | 8.99379900 | 12.28302700 | -0.22298500 |
| C | 10.05685300 | 14.47352900 | 1.14776400 |
| H | 10.58464800 | 13.04774900 | 2.66746700 |
| C | 8.90993300 | 13.56457300 | -0.77411100 |
| H | 8.57724000 | 11.44364000 | -0.76962400 |
| C | 9.43619100 | 14.66432600 | -0.08962700 |
| H | 10.46668600 | 15.31676300 | 1.69713400 |
| H | 8.42976200 | 13.70243600 | -1.73979100 |
| H | 9.36191000 | 15.66068600 | -0.51782500 |

**Ni(PPh$_3$)$_3$**

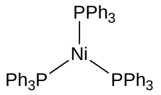


E = -3279.08460787

$G_{corr}$ = 0.735357

| | | | |
|---|---|---|---|
| Ni | 8.54521400 | 10.27566900 | 3.62939900 |
| P | 9.38890200 | 10.48884300 | 1.65003000 |
| C | 8.18658000 | 9.78380600 | 0.43371600 |
| C | 7.92271300 | 10.34415900 | -0.82408100 |
| H | 8.47227500 | 11.21982600 | -1.15604700 |
| C | 6.94597000 | 9.78810300 | -1.65629300 |
| H | 6.74934400 | 10.23612800 | -2.62710400 |
| C | 6.22433600 | 8.66530600 | -1.24403900 |
| H | 5.46333800 | 8.23767700 | -1.89158500 |
| C | 6.48268300 | 8.09636500 | 0.00786100 |
| H | 5.92197500 | 7.22604400 | 0.33935800 |

| | | | |
|---|---|---|---|
| C | 7.45234400 | 8.65417300 | 0.84096400 |
| H | 7.63155300 | 8.23339300 | 1.82776900 |
| C | 10.95421900 | 9.57189200 | 1.28440000 |
| C | 11.96681300 | 9.63308600 | 2.25591000 |
| H | 11.78627900 | 10.18183800 | 3.17769100 |
| C | 13.18068300 | 8.97590500 | 2.06177000 |
| H | 13.95157700 | 9.02601700 | 2.82594400 |
| C | 13.39218700 | 8.22808700 | 0.89866800 |
| H | 14.33155600 | 7.70108800 | 0.75289100 |
| C | 12.38707800 | 8.14981500 | -0.06788800 |
| H | 12.54499200 | 7.56717600 | -0.97222400 |
| C | 11.17572000 | 8.82289900 | 0.12013500 |
| H | 10.40512200 | 8.76466200 | -0.64345800 |
| P | 7.12552800 | 11.69629900 | 4.45904400 |
| C | 5.92922200 | 11.09785400 | 5.74359900 |
| C | 6.42341700 | 10.72140900 | 7.00607600 |
| H | 7.46196900 | 10.90828500 | 7.25740000 |
| C | 5.59485000 | 10.10256100 | 7.94085900 |
| H | 6.00016100 | 9.81771800 | 8.90843700 |
| C | 4.25667800 | 9.83790700 | 7.63086500 |
| H | 3.61359600 | 9.34584800 | 8.35586500 |
| C | 3.75417000 | 10.21040300 | 6.38278400 |
| H | 2.71435800 | 10.01452000 | 6.13238900 |
| C | 4.58234200 | 10.83829400 | 5.44688300 |
| H | 4.17427200 | 11.11504100 | 4.47938700 |
| C | 7.96901300 | 13.10545600 | 5.30704800 |
| C | 7.40462800 | 13.83818300 | 6.36275200 |
| H | 6.41021900 | 13.59240600 | 6.72331500 |
| C | 8.11980000 | 14.87532100 | 6.96668500 |
| H | 7.67233100 | 15.43154600 | 7.78651200 |
| C | 9.40510800 | 15.19616600 | 6.52089800 |
| H | 9.96046200 | 16.00159200 | 6.99448000 |
| C | 9.97518500 | 14.47485200 | 5.46778700 |
| H | 10.97593300 | 14.71580100 | 5.11840900 |
| C | 9.26321100 | 13.43406300 | 4.86899100 |
| H | 9.70959000 | 12.85636100 | 4.06374600 |
| P | 9.05279500 | 8.60018700 | 4.91027800 |
| C | 10.45809000 | 7.46459600 | 4.49561900 |
| C | 11.59952800 | 7.30476100 | 5.29227400 |
| H | 11.68002600 | 7.83072000 | 6.23912600 |
| C | 12.64119300 | 6.46592700 | 4.87947700 |
| H | 13.52183900 | 6.35387400 | 5.50739300 |
| C | 12.54896400 | 5.77083900 | 3.67293200 |
| H | 13.35884200 | 5.12047500 | 3.35278900 |
| C | 11.41154300 | 5.92359600 | 2.87129700 |
| H | 11.33722000 | 5.39708400 | 1.92355700 |
| C | 10.38242200 | 6.77021100 | 3.27498400 |
| H | 9.51507000 | 6.90500300 | 2.63327800 |
| C | 7.70169400 | 7.36439500 | 5.18441600 |
| C | 6.39155900 | 7.74437900 | 4.85597900 |
| H | 6.21598300 | 8.73364900 | 4.44808400 |
| C | 5.32350400 | 6.86586200 | 5.05064400 |
| H | 4.31457400 | 7.18151800 | 4.79853400 |
| C | 5.55511700 | 5.58925000 | 5.56819600 |
| H | 4.72645000 | 4.90202300 | 5.71872600 |
| C | 6.85867900 | 5.19418900 | 5.88629900 |
| H | 7.04496800 | 4.19928600 | 6.28300400 |
| C | 7.92557200 | 6.07398400 | 5.69353900 |
| H | 8.93530200 | 5.75415900 | 5.93570900 |
| C | 9.49035900 | 9.19104500 | 6.60796000 |
| C | 9.15024000 | 8.53349200 | 7.79820400 |
| C | 10.12710900 | 10.44203400 | 6.68292100 |
| C | 9.42787100 | 9.12283600 | 9.03548700 |
| H | 8.64633400 | 7.57264300 | 7.76619700 |
| C | 10.41070500 | 11.02824700 | 7.91623200 |
| H | 10.36122600 | 10.97235700 | 5.76373300 |
| C | 10.05458100 | 10.37034500 | 9.09820700 |
| H | 9.14678700 | 8.60802200 | 9.95086700 |
| H | 10.88627000 | 12.00493100 | 7.95258700 |
| H | 10.25847700 | 10.83070800 | 10.06150700 |
| C | 5.98866100 | 12.51927900 | 3.24632700 |
| C | 5.50258900 | 13.82811000 | 3.37923900 |
| C | 5.63781400 | 11.77678700 | 2.10680300 |
| C | 4.69013900 | 14.38344000 | 2.38666500 |
| H | 5.76615100 | 14.42234700 | 4.24956000 |
| C | 4.81384400 | 12.32473000 | 1.12300700 |
| H | 6.03854100 | 10.77504400 | 1.98186600 |
| C | 4.34270200 | 13.63399200 | 1.25892200 |
| H | 4.32960200 | 15.40349300 | 2.49427700 |
| H | 4.55973500 | 11.73600100 | 0.24535100 |
| H | 3.71394400 | 14.07084000 | 0.48734100 |
| C | 9.77915100 | 12.15450300 | 0.92525900 |
| C | 10.84143500 | 12.39242300 | 0.03835900 |
| C | 8.95482300 | 13.23097300 | 1.29046700 |
| C | 11.06987600 | 13.67502300 | -0.46820600 |
| H | 11.49410500 | 11.57823800 | -0.26143300 |
| C | 9.17501000 | 14.50939300 | 0.77677400 |
| H | 8.13720100 | 13.06340300 | 1.98114300 |
| C | 10.23740600 | 14.73627700 | -0.10278600 |
| H | 11.89927100 | 13.84298000 | -1.15083200 |
| H | 8.52114500 | 15.32624400 | 1.07191600 |
| H | 10.41792300 | 15.73287300 | -0.49752100 |

**Ni(PPh$_3$)$_2$**

Ph$_3$P—Ni—PPh$_3$

E = -2242.46752288

$G_{corr}$ = 0.477374

| | | | |
|---|---|---|---|
| Ni | -1.29396100 | 1.93989600 | -0.01465100 |
| P | -2.05630200 | 3.89533300 | -0.39166100 |
| C | -2.90685900 | 4.68036000 | 1.04850900 |
| C | -2.92276700 | 6.06035900 | 1.30058700 |
| H | -2.40181000 | 6.74269100 | 0.63516600 |
| C | -3.60553500 | 6.56620100 | 2.41017600 |
| H | -3.60949100 | 7.63690600 | 2.59783800 |
| C | -4.27870200 | 5.70093400 | 3.27677500 |
| H | -4.80640700 | 6.09736800 | 4.14033600 |
| C | -4.26366600 | 4.32376000 | 3.03557600 |
| H | -4.77761800 | 3.64491300 | 3.71121400 |

| | | | |
|---|---|---|---|
| C | -3.57601800 | 3.81677800 | 1.93231600 |
| H | -3.54156300 | 2.74450000 | 1.74904300 |
| C | -0.78470300 | 5.13978400 | -0.88779800 |
| C | -1.04785300 | 6.24955800 | -1.70535000 |
| H | -2.04718800 | 6.41224100 | -2.09864200 |
| C | -0.02687200 | 7.14952800 | -2.02181100 |
| H | -0.24123400 | 8.00564900 | -2.65643800 |
| C | 1.26493800 | 6.95041900 | -1.52691400 |
| H | 2.05764200 | 7.65051300 | -1.77757200 |
| C | 1.53733600 | 5.84367400 | -0.71733100 |
| H | 2.54230200 | 5.67831000 | -0.33770000 |
| C | 0.51954000 | 4.94131000 | -0.40474100 |
| H | 0.72737100 | 4.06599400 | 0.20767900 |
| P | -0.47832600 | 0.01037100 | 0.38061300 |
| C | 1.09461800 | -0.35527400 | -0.51672900 |
| C | 2.10583800 | -1.19260200 | -0.02196100 |
| H | 1.99775700 | -1.66135400 | 0.95189900 |
| C | 3.25894700 | -1.42675100 | -0.77584300 |
| H | 4.03668800 | -2.07642000 | -0.38248100 |
| C | 3.41365400 | -0.82803100 | -2.02913600 |
| H | 4.31276800 | -1.01033300 | -2.61200500 |
| C | 2.41368000 | 0.01287200 | -2.52683800 |
| H | 2.53273900 | 0.48932800 | -3.49655900 |
| C | 1.26451500 | 0.25246300 | -1.77215600 |
| H | 0.49088800 | 0.92178200 | -2.14355500 |
| C | -1.57299200 | -1.41201000 | -0.05621600 |
| C | -2.95735100 | -1.20192800 | 0.06102500 |
| H | -3.31827600 | -0.22294300 | 0.37062900 |
| C | -3.85786300 | -2.22858500 | -0.22596000 |
| H | -4.92633800 | -2.05308900 | -0.13074200 |
| C | -3.38482500 | -3.47489800 | -0.64777600 |
| H | -4.08451000 | -4.27356600 | -0.87978800 |
| C | -2.01011300 | -3.68896200 | -0.77922500 |
| H | -1.63877000 | -4.65463100 | -1.11278900 |
| C | -1.10687800 | -2.66398200 | -0.48463500 |
| H | -0.04054000 | -2.84177500 | -0.59080400 |
| C | -0.05242300 | -0.30101200 | 2.15104400 |
| C | -0.08176900 | -1.56651100 | 2.75636800 |
| C | 0.32370900 | 0.81441600 | 2.91803000 |
| C | 0.27180400 | -1.71296900 | 4.10040400 |
| H | -0.38169000 | -2.43866600 | 2.18255000 |
| C | 0.68179500 | 0.66609100 | 4.25867400 |
| H | 0.32171400 | 1.79866900 | 2.45368900 |
| C | 0.65626500 | -0.59946600 | 4.85234500 |
| H | 0.24485100 | -2.69764900 | 4.56008100 |
| H | 0.97050500 | 1.53730500 | 4.84100100 |
| H | 0.92793100 | -0.71650200 | 5.89823700 |
| C | -3.31162200 | 3.99829400 | -1.74319100 |
| C | -3.18790800 | 3.07364400 | -2.79387100 |
| C | -4.35815000 | 4.93237500 | -1.77651500 |
| C | -4.08031700 | 3.09473600 | -3.86658700 |
| H | -2.39165800 | 2.33260200 | -2.75441900 |
| C | -5.25489300 | 4.94829200 | -2.84821400 |
| H | -4.47739400 | 5.64674900 | -0.96685400 |
| C | -5.11664300 | 4.03286300 | -3.89504300 |
| H | -3.97364100 | 2.37372500 | -4.67299100 |
| H | -6.06270100 | 5.67563100 | -2.86345100 |
| H | -5.81779200 | 4.04572700 | -4.72549300 |

**Ni(PPh$_3$)**

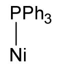


E = -1205.85045751

$G_{corr}$ = 0.227205

| | | | |
|---|---|---|---|
| Ni | 9.72302200 | 9.91145400 | 3.80698600 |
| P | 9.17286800 | 8.33567900 | 4.96691400 |
| C | 10.27997700 | 6.85324400 | 4.86740700 |
| C | 10.52040500 | 5.98916200 | 5.94704100 |
| H | 10.05690100 | 6.17664400 | 6.91123100 |
| C | 11.35824500 | 4.88131700 | 5.79150200 |
| H | 11.53901600 | 4.22090100 | 6.63581900 |
| C | 11.96302800 | 4.62358500 | 4.55864900 |
| H | 12.61647000 | 3.76302300 | 4.44145500 |
| C | 11.73081900 | 5.48040000 | 3.47834600 |
| H | 12.20361500 | 5.28951000 | 2.51834800 |
| C | 10.89974700 | 6.59022700 | 3.63475500 |
| H | 10.73004800 | 7.27163600 | 2.80367600 |
| C | 7.49495200 | 7.63416900 | 4.61273500 |
| C | 6.49773300 | 8.52910500 | 4.19112600 |
| H | 6.75282300 | 9.57855900 | 4.05934100 |
| C | 5.20162400 | 8.08098700 | 3.93428700 |
| H | 4.43993700 | 8.78527700 | 3.60985700 |
| C | 4.88819000 | 6.72655400 | 4.08374700 |
| H | 3.88095300 | 6.37408500 | 3.87726900 |
| C | 5.87569200 | 5.82648700 | 4.49202900 |
| H | 5.63857700 | 4.77158100 | 4.60460100 |
| C | 7.17252900 | 6.27593400 | 4.75609500 |
| H | 7.92975400 | 5.56547400 | 5.07393300 |
| C | 9.10847500 | 8.68008700 | 6.78649000 |
| C | 8.16461000 | 8.11119900 | 7.65485400 |
| C | 10.07543400 | 9.55374800 | 7.31125700 |
| C | 8.19490600 | 8.40486400 | 9.02127000 |
| H | 7.40390300 | 7.43827000 | 7.27069200 |
| C | 10.11028500 | 9.83975800 | 8.67626800 |
| H | 10.79406500 | 10.01296300 | 6.63552100 |
| C | 9.16763900 | 9.26571500 | 9.53510200 |
| H | 7.45646400 | 7.95970600 | 9.68329900 |
| H | 10.86568000 | 10.51605800 | 9.06806900 |
| H | 9.18775600 | 9.49345700 | 10.59765400 |

**PPh$_3$**

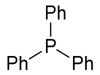


E = -1036.54805321

$G_{corr}$ = 0.228088

| | | | |
|---|---|---|---|
| P | -1.33822500 | -0.20916800 | -0.01106700 |

| | | | |
|---|---|---|---|
| C | -0.49056200 | 0.65826200 | -1.41058800 |
| C | -1.08786100 | 0.55928300 | -2.67884800 |
| C | 0.69563800 | 1.39704900 | -1.27931000 |
| C | -0.50573100 | 1.16860500 | -3.79121200 |
| H | -2.01393800 | 0.00067700 | -2.79755200 |
| C | 1.27202100 | 2.01620100 | -2.39123900 |
| H | 1.17486600 | 1.48781200 | -0.30911600 |
| C | 0.67555600 | 1.90154900 | -3.64912000 |
| H | -0.97867100 | 1.07815100 | -4.76575700 |
| H | 2.19061600 | 2.58559000 | -2.27346300 |
| H | 1.12616600 | 2.38348700 | -4.51284000 |
| C | -0.49628800 | 0.52031500 | 1.46812700 |
| C | -1.04301600 | 1.70211100 | 1.99702100 |
| C | 0.63639000 | -0.03333400 | 2.08465800 |
| C | -0.46285700 | 2.32381300 | 3.10358200 |
| H | -1.92782100 | 2.13988000 | 1.53963200 |
| C | 1.21042300 | 0.58344600 | 3.19898000 |
| H | 1.07550200 | -0.94690200 | 1.69537000 |
| C | 0.66520500 | 1.76377900 | 3.70925100 |
| H | -0.89603700 | 3.23968000 | 3.49746900 |
| H | 2.08692800 | 0.14131200 | 3.66611400 |
| H | 1.11355500 | 2.24207700 | 4.57619200 |
| C | -0.59117500 | -1.90143100 | -0.09580600 |
| C | -1.25582700 | -2.93147000 | 0.59084300 |
| C | 0.58661200 | -2.20728600 | -0.79589000 |
| C | -0.74787500 | -4.23162000 | 0.59154200 |
| H | -2.17622700 | -2.71534000 | 1.12918700 |
| C | 1.08920600 | -3.51047700 | -0.80332800 |
| H | 1.11558700 | -1.42673100 | -1.33446600 |
| C | 0.42562400 | -4.52450000 | -0.10823000 |
| H | -1.27204200 | -5.01638400 | 1.13092100 |
| H | 2.00170300 | -3.73212600 | -1.35122300 |
| H | 0.81808800 | -5.53801100 | -0.11533000 |

**PhI**

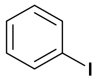


E = -243.107736972

$G_{corr}$ = 0.058614

| | | | |
|---|---|---|---|
| C | 2.37152000 | -0.21742400 | 0.00004400 |
| C | 3.76861100 | -0.23321200 | 0.00051100 |
| C | 4.46008500 | 0.98167600 | -0.00005500 |
| C | 3.76413600 | 2.19271900 | -0.00103700 |
| C | 2.36729000 | 2.19055000 | -0.00148300 |
| C | 1.65935800 | 0.98487600 | -0.00095000 |
| H | 4.31214000 | -1.17186400 | 0.00128900 |
| H | 5.54689700 | 0.97369200 | 0.00029800 |
| H | 4.30771100 | 3.13330800 | -0.00147300 |
| H | 1.81823800 | 3.12843600 | -0.00226000 |
| H | 0.57433100 | 0.98727300 | -0.00129000 |
| I | 1.31057300 | -2.06376000 | 0.00090600 |

**PhBr**

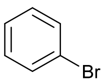


E = -244.896841005

$G_{corr}$ = 0.059764

| | | | |
|---|---|---|---|
| C | -0.85796100 | 0.70377900 | 0.00009600 |
| C | 0.53709300 | 0.72585000 | 0.00058500 |
| C | 1.25386900 | 1.92286100 | -0.00001800 |
| C | 0.54635900 | 3.12815400 | -0.00104900 |
| C | -0.85051400 | 3.13003500 | -0.00149500 |
| C | -1.54762700 | 1.91946500 | -0.00096300 |
| H | -1.39816700 | -0.23693400 | 0.00054500 |
| H | 2.33857700 | 1.91999100 | 0.00033700 |
| H | 1.09606600 | 4.06534300 | -0.00150300 |
| H | -1.39348900 | 4.07076600 | -0.00228700 |
| H | -2.63418700 | 1.91251600 | -0.00132300 |
| Br | 1.50685400 | -0.95204800 | 0.00213300 |

**PhCl**

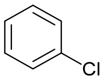


E = -691.950425949

$G_{corr}$ = 0.061491

| | | | |
|---|---|---|---|
| C | -0.85716900 | 0.70386000 | 0.00011200 |
| C | 0.53709400 | 0.72607000 | 0.00061000 |
| C | 1.25354400 | 1.92236600 | -0.00000700 |
| C | 0.54680700 | 3.12722500 | -0.00104300 |
| C | -0.84999300 | 3.12910400 | -0.00148800 |
| C | -1.54697700 | 1.91855900 | -0.00096300 |
| H | -1.39124900 | -0.24059900 | 0.00055500 |
| H | 2.33849700 | 1.91237000 | 0.00034100 |
| H | 1.09591200 | 4.06475600 | -0.00151000 |
| H | -1.39301300 | 4.06980900 | -0.00229500 |
| H | -2.63353100 | 1.91212000 | -0.00133500 |
| Cl | 1.42199500 | -0.80593300 | 0.00190100 |

**PhH**

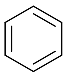

E = -232.324141291

$G_{corr}$ = 0.073358

| | | | |
|---|---|---|---|
| C | -0.58967400 | 0.46982000 | 0.00007300 |
| C | 0.80830200 | 0.46978900 | 0.00059400 |
| C | 1.50730200 | 1.68026600 | -0.00009700 |
| C | 0.80833000 | 2.89101300 | -0.00101000 |
| C | -0.58944100 | 2.89104200 | -0.00141100 |
| C | -1.28854600 | 1.68039600 | -0.00099600 |
| H | -1.13306100 | -0.47145200 | 0.00044300 |
| H | 1.35156700 | -0.47156400 | 0.00138300 |

| | | | |
|---|---|---|---|
| H | 2.59421000 | 1.68036700 | 0.00014600 |
| H | 1.35195200 | 3.83214100 | -0.00144800 |
| H | -1.13292200 | 3.83226200 | -0.00224200 |
| H | -2.37546200 | 1.68056300 | -0.00134700 |

**Ph•**

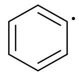

E = -231.634164717

$G_{corr}$ = 0.059620

| | | | |
|---|---|---|---|
| C | 1.08864700 | -0.03769400 | 0.00006900 |
| C | 2.48722700 | -0.02963100 | 0.00046900 |
| C | 3.19370500 | 1.17742900 | -0.00006000 |
| C | 2.50180800 | 2.40134400 | -0.00094100 |
| C | 1.12511300 | 2.33009200 | -0.00131600 |
| C | 0.37481100 | 1.17352800 | -0.00088100 |
| H | 0.54648100 | -0.98062200 | 0.00048300 |
| H | 3.03049000 | -0.97076800 | 0.00119500 |
| H | 4.28145500 | 1.17540900 | 0.00024800 |
| H | 3.03893100 | 3.34652400 | -0.00135700 |
| H | -0.71237300 | 1.18106900 | -0.00119200 |

**THF**

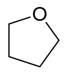


E = -232.533046146

$G_{corr}$ = 0.089224

| | | | |
|---|---|---|---|
| C | -1.56230200 | -0.45607500 | -0.24398700 |
| O | -0.20588500 | -0.90584700 | -0.08615900 |
| C | 0.64395600 | 0.21838200 | 0.19170000 |
| C | -0.17133000 | 1.46792000 | -0.15074900 |
| C | -1.59214100 | 1.00795700 | 0.20704100 |
| H | -1.85390200 | -0.55044800 | -1.30149300 |
| H | -2.22154100 | -1.10312100 | 0.34786000 |
| H | 0.92674500 | 0.21260800 | 1.25650900 |
| H | 1.56037900 | 0.12037000 | -0.40278100 |
| H | 0.15644900 | 2.35268300 | 0.40361300 |
| H | -0.10111600 | 1.68671800 | -1.22326500 |
| H | -1.74999800 | 1.07166800 | 1.29050300 |
| H | -2.37879700 | 1.58492900 | -0.28879800 |

**THF radical**

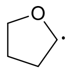


E = -231.874382497

$G_{corr}$ = 0.075589

| | | | |
|---|---|---|---|
| C | -1.24722000 | -0.70316900 | 0.08386900 |
| O | 0.10174200 | -0.69340000 | -0.43563800 |
| C | 0.60453000 | 0.57802300 | -0.27297400 |
| C | -0.47918200 | 1.56038500 | 0.07988100 |
| C | -1.76070900 | 0.72121600 | -0.13708100 |
| H | -1.80208700 | -1.47616200 | -0.45435600 |
| H | -1.22033300 | -0.96284000 | 1.15191900 |
| H | 1.46881700 | 0.78697400 | -0.89743500 |
| H | -0.40745000 | 1.88985200 | 1.12873600 |
| H | -0.45368500 | 2.46651100 | -0.53792000 |
| H | -2.57654100 | 0.98252500 | 0.54294400 |
| H | -2.11662300 | 0.83286300 | -1.16731300 |

**H Abstraction TS: Ph• + THF**

E = -464.165098393

$G_{corr}$ = 0.161901

ν = -902.54 $cm^{-1}$

| | | | |
|---|---|---|---|
| C | -1.83225600 | 0.60865000 | 0.00283100 |
| C | -0.48010100 | 0.98016900 | -0.00381900 |
| C | -0.06053200 | 1.94830700 | -0.90496600 |
| C | -0.92424800 | 2.55791900 | -1.80434300 |
| C | -2.27557300 | 2.18081000 | -1.79230100 |
| C | -2.72469000 | 1.20981800 | -0.89097300 |
| H | -2.18644900 | -0.14621200 | 0.70168700 |
| H | 0.22898300 | 0.52288900 | 0.68344600 |
| H | -0.57330500 | 3.31260600 | -2.50566400 |
| H | -2.97413600 | 2.64468700 | -2.48567200 |
| H | -3.77274300 | 0.92112300 | -0.88592900 |
| C | 2.66362800 | 2.10019400 | -0.67812000 |
| O | 2.88774700 | 1.06445200 | 0.24846100 |
| C | 3.26725100 | 1.66796600 | -2.00501800 |
| H | 2.99522800 | 3.05639800 | -0.25961800 |
| C | 3.21888000 | -0.15140200 | -0.46370300 |
| C | 3.01532100 | 0.15209900 | -1.95241100 |
| H | 4.34358300 | 1.88664200 | -2.02737600 |
| H | 2.79627000 | 2.15951200 | -2.86190900 |
| H | 4.26483000 | -0.40086100 | -0.23909900 |
| H | 2.58177700 | -0.96078400 | -0.09203600 |
| H | 3.68703000 | -0.42773700 | -2.59177300 |
| H | 1.98207700 | -0.06356900 | -2.24658200 |
| H | 1.44807700 | 2.19700900 | -0.83631200 |

**1-I-$PMe_3$**

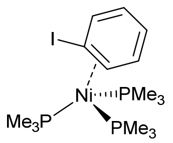


E = -1796.04683539

$G_{corr}$ = 0.375599

| | | | |
|---|---|---|---|
| Ni | -1.26811300 | -0.67801000 | 0.33288100 |
| P | -0.49691400 | 0.41599500 | -1.42677400 |
| P | -0.41998400 | -2.74211900 | 0.27898300 |
| P | -3.49275500 | -0.79442200 | 0.14759700 |

| | | | |
|---|---|---|---|
| C | -0.64408300 | 0.70753700 | 1.96746700 |
| C | -1.43510000 | -0.15462000 | 2.77493900 |
| C | 0.77285400 | 0.58287800 | 2.01858100 |
| C | -0.80473100 | -1.08237600 | 3.63381500 |
| H | -2.50027000 | 0.01709800 | 2.86637800 |
| C | 1.36575400 | -0.35677900 | 2.85141500 |
| H | 1.38897500 | 1.24783800 | 1.42518800 |
| C | 0.57874000 | -1.17944500 | 3.68269300 |
| H | -1.42471600 | -1.70667100 | 4.27268900 |
| H | 2.44965400 | -0.44156600 | 2.86805400 |
| H | 1.05553600 | -1.88671900 | 4.35609900 |
| C | -4.44285100 | -1.96379800 | 1.23501200 |
| H | -4.31838800 | -1.67142100 | 2.28393600 |
| H | -5.51466100 | -1.97781700 | 0.99779000 |
| H | -4.04021300 | -2.97649400 | 1.12037700 |
| C | -4.55024100 | 0.72065100 | 0.33101800 |
| H | -5.60908300 | 0.50714600 | 0.13461100 |
| H | -4.45141200 | 1.12019800 | 1.34613800 |
| H | -4.20507200 | 1.49143700 | -0.36561900 |
| C | -4.06364300 | -1.38104300 | -1.51866500 |
| H | -3.70743500 | -0.69010600 | -2.28984300 |
| H | -3.63621700 | -2.36768700 | -1.72659400 |
| H | -5.15755600 | -1.44600900 | -1.58054600 |
| C | -0.18396800 | -0.62762500 | -2.92948400 |
| H | 0.13470400 | -0.01997800 | -3.78578200 |
| H | 0.59583300 | -1.36531700 | -2.71329900 |
| H | -1.10093600 | -1.16298900 | -3.19888300 |
| C | -1.59011300 | 1.71185300 | -2.18656800 |
| H | -2.55887100 | 1.27203900 | -2.44554400 |
| H | -1.76238100 | 2.51509000 | -1.46391700 |
| H | -1.14299300 | 2.13825000 | -3.09378500 |
| C | 1.09278500 | 1.36990000 | -1.36062900 |
| H | 1.90379000 | 0.71876700 | -1.01796100 |
| H | 1.35532600 | 1.78287000 | -2.34264900 |
| H | 0.98995900 | 2.19729100 | -0.65083400 |
| C | 1.41910900 | -2.93097500 | 0.10034900 |
| H | 1.91456100 | -2.44982700 | 0.95030800 |
| H | 1.72830400 | -3.98326700 | 0.05299600 |
| H | 1.74728500 | -2.42204500 | -0.81304900 |
| C | -0.99013200 | -3.80969500 | -1.13128200 |
| H | -0.83253600 | -3.29833400 | -2.08530700 |
| H | -0.45659100 | -4.76855400 | -1.15139400 |
| H | -2.06214400 | -4.00908500 | -1.02617500 |
| C | -0.72381800 | -3.92968800 | 1.67408700 |
| H | -0.23573200 | -3.56738300 | 2.58242500 |
| H | -1.80025700 | -3.99008500 | 1.86934400 |
| H | -0.34350600 | -4.93215800 | 1.43972700 |
| I | -1.40257900 | 2.78205300 | 1.67982500 |

**1-I-PMe$_2$Ph**

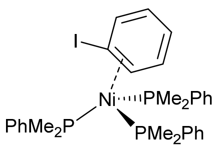


E = -2371.43853795

$G_{corr}$ = 0.523498

| | | | |
|---|---|---|---|
| Ni | -0.35032100 | 0.73709900 | -0.43214600 |
| P | 0.16086700 | 0.76725700 | -2.56401900 |
| P | 1.52361900 | 0.77784700 | 0.71676800 |
| P | -1.92677800 | 2.20872300 | -0.01240900 |
| C | 1.64705600 | 1.80645500 | -2.91943100 |
| C | 1.55014700 | 3.20056600 | -2.75606700 |
| H | 0.59812900 | 3.65163100 | -2.49669800 |
| C | 2.67124900 | 4.01802000 | -2.89440200 |
| H | 2.56899100 | 5.09189600 | -2.75947000 |
| C | 3.91999600 | 3.45908300 | -3.18594600 |
| H | 4.79647700 | 4.09438700 | -3.28376700 |
| C | 4.03131600 | 2.07634100 | -3.34794900 |
| H | 4.99629300 | 1.62985100 | -3.57590100 |
| C | 2.90440000 | 1.25734800 | -3.22021600 |
| H | 3.01860300 | 0.18520700 | -3.35079600 |
| C | -1.04503600 | 1.47237300 | -3.79352400 |
| H | -1.97498400 | 0.89634900 | -3.73689100 |
| H | -0.66101200 | 1.43668400 | -4.82028600 |
| H | -1.27236600 | 2.51052700 | -3.53613700 |
| C | 0.56377900 | -0.81857500 | -3.44410500 |
| H | -0.34874000 | -1.42365800 | -3.48017800 |
| H | 1.32109800 | -1.38613100 | -2.89429600 |
| H | 0.91381500 | -0.65232700 | -4.46955800 |
| C | 1.47031600 | 0.70449100 | 2.56692400 |
| C | 0.99771500 | 1.82851200 | 3.26934800 |
| H | 0.72377400 | 2.72690500 | 2.72300700 |
| C | 0.86984600 | 1.81104300 | 4.65697700 |
| H | 0.50797200 | 2.69504900 | 5.17660100 |
| C | 1.20289100 | 0.65908000 | 5.37914100 |
| H | 1.10159000 | 0.64230600 | 6.46120900 |
| C | 1.66855300 | -0.46575100 | 4.69709200 |
| H | 1.93096300 | -1.36636700 | 5.24705900 |
| C | 1.80340800 | -0.44295100 | 3.30471100 |
| H | 2.17199300 | -1.33165200 | 2.80099000 |
| C | 2.68606800 | -0.61186000 | 0.30687500 |
| H | 2.17501900 | -1.57631700 | 0.39735200 |
| H | 3.59218300 | -0.62362900 | 0.92488500 |
| H | 2.97661400 | -0.48381500 | -0.74153000 |
| C | 2.68389800 | 2.20902400 | 0.48222200 |
| H | 2.17087300 | 3.15214600 | 0.68333500 |
| H | 3.01597700 | 2.22275700 | -0.55862500 |
| H | 3.55831900 | 2.12430800 | 1.13764300 |
| C | -1.34121200 | 3.89718700 | -0.49873500 |
| C | -1.79039800 | 4.56104500 | -1.65241900 |
| H | -2.57692700 | 4.12948400 | -2.26316200 |
| C | -1.23035900 | 5.78235400 | -2.04362700 |

| | | | |
|---|---|---|---|
| H | -1.58889700 | 6.27200000 | -2.94581300 |
| C | -0.21855500 | 6.37054100 | -1.28251800 |
| H | 0.21793500 | 7.31789100 | -1.58779600 |
| C | 0.22404700 | 5.73076800 | -0.11926700 |
| H | 1.00577500 | 6.18142500 | 0.48729900 |
| C | -0.32937800 | 4.50921300 | 0.26267200 |
| H | 0.03515400 | 4.01999300 | 1.16074000 |
| C | -2.56023300 | 2.55145100 | 1.70302700 |
| H | -3.16139800 | 1.69462900 | 2.02100300 |
| H | -3.17768500 | 3.45677000 | 1.74241400 |
| H | -1.72389400 | 2.64794700 | 2.40033600 |
| C | -3.56797400 | 2.12295400 | -0.88088300 |
| H | -4.11114200 | 1.26490700 | -0.47568600 |
| H | -3.42880700 | 1.95085000 | -1.95226200 |
| H | -4.17015100 | 3.02699800 | -0.73014700 |
| C | -2.28197500 | -1.10316800 | 1.60960400 |
| C | -1.07563200 | -1.29893500 | 0.90662100 |
| H | -0.14760600 | -1.41542200 | 1.45002800 |
| C | -1.12880100 | -1.54786500 | -0.47938200 |
| H | -0.23219700 | -1.87209100 | -0.99545300 |
| C | -3.51297300 | -1.13588200 | 0.96595500 |
| H | -4.43465300 | -0.98387400 | 1.51838500 |
| C | -2.37684500 | -1.57399200 | -1.13676300 |
| H | -2.41387100 | -1.77764700 | -2.20350700 |
| C | -3.55122000 | -1.37210500 | -0.42165700 |
| H | -4.51245900 | -1.39831500 | -0.92794400 |
| I | -2.18505600 | -0.77562200 | 3.70614600 |

**1-I-$PMePh_2$**

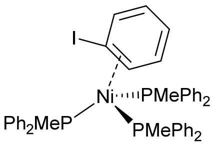


E = -2946.82558215

$G_{corr}$ = 0.672645

| | | | |
|---|---|---|---|
| Ni | 9.04923100 | 10.20978200 | 3.67887300 |
| P | 9.98827100 | 8.32627800 | 3.17714500 |
| C | 8.57157600 | 7.26872400 | 2.62356400 |
| C | 7.91661900 | 7.63090000 | 1.43129600 |
| H | 8.30874800 | 8.44661000 | 0.82992200 |
| C | 6.75413400 | 6.97580600 | 1.02412600 |
| H | 6.26337400 | 7.27272200 | 0.10054500 |
| C | 6.21527600 | 5.94909200 | 1.80751700 |
| H | 5.30478500 | 5.44353400 | 1.49676600 |
| C | 6.85773700 | 5.57888000 | 2.99101900 |
| H | 6.44874800 | 4.78166300 | 3.60692100 |
| C | 8.02734700 | 6.23020200 | 3.39475200 |
| H | 8.50799600 | 5.93470000 | 4.32132700 |
| C | 10.80532800 | 7.33522500 | 4.51081100 |
| C | 11.01768200 | 7.96515900 | 5.74395500 |
| H | 10.68944200 | 8.99062500 | 5.86975200 |
| C | 11.63762300 | 7.29054600 | 6.79794200 |
| H | 11.78943500 | 7.80051300 | 7.74496900 |
| C | 12.04896600 | 5.96701900 | 6.62976700 |
| H | 12.52642300 | 5.43473500 | 7.44873000 |
| C | 11.84241500 | 5.32436500 | 5.40366500 |
| H | 12.15841900 | 4.29262100 | 5.26963700 |
| C | 11.22964700 | 6.00523800 | 4.35007700 |
| H | 11.06626800 | 5.49097000 | 3.40627400 |
| C | 11.18765000 | 8.03686400 | 1.77884300 |
| H | 11.42885000 | 6.97645600 | 1.64773800 |
| H | 12.10932700 | 8.58955700 | 1.98542400 |
| H | 10.75477700 | 8.41505700 | 0.84782200 |
| P | 8.38601300 | 11.73082900 | 2.29075000 |
| C | 7.86668600 | 13.29304100 | 3.13344900 |
| C | 8.85105000 | 14.17331800 | 3.62054200 |
| H | 9.89683600 | 14.00190300 | 3.37799600 |
| C | 8.50598000 | 15.26615200 | 4.41506300 |
| H | 9.28251200 | 15.93513200 | 4.77788100 |
| C | 7.16666300 | 15.49935800 | 4.74796400 |
| H | 6.89738100 | 16.34677600 | 5.37287100 |
| C | 6.18019500 | 14.63452700 | 4.26977800 |
| H | 5.13617500 | 14.80456000 | 4.52147000 |
| C | 6.52569800 | 13.54214600 | 3.46781300 |
| H | 5.74048100 | 12.87777700 | 3.12172800 |
| C | 9.46484600 | 12.38235000 | 0.92430700 |
| C | 9.19719600 | 13.58067500 | 0.24282200 |
| H | 8.34947900 | 14.19381700 | 0.53824000 |
| C | 10.01683400 | 13.99710800 | -0.80808500 |
| H | 9.80092300 | 14.92844600 | -1.32598000 |
| C | 11.11364400 | 13.21939000 | -1.19538600 |
| H | 11.75166700 | 13.54671000 | -2.01231600 |
| C | 11.38756100 | 12.02460400 | -0.52573300 |
| H | 12.24111200 | 11.41711800 | -0.81699900 |
| C | 10.56864200 | 11.61367200 | 0.52967900 |
| H | 10.78860400 | 10.69979700 | 1.07263500 |
| C | 6.88162700 | 11.30376900 | 1.28568700 |
| H | 6.50846500 | 12.16966600 | 0.72778200 |
| H | 6.09078700 | 10.90929400 | 1.92784800 |
| H | 7.16398100 | 10.51886900 | 0.57751600 |
| P | 7.89934200 | 10.37870700 | 5.52668400 |
| C | 8.00635100 | 9.06598800 | 6.83727500 |
| C | 8.61838700 | 9.28435900 | 8.08072400 |
| H | 8.96158100 | 10.27465700 | 8.35895600 |
| C | 8.81913700 | 8.22740600 | 8.97501500 |
| H | 9.30678100 | 8.41837000 | 9.92818700 |
| C | 8.40078500 | 6.93648400 | 8.64858500 |
| H | 8.56173300 | 6.11527500 | 9.34207600 |
| C | 7.77034900 | 6.71062500 | 7.42030600 |
| H | 7.43357100 | 5.71138000 | 7.15459700 |
| C | 7.58114600 | 7.76236300 | 6.52558000 |
| H | 7.10526300 | 7.56444800 | 5.57071000 |
| C | 6.10078500 | 10.28403300 | 5.09139800 |
| C | 5.72931900 | 9.40176000 | 4.06124400 |
| H | 6.49803300 | 8.82777500 | 3.55109000 |
| C | 4.39566600 | 9.28018300 | 3.66751800 |
| H | 4.13264700 | 8.58766800 | 2.87199600 |
| C | 3.41125900 | 10.06029900 | 4.28164800 |

| | | | |
|---|---|---|---|
| H | 2.37340000 | 9.97816100 | 3.96912900 |
| C | 3.77027100 | 10.95100800 | 5.29677300 |
| H | 3.01127700 | 11.56416400 | 5.77657600 |
| C | 5.10339800 | 11.05725200 | 5.70460500 |
| H | 5.35575400 | 11.75277400 | 6.49872700 |
| C | 8.01354000 | 11.92949200 | 6.53352100 |
| H | 7.39656800 | 11.90761000 | 7.43812300 |
| H | 7.71196700 | 12.77387000 | 5.90852800 |
| H | 9.05661500 | 12.07904200 | 6.82747500 |
| C | 11.65939000 | 11.83205200 | 5.04466600 |
| H | 10.82778400 | 12.43810800 | 5.38471300 |
| C | 12.46186900 | 11.12509300 | 5.94343400 |
| C | 13.52787000 | 10.33646900 | 5.50301700 |
| H | 14.13469500 | 9.77991400 | 6.20901900 |
| C | 11.95078100 | 11.75449600 | 3.67927300 |
| H | 11.34252300 | 12.30806200 | 2.97559900 |
| C | 13.01813800 | 10.97920800 | 3.22132500 |
| H | 13.23218200 | 10.93013900 | 2.15764700 |
| C | 13.80026300 | 10.26774600 | 4.13331800 |
| H | 14.62856800 | 9.65529300 | 3.78719700 |
| I | 12.04235400 | 11.22799100 | 8.02346000 |

**1-I-$PPh_3$**

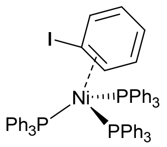


E = -3522.20848963

$G_{corr}$ = 0.817953

| | | | |
|---|---|---|---|
| Ni | 9.88482800 | 11.29468700 | 3.81988700 |
| P | 10.12839700 | 10.69239700 | 1.72997000 |
| C | 9.02492700 | 9.39146600 | 1.01094400 |
| C | 7.77605900 | 9.18483900 | 1.61472400 |
| H | 7.52209600 | 9.73207800 | 2.51562300 |
| C | 6.85875200 | 8.28726600 | 1.06263200 |
| H | 5.88856100 | 8.15681900 | 1.53385600 |
| C | 7.18920600 | 7.56393900 | -0.08561300 |
| H | 6.47831500 | 6.86113900 | -0.51252600 |
| C | 8.44097400 | 7.74512800 | -0.68201800 |
| H | 8.70678000 | 7.18217500 | -1.57327100 |
| C | 9.35083800 | 8.65634200 | -0.14164900 |
| H | 10.31395100 | 8.79956100 | -0.62294000 |
| C | 11.78077900 | 10.14800500 | 1.08533000 |
| C | 12.34719700 | 8.99166700 | 1.64852400 |
| H | 11.78720000 | 8.42350400 | 2.38385600 |
| C | 13.63045400 | 8.57763100 | 1.29467100 |
| H | 14.05265300 | 7.68704400 | 1.75247700 |
| C | 14.37617600 | 9.31917400 | 0.37174900 |
| H | 15.38198600 | 9.00627900 | 0.10363300 |
| C | 13.82034500 | 10.46453100 | -0.20215000 |
| H | 14.38963900 | 11.04330700 | -0.92549700 |
| C | 12.52957900 | 10.87483700 | 0.14791500 |
| H | 12.11437400 | 11.77022100 | -0.30390700 |
| P | 8.08400800 | 12.43759100 | 4.25442300 |
| C | 7.38743000 | 12.39178900 | 5.97250100 |
| C | 6.57900800 | 13.40649200 | 6.51036400 |
| H | 6.32535400 | 14.27660100 | 5.91178700 |
| C | 6.10507600 | 13.31369300 | 7.81987600 |
| H | 5.48673300 | 14.10947300 | 8.22801300 |
| C | 6.42576700 | 12.20105200 | 8.60529100 |
| H | 6.06142700 | 12.13212500 | 9.62721400 |
| C | 7.22367200 | 11.18554200 | 8.07569000 |
| H | 7.49369200 | 10.32690100 | 8.68229100 |
| C | 7.70474900 | 11.28323100 | 6.76796200 |
| H | 8.35228400 | 10.50947500 | 6.36440600 |
| C | 8.36729900 | 14.25016500 | 3.98895200 |
| C | 7.37441900 | 15.15901600 | 3.59041800 |
| H | 6.36101800 | 14.81361600 | 3.40974600 |
| C | 7.68075200 | 16.51030200 | 3.40756900 |
| H | 6.90135000 | 17.19953900 | 3.09244800 |
| C | 8.98039800 | 16.97557800 | 3.62684400 |
| H | 9.21583600 | 18.02653900 | 3.47991900 |
| C | 9.97550700 | 16.08083000 | 4.03049900 |
| H | 10.99356600 | 16.42473800 | 4.19531400 |
| C | 9.66919000 | 14.73094100 | 4.20505400 |
| H | 10.45178700 | 14.03438400 | 4.48645800 |
| P | 11.60352200 | 11.21891600 | 5.13972300 |
| C | 11.48232800 | 11.51471700 | 6.96856600 |
| C | 10.61033700 | 12.52396800 | 7.40744300 |
| H | 10.04901000 | 13.10032600 | 6.67920100 |
| C | 10.44282400 | 12.78664500 | 8.76774900 |
| H | 9.75784600 | 13.56926400 | 9.08306300 |
| C | 11.13165300 | 12.02643400 | 9.71657700 |
| H | 10.99205300 | 12.21818300 | 10.77737400 |
| C | 11.99904800 | 11.01565700 | 9.29314200 |
| H | 12.54147300 | 10.42098400 | 10.02427900 |
| C | 12.17850800 | 10.76511300 | 7.92996900 |
| H | 12.85790000 | 9.97714300 | 7.62106800 |
| C | 12.78906800 | 12.53442500 | 4.59222500 |
| C | 13.03329600 | 13.70822900 | 5.32144700 |
| H | 12.61040100 | 13.83524700 | 6.31288700 |
| C | 13.81755900 | 14.73219700 | 4.77946000 |
| H | 13.99285700 | 15.63521900 | 5.35910700 |
| C | 14.37745400 | 14.59607300 | 3.50741200 |
| H | 14.98535900 | 15.39357100 | 3.08819900 |
| C | 14.15429100 | 13.42233200 | 2.77985100 |
| H | 14.58864500 | 13.29692500 | 1.79117000 |
| C | 13.36425200 | 12.40554100 | 3.31438700 |
| H | 13.19621800 | 11.50395900 | 2.73695700 |
| C | 12.61030900 | 9.67218800 | 5.08226700 |
| C | 11.89329800 | 8.46902500 | 5.00284600 |
| C | 14.01129000 | 9.62707100 | 5.11580900 |
| C | 12.55634000 | 7.24205700 | 4.97081500 |
| H | 10.80938600 | 8.51046400 | 4.94754300 |
| C | 14.67889600 | 8.40021500 | 5.06364900 |
| H | 14.58414100 | 10.54769300 | 5.17794100 |
| C | 13.95455200 | 7.20633400 | 4.99561600 |
| H | 11.98625300 | 6.31835600 | 4.91163400 |

| | | | |
|---|---|---|---|
| H | 15.76567300 | 8.37734400 | 5.07954900 |
| H | 14.47657900 | 6.25371500 | 4.95767500 |
| C | 6.56710200 | 12.08443500 | 3.25162800 |
| C | 6.50102900 | 12.49821100 | 1.90874400 |
| C | 5.56317400 | 11.23359400 | 3.74250400 |
| C | 5.45852700 | 12.07637400 | 1.08395400 |
| H | 7.27065800 | 13.14609600 | 1.50228000 |
| C | 4.52333000 | 10.80609700 | 2.91195600 |
| H | 5.59801400 | 10.88766500 | 4.77132700 |
| C | 4.46661500 | 11.22458700 | 1.58035000 |
| H | 5.42896700 | 12.40662200 | 0.04868600 |
| H | 3.75729900 | 10.14495800 | 3.30984600 |
| H | 3.65922800 | 10.88936500 | 0.93451800 |
| C | 9.76264200 | 12.16537100 | 0.66706100 |
| C | 10.22113000 | 13.40974100 | 1.13234900 |
| C | 8.99627600 | 12.12414900 | -0.50640600 |
| C | 9.90899900 | 14.58678500 | 0.45299200 |
| H | 10.80291800 | 13.45202900 | 2.04785500 |
| C | 8.67977400 | 13.30403800 | -1.18665300 |
| H | 8.62514900 | 11.17648100 | -0.88337100 |
| C | 9.12914600 | 14.53723000 | -0.70720100 |
| H | 10.25512300 | 15.54008800 | 0.84320900 |
| H | 8.07413900 | 13.25772900 | -2.08843900 |
| H | 8.87145400 | 15.45382700 | -1.23160800 |
| C | 7.80575800 | 7.73887500 | 6.74728200 |
| C | 7.99227800 | 7.45094900 | 5.39253900 |
| C | 6.52564400 | 7.94048500 | 7.27119200 |
| C | 6.87693100 | 7.36890400 | 4.55481400 |
| H | 8.98485000 | 7.28841400 | 4.98818800 |
| C | 5.42067000 | 7.86371500 | 6.41805900 |
| H | 6.38643100 | 8.16415900 | 8.32294900 |
| C | 5.59266000 | 7.58129400 | 5.06088200 |
| H | 7.02266300 | 7.14542200 | 3.50342300 |
| H | 4.42543700 | 8.02702700 | 6.82273500 |
| H | 4.73114000 | 7.52333900 | 4.40165800 |
| I | 9.50022600 | 7.93387800 | 8.01184700 |

### 1-Br-PMe$_3$

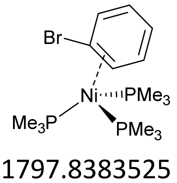


E = -1797.8383525

$G_{corr}$ = 0.37561

| | | | |
|---|---|---|---|
| Ni | -1.26659300 | -0.71246100 | 0.36778700 |
| P | -0.53301100 | 0.31658100 | -1.46597500 |
| P | -0.52487800 | -2.85111600 | 0.14705800 |
| P | -3.52146500 | -0.88934500 | -0.01169600 |
| C | -0.64872900 | 0.56034500 | 1.72586800 |
| C | -1.45073200 | -0.38760800 | 2.45563300 |
| C | 0.77447500 | 0.55081700 | 1.92940800 |
| Br | -1.40745300 | 2.49600600 | 1.60739600 |
| C | -0.80277500 | -1.27845400 | 3.37164100 |
| H | -2.50049100 | -0.17436200 | 2.62947500 |
| C | 1.35950800 | -0.37191600 | 2.76818400 |
| H | 1.38470700 | 1.28553200 | 1.41595500 |
| C | 0.56608200 | -1.29132600 | 3.51144100 |
| H | -1.42387900 | -1.93454400 | 3.97825500 |
| H | 2.44208600 | -0.38455900 | 2.87465800 |
| H | 1.04429600 | -1.98115300 | 4.20243200 |
| C | -4.42301500 | -2.15916500 | 0.99657000 |
| H | -4.22663700 | -1.99063900 | 2.06118700 |
| H | -5.50679500 | -2.13368300 | 0.82561200 |
| H | -4.05095100 | -3.15679900 | 0.73842300 |
| C | -4.56169000 | 0.60556300 | 0.32601400 |
| H | -5.62913900 | 0.41252000 | 0.15888000 |
| H | -4.41334900 | 0.92574100 | 1.36247900 |
| H | -4.23859400 | 1.42587100 | -0.32261800 |
| C | -4.12455200 | -1.35059700 | -1.70441200 |
| H | -3.79452800 | -0.60541700 | -2.43524400 |
| H | -3.69795200 | -2.31750800 | -1.99240800 |
| H | -5.21898100 | -1.42060100 | -1.74416300 |
| C | -0.32714800 | -0.70421900 | -2.99747600 |
| H | -0.04585900 | -0.08420900 | -3.85743900 |
| H | 0.45190100 | -1.45732600 | -2.83921600 |
| H | -1.26579900 | -1.21953700 | -3.22528000 |
| C | -1.60700800 | 1.67669400 | -2.12107700 |
| H | -2.60197300 | 1.28621400 | -2.35604900 |
| H | -1.71422100 | 2.44992800 | -1.35524400 |
| H | -1.18090100 | 2.12403000 | -3.02786000 |
| C | 1.09928200 | 1.18794600 | -1.42543600 |
| H | 1.88263300 | 0.49517900 | -1.10080700 |
| H | 1.36186700 | 1.59213300 | -2.41065500 |
| H | 1.04995100 | 2.01361500 | -0.70907800 |
| C | 1.30806900 | -3.08399000 | -0.00967000 |
| H | 1.79864500 | -2.64777800 | 0.86735200 |
| H | 1.58866400 | -4.14187900 | -0.09145900 |
| H | 1.66677800 | -2.55078200 | -0.89752400 |
| C | -1.11515300 | -3.85082500 | -1.30098900 |
| H | -0.87693900 | -3.34735100 | -2.24149000 |
| H | -0.65877400 | -4.84867900 | -1.31055800 |
| H | -2.20277800 | -3.96775100 | -1.24139900 |
| C | -0.89750200 | -4.06455000 | 1.49874600 |
| H | -0.39704300 | -3.75492500 | 2.41933300 |
| H | -1.97662700 | -4.08094400 | 1.68690200 |
| H | -0.56490000 | -5.07514300 | 1.23076300 |

### 1-Cl-PMe$_3$

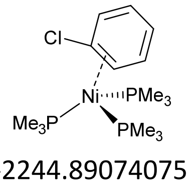


E = -2244.89074075

$G_{corr}$ = 0.378569

| | | | |
|---|---|---|---|
| Ni | -1.28640400 | -0.69503500 | 0.35771500 |
| P | -0.55764000 | 0.32899600 | -1.47456200 |

| | | | |
|---|---|---|---|
| P | -0.50777400 | -2.82674800 | 0.17771900 |
| P | -3.51805700 | -0.89376200 | -0.00703100 |
| C | -0.59406700 | 0.59627900 | 1.71104100 |
| C | -1.46808300 | -0.29594400 | 2.42879500 |
| C | 0.82382600 | 0.49428600 | 1.92764600 |
| C | -0.88558700 | -1.22766600 | 3.34910700 |
| H | -2.50052900 | -0.00528300 | 2.59762200 |
| C | 1.33934600 | -0.46079600 | 2.77370600 |
| H | 1.48020600 | 1.19298300 | 1.41974500 |
| C | 0.47753300 | -1.32584600 | 3.50738700 |
| H | -1.55329400 | -1.84916800 | 3.94226400 |
| H | 2.41711000 | -0.54250900 | 2.89470400 |
| H | 0.90129100 | -2.04571900 | 4.20318100 |
| C | -4.39426100 | -2.18248400 | 0.99880900 |
| H | -4.19054800 | -2.01476600 | 2.06215300 |
| H | -5.47960400 | -2.17420700 | 0.83612900 |
| H | -4.00689900 | -3.17230900 | 0.73347900 |
| C | -4.58973400 | 0.57941500 | 0.33857500 |
| H | -5.65117600 | 0.37241700 | 0.15119700 |
| H | -4.46855900 | 0.88300100 | 1.38431300 |
| H | -4.27264400 | 1.41668200 | -0.29216300 |
| C | -4.11504300 | -1.35409600 | -1.70184300 |
| H | -3.80613000 | -0.59276300 | -2.42532900 |
| H | -3.66347600 | -2.30625500 | -2.00062900 |
| H | -5.20730700 | -1.45302000 | -1.73958200 |
| C | -0.38227600 | -0.68312600 | -3.01665300 |
| H | -0.11037900 | -0.05955400 | -3.87717700 |
| H | 0.39509300 | -1.44142500 | -2.87567300 |
| H | -1.32670100 | -1.19230800 | -3.23415900 |
| C | -1.61742900 | 1.71007600 | -2.11218400 |
| H | -2.62273900 | 1.33498700 | -2.32888800 |
| H | -1.69838200 | 2.48422700 | -1.34377700 |
| H | -1.20137300 | 2.15318800 | -3.02585200 |
| C | 1.09127700 | 1.17148800 | -1.44863400 |
| H | 1.86611300 | 0.45870500 | -1.14705700 |
| H | 1.34733300 | 1.58498900 | -2.43186900 |
| H | 1.06908700 | 1.98630300 | -0.71878000 |
| C | 1.32816300 | -3.04576800 | 0.01607000 |
| H | 1.81876000 | -2.61397800 | 0.89500400 |
| H | 1.61836300 | -4.10030900 | -0.07723300 |
| H | 1.67976500 | -2.50045700 | -0.86744700 |
| C | -1.08328200 | -3.83137500 | -1.27504100 |
| H | -0.84809400 | -3.32278300 | -2.21350800 |
| H | -0.61695100 | -4.82479800 | -1.28730600 |
| H | -2.16998900 | -3.95903800 | -1.21972400 |
| C | -0.86466500 | -4.06014700 | 1.51877600 |
| H | -0.37954400 | -3.74882800 | 2.44675600 |
| H | -1.94477700 | -4.10023600 | 1.69824500 |
| H | -0.50969300 | -5.06201500 | 1.24598100 |
| Cl | -1.18004200 | 2.35007400 | 1.48821100 |

**TS-1-2-I-$PMe_3$**

E = -1796.04680227

$G_{corr}$ = 0.377806

| | | | |
|---|---|---|---|
| Ni | -1.26352300 | -0.67705500 | 0.33125100 |
| P | -0.49319800 | 0.41808800 | -1.42936700 |
| P | -0.41738100 | -2.74270800 | 0.27411300 |
| P | -3.49025200 | -0.79489900 | 0.14889000 |
| C | -0.65193400 | 0.70142700 | 1.96673200 |
| C | -1.44280400 | -0.15226100 | 2.78351300 |
| C | 0.76455700 | 0.57089800 | 2.01407500 |
| C | -0.81404600 | -1.07608100 | 3.64661800 |
| H | -2.50809500 | 0.02041900 | 2.87226800 |
| C | 1.35688900 | -0.36446700 | 2.85298600 |
| H | 1.38125500 | 1.22873700 | 1.41348500 |
| C | 0.56977200 | -1.17748700 | 3.69272200 |
| H | -1.43435600 | -1.69412000 | 4.29126800 |
| H | 2.44054500 | -0.45291000 | 2.86681500 |
| H | 1.04588300 | -1.88149200 | 4.37002200 |
| C | -4.43865900 | -1.96586900 | 1.23595600 |
| H | -4.31304200 | -1.67468600 | 2.28504300 |
| H | -5.51070500 | -1.97991700 | 0.99986300 |
| H | -4.03589600 | -2.97832000 | 1.11966400 |
| C | -4.54746000 | 0.71984700 | 0.33490200 |
| H | -5.60646200 | 0.50636100 | 0.13942400 |
| H | -4.44758600 | 1.11844200 | 1.35027900 |
| H | -4.20313300 | 1.49133800 | -0.36135800 |
| C | -4.06207600 | -1.38003700 | -1.51751400 |
| H | -3.70690700 | -0.68802900 | -2.28820500 |
| H | -3.63435300 | -2.36624300 | -1.72690300 |
| H | -5.15599500 | -1.44553600 | -1.57852200 |
| C | -0.18287300 | -0.62635100 | -2.93197200 |
| H | 0.13413800 | -0.01920600 | -3.78923600 |
| H | 0.59726400 | -1.36402300 | -2.71690300 |
| H | -1.10040600 | -1.16182500 | -3.19925200 |
| C | -1.58838100 | 1.71337300 | -2.18706200 |
| H | -2.55781100 | 1.27341800 | -2.44323800 |
| H | -1.75879000 | 2.51696500 | -1.46436800 |
| H | -1.14356200 | 2.13929900 | -3.09562600 |
| C | 1.09624500 | 1.37259300 | -1.36594500 |
| H | 1.90829200 | 0.72148800 | -1.02563200 |
| H | 1.35648000 | 1.78629800 | -2.34827400 |
| H | 0.99469600 | 2.19959900 | -0.65545400 |
| C | 1.42129200 | -2.93150200 | 0.09305000 |
| H | 1.91791200 | -2.44999900 | 0.94211600 |
| H | 1.73015900 | -3.98388700 | 0.04594600 |
| H | 1.74838300 | -2.42319200 | -0.82104000 |
| C | -0.99030400 | -3.81227700 | -1.13325200 |
| H | -0.83202400 | -3.30318100 | -2.08842200 |
| H | -0.45848700 | -4.77210500 | -1.15143100 |
| H | -2.06261600 | -4.00945300 | -1.02725500 |
| C | -0.71949500 | -3.92544100 | 1.67324900 |
| H | -0.23214600 | -3.55839400 | 2.58014700 |
| H | -1.79584300 | -3.98654700 | 1.86866400 |

| | | | |
|---|---|---|---|
| H | -0.33746200 | -4.92819900 | 1.44302300 |
| I | -1.40252700 | 2.78389900 | 1.68028700 |

**TS-1-2-I-PMe$_2$Ph**

E = -2371.42764175

G$_{corr}$ = 0.528074

| | | | |
|---|---|---|---|
| Ni | -1.74979600 | 3.37904100 | 0.08652900 |
| P | -1.25700300 | 3.37594500 | -2.08759300 |
| P | 0.19711900 | 3.46751600 | 1.17526800 |
| P | -3.18227900 | 5.04013700 | 0.40522900 |
| C | 0.22338400 | 4.42093300 | -2.47338700 |
| C | 0.12527000 | 5.82191800 | -2.40823400 |
| H | -0.82953400 | 6.29058400 | -2.20004500 |
| C | 1.24784300 | 6.63044500 | -2.58942200 |
| H | 1.14155100 | 7.71072300 | -2.53162200 |
| C | 2.49978000 | 6.05661500 | -2.83037300 |
| H | 3.37608500 | 6.68546600 | -2.96456000 |
| C | 2.61346800 | 4.66594500 | -2.89686100 |
| H | 3.58044300 | 4.20562000 | -3.08533700 |
| C | 1.48655800 | 3.85685500 | -2.72331500 |
| H | 1.60475900 | 2.77882900 | -2.77767200 |
| C | -2.50073400 | 4.10732400 | -3.26200000 |
| H | -3.41017400 | 3.49793500 | -3.21666000 |
| H | -2.13243100 | 4.12789600 | -4.29483200 |
| H | -2.75563800 | 5.12511400 | -2.95682300 |
| C | -0.85705500 | 1.84842200 | -3.07423800 |
| H | -1.77636400 | 1.27079000 | -3.19962100 |
| H | -0.13015900 | 1.22072200 | -2.55110100 |
| H | -0.46549500 | 2.10619300 | -4.06485600 |
| C | 0.24234300 | 3.44704000 | 3.02459400 |
| C | -0.25474700 | 4.56762800 | 3.71409200 |
| H | -0.58457500 | 5.43953200 | 3.15489500 |
| C | -0.35315500 | 4.57278000 | 5.10413200 |
| H | -0.73991700 | 5.45112500 | 5.61537700 |
| C | 0.03834500 | 3.44773700 | 5.83971900 |
| H | -0.04235400 | 3.44663000 | 6.92364800 |
| C | 0.54217000 | 2.33120900 | 5.16939600 |
| H | 0.85635400 | 1.45479100 | 5.73133800 |
| C | 0.64694100 | 2.33235800 | 3.77487500 |
| H | 1.03395000 | 1.44757700 | 3.27831600 |
| C | 1.40661600 | 2.12423200 | 0.74234000 |
| H | 0.97113900 | 1.13194300 | 0.89424700 |
| H | 2.34332000 | 2.19499100 | 1.30800400 |
| H | 1.63028600 | 2.23060700 | -0.32445000 |
| C | 1.31282600 | 4.92262400 | 0.86607800 |
| H | 0.80123700 | 5.85834500 | 1.09729000 |
| H | 1.58856400 | 4.94103400 | -0.19000300 |
| H | 2.22334900 | 4.85146400 | 1.47217100 |
| C | -2.56082600 | 6.69432400 | -0.15126100 |
| C | -3.03570300 | 7.34926300 | -1.29970700 |
| H | -3.84846000 | 6.92494800 | -1.87980500 |
| C | -2.46872700 | 8.55522400 | -1.72606700 |
| H | -2.84810000 | 9.03793300 | -2.62337800 |
| C | -1.42442100 | 9.13709100 | -1.00537300 |
| H | -0.98241500 | 10.07254500 | -1.33809000 |
| C | -0.95784600 | 8.50839300 | 0.15409900 |
| H | -0.15194300 | 8.95575500 | 0.73055300 |
| C | -1.52017900 | 7.30274600 | 0.57214200 |
| H | -1.14300400 | 6.82738600 | 1.47262900 |
| C | -3.79051200 | 5.50597200 | 2.10333900 |
| H | -4.43818500 | 4.70724900 | 2.47627800 |
| H | -4.35562600 | 6.44505800 | 2.08417500 |
| H | -2.94894400 | 5.60133400 | 2.79471200 |
| C | -4.83229400 | 4.97139400 | -0.44672900 |
| H | -5.40135700 | 4.14467800 | -0.01413500 |
| H | -4.71330800 | 4.76566300 | -1.51383800 |
| H | -5.40447300 | 5.89713800 | -0.31434800 |
| C | -3.21971500 | 1.72218200 | 1.21338600 |
| C | -2.14833900 | 1.07133600 | 0.55982800 |
| H | -1.22477100 | 0.87105200 | 1.08685500 |
| C | -2.40958100 | 0.39599000 | -0.65537000 |
| H | -1.59866600 | -0.15288700 | -1.12404000 |
| C | -4.51196600 | 1.71698500 | 0.63370700 |
| H | -5.33892600 | 2.18327600 | 1.15866500 |
| C | -3.67143900 | 0.41121200 | -1.22996700 |
| H | -3.85287700 | -0.11360100 | -2.16409100 |
| C | -4.72380900 | 1.09001600 | -0.58596400 |
| H | -5.71782600 | 1.10152200 | -1.02581500 |
| I | -3.12594700 | 2.00355000 | 3.34339400 |

**TS-1-2-I-PMePh$_2$**

E = -2946.81030933

G$_{corr}$ = 0.678608

| | | | |
|---|---|---|---|
| Ni | -2.39362100 | 0.71517600 | -0.00066400 |
| P | -1.97553200 | -1.34589900 | -0.75826200 |
| C | -3.46716100 | -2.39100200 | -1.13934200 |
| C | -4.19916500 | -2.13501700 | -2.31230600 |
| H | -3.84503600 | -1.39603300 | -3.02235300 |
| C | -5.39338900 | -2.80742800 | -2.57788300 |
| H | -5.94095600 | -2.58930000 | -3.49155000 |
| C | -5.88432000 | -3.75371700 | -1.67353500 |
| H | -6.81693100 | -4.27385500 | -1.87550400 |
| C | -5.16004400 | -4.02879000 | -0.51138400 |
| H | -5.52448300 | -4.76930200 | 0.19637300 |
| C | -3.96231900 | -3.35842100 | -0.24950500 |
| H | -3.41730600 | -3.59028800 | 0.65937100 |
| C | -1.01087200 | -2.48029200 | 0.34458700 |
| C | -1.30303000 | -2.44361900 | 1.71661400 |
| H | -2.05411100 | -1.75321800 | 2.07901600 |
| C | -0.63199200 | -3.26890800 | 2.61987900 |
| H | -0.88676900 | -3.22520400 | 3.67557900 |
| C | 0.37083600 | -4.12941500 | 2.16666000 |
| H | 0.90613800 | -4.76518000 | 2.86725400 |
| C | 0.68640600 | -4.16278300 | 0.80512500 |
| H | 1.46993700 | -4.82456000 | 0.44414500 |
| C | -0.00365800 | -3.35224300 | -0.10054800 |

| | | | |
|---|---|---|---|
| H | 0.25729600 | -3.40661400 | -1.15208900 |
| C | -1.06777900 | -1.52894500 | -2.36988200 |
| H | -0.99566900 | -2.56911400 | -2.70341200 |
| H | -0.06344800 | -1.10781500 | -2.26212400 |
| H | -1.59578300 | -0.95879300 | -3.13773700 |
| P | -3.25839000 | 2.05537400 | -1.59018800 |
| C | -4.07333600 | 3.56101100 | -0.88658100 |
| C | -3.28606600 | 4.60666800 | -0.36820900 |
| H | -2.20677400 | 4.57303900 | -0.47262100 |
| C | -3.87175000 | 5.69803100 | 0.27183700 |
| H | -3.24296200 | 6.49645200 | 0.65807800 |
| C | -5.26185400 | 5.76331400 | 0.42031300 |
| H | -5.71955400 | 6.60920900 | 0.92644800 |
| C | -6.05421600 | 4.73424500 | -0.08969500 |
| H | -7.13495100 | 4.76933800 | 0.02124400 |
| C | -5.46665700 | 3.64548700 | -0.74168100 |
| H | -6.10799400 | 2.85529600 | -1.11677400 |
| C | -2.22450500 | 2.78935000 | -2.95081300 |
| C | -2.50595200 | 4.02674200 | -3.55252600 |
| H | -3.32329300 | 4.63919100 | -3.18338400 |
| C | -1.74299900 | 4.48489100 | -4.62971400 |
| H | -1.97263200 | 5.44717800 | -5.08075700 |
| C | -0.69488100 | 3.70813100 | -5.13187200 |
| H | -0.10292400 | 4.06545600 | -5.97057400 |
| C | -0.41192900 | 2.47167500 | -4.54664400 |
| H | 0.40574800 | 1.86188300 | -4.92312900 |
| C | -1.16693000 | 2.02296100 | -3.46076100 |
| H | -0.91751800 | 1.08307000 | -2.98402500 |
| C | -4.59287400 | 1.39595700 | -2.70795000 |
| H | -5.06532100 | 2.20613000 | -3.27369800 |
| H | -5.35181500 | 0.84512600 | -2.15150300 |
| H | -4.11717400 | 0.71386800 | -3.41761200 |
| P | -3.91743700 | 0.90510500 | 1.67732800 |
| C | -4.07925000 | -0.32754700 | 3.06774200 |
| C | -3.71234300 | -0.04448600 | 4.39281300 |
| H | -3.35767400 | 0.94346900 | 4.66626000 |
| C | -3.78440000 | -1.02906000 | 5.38327000 |
| H | -3.49275800 | -0.78508500 | 6.40207800 |
| C | -4.22735300 | -2.31578600 | 5.07032100 |
| H | -4.27898500 | -3.08104700 | 5.84046500 |
| C | -4.61005300 | -2.60718300 | 3.75735300 |
| H | -4.96283900 | -3.60217500 | 3.49679100 |
| C | -4.53696700 | -1.62344900 | 2.77147700 |
| H | -4.83438700 | -1.87259800 | 1.75843700 |
| C | -5.68271400 | 0.85021300 | 1.10247000 |
| C | -6.02087000 | -0.07802200 | 0.10667100 |
| H | -5.24592300 | -0.70235700 | -0.32210300 |
| C | -7.33499600 | -0.19830100 | -0.35132000 |
| H | -7.56760000 | -0.92885300 | -1.12174900 |
| C | -8.33483700 | 0.62584400 | 0.17098200 |
| H | -9.35699900 | 0.54460200 | -0.18974700 |
| C | -8.01101000 | 1.55575000 | 1.16333800 |
| H | -8.78163200 | 2.20192200 | 1.57661300 |
| C | -6.69890600 | 1.66212400 | 1.63079700 |
| H | -6.47812000 | 2.39393800 | 2.40030200 |
| C | -3.86736800 | 2.50621200 | 2.60884900 |
| H | -4.57230400 | 2.54959900 | 3.44471900 |
| H | -4.08538800 | 3.31658000 | 1.90957800 |
| H | -2.85705100 | 2.65540600 | 2.99801300 |
| C | -0.69183200 | 2.27190600 | 0.58059200 |
| H | -1.33550500 | 2.92606400 | 1.15707600 |
| C | -0.27938700 | 1.01983400 | 1.10003400 |
| C | 0.75866100 | 0.29311100 | 0.45467200 |
| H | 1.07724000 | -0.65953100 | 0.86219600 |
| C | -0.00205700 | 2.79576800 | -0.54047000 |
| H | -0.27624500 | 3.77806400 | -0.91214900 |
| C | 1.01689300 | 2.08860400 | -1.15405900 |
| H | 1.53187500 | 2.51159800 | -2.01121700 |
| C | 1.37587700 | 0.81386700 | -0.66962100 |
| H | 2.16482100 | 0.24586200 | -1.15657500 |
| I | -0.35864800 | 0.72468200 | 3.24761900 |

**TS-1-2-I-PPh$_3$**

E = -3522.177200

$G_{corr}$ = 0.828053

| | | | |
|---|---|---|---|
| Ni | -0.14490500 | 0.11140500 | 0.08611000 |
| P | 0.02944800 | -0.36614700 | -2.17758100 |
| C | -1.39465200 | -1.03523500 | -3.17176500 |
| C | -2.52690600 | -0.22688700 | -3.35940400 |
| H | -2.53202200 | 0.78160300 | -2.97240800 |
| C | -3.64607700 | -0.70173700 | -4.04205400 |
| H | -4.50529100 | -0.04966200 | -4.17537000 |
| C | -3.66086300 | -2.00620400 | -4.54578800 |
| H | -4.53455200 | -2.38221400 | -5.07192600 |
| C | -2.53951100 | -2.81899100 | -4.37062000 |
| H | -2.53469900 | -3.83403500 | -4.76021900 |
| C | -1.41447900 | -2.33723600 | -3.69434400 |
| H | -0.55931600 | -2.99169000 | -3.56869200 |
| C | 1.31662500 | -1.61430600 | -2.66574200 |
| C | 1.56642600 | -2.67107400 | -1.78039100 |
| H | 1.05511200 | -2.68966800 | -0.82479200 |
| C | 2.48072100 | -3.67576700 | -2.10772900 |
| H | 2.66322900 | -4.48401600 | -1.40516300 |
| C | 3.17699700 | -3.62064900 | -3.31647100 |
| H | 3.89981300 | -4.39273600 | -3.56721400 |
| C | 2.94502000 | -2.56219700 | -4.20148600 |
| H | 3.48670300 | -2.51027400 | -5.14265900 |
| C | 2.01367400 | -1.57177400 | -3.88465500 |
| H | 1.83730900 | -0.76171400 | -4.58530300 |
| P | -1.20480000 | 2.22888300 | 0.23277800 |
| C | -2.16678800 | 2.71576200 | 1.77750900 |
| C | -1.68233500 | 3.72247700 | 2.63591400 |
| H | -0.78013900 | 4.26687800 | 2.38532900 |
| C | -2.33533200 | 4.04202000 | 3.82840000 |
| H | -1.92912400 | 4.82558000 | 4.46317300 |
| C | -3.50229500 | 3.37129200 | 4.19879400 |
| H | -4.01687100 | 3.62539200 | 5.12161600 |
| C | -3.99480700 | 2.36525400 | 3.36737900 |

| | | | |
|---|---|---|---|
| H | -4.89409300 | 1.81887100 | 3.63959100 |
| C | -3.32743100 | 2.03418400 | 2.18696600 |
| H | -3.72657300 | 1.21328400 | 1.60786600 |
| C | -0.14550600 | 3.75277600 | 0.08685700 |
| C | -0.68894600 | 5.02422800 | -0.17521800 |
| H | -1.75778000 | 5.13611900 | -0.32850000 |
| C | 0.13159800 | 6.14958400 | -0.24035100 |
| H | -0.30591900 | 7.12229900 | -0.45003100 |
| C | 1.51077800 | 6.02973500 | -0.03336300 |
| H | 2.14898400 | 6.90788700 | -0.08764700 |
| C | 2.05849900 | 4.77755500 | 0.24592100 |
| H | 3.12668500 | 4.66324600 | 0.40810300 |
| C | 1.22987100 | 3.65408600 | 0.30690100 |
| H | 1.66152800 | 2.68773600 | 0.51770500 |
| P | 1.91455700 | 0.00775700 | 1.11200600 |
| C | 2.10253300 | 0.87485000 | 2.74900100 |
| C | 1.11595200 | 1.77135200 | 3.16628100 |
| H | 0.23684700 | 1.90409400 | 2.55245900 |
| C | 1.23361000 | 2.46765000 | 4.37178900 |
| H | 0.44377500 | 3.15028200 | 4.67307700 |
| C | 2.35069500 | 2.26852100 | 5.18361500 |
| H | 2.44666700 | 2.80393000 | 6.12478600 |
| C | 3.34775400 | 1.37177800 | 4.78144400 |
| H | 4.22171100 | 1.21020600 | 5.40738900 |
| C | 3.22503200 | 0.68165800 | 3.57566000 |
| H | 4.00886200 | -0.00722800 | 3.27524200 |
| C | 3.35559100 | 0.75866200 | 0.18155900 |
| C | 4.11824100 | 1.83226000 | 0.67477900 |
| H | 3.91588800 | 2.24632600 | 1.65545200 |
| C | 5.13803800 | 2.40250100 | -0.09252200 |
| H | 5.70580300 | 3.23620300 | 0.31306100 |
| C | 5.42938100 | 1.90852200 | -1.36563100 |
| H | 6.22306900 | 2.35386600 | -1.95958300 |
| C | 4.68368600 | 0.84044300 | -1.86771000 |
| H | 4.88362800 | 0.44448500 | -2.85995800 |
| C | 3.65563000 | 0.28430800 | -1.10630000 |
| H | 3.07145100 | -0.51784000 | -1.53307600 |
| C | 2.62631600 | -1.64948900 | 1.60742700 |
| C | 2.18822600 | -2.23311400 | 2.81107900 |
| C | 3.54570100 | -2.36561600 | 0.82772100 |
| C | 2.64057900 | -3.49037100 | 3.21157500 |
| H | 1.48853100 | -1.70078900 | 3.44622900 |
| C | 3.99934300 | -3.62675800 | 1.22717000 |
| H | 3.92810900 | -1.95029300 | -0.09570000 |
| C | 3.54712000 | -4.19804200 | 2.41705400 |
| H | 2.28403800 | -3.91369100 | 4.14735000 |
| H | 4.71432700 | -4.15656500 | 0.60271100 |
| H | 3.90200800 | -5.17742600 | 2.72713000 |
| C | -2.43095300 | 2.58603300 | -1.13411400 |
| C | -1.98067600 | 3.18800800 | -2.32545400 |
| C | -3.77716800 | 2.18803700 | -1.07432900 |
| C | -2.84684000 | 3.40652800 | -3.39781600 |
| H | -0.94582900 | 3.49002200 | -2.42692100 |
| C | -4.64549300 | 2.40936600 | -2.14656200 |
| H | -4.17342400 | 1.70313200 | -0.19336700 |
| C | -4.18714600 | 3.02279200 | -3.31397200 |
| H | -2.46351000 | 3.87146900 | -4.30209700 |
| H | -5.68318300 | 2.09622400 | -2.06431800 |
| H | -4.86262700 | 3.19338700 | -4.14789900 |
| C | 0.51711600 | 1.09461300 | -3.21514500 |
| C | 1.32662600 | 2.06259500 | -2.60656000 |
| C | 0.12335700 | 1.29298700 | -4.54863500 |
| C | 1.73281000 | 3.20460900 | -3.29756300 |
| H | 1.61601400 | 1.92625200 | -1.57483100 |
| C | 0.51923300 | 2.43980200 | -5.24162800 |
| H | -0.50346300 | 0.56139200 | -5.04780000 |
| C | 1.32292300 | 3.39993900 | -4.61885600 |
| H | 2.34881000 | 3.94403300 | -2.79313300 |
| H | 0.19710700 | 2.58314000 | -6.27004400 |
| H | 1.62296100 | 4.29394800 | -5.15947600 |
| I | -1.39476300 | -0.76157800 | 3.45212600 |
| C | -1.23931100 | -1.42545300 | 1.36322200 |
| C | -2.26925500 | -1.02580000 | 0.47308600 |
| C | -0.68116500 | -2.72605600 | 1.21632900 |
| C | -2.74949000 | -1.93421300 | -0.49622800 |
| H | -2.84801600 | -0.13755200 | 0.66863300 |
| C | -1.13780900 | -3.57989000 | 0.22529200 |
| H | 0.08294100 | -3.06357400 | 1.90395000 |
| C | -2.19751900 | -3.19742700 | -0.62064800 |
| H | -3.57157900 | -1.62848800 | -1.13512800 |
| H | -0.68692500 | -4.56416500 | 0.12519200 |
| H | -2.58038600 | -3.88931600 | -1.36414900 |

**TS-1-2-Br-PMe$_3$**

E = -1797.83515434

$G_{corr}$ = 0.376299

| | | | |
|---|---|---|---|
| Ni | -1.23515300 | -0.74540800 | 0.35925700 |
| P | -0.41258600 | 0.27447900 | -1.44617500 |
| P | -0.56820900 | -2.88579000 | 0.02357100 |
| P | -3.53500700 | -0.92565200 | -0.03821600 |
| C | -0.78466100 | 0.45242300 | 1.72307400 |
| C | -1.47471000 | -0.46800500 | 2.56445000 |
| C | 0.60166700 | 0.68369200 | 1.94410100 |
| Br | -1.85127300 | 2.67100200 | 1.70112100 |
| C | -0.75085500 | -1.17774000 | 3.56317300 |
| H | -2.55637000 | -0.45026800 | 2.63177100 |
| C | 1.28503400 | -0.04644900 | 2.90206600 |
| H | 1.12104700 | 1.42405300 | 1.34748300 |
| C | 0.61016100 | -0.98718800 | 3.71782800 |
| H | -1.29288100 | -1.84363300 | 4.23182700 |
| H | 2.35556300 | 0.10513900 | 3.02465900 |
| H | 1.15922000 | -1.53250900 | 4.48142200 |
| C | -4.39638400 | -2.31153100 | 0.83984600 |
| H | -4.23455900 | -2.21499800 | 1.91913400 |
| H | -5.47566900 | -2.31718200 | 0.64251800 |
| H | -3.97408500 | -3.26971700 | 0.51817800 |
| C | -4.60876700 | 0.50338300 | 0.42556400 |
| H | -5.66899500 | 0.29325700 | 0.23581700 |

| | | | |
|---|---|---|---|
| H | -4.46708700 | 0.73853400 | 1.48450400 |
| H | -4.30153500 | 1.38579700 | -0.14299600 |
| C | -4.08395800 | -1.23399800 | -1.78089400 |
| H | -3.81880200 | -0.37676400 | -2.40858900 |
| H | -3.57368000 | -2.11892700 | -2.17546500 |
| H | -5.16776000 | -1.39176900 | -1.84440100 |
| C | -0.42072400 | -0.73219000 | -2.99808900 |
| H | -0.13842600 | -0.12391200 | -3.86578700 |
| H | 0.28324600 | -1.56581700 | -2.91077000 |
| H | -1.42325600 | -1.14036500 | -3.16057000 |
| C | -1.37056400 | 1.75597500 | -1.98073000 |
| H | -2.37675900 | 1.44853000 | -2.28417200 |
| H | -1.46190000 | 2.44249600 | -1.13415900 |
| H | -0.88621900 | 2.26352600 | -2.82404700 |
| C | 1.31388500 | 0.93451400 | -1.45576800 |
| H | 2.00849700 | 0.17445700 | -1.08407200 |
| H | 1.61313000 | 1.23419700 | -2.46726800 |
| H | 1.37736800 | 1.81015100 | -0.80290400 |
| C | 1.23979400 | -3.11216200 | -0.28563700 |
| H | 1.79613700 | -2.61872100 | 0.51870100 |
| H | 1.51709000 | -4.17296200 | -0.31834800 |
| H | 1.52306200 | -2.64260800 | -1.23358900 |
| C | -1.31818000 | -3.91937900 | -1.31365900 |
| H | -1.15531400 | -3.45720700 | -2.29154500 |
| H | -0.88670800 | -4.92763900 | -1.32211200 |
| H | -2.39715500 | -4.00121900 | -1.14572100 |
| C | -0.81645000 | -3.98245800 | 1.48861000 |
| H | -0.23750000 | -3.59375900 | 2.33090400 |
| H | -1.87340400 | -3.97990900 | 1.77336600 |
| H | -0.50121400 | -5.01054500 | 1.27372600 |

**TS-1-2-Cl-PMe$_3$**

E = -2244.88655801

$G_{corr}$ = 0.375799

| | | | |
|---|---|---|---|
| Ni | -1.23252900 | -0.77061800 | 0.35454000 |
| P | -0.39112000 | 0.25867200 | -1.43565400 |
| P | -0.57644300 | -2.90774000 | -0.00475400 |
| P | -3.53626200 | -0.93447600 | -0.04528100 |
| C | -0.81171000 | 0.42225500 | 1.71381400 |
| C | -1.47089600 | -0.49380800 | 2.58062200 |
| C | 0.55292000 | 0.73837300 | 1.94636900 |
| C | -0.72318600 | -1.14949700 | 3.59695800 |
| H | -2.55301100 | -0.53409500 | 2.63065500 |
| C | 1.26169900 | 0.06361700 | 2.92740900 |
| H | 1.03966300 | 1.49203100 | 1.33877400 |
| C | 0.62776900 | -0.89436600 | 3.75400900 |
| H | -1.23898100 | -1.82481500 | 4.27664800 |
| H | 2.32172700 | 0.27205600 | 3.05724500 |
| H | 1.19668800 | -1.39906200 | 4.53075000 |
| C | -4.40836400 | -2.31869500 | 0.82307900 |
| H | -4.25148300 | -2.22510400 | 1.90338200 |
| H | -5.48661400 | -2.31816000 | 0.62057300 |
| H | -3.98935900 | -3.27825100 | 0.50106900 |
| C | -4.60245000 | 0.50020800 | 0.41607500 |
| H | -5.66013600 | 0.30174700 | 0.20174900 |
| H | -4.48170900 | 0.71859000 | 1.48094100 |
| H | -4.27450400 | 1.38717300 | -0.13347500 |
| C | -4.07187900 | -1.23082000 | -1.79378000 |
| H | -3.79102500 | -0.37525400 | -2.41735300 |
| H | -3.57117000 | -2.12176200 | -2.18684400 |
| H | -5.15711700 | -1.37395200 | -1.86547700 |
| C | -0.45636600 | -0.72279600 | -3.00113400 |
| H | -0.18172500 | -0.10704200 | -3.86594400 |
| H | 0.23681900 | -1.56759000 | -2.93824900 |
| H | -1.46779600 | -1.11339400 | -3.14716700 |
| C | -1.32384400 | 1.77771300 | -1.89879200 |
| H | -2.35560200 | 1.50771900 | -2.14761000 |
| H | -1.35048500 | 2.44937300 | -1.03562800 |
| H | -0.87026200 | 2.28512000 | -2.75914900 |
| C | 1.35451100 | 0.86194300 | -1.46994700 |
| H | 2.03222800 | 0.06450200 | -1.14802900 |
| H | 1.63497800 | 1.18886600 | -2.47834700 |
| H | 1.46772400 | 1.70860800 | -0.78692800 |
| C | 1.22930100 | -3.12577900 | -0.32556500 |
| H | 1.78745700 | -2.62066700 | 0.47013300 |
| H | 1.51269400 | -4.18510800 | -0.35009000 |
| H | 1.50186100 | -2.66394300 | -1.28037900 |
| C | -1.33708900 | -3.94376900 | -1.33217700 |
| H | -1.17889600 | -3.48393000 | -2.31216400 |
| H | -0.90711400 | -4.95260700 | -1.34034200 |
| H | -2.41506200 | -4.02274200 | -1.15721600 |
| C | -0.81284400 | -3.98595000 | 1.47473700 |
| H | -0.23132700 | -3.58125500 | 2.30809600 |
| H | -1.86824100 | -3.98399200 | 1.76514200 |
| H | -0.49325500 | -5.01532000 | 1.27318800 |
| Cl | -1.92004300 | 2.54502000 | 1.66250700 |

**2-I-PMe$_3$**

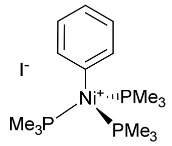


E = -1796.11261245

$G_{corr}$ = 0.377983

| | | | |
|---|---|---|---|
| Ni | -1.34730500 | -1.28806300 | -0.05416300 |
| P | 0.13800300 | -0.01722600 | -1.18121900 |
| P | -0.74326100 | -3.31385500 | -0.98594800 |
| P | -3.38744800 | -1.83341900 | 0.77960900 |
| C | -1.40212600 | 0.17175600 | 1.21355600 |
| C | -0.58311700 | -0.07766000 | 2.33232500 |
| C | -2.13038100 | 1.37032500 | 1.19689000 |
| C | -0.50799000 | 0.82572200 | 3.39705100 |
| H | 0.00752700 | -0.99297300 | 2.37673000 |
| C | -2.06431700 | 2.27712000 | 2.26125200 |
| H | -2.74733200 | 1.61728900 | 0.33740200 |
| C | -1.25519200 | 2.00708100 | 3.36738700 |

| | | | |
|---|---|---|---|
| H | 0.13665000 | 0.60906500 | 4.24593900 |
| H | -2.63841100 | 3.20024500 | 2.22009900 |
| H | -1.19802000 | 2.71332300 | 4.19147700 |
| C | -4.17399900 | -3.48258900 | 0.51731200 |
| H | -3.71755800 | -4.23001100 | 1.17070400 |
| H | -5.24152000 | -3.41777800 | 0.75658500 |
| H | -4.06660900 | -3.79909400 | -0.52460100 |
| C | -3.72250400 | -1.55861500 | 2.56705800 |
| H | -4.76834600 | -1.79599500 | 2.79056600 |
| H | -3.07293400 | -2.20615600 | 3.16517000 |
| H | -3.51828600 | -0.51916300 | 2.83195000 |
| C | -4.60170000 | -0.74448700 | -0.07742700 |
| H | -4.38939900 | 0.30462700 | 0.13486500 |
| H | -4.53288800 | -0.90699700 | -1.15836500 |
| H | -5.61960200 | -0.97687200 | 0.25492300 |
| C | 0.30723400 | -0.41278500 | -2.97778100 |
| H | 1.02730700 | 0.27255900 | -3.43854000 |
| H | 0.63944700 | -1.43776400 | -3.14998800 |
| H | -0.66661300 | -0.27684800 | -3.46050400 |
| C | -0.19920200 | 1.78606700 | -1.29012400 |
| H | -1.20804000 | 1.95713700 | -1.67873500 |
| H | -0.10791900 | 2.25107200 | -0.30809300 |
| H | 0.52949200 | 2.24314900 | -1.96761500 |
| C | 1.84127500 | -0.06956900 | -0.50175200 |
| H | 2.21464300 | -1.09595100 | -0.47971500 |
| H | 2.51211800 | 0.55459800 | -1.10033800 |
| H | 1.81716600 | 0.32185100 | 0.51907200 |
| C | 1.07512900 | -3.57170300 | -1.19335200 |
| H | 1.56618100 | -3.46228800 | -0.22087100 |
| H | 1.25544700 | -4.58580200 | -1.56707500 |
| H | 1.51918100 | -2.86034200 | -1.89179100 |
| C | -1.42120800 | -3.78217700 | -2.63240800 |
| H | -1.10520100 | -3.05097000 | -3.38243100 |
| H | -1.07147600 | -4.77688400 | -2.93058100 |
| H | -2.51570500 | -3.78386200 | -2.59297000 |
| C | -1.12546400 | -4.79071300 | 0.05803300 |
| H | -0.96087900 | -4.55301400 | 1.11387900 |
| H | -2.15987700 | -5.10749500 | -0.07955400 |
| H | -0.47011100 | -5.62201600 | -0.22415700 |
| I | 2.81328000 | 3.68537600 | 0.96125000 |

**2-I-$PMe_2Ph$**

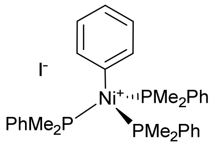


E = -2371.42764175

$G_{corr}$ = 0.528074

| | | | |
|---|---|---|---|
| Ni | -0.93316700 | 0.61845000 | -0.00635200 |
| P | -0.22208100 | 1.02092400 | -2.19260500 |
| P | 1.11506900 | -0.05969100 | 0.80551100 |
| P | -2.99989100 | 1.40368700 | -0.37598200 |
| C | 1.49144800 | 1.61233000 | -2.45954500 |
| C | 1.78079800 | 2.94429200 | -2.11336300 |
| H | 0.99525600 | 3.58972400 | -1.72597600 |
| C | 3.07396000 | 3.44542100 | -2.25396700 |
| H | 3.28586700 | 4.47609100 | -1.98256000 |
| C | 4.09701700 | 2.62168400 | -2.73590100 |
| H | 5.10676600 | 3.00982600 | -2.83720500 |
| C | 3.81472900 | 1.30185700 | -3.09310100 |
| H | 4.60250300 | 0.66018200 | -3.47853500 |
| C | 2.51625700 | 0.79919600 | -2.96166400 |
| H | 2.31275800 | -0.22656100 | -3.25384500 |
| C | -1.10451400 | 2.08001100 | -3.41792800 |
| H | -2.15989600 | 1.81491800 | -3.49785400 |
| H | -0.63204500 | 1.93687000 | -4.39520200 |
| H | -1.02745300 | 3.13158700 | -3.13450600 |
| C | -0.34322200 | -0.63801200 | -2.98027400 |
| H | -1.40116300 | -0.92107800 | -2.96527500 |
| H | 0.21637200 | -1.38598600 | -2.41389100 |
| H | 0.00159800 | -0.62032500 | -4.01913800 |
| C | 1.14716200 | -0.62951700 | 2.55119800 |
| C | 1.45641700 | 0.26353800 | 3.58868100 |
| H | 1.72115700 | 1.29385000 | 3.36992700 |
| C | 1.41400800 | -0.15529900 | 4.92019600 |
| H | 1.65607300 | 0.54785800 | 5.71276900 |
| C | 1.05762700 | -1.46874400 | 5.23213400 |
| H | 1.01822900 | -1.79257700 | 6.26864300 |
| C | 0.74916600 | -2.36439700 | 4.20474700 |
| H | 0.46676900 | -3.38728700 | 4.43845000 |
| C | 0.79524300 | -1.94965100 | 2.87368300 |
| H | 0.53333300 | -2.65521100 | 2.09190400 |
| C | 1.86088200 | -1.47795700 | -0.10595000 |
| H | 1.14105000 | -2.29705100 | -0.18802700 |
| H | 2.75963100 | -1.83314900 | 0.40818900 |
| H | 2.13178600 | -1.14390100 | -1.11082000 |
| C | 2.47007900 | 1.19390400 | 0.77180600 |
| H | 2.10410600 | 2.16724100 | 1.10966700 |
| H | 2.84129300 | 1.29838900 | -0.24767800 |
| H | 3.29189900 | 0.87031300 | 1.41792400 |
| C | -2.92560300 | 3.11716200 | -1.01631200 |
| C | -3.84648100 | 3.63320000 | -1.93768500 |
| H | -4.64668700 | 3.01278400 | -2.32889900 |
| C | -3.71454600 | 4.94686000 | -2.39407000 |
| H | -4.42205500 | 5.33409000 | -3.12193600 |
| C | -2.67299200 | 5.75393500 | -1.92877200 |
| H | -2.57251200 | 6.77349900 | -2.29092300 |
| C | -1.75649100 | 5.24725500 | -1.00150900 |
| H | -0.94298900 | 5.87046400 | -0.63946700 |
| C | -1.87977500 | 3.93191500 | -0.55199900 |
| H | -1.14997600 | 3.52088600 | 0.14385500 |
| C | -4.20131000 | 1.56148100 | 1.00890000 |
| H | -4.44505300 | 0.57375100 | 1.40761400 |
| H | -5.10956400 | 2.04289900 | 0.63083400 |
| H | -3.77444200 | 2.17114000 | 1.80917200 |
| C | -3.93471200 | 0.37476000 | -1.57178800 |
| H | -4.00956700 | -0.63156400 | -1.14541300 |
| H | -3.41764900 | 0.31391800 | -2.53077600 |

| | | | |
|---|---|---|---|
| H | -4.94369100 | 0.76398300 | -1.73494300 |
| C | -1.73308900 | -0.22470700 | 1.52769500 |
| C | -2.26207200 | -1.51159100 | 1.32920100 |
| C | -1.81720100 | 0.32846600 | 2.81520800 |
| C | -2.84055300 | -2.22892000 | 2.38429000 |
| H | -2.22579800 | -1.96406100 | 0.33889500 |
| C | -2.39034200 | -0.38235700 | 3.87240400 |
| H | -1.41541100 | 1.32170600 | 3.00190900 |
| C | -2.90406000 | -1.66633400 | 3.66151800 |
| H | -3.23924500 | -3.22563300 | 2.20631500 |
| H | -2.43136800 | 0.06321800 | 4.86391100 |
| H | -3.34887200 | -2.22097100 | 4.48389400 |
| I | -4.74170800 | 1.36343400 | -5.45802900 |

**2-I-PMePh$_2$**

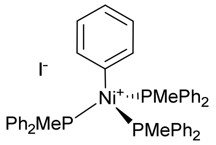


E = -2946.88279278

$G_{corr}$ = 0.674795

| | | | |
|---|---|---|---|
| Ni | 9.47698500 | 10.32069500 | 4.06894500 |
| P | 10.06328100 | 11.20336200 | 2.04354300 |
| C | 8.80627800 | 11.80614100 | 0.83337800 |
| C | 8.42108500 | 13.15634000 | 0.82308500 |
| H | 8.86331700 | 13.86140400 | 1.51845800 |
| C | 7.44745700 | 13.60800500 | -0.06972400 |
| H | 7.15909800 | 14.65531900 | -0.05830100 |
| C | 6.84264300 | 12.72009400 | -0.96165800 |
| H | 6.08412800 | 13.07365600 | -1.65474000 |
| C | 7.22133100 | 11.37481000 | -0.96096400 |
| H | 6.76247900 | 10.67705400 | -1.65641000 |
| C | 8.19665500 | 10.92033400 | -0.07163900 |
| H | 8.48402400 | 9.87361500 | -0.09053500 |
| C | 10.86761100 | 9.83067600 | 1.12892900 |
| C | 10.39771800 | 8.52468500 | 1.34359600 |
| H | 9.59091200 | 8.35556500 | 2.05316100 |
| C | 10.98051200 | 7.44385500 | 0.68124500 |
| H | 10.61173500 | 6.43717000 | 0.85800600 |
| C | 12.04740400 | 7.65819800 | -0.19585800 |
| H | 12.51093800 | 6.81750300 | -0.70473200 |
| C | 12.52052800 | 8.95493400 | -0.41360500 |
| H | 13.35159600 | 9.12448400 | -1.09280000 |
| C | 11.93129800 | 10.03917800 | 0.24080200 |
| H | 12.31030400 | 11.04074000 | 0.06186000 |
| C | 11.28020400 | 12.58127900 | 2.08404100 |
| H | 11.45610200 | 12.98274200 | 1.08249500 |
| H | 12.21570800 | 12.21937700 | 2.51448200 |
| H | 10.88782000 | 13.37460700 | 2.72585400 |
| P | 7.23491000 | 10.94998900 | 4.04994400 |
| C | 6.31666000 | 10.72214100 | 5.62798400 |
| C | 6.74772400 | 11.43926700 | 6.75636800 |
| H | 7.58848900 | 12.12449000 | 6.68716800 |
| C | 6.10323300 | 11.27890200 | 7.98283200 |
| H | 6.45245900 | 11.84106500 | 8.84451500 |
| C | 5.02018200 | 10.40164300 | 8.09663400 |
| H | 4.51853400 | 10.27526000 | 9.05232000 |
| C | 4.58552200 | 9.68877100 | 6.97738100 |
| H | 3.74884100 | 9.00012500 | 7.05830200 |
| C | 5.22863600 | 9.84641700 | 5.74713800 |
| H | 4.88675700 | 9.26838700 | 4.89574600 |
| C | 7.21253800 | 12.77911900 | 3.85917100 |
| C | 6.11903200 | 13.46492600 | 3.31016600 |
| H | 5.24726600 | 12.92357100 | 2.95658300 |
| C | 6.14890300 | 14.85550900 | 3.19634900 |
| H | 5.30108700 | 15.37685100 | 2.76001800 |
| C | 7.26320700 | 15.57553100 | 3.63847800 |
| H | 7.28159600 | 16.65834500 | 3.54962100 |
| C | 8.34882600 | 14.90077800 | 4.20175100 |
| H | 9.21286700 | 15.45089500 | 4.56259300 |
| C | 8.32456300 | 13.50874100 | 4.30678000 |
| H | 9.17570900 | 12.99153300 | 4.73967400 |
| C | 6.08185600 | 10.32207900 | 2.76310700 |
| H | 5.06305000 | 10.68741600 | 2.91942100 |
| H | 6.07094900 | 9.22982900 | 2.78576700 |
| H | 6.43613300 | 10.64816400 | 1.78330600 |
| P | 9.42633400 | 8.77091800 | 5.70288600 |
| C | 10.76888300 | 7.51995600 | 5.55414600 |
| C | 11.86240200 | 7.51423500 | 6.43167900 |
| H | 11.91819700 | 8.22215000 | 7.25173100 |
| C | 12.91326400 | 6.61388900 | 6.24209300 |
| H | 13.75558200 | 6.62321100 | 6.92857500 |
| C | 12.88751900 | 5.71520700 | 5.17392500 |
| H | 13.70866800 | 5.01918000 | 5.02551700 |
| C | 11.79991100 | 5.71506600 | 4.29594600 |
| H | 11.77081200 | 5.01909800 | 3.46172100 |
| C | 10.74781000 | 6.61097700 | 4.48458900 |
| H | 9.91209500 | 6.59931600 | 3.79208300 |
| C | 7.91671300 | 7.72570900 | 5.66471700 |
| C | 7.38104100 | 7.39056200 | 4.41055800 |
| H | 7.84308200 | 7.78481500 | 3.50874300 |
| C | 6.25606900 | 6.57109500 | 4.31395300 |
| H | 5.85209200 | 6.31825600 | 3.33727200 |
| C | 5.64669000 | 6.08724300 | 5.47527100 |
| H | 4.76497600 | 5.45617600 | 5.40513300 |
| C | 6.17045500 | 6.42145900 | 6.72662700 |
| H | 5.69517400 | 6.05332700 | 7.63167500 |
| C | 7.30312600 | 7.23305200 | 6.82440900 |
| H | 7.69071200 | 7.48856900 | 7.80545300 |
| C | 9.56129200 | 9.41653700 | 7.41090900 |
| H | 9.72192700 | 8.61385300 | 8.13618900 |
| H | 8.64365300 | 9.95434000 | 7.65683300 |
| H | 10.39466500 | 10.12159500 | 7.45611300 |
| C | 11.39306200 | 10.42719000 | 4.45787000 |
| C | 11.70278400 | 11.45595700 | 5.36737400 |
| C | 12.45279500 | 9.66544700 | 3.94390100 |
| C | 13.02500500 | 11.72399000 | 5.73799300 |
| H | 10.91090300 | 12.05322100 | 5.81404200 |

| | | | |
|---|---|---|---|
| C | 13.77761500 | 9.93459500 | 4.30476000 |
| H | 12.25141600 | 8.84491600 | 3.26391700 |
| C | 14.07015100 | 10.96697000 | 5.20071200 |
| H | 13.22820400 | 12.52188700 | 6.44893500 |
| H | 14.58013300 | 9.32734800 | 3.89121300 |
| H | 15.09932400 | 11.17278100 | 5.48485100 |
| I | 9.81287400 | 13.92470900 | 8.17378800 |

**2-I-PPh$_3$**

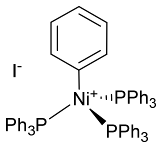


E = -3522.24185588

$G_{corr}$ = 0.824754

| | | | |
|---|---|---|---|
| Ni | 9.35558800 | 10.48196500 | 4.19095400 |
| P | 9.33806700 | 10.20429700 | 1.79316600 |
| C | 7.96021900 | 9.26553800 | 1.03570300 |
| C | 6.75999300 | 9.90573200 | 0.68930600 |
| H | 6.67119500 | 10.98238100 | 0.76271200 |
| C | 5.66271600 | 9.16006700 | 0.25446200 |
| H | 4.74295500 | 9.67392600 | -0.01081700 |
| C | 5.74405200 | 7.76823300 | 0.17010100 |
| H | 4.88745200 | 7.18989500 | -0.16507500 |
| C | 6.92974900 | 7.12287300 | 0.52949300 |
| H | 7.00074900 | 6.03973600 | 0.48038800 |
| C | 8.02848900 | 7.86321200 | 0.96506700 |
| H | 8.93483300 | 7.34402000 | 1.25447900 |
| C | 10.89720200 | 9.46296200 | 1.13876800 |
| C | 12.09362300 | 9.81321300 | 1.78020900 |
| H | 12.06879800 | 10.40696400 | 2.68702200 |
| C | 13.32454900 | 9.41092100 | 1.26398900 |
| H | 14.23660900 | 9.67395500 | 1.79073000 |
| C | 13.37259200 | 8.63978400 | 0.10081400 |
| H | 14.32958700 | 8.31063000 | -0.29470700 |
| C | 12.18677800 | 8.29392000 | -0.55149900 |
| H | 12.21676800 | 7.70324600 | -1.46330800 |
| C | 10.95417000 | 8.71282800 | -0.04562100 |
| H | 10.04719400 | 8.45724500 | -0.58271600 |
| P | 8.04590200 | 12.38190700 | 4.44131000 |
| C | 7.09975100 | 12.24380700 | 6.02106800 |
| C | 6.86620500 | 13.33692000 | 6.86596200 |
| H | 7.25012000 | 14.31984900 | 6.61613100 |
| C | 6.14643600 | 13.16785300 | 8.05203200 |
| H | 5.97861200 | 14.02306300 | 8.70103400 |
| C | 5.64975200 | 11.91157900 | 8.40592500 |
| H | 5.09282800 | 11.78504400 | 9.33023400 |
| C | 5.88304400 | 10.81568500 | 7.57066000 |
| H | 5.51540200 | 9.82962500 | 7.84167200 |
| C | 6.61042300 | 10.98117000 | 6.39299400 |
| H | 6.82715900 | 10.11817200 | 5.77501000 |
| C | 8.83714300 | 14.03860000 | 4.51089100 |
| C | 8.10737100 | 15.22525200 | 4.32243300 |
| H | 7.04216500 | 15.18746700 | 4.11804000 |
| C | 8.74761800 | 16.46347600 | 4.38881700 |
| H | 8.17374600 | 17.37319100 | 4.23476600 |
| C | 10.11855000 | 16.53340600 | 4.65202300 |
| H | 10.61529900 | 17.49863700 | 4.69962100 |
| C | 10.84696700 | 15.35922000 | 4.85643400 |
| H | 11.91197600 | 15.40314100 | 5.06682400 |
| C | 10.21050200 | 14.11893500 | 4.78675200 |
| H | 10.78320700 | 13.21277600 | 4.94473900 |
| P | 9.29762900 | 8.26094700 | 4.78439400 |
| C | 10.40071800 | 7.10217600 | 3.88040300 |
| C | 11.77144800 | 7.40577000 | 3.83196800 |
| H | 12.15872500 | 8.29896200 | 4.31275400 |
| C | 12.65761400 | 6.56899400 | 3.15319100 |
| H | 13.71202300 | 6.82592300 | 3.13266200 |
| C | 12.18320600 | 5.43529600 | 2.49110000 |
| H | 12.87236000 | 4.79292400 | 1.94967000 |
| C | 10.81904400 | 5.13517800 | 2.51930400 |
| H | 10.44058700 | 4.25818400 | 2.00070400 |
| C | 9.93082600 | 5.95882700 | 3.21342600 |
| H | 8.87545000 | 5.71215500 | 3.21699500 |
| C | 7.57902700 | 7.64624600 | 4.53624400 |
| C | 6.62033500 | 8.45692200 | 3.91284900 |
| H | 6.89426800 | 9.43961100 | 3.54904000 |
| C | 5.30540000 | 8.01510200 | 3.75644600 |
| H | 4.57989000 | 8.65993900 | 3.26970700 |
| C | 4.93614700 | 6.74987000 | 4.21461500 |
| H | 3.91466600 | 6.40091400 | 4.08961500 |
| C | 5.88291400 | 5.93249400 | 4.84119600 |
| H | 5.59909100 | 4.94913700 | 5.20580700 |
| C | 7.19346900 | 6.37884100 | 5.01010700 |
| H | 7.91473100 | 5.74456500 | 5.51682800 |
| C | 9.58452000 | 7.90507700 | 6.57131100 |
| C | 10.86257600 | 7.60673200 | 7.06489000 |
| C | 8.52453300 | 8.06961300 | 7.47848600 |
| C | 11.07343200 | 7.47630200 | 8.43875900 |
| H | 11.70226900 | 7.48055600 | 6.39174900 |
| C | 8.73863900 | 7.93428400 | 8.84990600 |
| H | 7.53023200 | 8.31331200 | 7.12094000 |
| C | 10.01539900 | 7.64173400 | 9.33514800 |
| H | 12.07079500 | 7.24810000 | 8.80418600 |
| H | 7.90759500 | 8.06725400 | 9.53760300 |
| H | 10.18502500 | 7.54333600 | 10.40396900 |
| C | 6.67956600 | 12.56409200 | 3.19951100 |
| C | 6.83045000 | 13.37612200 | 2.06206900 |
| C | 5.47541500 | 11.85705700 | 3.36363600 |
| C | 5.79883500 | 13.48971900 | 1.12777100 |
| H | 7.74619300 | 13.93096800 | 1.89960500 |
| C | 4.44953100 | 11.96674100 | 2.42354900 |
| H | 5.32103700 | 11.22525000 | 4.23070800 |
| C | 4.60456400 | 12.78695300 | 1.30366400 |
| H | 5.93816800 | 14.12725000 | 0.25933600 |
| H | 3.52548800 | 11.41529300 | 2.57471400 |
| H | 3.80354500 | 12.87681300 | 0.57503700 |
| C | 9.45268300 | 11.83337500 | 0.94571900 |

| | | | |
|---|---|---|---|
| C | 10.18258300 | 12.82161100 | 1.62179700 |
| C | 8.95366100 | 12.10261000 | -0.33528100 |
| C | 10.37649400 | 14.07891900 | 1.04972600 |
| H | 10.58293500 | 12.60885300 | 2.60801100 |
| C | 9.14019200 | 13.36446300 | -0.90392900 |
| H | 8.41293600 | 11.34053400 | -0.88698000 |
| C | 9.84235300 | 14.35611700 | -0.21165900 |
| H | 10.92543500 | 14.84008100 | 1.59695000 |
| H | 8.73708700 | 13.57204400 | -1.89150100 |
| H | 9.97857800 | 15.33753600 | -0.65758500 |
| C | 10.29692000 | 10.84724400 | 5.84082200 |
| C | 9.88322800 | 11.06101000 | 7.15975600 |
| C | 11.67531400 | 10.91487700 | 5.56393300 |
| C | 10.80842300 | 11.36991200 | 8.16368300 |
| H | 8.84090200 | 10.96629200 | 7.43094800 |
| C | 12.60294300 | 11.22961500 | 6.56329600 |
| H | 12.05447400 | 10.71609500 | 4.56488400 |
| C | 12.16990800 | 11.46515000 | 7.86977600 |
| H | 10.45676900 | 11.52515800 | 9.18136700 |
| H | 13.65995200 | 11.26353200 | 6.31408800 |
| H | 12.88804100 | 11.69946500 | 8.65141100 |
| I | 15.56255500 | 9.03535600 | 4.75823300 |

**2-Br-$PMe_3$**

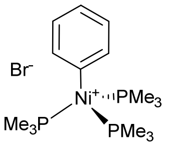


E = -1797.88492862

$G_{corr}$ = 0.379868

| | | | |
|---|---|---|---|
| Ni | -1.32885000 | -1.27301000 | -0.04172500 |
| P | 0.20292200 | -0.14652300 | -1.23590700 |
| P | -0.87418800 | -3.36374000 | -0.90011100 |
| P | -3.42015100 | -1.63019400 | 0.77462500 |
| C | -1.25284400 | 0.20514100 | 1.20714700 |
| C | -0.47441800 | -0.11853500 | 2.33617000 |
| C | -1.88761100 | 1.45534300 | 1.19442200 |
| Br | 2.69629100 | 3.31741200 | 0.66042400 |
| C | -0.36906200 | 0.75358300 | 3.42425100 |
| H | 0.05279800 | -1.07220900 | 2.37381000 |
| C | -1.78737100 | 2.33372800 | 2.28009300 |
| H | -2.46682400 | 1.76182200 | 0.32812300 |
| C | -1.03625400 | 1.98235200 | 3.40426700 |
| H | 0.23640300 | 0.47521800 | 4.28411300 |
| H | -2.29350300 | 3.29595800 | 2.24360900 |
| H | -0.95638800 | 2.66400500 | 4.24700000 |
| C | -4.34160100 | -3.21791200 | 0.58274300 |
| H | -3.97045800 | -3.96127900 | 1.29313500 |
| H | -5.40466000 | -3.04381500 | 0.78369600 |
| H | -4.23552000 | -3.60490400 | -0.43505000 |
| C | -3.78404900 | -1.22210900 | 2.53140800 |
| H | -4.84910200 | -1.37713200 | 2.73658300 |
| H | -3.19529100 | -1.87180300 | 3.18710700 |
| H | -3.51923500 | -0.18352000 | 2.74081700 |
| C | -4.50769000 | -0.49347800 | -0.18506100 |
| H | -4.16326200 | 0.53766000 | -0.08592700 |
| H | -4.47383300 | -0.77398000 | -1.24336900 |
| H | -5.54197100 | -0.56287300 | 0.17031300 |
| C | 0.30829700 | -0.65782500 | -3.00936900 |
| H | 1.04754100 | -0.03484300 | -3.52519900 |
| H | 0.59150700 | -1.70512100 | -3.12700800 |
| H | -0.66919600 | -0.50673600 | -3.47989600 |
| C | -0.06454900 | 1.65830500 | -1.44176900 |
| H | -1.09584100 | 1.84923600 | -1.75548500 |
| H | 0.14818800 | 2.18465000 | -0.50979800 |
| H | 0.62015400 | 2.03415900 | -2.20915600 |
| C | 1.91563800 | -0.25285800 | -0.58759700 |
| H | 2.16493100 | -1.28617800 | -0.33410000 |
| H | 2.62872200 | 0.13005000 | -1.32481800 |
| H | 1.98860500 | 0.37485500 | 0.30564900 |
| C | 0.92668800 | -3.74242100 | -1.07265900 |
| H | 1.39957500 | -3.66426900 | -0.08803600 |
| H | 1.05246000 | -4.76564300 | -1.44367300 |
| H | 1.43185300 | -3.05713100 | -1.75559800 |
| C | -1.57884400 | -3.86745200 | -2.52466800 |
| H | -1.20206500 | -3.21101400 | -3.31416700 |
| H | -1.31227900 | -4.90387900 | -2.76038700 |
| H | -2.66974800 | -3.77659400 | -2.49376900 |
| C | -1.36109200 | -4.75926900 | 0.20736100 |
| H | -1.15334800 | -4.49717000 | 1.24987000 |
| H | -2.42308700 | -4.98396300 | 0.10305400 |
| H | -0.79024100 | -5.65686500 | -0.05449800 |

**2-Cl-$PMe_3$**

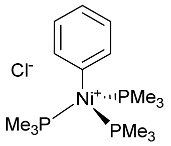


E = -2244.9507208

$G_{corr}$ = 0.382069

| | | | |
|---|---|---|---|
| Ni | -1.31385700 | -1.15926500 | -0.02597300 |
| P | 0.87190400 | -0.66263600 | -0.52962100 |
| P | -1.50140100 | -2.82142200 | -1.61872800 |
| P | -3.40213100 | -1.33931800 | 0.86406500 |
| C | -1.04602300 | 0.14308100 | 1.37975500 |
| C | -0.64618800 | -0.24063400 | 2.67265600 |
| C | -1.24315900 | 1.51413100 | 1.13611300 |
| C | -0.44766500 | 0.70524400 | 3.68470100 |
| H | -0.48415000 | -1.29443200 | 2.89514900 |
| C | -1.03507300 | 2.46586500 | 2.14035700 |
| H | -1.57898700 | 1.82723100 | 0.14973500 |
| C | -0.63699500 | 2.06569800 | 3.42057000 |
| H | -0.14074000 | 0.38177600 | 4.67773100 |
| H | -1.19220100 | 3.52142100 | 1.92576400 |
| H | -0.47989100 | 2.80407900 | 4.20331800 |
| C | -3.46710800 | -2.77354900 | 2.02137300 |

| | | | |
|---|---|---|---|
| H | -2.73170800 | -2.62414100 | 2.81906200 |
| H | -4.46187700 | -2.88325400 | 2.46841600 |
| H | -3.21061300 | -3.69231500 | 1.48364200 |
| C | -4.02328500 | 0.03787400 | 1.92121400 |
| H | -5.01781500 | -0.22354700 | 2.29968000 |
| H | -3.35095300 | 0.22245000 | 2.76053200 |
| H | -4.09465100 | 0.94976900 | 1.32131000 |
| C | -4.87351300 | -1.58555400 | -0.22447900 |
| H | -4.73343600 | -0.99053900 | -1.13103100 |
| H | -5.00384100 | -2.63660800 | -0.48378900 |
| H | -5.77379400 | -1.24765700 | 0.29980100 |
| C | 2.06114500 | -1.88818400 | -1.24685900 |
| H | 2.98984900 | -1.36955500 | -1.51021800 |
| H | 2.29221800 | -2.65228400 | -0.49744600 |
| H | 1.67555200 | -2.37963600 | -2.14168400 |
| C | 0.87231700 | 0.69348700 | -1.77115100 |
| H | 0.30067900 | 0.39382400 | -2.65204000 |
| H | 0.37927200 | 1.56670500 | -1.33641300 |
| H | 1.89819800 | 0.95519400 | -2.05504200 |
| C | 1.95775600 | 0.01810400 | 0.79742400 |
| H | 2.05401000 | -0.70455800 | 1.61293100 |
| H | 2.94988400 | 0.22045700 | 0.37853800 |
| H | 1.53955600 | 0.94248000 | 1.19943000 |
| C | -1.00237500 | -2.30761200 | -3.31676700 |
| H | 0.00448600 | -1.88636300 | -3.34100700 |
| H | -1.05097000 | -3.15705300 | -4.00781800 |
| H | -1.70066300 | -1.52537500 | -3.62915300 |
| C | -3.09176800 | -3.66586200 | -2.02131300 |
| H | -3.80594600 | -2.95359600 | -2.43965900 |
| H | -2.90017800 | -4.44996100 | -2.76296100 |
| H | -3.51814100 | -4.12966900 | -1.12755200 |
| C | -0.47920200 | -4.31413200 | -1.23834000 |
| H | 0.55240700 | -4.05168500 | -1.00078700 |
| H | -0.90888700 | -4.81262900 | -0.36176000 |
| H | -0.48608800 | -5.01724100 | -2.07927600 |
| Cl | -2.83582000 | 0.67862500 | -2.23140800 |

**3-I-PMe$_3$**

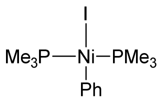


E = -1334.92584591

$G_{corr}$ = 0.268058

| | | | |
|---|---|---|---|
| Ni | -1.11476700 | 0.34353300 | -0.80171100 |
| P | -2.83254200 | 1.74712700 | -0.38873500 |
| C | -4.00662500 | 0.92538200 | 0.76281300 |
| C | -3.88587800 | 2.27764000 | -1.80325600 |
| C | -2.49905500 | 3.35681700 | 0.43969900 |
| H | -4.84991500 | 1.58086500 | 1.00756600 |
| H | -3.47554500 | 0.66042300 | 1.68321000 |
| H | -4.66215500 | 2.97009300 | -1.45845000 |
| H | -4.35282900 | 1.40770400 | -2.27045600 |
| H | -1.83979900 | 3.96578500 | -0.18646000 |
| H | -2.00807800 | 3.19413800 | 1.40200500 |
| P | 0.67196600 | -1.01929300 | -1.00227300 |
| C | 1.20444200 | -1.43400100 | -2.71524700 |
| C | 2.26611400 | -0.53010400 | -0.22479400 |
| C | 0.33173900 | -2.65216500 | -0.22606000 |
| H | 1.50239900 | -0.51567800 | -3.23271500 |
| H | 0.37503800 | -1.88799600 | -3.26277500 |
| H | 2.62661500 | 0.40042500 | -0.67276500 |
| H | 3.01206500 | -1.31778000 | -0.37822100 |
| H | 0.08847000 | -2.50008300 | 0.83094700 |
| H | 1.20153800 | -3.31416100 | -0.30369400 |
| H | -4.37744700 | 0.00685200 | 0.29839000 |
| H | -3.26325900 | 2.78024000 | -2.55092300 |
| H | -3.44119600 | 3.89392800 | 0.59659400 |
| H | 2.05372800 | -2.12634000 | -2.69588600 |
| H | 2.12586500 | -0.36330300 | 0.84626900 |
| H | -0.52734600 | -3.11633300 | -0.71862200 |
| C | -0.15254900 | 1.29019700 | 0.52112300 |
| C | -0.26054300 | 0.91129300 | 1.87044600 |
| C | 0.43879600 | 1.59412800 | 2.87347000 |
| C | 1.26406700 | 2.67393200 | 2.54440800 |
| C | 1.38251300 | 3.06221400 | 1.20580200 |
| C | 0.68143200 | 2.37678600 | 0.20850500 |
| H | -0.90022700 | 0.07401900 | 2.14459000 |
| H | 0.33846200 | 1.28235600 | 3.91130400 |
| H | 1.80787900 | 3.20608300 | 3.32099000 |
| H | 2.02175900 | 3.90131500 | 0.93810200 |
| H | 0.78509800 | 2.69294300 | -0.82780600 |
| I | -2.57756700 | -1.17052900 | -2.44665100 |

**3-I-PMe$_2$Ph**

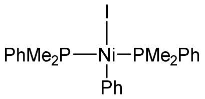


E = -1718.51055427

$G_{corr}$ = 0.367239

| | | | |
|---|---|---|---|
| Ni | -0.88779600 | 1.28886800 | 0.17164000 |
| P | -1.92336000 | 0.82913900 | -1.78754000 |
| P | 0.26010600 | 1.35214600 | 2.12148600 |
| C | -0.67528100 | 0.97875500 | -3.12273600 |
| C | 0.17869600 | 2.09458200 | -3.08611000 |
| H | 0.07412500 | 2.82157300 | -2.28383300 |
| C | 1.16445900 | 2.26221000 | -4.05888300 |
| H | 1.81846800 | 3.12936100 | -4.02106900 |
| C | 1.31626700 | 1.31002200 | -5.07201300 |
| H | 2.08868300 | 1.43527000 | -5.82615300 |
| C | 0.47624200 | 0.19482700 | -5.10936500 |
| H | 0.59386700 | -0.55021000 | -5.89191200 |
| C | -0.51654700 | 0.02775000 | -4.13975100 |
| H | -1.15528700 | -0.84925400 | -4.17888600 |
| C | -3.30824500 | 1.89345700 | -2.38107900 |
| H | -4.11875700 | 1.87470800 | -1.64533200 |
| H | -3.67703700 | 1.52215900 | -3.34353200 |

| | | | |
|---|---|---|---|
| H | -2.96523600 | 2.92386300 | -2.49482700 |
| C | -2.66097000 | -0.84540100 | -1.96651600 |
| H | -3.37488400 | -0.99081900 | -1.14915000 |
| H | -1.89174700 | -1.61607000 | -1.89342700 |
| H | -3.19204100 | -0.93927300 | -2.91917200 |
| C | -0.74057700 | 0.45272400 | 3.36839800 |
| C | -2.11951100 | 0.72377300 | 3.40708900 |
| H | -2.54218400 | 1.44772000 | 2.71415900 |
| C | -2.94526900 | 0.06053800 | 4.31509500 |
| H | -4.00955900 | 0.27958400 | 4.33633200 |
| C | -2.40512000 | -0.89018000 | 5.18704500 |
| H | -3.04869500 | -1.41293900 | 5.88961000 |
| C | -1.03722900 | -1.16952100 | 5.14929900 |
| H | -0.61333600 | -1.91059800 | 5.82194200 |
| C | -0.20644700 | -0.50179600 | 4.24501200 |
| H | 0.85305700 | -0.73741600 | 4.22634800 |
| C | 1.92462100 | 0.57195000 | 2.14644100 |
| H | 1.86061600 | -0.48950200 | 1.90095000 |
| H | 2.40016800 | 0.69913700 | 3.12415800 |
| H | 2.54105900 | 1.06613500 | 1.38830000 |
| C | 0.61216400 | 2.96358700 | 2.94520800 |
| H | -0.32225000 | 3.48455000 | 3.16372400 |
| H | 1.21182800 | 3.58905000 | 2.27601100 |
| H | 1.16223600 | 2.79221700 | 3.87704000 |
| C | -0.19393500 | -0.48166100 | 0.02175500 |
| C | 0.93378300 | -0.74482900 | -0.77601900 |
| C | -0.78247000 | -1.56246300 | 0.70173200 |
| C | 1.45418100 | -2.03833900 | -0.89251800 |
| H | 1.40930000 | 0.06749900 | -1.31981000 |
| C | -0.26443000 | -2.85765800 | 0.59114200 |
| H | -1.65219600 | -1.39181000 | 1.33091600 |
| C | 0.85690500 | -3.10151300 | -0.20804800 |
| H | 2.32713000 | -2.21452900 | -1.51788300 |
| H | -0.73844700 | -3.67613600 | 1.12924400 |
| H | 1.26023700 | -4.10739200 | -0.29617100 |
| I | -1.80826200 | 3.77488400 | 0.35355800 |

## 3-I-PMePh$_2$

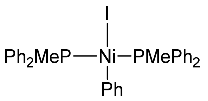


E = -2102.09126305

$G_{corr}$ = 0.464839

| | | | |
|---|---|---|---|
| Ni | -0.08211300 | 1.08796500 | -0.07821600 |
| P | 1.40454700 | 0.74332100 | -1.73438000 |
| C | 0.50619900 | 0.95557600 | -3.32110600 |
| C | 0.04501500 | 2.24782700 | -3.62684300 |
| H | 0.27767600 | 3.08052200 | -2.96833400 |
| C | -0.72810000 | 2.47045300 | -4.76675600 |
| H | -1.07753100 | 3.47454800 | -4.99252800 |
| C | -1.05479900 | 1.40449000 | -5.61045700 |
| H | -1.65854100 | 1.57655400 | -6.49748300 |
| C | -0.60502200 | 0.11715900 | -5.30748700 |
| H | -0.85929600 | -0.71651200 | -5.95666500 |
| C | 0.16694300 | -0.11053300 | -4.16546000 |
| H | 0.49602000 | -1.11775700 | -3.93265000 |
| P | -1.64881200 | 1.91993700 | 1.32409500 |
| C | -2.53698900 | 3.20179400 | 0.35907200 |
| C | -2.70189200 | 2.99081300 | -1.02110200 |
| C | -3.02546600 | 4.38427800 | 0.93415000 |
| C | -3.34331000 | 3.94576800 | -1.81030600 |
| H | -2.31910900 | 2.08136000 | -1.47616500 |
| C | -3.66543400 | 5.33960900 | 0.14042800 |
| H | -2.90872200 | 4.57015300 | 1.99726000 |
| C | -3.82457700 | 5.12336500 | -1.23076800 |
| H | -3.45904100 | 3.77006000 | -2.87640100 |
| H | -4.03749300 | 6.25394500 | 0.59497900 |
| H | -4.31958300 | 5.87072000 | -1.84522100 |
| C | 0.99270800 | 2.54184100 | 0.50622100 |
| C | 1.99885500 | 2.30174600 | 1.45884700 |
| H | 2.15173200 | 1.29291700 | 1.83856700 |
| C | 2.81231800 | 3.33790700 | 1.92999500 |
| H | 3.58366200 | 3.12686800 | 2.66803500 |
| C | 0.83268500 | 3.85612300 | 0.03627600 |
| H | 0.05976600 | 4.07476300 | -0.69649800 |
| C | 2.63609800 | 4.64213700 | 1.45598800 |
| H | 3.26658000 | 5.44889200 | 1.82150900 |
| C | 1.64277500 | 4.89679000 | 0.50605500 |
| H | 1.49655500 | 5.90690800 | 0.12853200 |
| I | -1.53780000 | -1.02051600 | -0.82798900 |
| C | 2.24490000 | -0.88259200 | -1.75666400 |
| C | 2.17290500 | -1.68922300 | -0.61165000 |
| C | 3.02320500 | -1.30195300 | -2.84790100 |
| C | 2.86529300 | -2.90079000 | -0.55788600 |
| H | 1.56421400 | -1.36838000 | 0.22875500 |
| C | 3.70454300 | -2.51880900 | -2.79644100 |
| H | 3.08976900 | -0.68643900 | -3.74089600 |
| C | 3.62724000 | -3.31853400 | -1.65183000 |
| H | 2.80247600 | -3.51883100 | 0.33361100 |
| H | 4.29705900 | -2.84137100 | -3.64839300 |
| H | 4.16025400 | -4.26482200 | -1.61396900 |
| C | 2.82720400 | 1.90142600 | -1.92388000 |
| H | 2.46800800 | 2.92885400 | -2.01405300 |
| H | 3.45830700 | 1.83463500 | -1.03348000 |
| H | 3.41217000 | 1.63694500 | -2.80995400 |
| C | -2.95066600 | 0.81062100 | 1.99636100 |
| C | -4.31644900 | 1.01067400 | 1.75986800 |
| C | -2.54009000 | -0.27427200 | 2.78799600 |
| C | -5.25943400 | 0.14033000 | 2.31480100 |
| H | -4.64904400 | 1.83976300 | 1.14302600 |
| C | -3.48318400 | -1.13481900 | 3.34827600 |
| H | -1.48089300 | -0.45454400 | 2.95614500 |
| C | -4.84662200 | -0.92997400 | 3.11073600 |
| H | -6.31695700 | 0.30210000 | 2.12302500 |
| H | -3.15507100 | -1.96990600 | 3.96148400 |
| H | -5.58165500 | -1.60475500 | 3.54132800 |
| C | -1.07891600 | 2.75963700 | 2.86319800 |
| H | -0.47654100 | 2.04690600 | 3.43454700 |

| | | | |
|---|---|---|---|
| H | -0.45721800 | 3.62292700 | 2.61751200 |
| H | -1.93080700 | 3.06926300 | 3.47524500 |

**3-I-PPh$_3$**

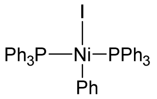


E = -2485.66788399

$G_{corr}$ = 0.564945

| | | | |
|---|---|---|---|
| Ni | -1.16813600 | 0.06610900 | 0.00127000 |
| P | 0.33072600 | -0.35533400 | -1.66331400 |
| C | -0.18342600 | -1.11965200 | -3.26507600 |
| C | -1.15088600 | -0.43098300 | -4.01739300 |
| H | -1.58399100 | 0.48721600 | -3.63162500 |
| C | -1.56026900 | -0.91475700 | -5.25804200 |
| H | -2.30787400 | -0.36970500 | -5.82798200 |
| C | -1.01632400 | -2.10096700 | -5.76221500 |
| H | -1.33917600 | -2.48173800 | -6.72752700 |
| C | -0.05459500 | -2.78936600 | -5.02213000 |
| H | 0.37922600 | -3.70747700 | -5.40962900 |
| C | 0.36583600 | -2.30055100 | -3.78079900 |
| H | 1.12644300 | -2.84267500 | -3.23008000 |
| C | 1.68749500 | -1.40972400 | -1.02902300 |
| C | 2.96838700 | -0.92242800 | -0.74061500 |
| H | 3.23212200 | 0.10220000 | -0.97736600 |
| C | 3.90584900 | -1.74443300 | -0.11055400 |
| H | 4.89354600 | -1.35246200 | 0.11663100 |
| C | 3.57548400 | -3.05638100 | 0.23437100 |
| H | 4.30765500 | -3.69239000 | 0.72461500 |
| C | 2.29514000 | -3.54527800 | -0.04149100 |
| H | 2.02589400 | -4.56150400 | 0.23363400 |
| C | 1.35175500 | -2.72375100 | -0.65761200 |
| H | 0.34893100 | -3.10112400 | -0.84149100 |
| P | -2.42605900 | 0.95622900 | 1.66722300 |
| C | -2.96952800 | -0.28390200 | 2.90345200 |
| C | -2.74442700 | -1.64295200 | 2.63930300 |
| H | -2.26819300 | -1.93495400 | 1.70968200 |
| C | -3.13215100 | -2.61556300 | 3.56324600 |
| H | -2.95211300 | -3.66493600 | 3.34639100 |
| C | -3.74524900 | -2.23870600 | 4.76016100 |
| H | -4.04849900 | -2.99538600 | 5.47878000 |
| C | -3.96093300 | -0.88527500 | 5.03756000 |
| H | -4.42855200 | -0.58685600 | 5.97189700 |
| C | -3.56938500 | 0.08906100 | 4.11899700 |
| H | -3.71916800 | 1.13826300 | 4.35521900 |
| C | -1.64899100 | 2.27388100 | 2.69854600 |
| C | -1.73667600 | 3.62429300 | 2.32978200 |
| H | -2.34210600 | 3.92371300 | 1.48006200 |
| C | -1.04944700 | 4.60123900 | 3.05469500 |
| H | -1.12951400 | 5.64382500 | 2.75819100 |
| C | -0.27075400 | 4.24213800 | 4.15644500 |
| H | 0.26367700 | 5.00304200 | 4.71898300 |
| C | -0.18233800 | 2.89866000 | 4.53187000 |
| H | 0.42558300 | 2.60746700 | 5.38411200 |
| C | -0.86349000 | 1.92082300 | 3.80836400 |
| H | -0.76625400 | 0.88042200 | 4.09876900 |

| | | | |
|---|---|---|---|
| C | -3.92362000 | 1.78267300 | 0.99108700 |
| C | -3.77366500 | 2.46539400 | -0.22773900 |
| C | -5.17343900 | 1.77347700 | 1.62295700 |
| C | -4.84918800 | 3.15012100 | -0.79234400 |
| H | -2.81599700 | 2.44115300 | -0.74207200 |
| C | -6.25400600 | 2.44672600 | 1.04674400 |
| H | -5.31733400 | 1.23176000 | 2.55163800 |
| C | -6.09378200 | 3.13985900 | -0.15534200 |
| H | -4.72018700 | 3.67645100 | -1.73425000 |
| H | -7.22250500 | 2.42597400 | 1.53919600 |
| H | -6.93684500 | 3.66233200 | -0.59932800 |
| C | 1.04647700 | 1.21400000 | -2.29543500 |
| C | 2.16829200 | 1.23232500 | -3.13932300 |
| C | 0.36811000 | 2.41199400 | -2.02887200 |
| C | 2.62306400 | 2.43764400 | -3.67502300 |
| H | 2.68190000 | 0.30650300 | -3.38243300 |
| C | 0.81705500 | 3.61581600 | -2.57587000 |
| H | -0.51053400 | 2.39731100 | -1.38834000 |
| C | 1.94924800 | 3.63047800 | -3.39345600 |
| H | 3.49750600 | 2.44444500 | -4.32012300 |
| H | 0.28398500 | 4.53830900 | -2.36180900 |
| H | 2.30330300 | 4.56684000 | -3.81621700 |
| C | 0.36021900 | 0.43346100 | 1.09332000 |
| C | 0.73319500 | -0.62022500 | 1.94787200 |
| H | 0.16774800 | -1.54868900 | 1.94035700 |
| C | 1.83427000 | -0.50620100 | 2.80156400 |
| H | 2.10458200 | -1.33950900 | 3.44659100 |
| C | 1.11981200 | 1.61117000 | 1.13441000 |
| H | 0.84936100 | 2.45645700 | 0.51090900 |
| C | 2.58850700 | 0.67122600 | 2.82065800 |
| H | 3.44665500 | 0.76252600 | 3.48199000 |
| C | 2.22573100 | 1.72909100 | 1.98388000 |
| H | 2.79703400 | 2.65493300 | 1.99616900 |
| I | -3.25565200 | -1.14711300 | -1.14193200 |

**3-Br-$PMe_3$**

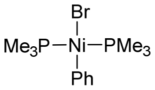


E = -1336.71315151

$G_{corr}$ = 0.270212

| | | | |
|---|---|---|---|
| Ni | 2.07944600 | 0.17680000 | -0.62344700 |
| P | 0.35120200 | 1.57309300 | -0.28472500 |
| C | -1.11884300 | 0.73094700 | 0.43233300 |
| C | -0.28499500 | 2.34146800 | -1.83066800 |
| C | 0.55356800 | 3.02613500 | 0.82478300 |
| H | -1.95671700 | 1.42892800 | 0.54074500 |
| H | -0.85674000 | 0.32924500 | 1.41723600 |
| H | -1.16282200 | 2.96420200 | -1.62415200 |
| H | -0.54822900 | 1.55271000 | -2.53996400 |
| H | 1.34103600 | 3.68097500 | 0.44121600 |
| H | 0.84768300 | 2.69261200 | 1.82399500 |
| P | 3.84578700 | -1.17240100 | -0.94103700 |
| C | 4.32071000 | -1.37831800 | -2.70653300 |
| C | 5.45458400 | -0.76998500 | -0.14695300 |
| C | 3.52929900 | -2.88862900 | -0.35829900 |
| H | 4.62482100 | -0.40769000 | -3.11310800 |
| H | 3.45613200 | -1.73659400 | -3.27085900 |
| H | 5.80498600 | 0.20738500 | -0.49079400 |
| H | 6.20038500 | -1.53251900 | -0.39722700 |
| H | 3.34465300 | -2.87380000 | 0.72141600 |
| H | 4.38509100 | -3.54018300 | -0.56874500 |
| H | -1.40832200 | -0.09996600 | -0.21630900 |
| H | 0.49952900 | 2.96285500 | -2.27605400 |
| H | -0.38549700 | 3.58691000 | 0.88956400 |
| H | 5.15157600 | -2.08575600 | -2.80724400 |
| H | 5.33142400 | -0.72330800 | 0.93854600 |
| H | 2.63742500 | -3.27504200 | -0.85893600 |
| C | 3.11041500 | 1.20800400 | 0.57785400 |
| C | 3.13036300 | 0.90450000 | 1.95084000 |
| C | 3.88880900 | 1.65948200 | 2.85351000 |
| C | 4.64930700 | 2.74197000 | 2.40037300 |
| C | 4.64267400 | 3.05876200 | 1.03839400 |
| C | 3.88237500 | 2.29912700 | 0.14173000 |
| H | 2.54431000 | 0.06544600 | 2.32320700 |
| H | 3.88543400 | 1.40210200 | 3.91107000 |
| H | 5.23915500 | 3.33032900 | 3.09899800 |
| H | 5.23104900 | 3.89843500 | 0.67302700 |
| H | 3.89164900 | 2.56112000 | -0.91520000 |
| Br | 0.70472600 | -1.19764500 | -2.14301000 |

**3-Cl-$PMe_3$**

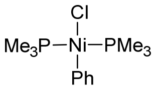


E = -1783.76597845

$G_{corr}$ = 0.270514

| | | | |
|---|---|---|---|
| Ni | 0.11907600 | 0.69356100 | 0.02506100 |
| C | 2.00844400 | 0.65361100 | -0.01394400 |
| C | 2.72715300 | 0.37054900 | -1.18893900 |
| C | 2.75051500 | 0.90674000 | 1.15459800 |
| C | 4.12679400 | 0.33042200 | -1.19756300 |
| H | 2.18664200 | 0.17396800 | -2.11328100 |
| C | 4.14887700 | 0.87220900 | 1.15340900 |
| H | 2.22720700 | 1.13148400 | 2.08271800 |
| C | 4.84505900 | 0.58137200 | -0.02470000 |
| H | 4.65628300 | 0.10573900 | -2.12164200 |
| H | 4.69623600 | 1.07031500 | 2.07315800 |
| H | 5.93196800 | 0.55208800 | -0.02812900 |
| P | 0.18217600 | 2.89527700 | -0.39461500 |
| P | 0.18101400 | -1.53705400 | 0.29706600 |
| C | -0.46549900 | 3.86221500 | 1.02900500 |
| H | -0.47734300 | 4.93623800 | 0.81132000 |
| H | -1.47940000 | 3.51916900 | 1.25336300 |
| H | 0.16586900 | 3.67923000 | 1.90513600 |
| C | 1.78219600 | 3.71981300 | -0.76708500 |

| | | | |
|---|---|---|---|
| H | 2.47367100 | 3.58424100 | 0.06913500 |
| H | 2.23331300 | 3.26887300 | -1.65596700 |
| H | 1.62694100 | 4.79004400 | -0.94338100 |
| C | -0.87574100 | 3.41302800 | -1.80640100 |
| H | -1.89675200 | 3.06545900 | -1.63091400 |
| H | -0.86711500 | 4.50230200 | -1.92561900 |
| H | -0.50337700 | 2.94840900 | -2.72589800 |
| C | 1.62394300 | -2.30126100 | 1.14465600 |
| H | 2.54576900 | -2.04227700 | 0.61825400 |
| H | 1.69338700 | -1.92361800 | 2.16984900 |
| H | 1.51331300 | -3.39110100 | 1.17131700 |
| C | 0.19754500 | -2.34026000 | -1.35903900 |
| H | 0.19609500 | -3.43256700 | -1.27018100 |
| H | -0.68529700 | -2.02049800 | -1.92203800 |
| H | 1.09286400 | -2.02432900 | -1.90333100 |
| C | -1.24283300 | -2.33409100 | 1.14452200 |
| H | -2.16881000 | -2.05824600 | 0.63505600 |
| H | -1.12880400 | -3.42401100 | 1.14777700 |
| H | -1.30037500 | -1.97320400 | 2.17670600 |
| Cl | -2.17902600 | 0.77818700 | 0.10558400 |

**4-I-PMe$_3$**

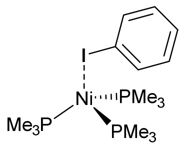

E = -1796.05427209

$G_{corr}$ = 0.373122

| | | | |
|---|---|---|---|
| Ni | -2.68722900 | 0.81089200 | -0.14483600 |
| P | -3.66742600 | 2.35298400 | -1.33936100 |
| C | -4.12724100 | 3.96460900 | -0.53499200 |
| C | -5.27247300 | 1.96983200 | -2.19612200 |
| C | -2.68564800 | 2.99467000 | -2.77863100 |
| H | -4.59588600 | 4.66484600 | -1.23905400 |
| H | -3.22643400 | 4.43177500 | -0.12107500 |
| H | -5.63388500 | 2.80809800 | -2.80629500 |
| H | -6.03329400 | 1.72259800 | -1.44666700 |
| H | -2.49795800 | 2.17853500 | -3.48385000 |
| H | -1.71667800 | 3.36283000 | -2.42427900 |
| P | -1.16623200 | 1.73310900 | 1.13600600 |
| P | -2.16836800 | -0.96629500 | -1.31013500 |
| C | -0.15786000 | 3.08249200 | 0.34966300 |
| C | 0.21609300 | 0.75998900 | 1.91728000 |
| C | -1.77927200 | 2.62888000 | 2.64378100 |
| C | -2.08702200 | -0.78017200 | -3.15849700 |
| C | -0.52550600 | -1.79587300 | -1.03129300 |
| C | -3.28329900 | -2.44705400 | -1.19171800 |
| H | 0.56071800 | 3.52279600 | 1.05330600 |
| H | -0.82054900 | 3.87287800 | -0.01911800 |
| H | 0.90206800 | 1.40457900 | 2.48294300 |
| H | 0.78301900 | 0.23440000 | 1.14048900 |
| H | -2.26883000 | 1.91306100 | 3.31371800 |
| H | -2.52689100 | 3.37087000 | 2.34221100 |
| H | -1.33754000 | -0.02307900 | -3.41440900 |
| H | -3.05842500 | -0.43812200 | -3.53216600 |
| H | 0.27656600 | -1.06442700 | -1.18173000 |
| H | -0.36514000 | -2.64298000 | -1.71165300 |
| H | -3.31824700 | -2.79186400 | -0.15241400 |
| H | -2.94596400 | -3.27342000 | -1.83071700 |
| H | -4.81923500 | 3.77538200 | 0.29348600 |
| H | -5.13810600 | 1.09232100 | -2.83907800 |
| H | -3.20690300 | 3.80573300 | -3.30358300 |
| H | -1.82479400 | -1.72166200 | -3.65894800 |
| H | -0.46399900 | -2.15854600 | 0.00041500 |
| H | -4.29954000 | -2.16130000 | -1.48505500 |
| H | -0.97077700 | 3.13259200 | 3.18940300 |
| H | 0.39084100 | 2.67317000 | -0.50596000 |
| H | -0.20104600 | 0.01164600 | 2.59919800 |
| I | -4.60759000 | 0.05979400 | 1.50943800 |
| C | -3.74504700 | -1.46738800 | 2.74648800 |
| C | -2.43050900 | -1.84774600 | 2.48064100 |
| C | -4.47725900 | -2.05610400 | 3.77933100 |
| C | -1.83481600 | -2.83877200 | 3.26661500 |
| H | -1.88769500 | -1.36727400 | 1.67052800 |
| C | -3.86942000 | -3.04600100 | 4.55892400 |
| H | -5.50138600 | -1.75347900 | 3.97802900 |
| C | -2.55145300 | -3.43845900 | 4.30574800 |
| H | -0.80943300 | -3.13812100 | 3.06324900 |
| H | -4.43136300 | -3.50910000 | 5.36603500 |
| H | -2.08648300 | -4.20792900 | 4.91599800 |

**4-I-PMe$_2$Ph**

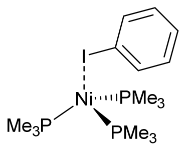

E = -2371.44101625

$G_{corr}$ = 0.520621

| | | | |
|---|---|---|---|
| Ni | -0.28787400 | 0.20661100 | 0.10664600 |
| P | 0.18039100 | -0.32849400 | -1.95012300 |
| P | -1.74657500 | -1.18795100 | 0.91742300 |
| P | 1.46724900 | 0.60923200 | 1.33973400 |
| C | 1.61298000 | 0.59628900 | -2.66746200 |
| C | 1.47843700 | 1.98279500 | -2.86850200 |
| H | 0.52004800 | 2.46489900 | -2.68310900 |
| C | 2.55999000 | 2.75773000 | -3.28437200 |
| H | 2.43089800 | 3.82639400 | -3.43845200 |
| C | 3.81111000 | 2.16521800 | -3.49236800 |
| H | 4.65804800 | 2.76987800 | -3.80596100 |
| C | 3.96111900 | 0.79188200 | -3.29249900 |
| H | 4.92758500 | 0.31986400 | -3.45297300 |
| C | 2.87073800 | 0.01314200 | -2.88869100 |
| H | 3.01475900 | -1.05299800 | -2.73942200 |
| C | -1.10912300 | -0.07017100 | -3.26413700 |
| H | -1.99249400 | -0.66237800 | -3.00145200 |
| H | -0.75710400 | -0.36467000 | -4.26014900 |

| | | | |
|---|---|---|---|
| H | -1.40721100 | 0.98290500 | -3.28583900 |
| C | 0.61424100 | -2.09892900 | -2.29105100 |
| H | -0.28291200 | -2.69809000 | -2.10241900 |
| H | 1.39836600 | -2.43854600 | -1.60728400 |
| H | 0.93577700 | -2.26519300 | -3.32624100 |
| C | -3.00827200 | -1.69701000 | -0.33282300 |
| C | -2.96309900 | -2.92414800 | -1.01450400 |
| H | -2.23113400 | -3.67770800 | -0.73846900 |
| C | -3.85221600 | -3.19710200 | -2.05973100 |
| H | -3.79710700 | -4.15286700 | -2.57558700 |
| C | -4.80864300 | -2.25238100 | -2.43683000 |
| H | -5.49953800 | -2.46517300 | -3.24840900 |
| C | -4.87090700 | -1.02923800 | -1.75853000 |
| H | -5.61410300 | -0.28721200 | -2.04068700 |
| C | -3.97706600 | -0.75346100 | -0.72453700 |
| H | -4.02516700 | 0.20937200 | -0.22172100 |
| C | -2.84710400 | -0.80550700 | 2.36607400 |
| H | -3.49937000 | 0.03885200 | 2.13020300 |
| H | -2.21855800 | -0.51622200 | 3.21427500 |
| H | -3.46533500 | -1.66717100 | 2.64662300 |
| C | -1.04631200 | -2.80893700 | 1.48643900 |
| H | -0.47370300 | -2.61701800 | 2.40044200 |
| H | -0.35382300 | -3.20563600 | 0.73755800 |
| H | -1.81890700 | -3.55575300 | 1.70644800 |
| C | 1.12589300 | 1.41583100 | 2.96775900 |
| C | 0.48538900 | 0.67063700 | 3.97603200 |
| H | 0.28811500 | -0.38751700 | 3.82115100 |
| C | 0.08073100 | 1.27022600 | 5.16821100 |
| H | -0.40898700 | 0.67290000 | 5.93354000 |
| C | 0.29374900 | 2.63825800 | 5.37586700 |
| H | -0.02987300 | 3.10943100 | 6.30019900 |
| C | 0.92366400 | 3.39189500 | 4.38345300 |
| H | 1.09363300 | 4.45545500 | 4.53249800 |
| C | 1.33880400 | 2.78651000 | 3.19214400 |
| H | 1.82243900 | 3.39574100 | 2.43437900 |
| C | 2.48281000 | -0.85742600 | 1.85992600 |
| H | 1.86678700 | -1.55922500 | 2.43073300 |
| H | 3.35425300 | -0.57241100 | 2.46171700 |
| H | 2.82229200 | -1.37101700 | 0.95331500 |
| C | 2.81483700 | 1.67729600 | 0.64188200 |
| H | 2.40139500 | 2.62710900 | 0.28881400 |
| H | 3.22727500 | 1.15696900 | -0.22871100 |
| H | 3.62138500 | 1.87582800 | 1.35837600 |
| I | -1.44110400 | 2.56136600 | -0.15989300 |
| C | -2.88546900 | 2.68983000 | 1.40893200 |
| C | -2.44280900 | 2.66714800 | 2.73407600 |
| C | -4.24858100 | 2.71056600 | 1.10017200 |
| C | -3.38648500 | 2.66309400 | 3.76427800 |
| H | -1.38431600 | 2.63012300 | 2.96203500 |
| C | -5.18139700 | 2.69849300 | 2.14307900 |
| H | -4.58209300 | 2.72224300 | 0.06702800 |
| C | -4.75368400 | 2.67389600 | 3.47322300 |
| H | -3.04317000 | 2.64091500 | 4.79518300 |
| H | -6.24290800 | 2.70607800 | 1.90916900 |
| H | -5.48305000 | 2.66175100 | 4.27856200 |

**4-I-PMePh$_2$**

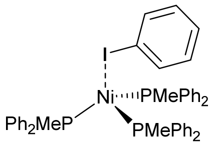


E = -2946.83103576

$G_{corr}$ = 0.672649

| | | | |
|---|---|---|---|
| Ni | -1.22910900 | 0.81013200 | -0.04172700 |
| P | -0.98543800 | 0.74676200 | -2.20350100 |
| C | -2.46391900 | 0.01537400 | -3.04233100 |
| C | -3.61960600 | 0.79480500 | -3.22201200 |
| H | -3.60552300 | 1.85136600 | -2.97239200 |
| C | -4.79905600 | 0.22532700 | -3.70660800 |
| H | -5.68150400 | 0.84645100 | -3.84011900 |
| C | -4.84660400 | -1.13715700 | -4.01751500 |
| H | -5.76548600 | -1.58200400 | -4.39025100 |
| C | -3.70187900 | -1.92070100 | -3.84828100 |
| H | -3.72587800 | -2.98049800 | -4.09003300 |
| C | -2.52141000 | -1.35023300 | -3.36710700 |
| H | -1.64344600 | -1.97464500 | -3.23504500 |
| C | 0.39138000 | -0.25575400 | -2.94565300 |
| C | 1.18449900 | -1.03439800 | -2.09175000 |
| H | 0.96426900 | -1.04090500 | -1.02982800 |
| C | 2.23077300 | -1.81398100 | -2.59398500 |
| H | 2.82480800 | -2.41859800 | -1.91409100 |
| C | 2.49685100 | -1.82319900 | -3.96430600 |
| H | 3.30987700 | -2.42678100 | -4.35989700 |
| C | 1.70702000 | -1.05770200 | -4.82960000 |
| H | 1.90518800 | -1.06707300 | -5.89864100 |
| C | 0.66061500 | -0.28426800 | -4.32503700 |
| H | 0.04632500 | 0.28864500 | -5.01445200 |
| C | -0.78944600 | 2.33820500 | -3.15305800 |
| H | -0.82484600 | 2.20361600 | -4.23914700 |
| H | 0.17414600 | 2.78176200 | -2.87966800 |
| H | -1.57822800 | 3.03544700 | -2.85646400 |
| P | -2.59696000 | 2.30255300 | 0.77589800 |
| C | -3.09004900 | 1.98731700 | 2.53046200 |
| C | -2.24945500 | 2.37455000 | 3.58974200 |
| H | -1.37184100 | 2.98029000 | 3.39117400 |
| C | -2.52910400 | 1.99133200 | 4.90165500 |
| H | -1.86381200 | 2.30540100 | 5.70186800 |
| C | -3.65046100 | 1.20396800 | 5.18342600 |
| H | -3.86481800 | 0.89995500 | 6.20478600 |
| C | -4.49255700 | 0.81211900 | 4.14008000 |
| H | -5.36581900 | 0.19719000 | 4.34320100 |
| C | -4.21670500 | 1.20110800 | 2.82645600 |
| H | -4.87655200 | 0.86660500 | 2.03243800 |
| C | -2.05284300 | 4.07554500 | 0.82333500 |
| C | -2.61771100 | 5.04128200 | 1.67162800 |
| H | -3.39580200 | 4.75717300 | 2.37494000 |
| C | -2.17460300 | 6.36504900 | 1.63519000 |
| H | -2.61238700 | 7.09952400 | 2.30677000 |

| | | | |
|---|---|---|---|
| C | -1.17321600 | 6.74740400 | 0.73623700 |
| H | -0.83063500 | 7.77862600 | 0.70852200 |
| C | -0.61460200 | 5.79768200 | -0.12315900 |
| H | 0.16733100 | 6.08516500 | -0.82180700 |
| C | -1.04623500 | 4.46932000 | -0.07001900 |
| H | -0.58746200 | 3.71734300 | -0.70439200 |
| C | -4.26025600 | 2.53190400 | -0.03134400 |
| H | -4.91882100 | 3.16858300 | 0.57025600 |
| H | -4.74084100 | 1.56432200 | -0.19812600 |
| H | -4.10098100 | 3.00756100 | -1.00373700 |
| P | -1.59155900 | -1.08339300 | 1.02844700 |
| C | -0.74825800 | -2.61659900 | 0.42818400 |
| C | 0.52126900 | -2.97649600 | 0.91420600 |
| H | 0.96757100 | -2.42630100 | 1.73814600 |
| C | 1.23219800 | -4.03503800 | 0.34129500 |
| H | 2.21227200 | -4.29783500 | 0.73239300 |
| C | 0.68893100 | -4.75432000 | -0.72624800 |
| H | 1.24368200 | -5.57492000 | -1.17338100 |
| C | -0.57602200 | -4.40998200 | -1.21250200 |
| H | -1.01228900 | -4.96539300 | -2.03936400 |
| C | -1.28515600 | -3.35212500 | -0.64263700 |
| H | -2.26299300 | -3.09261400 | -1.03765600 |
| C | -3.36699800 | -1.60737000 | 1.03797600 |
| C | -4.17455300 | -1.19359900 | -0.03314300 |
| H | -3.73459800 | -0.60023200 | -0.82723100 |
| C | -5.53278600 | -1.51760000 | -0.07462600 |
| H | -6.13660600 | -1.19138300 | -0.91749800 |
| C | -6.10821200 | -2.24791300 | 0.96849900 |
| H | -7.16748900 | -2.49134000 | 0.94720000 |
| C | -5.31408000 | -2.66464000 | 2.04152500 |
| H | -5.75564300 | -3.23295600 | 2.85655900 |
| C | -3.95232300 | -2.35382600 | 2.07271000 |
| H | -3.35499700 | -2.68736000 | 2.91593200 |
| C | -1.16001000 | -1.11230300 | 2.83297500 |
| H | -1.18345200 | -2.11477000 | 3.27272900 |
| H | -1.85851100 | -0.46227000 | 3.36640300 |
| H | -0.15758300 | -0.69365200 | 2.95449400 |
| I | 1.25347100 | 1.44898500 | 0.79240700 |
| C | 1.25550300 | 2.52196600 | 2.64009800 |
| C | 1.32151900 | 1.81616300 | 3.84508600 |
| C | 1.10414600 | 3.91211600 | 2.62940300 |
| C | 1.21945500 | 2.51433100 | 5.05198400 |
| H | 1.43824000 | 0.73751400 | 3.84889000 |
| C | 0.99805600 | 4.59680100 | 3.84374000 |
| H | 1.04129600 | 4.45465800 | 1.69375700 |
| C | 1.04986400 | 3.90167100 | 5.05486900 |
| H | 1.26484100 | 1.96641100 | 5.98954200 |
| H | 0.86355400 | 5.67516400 | 3.83365400 |
| H | 0.95955600 | 4.43733200 | 5.99579600 |

**4-I-PPh$_3$**

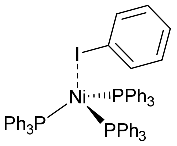


E = -3522.21388166

$G_{corr}$ = 0.823783

| | | | |
|---|---|---|---|
| Ni | 0.02935700 | 0.56132300 | -0.37060700 |
| P | 0.50429900 | 0.70327600 | -2.53514000 |
| C | -0.53935200 | -0.20767600 | -3.77843700 |
| C | -1.93168500 | -0.21016300 | -3.59155400 |
| H | -2.35782600 | 0.29819200 | -2.73554300 |
| C | -2.77614300 | -0.85984800 | -4.49292800 |
| H | -3.85000400 | -0.84005600 | -4.32754200 |
| C | -2.24089600 | -1.53054800 | -5.59620900 |
| H | -2.89526700 | -2.04383500 | -6.29610500 |
| C | -0.85786500 | -1.53513800 | -5.79396200 |
| H | -0.43120100 | -2.04987000 | -6.65135800 |
| C | -0.01361900 | -0.87353100 | -4.89733500 |
| H | 1.05651100 | -0.87856300 | -5.07651700 |
| C | 2.19871000 | 0.20660400 | -3.10240100 |
| C | 2.65172900 | -1.06901700 | -2.72932400 |
| H | 1.99749500 | -1.72251900 | -2.16197600 |
| C | 3.93652100 | -1.49710600 | -3.06047200 |
| H | 4.27089400 | -2.48271400 | -2.74884600 |
| C | 4.79657100 | -0.64802500 | -3.76471600 |
| H | 5.80447100 | -0.97259100 | -4.01024900 |
| C | 4.35432400 | 0.61974700 | -4.14918200 |
| H | 5.01490900 | 1.28376200 | -4.70130400 |
| C | 3.06068400 | 1.04288900 | -3.82782800 |
| H | 2.73146200 | 2.03160800 | -4.13216400 |
| P | -1.58635600 | 1.99558400 | 0.19124100 |
| C | -2.59999600 | 1.62418800 | 1.71017600 |
| C | -3.69269300 | 2.40950900 | 2.11564600 |
| H | -3.97404400 | 3.28858800 | 1.54402500 |
| C | -4.43324500 | 2.06332500 | 3.24586600 |
| H | -5.27595100 | 2.68017500 | 3.54845200 |
| C | -4.09804900 | 0.92223900 | 3.98562800 |
| H | -4.68060100 | 0.65250500 | 4.86304100 |
| C | -3.01475400 | 0.13577700 | 3.59201700 |
| H | -2.73922500 | -0.75188300 | 4.15511600 |
| C | -2.27240800 | 0.49230700 | 2.46240900 |
| H | -1.42163200 | -0.10353900 | 2.16075700 |
| C | -1.13005700 | 3.77149600 | 0.49944100 |
| C | -2.00247700 | 4.85813100 | 0.32263100 |
| H | -3.00952400 | 4.69557300 | -0.04786300 |
| C | -1.57943400 | 6.16159400 | 0.59264800 |
| H | -2.26649200 | 6.99064100 | 0.44225700 |
| C | -0.28037500 | 6.40248900 | 1.05032500 |
| H | 0.04630100 | 7.41875200 | 1.25544500 |
| C | 0.59524700 | 5.32959900 | 1.23452200 |
| H | 1.61273400 | 5.49790900 | 1.57800600 |
| C | 0.17002900 | 4.02918700 | 0.95706700 |

| | | | |
|---|---|---|---|
| H | 0.86339800 | 3.20528900 | 1.06900300 |
| P | 1.77700200 | 0.31179100 | 0.95767400 |
| C | 1.51056500 | 0.15083100 | 2.79403800 |
| C | 0.66707200 | 1.08383800 | 3.41996000 |
| H | 0.21352600 | 1.87433100 | 2.83176200 |
| C | 0.38975800 | 1.00257100 | 4.78449200 |
| H | -0.27007900 | 1.73480700 | 5.24231800 |
| C | 0.93698100 | -0.03054900 | 5.55046300 |
| H | 0.70968900 | -0.10565900 | 6.61066000 |
| C | 1.77751600 | -0.96580200 | 4.94222400 |
| H | 2.21428100 | -1.77040900 | 5.52875600 |
| C | 2.07033600 | -0.87089200 | 3.57812800 |
| H | 2.73128900 | -1.60449100 | 3.12865100 |
| C | 2.96404500 | 1.74367800 | 0.88744700 |
| C | 3.32148700 | 2.53237900 | 1.99175800 |
| H | 2.94763300 | 2.29327900 | 2.98144800 |
| C | 4.15602100 | 3.64428300 | 1.83305500 |
| H | 4.41446700 | 4.24591800 | 2.70104700 |
| C | 4.65989800 | 3.97714200 | 0.57406500 |
| H | 5.30661900 | 4.84230700 | 0.45371000 |
| C | 4.32910700 | 3.18367900 | -0.52885400 |
| H | 4.71806500 | 3.42206500 | -1.51566200 |
| C | 3.48736500 | 2.08380100 | -0.37194400 |
| H | 3.23976100 | 1.48044300 | -1.23634000 |
| C | 2.93420700 | -1.11610100 | 0.66893100 |
| C | 2.37878400 | -2.36632400 | 0.35380400 |
| C | 4.32959100 | -1.01293700 | 0.77656000 |
| C | 3.19076000 | -3.48361300 | 0.15265100 |
| H | 1.30404700 | -2.46202600 | 0.24452000 |
| C | 5.14582200 | -2.12630700 | 0.55982600 |
| H | 4.78801000 | -0.06181700 | 1.02798700 |
| C | 4.58034800 | -3.36561200 | 0.24971500 |
| H | 2.73781200 | -4.44137800 | -0.09116500 |
| H | 6.22528300 | -2.02383900 | 0.63825500 |
| H | 5.21629300 | -4.23119800 | 0.08307000 |
| C | -2.94579300 | 2.14193800 | -1.05880600 |
| C | -2.82373300 | 2.99324800 | -2.17149200 |
| C | -4.03860100 | 1.25676600 | -1.01330400 |
| C | -3.76586200 | 2.96410000 | -3.20067300 |
| H | -1.98823700 | 3.68112500 | -2.24038000 |
| C | -4.97914500 | 1.22679100 | -2.04587600 |
| H | -4.15336700 | 0.58022700 | -0.17200600 |
| C | -4.84676400 | 2.08006700 | -3.14488100 |
| H | -3.64721900 | 3.63122300 | -4.05057300 |
| H | -5.81545500 | 0.53428600 | -1.98929700 |
| H | -5.57729300 | 2.05528600 | -3.94920900 |
| C | 0.38895900 | 2.44260700 | -3.16102900 |
| C | 0.82892200 | 3.46096200 | -2.30126500 |
| C | -0.15086000 | 2.79597500 | -4.40635700 |
| C | 0.71984100 | 4.80297500 | -2.66546900 |
| H | 1.23260100 | 3.19608000 | -1.33119800 |
| C | -0.26819200 | 4.14054300 | -4.77021000 |
| H | -0.49706300 | 2.02653900 | -5.08940600 |
| C | 0.16115700 | 5.14699300 | -3.90041100 |
| H | 1.04968300 | 5.57485500 | -1.97511400 |
| H | -0.70102400 | 4.40075300 | -5.73305300 |
| H | 0.05879400 | 6.19190700 | -4.18197000 |
| I | -1.45173800 | -1.75913200 | -0.47396100 |
| C | -1.80793800 | -2.74648800 | 1.38587500 |
| C | -3.02818200 | -3.39465700 | 1.59685800 |
| C | -0.84161700 | -2.68650400 | 2.39243300 |
| C | -3.27630500 | -3.99091900 | 2.83716800 |
| H | -3.77974900 | -3.42873500 | 0.81380700 |
| C | -1.10528900 | -3.28054100 | 3.62979500 |
| H | 0.09408800 | -2.16544500 | 2.23222700 |
| C | -2.32021100 | -3.93330800 | 3.85510900 |
| H | -4.22449500 | -4.49503900 | 3.00539900 |
| H | -0.35713000 | -3.21825600 | 4.41555000 |
| H | -2.52264000 | -4.39247800 | 4.81886600 |

**4-Br-PMe$_3$**

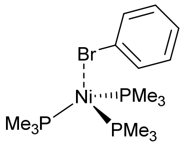


E = -1797.83621232

$G_{corr}$ = 0.373538

| | | | |
|---|---|---|---|
| Ni | 2.61103600 | 7.78219200 | 11.76114000 |
| P | 1.48320500 | 9.29356400 | 10.67376100 |
| C | 1.04476600 | 10.88616800 | 11.53358300 |
| C | -0.17310000 | 8.90367600 | 9.92072400 |
| C | 2.35001800 | 9.98124900 | 9.18057700 |
| H | 0.53744900 | 11.59665800 | 10.86742900 |
| H | 1.95751700 | 11.35489500 | 11.91808400 |
| H | -0.59229100 | 9.75315700 | 9.36510200 |
| H | -0.87690800 | 8.61839000 | 10.71145800 |
| H | 2.52272700 | 9.17751700 | 8.45700800 |
| H | 3.32577300 | 10.37950200 | 9.48024300 |
| P | 4.07279100 | 8.75247700 | 13.05470700 |
| P | 3.14001000 | 6.01293600 | 10.61320100 |
| C | 5.03007700 | 10.13205700 | 12.25640800 |
| C | 5.49159000 | 7.84249800 | 13.85119400 |
| C | 3.41930000 | 9.63857100 | 14.55162000 |
| C | 3.22336100 | 6.18681700 | 8.76240800 |
| C | 4.78883800 | 5.19064500 | 10.89178700 |
| C | 2.03526500 | 4.52359700 | 10.73607800 |
| H | 5.73103400 | 10.60910000 | 12.95390200 |
| H | 4.33601000 | 10.88988100 | 11.87719500 |
| H | 6.16212800 | 8.51879300 | 14.39820600 |
| H | 6.06852900 | 7.31664700 | 13.08201000 |
| H | 2.94351500 | 8.91342300 | 15.22134700 |
| H | 2.65300800 | 10.35484500 | 14.23632200 |
| H | 3.94726900 | 6.96783700 | 8.50407600 |
| H | 2.24290900 | 6.49184000 | 8.38055300 |
| H | 5.58779700 | 5.92142100 | 10.72188300 |
| H | 4.94673300 | 4.33248700 | 10.22454000 |
| H | 2.00854200 | 4.17335600 | 11.77368500 |
| H | 2.37444500 | 3.70160900 | 10.09218000 |

| | | | |
|---|---|---|---|
| H | 0.39063600 | 10.67328000 | 12.38705700 |
| H | -0.07021500 | 8.05016400 | 9.24130100 |
| H | 1.76915800 | 10.77816700 | 8.69836200 |
| H | 3.52067800 | 5.25109200 | 8.27049300 |
| H | 4.86066600 | 4.84527900 | 11.92885700 |
| H | 1.01547500 | 4.80380500 | 10.45015900 |
| H | 4.20618100 | 10.16954000 | 15.10315200 |
| H | 5.59316500 | 9.73434600 | 11.40472600 |
| H | 5.10300000 | 7.09690700 | 14.55306600 |
| Br | 0.78679500 | 6.93691200 | 13.42165500 |
| C | 1.54549600 | 5.54112800 | 14.54133900 |
| C | 2.88301100 | 5.21043900 | 14.34091400 |
| C | 0.76483800 | 4.90026100 | 15.50304200 |
| C | 3.45709900 | 4.20897300 | 15.12831300 |
| H | 3.44872100 | 5.73774400 | 13.57797200 |
| C | 1.35345300 | 3.89972500 | 16.28322600 |
| H | -0.27741900 | 5.16971800 | 15.64471900 |
| C | 2.69538000 | 3.55277200 | 16.09917700 |
| H | 4.50105000 | 3.94497200 | 14.97879400 |
| H | 0.75658000 | 3.39264500 | 17.03666600 |
| H | 3.14419900 | 2.77438900 | 16.70997000 |

**4-Cl-PMe$_3$**

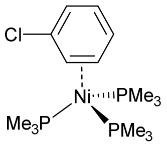


E = -2244.8916434

$G_{corr}$ = 0.374356

| | | | |
|---|---|---|---|
| Ni | 2.96763800 | 1.19341500 | -0.21067500 |
| P | 1.42451200 | 2.48576500 | -1.01080600 |
| C | 0.61658500 | 3.83252300 | -0.01261100 |
| C | -0.08931100 | 1.74478400 | -1.79610600 |
| C | 2.06728700 | 3.50082600 | -2.42905000 |
| H | -0.06989300 | 4.44155200 | -0.61589300 |
| H | 1.39024500 | 4.48423300 | 0.40926700 |
| H | -0.75313000 | 2.50747900 | -2.22445900 |
| H | -0.64922800 | 1.17437300 | -1.04681500 |
| H | 2.48475700 | 2.83889600 | -3.19503800 |
| H | 2.87412600 | 4.14927200 | -2.07020400 |
| P | 4.17358500 | 1.96282900 | 1.41499500 |
| P | 3.38857800 | -0.52025200 | -1.46374200 |
| C | 5.06139000 | 3.55136100 | 1.03556400 |
| C | 5.57838200 | 0.99280200 | 2.15839500 |
| C | 3.28210000 | 2.43981400 | 2.97221000 |
| C | 3.67382100 | -0.12280300 | -3.25687100 |
| C | 4.82943300 | -1.66474500 | -1.17488700 |
| C | 2.03958200 | -1.78884300 | -1.63713600 |
| H | 5.66345000 | 3.90083400 | 1.88423000 |
| H | 4.32835100 | 4.32330900 | 0.77664500 |
| H | 6.07691900 | 1.52891100 | 2.97689800 |
| H | 6.31561000 | 0.76872800 | 1.37865900 |
| H | 2.86132300 | 1.54411300 | 3.43911100 |
| H | 2.45402400 | 3.11187300 | 2.72367200 |
| H | 4.50796300 | 0.58145600 | -3.35034000 |
| H | 2.77796200 | 0.35372100 | -3.66926400 |
| H | 5.76047800 | -1.08677100 | -1.20425100 |
| H | 4.88281100 | -2.46816500 | -1.92134800 |
| H | 1.84976700 | -2.26542900 | -0.66925100 |
| H | 2.30257100 | -2.56801300 | -2.36409100 |
| H | 0.05845900 | 3.38731200 | 0.81762700 |
| H | 0.21670600 | 1.05495000 | -2.59061700 |
| H | 1.28270200 | 4.12317700 | -2.87819900 |
| H | 3.89982600 | -1.02128200 | -3.84548600 |
| H | 4.74629200 | -2.11182400 | -0.17735200 |
| H | 1.11713900 | -1.29443500 | -1.96033000 |
| H | 3.94746900 | 2.93379800 | 3.69217000 |
| H | 5.71772900 | 3.40626400 | 0.17018100 |
| H | 5.19280700 | 0.04063300 | 2.54049000 |
| C | 0.27772800 | 0.11414900 | 2.65444300 |
| C | 1.12778100 | -0.45978700 | 1.71067700 |
| C | 0.49229800 | -0.02861200 | 4.02533200 |
| C | 2.22525300 | -1.20031700 | 2.16197600 |
| H | 0.94328500 | -0.33277500 | 0.64952100 |
| C | 1.59497800 | -0.77038200 | 4.45747900 |
| H | -0.18581200 | 0.42879700 | 4.73825800 |
| C | 2.46123300 | -1.35690500 | 3.52964800 |
| H | 2.89605900 | -1.64127200 | 1.43265600 |
| H | 1.77273300 | -0.88744000 | 5.52293500 |
| H | 3.31695800 | -1.93198300 | 3.87178500 |
| Cl | -1.12298600 | 1.03953700 | 2.09739300 |

**TS-4-5-I-PMe$_3$**

E (OSS) = -1796.04522643

E (T) = -1796.02982302

$S^2$(OSS) = 0.5095

$G_{corr}$ = 0.371742

ν = -270.05 $cm^{-1}$

| | | | |
|---|---|---|---|
| Ni | -2.77813800 | 0.80421700 | -0.02224200 |
| P | -3.57721000 | 2.39008800 | -1.38115000 |
| C | -3.99742900 | 4.01400400 | -0.60033400 |
| C | -5.15366100 | 2.00546100 | -2.26987200 |
| C | -2.50314000 | 2.94822300 | -2.78148200 |
| H | -4.43538900 | 4.71222600 | -1.32468500 |
| H | -3.09097100 | 4.46403300 | -0.18160900 |
| H | -5.45994800 | 2.82258900 | -2.93510800 |
| H | -5.94413500 | 1.82251000 | -1.53430800 |
| H | -2.33323500 | 2.11347300 | -3.46787900 |
| H | -1.53204900 | 3.27180900 | -2.39245000 |
| P | -1.23823500 | 1.85676400 | 1.23939000 |
| P | -2.29799600 | -1.00269400 | -1.27498800 |
| C | -0.08894000 | 3.00078500 | 0.34243500 |
| C | -0.01836000 | 0.89022300 | 2.24729000 |

| | | | |
|---|---|---|---|
| C | -1.91564400 | 2.98860800 | 2.53835100 |
| C | -2.27862900 | -0.79961800 | -3.11662200 |
| C | -0.66522600 | -1.85110300 | -1.03576200 |
| C | -3.45658000 | -2.43370100 | -1.09571500 |
| H | 0.62494300 | 3.47568900 | 1.02696100 |
| H | -0.66179500 | 3.78404000 | -0.16544300 |
| H | 0.69312600 | 1.54968000 | 2.76036000 |
| H | 0.53632100 | 0.20553300 | 1.59624300 |
| H | -2.52675700 | 2.40545000 | 3.23548600 |
| H | -2.56546400 | 3.73232300 | 2.06560700 |
| H | -1.48434700 | -0.10029100 | -3.39835900 |
| H | -3.23581800 | -0.38839200 | -3.45414900 |
| H | 0.14566800 | -1.13011300 | -1.18887300 |
| H | -0.53339600 | -2.68860600 | -1.73293600 |
| H | -3.46471100 | -2.76340900 | -0.05171200 |
| H | -3.17153000 | -3.27582400 | -1.73866400 |
| H | -4.70685500 | 3.84997200 | 0.21723700 |
| H | -5.02475500 | 1.09176400 | -2.86038000 |
| H | -2.96088400 | 3.77677700 | -3.33580200 |
| H | -2.10501400 | -1.75569100 | -3.62601200 |
| H | -0.59466600 | -2.23262800 | -0.01216500 |
| H | -4.47091900 | -2.11143600 | -1.35410500 |
| H | -1.12299400 | 3.50235500 | 3.09668800 |
| H | 0.46634000 | 2.43755200 | -0.41580700 |
| H | -0.55110400 | 0.29366600 | 2.99452000 |
| I | -4.66198400 | 0.10816400 | 1.61614300 |
| C | -3.62568100 | -1.68183400 | 2.85939700 |
| C | -2.31065100 | -1.92961300 | 2.50047200 |
| C | -4.27305700 | -2.41823600 | 3.84406600 |
| C | -1.60563700 | -2.95235600 | 3.14808300 |
| H | -1.84267400 | -1.32410300 | 1.72179800 |
| C | -3.56458500 | -3.44244100 | 4.49029300 |
| H | -5.30708100 | -2.21152000 | 4.11400400 |
| C | -2.23529900 | -3.70814600 | 4.14309300 |
| H | -0.57091400 | -3.15713700 | 2.87905200 |
| H | -4.05209700 | -4.03127400 | 5.26479300 |
| H | -1.69165900 | -4.50262000 | 4.64814100 |

**TS-4-5-I-PMe$_2$Ph**

E (OSS) = -2371.43028050

E (T) = -2371.41605770

$S^2$(OSS) = 0.5479

$G_{corr}$ = 0.519749

ν = -276.90 $cm^{-1}$

| | | | |
|---|---|---|---|
| Ni | -0.34083600 | 0.44923300 | 0.01234500 |
| P | 0.04180000 | -0.14834500 | -2.09720300 |
| P | -1.65254700 | -1.15548400 | 0.87201600 |
| P | 1.49086700 | 0.73557400 | 1.28600800 |
| C | 1.63446600 | 0.56804900 | -2.68673000 |
| C | 1.71899800 | 1.96512500 | -2.82581100 |
| H | 0.83959500 | 2.57708400 | -2.63917300 |
| C | 2.92418800 | 2.57764300 | -3.16521600 |
| H | 2.96855600 | 3.65870500 | -3.27125600 |
| C | 4.07651300 | 1.80578500 | -3.35211200 |
| H | 5.01953100 | 2.28344800 | -3.60462800 |
| C | 4.00723200 | 0.41876600 | -3.20805900 |
| H | 4.89699700 | -0.18917600 | -3.35284500 |
| C | 2.79376100 | -0.19783300 | -2.88301000 |
| H | 2.76314800 | -1.27867200 | -2.78019400 |
| C | -1.13608100 | 0.37873000 | -3.42406300 |
| H | -2.10967800 | -0.08174100 | -3.22527700 |
| H | -0.78970600 | 0.08226300 | -4.42096500 |
| H | -1.26287600 | 1.46488700 | -3.39174200 |
| C | 0.19298300 | -1.95197600 | -2.46805600 |
| H | -0.78881200 | -2.40778800 | -2.30127800 |
| H | 0.90570600 | -2.42060200 | -1.78232000 |
| H | 0.50180200 | -2.14282000 | -3.50221400 |
| C | -2.93155400 | -1.72693300 | -0.32548100 |
| C | -3.12564100 | -3.06345900 | -0.70374700 |
| H | -2.53585600 | -3.85551200 | -0.25205700 |
| C | -4.07750100 | -3.39532300 | -1.67461500 |
| H | -4.21161800 | -4.43527800 | -1.96198200 |
| C | -4.85374200 | -2.39915500 | -2.26950400 |
| H | -5.59344500 | -2.65934900 | -3.02206200 |
| C | -4.67139700 | -1.06290300 | -1.89469000 |
| H | -5.26834200 | -0.28005400 | -2.35578800 |
| C | -3.71058900 | -0.72958800 | -0.94064600 |
| H | -3.54314200 | 0.31287100 | -0.67970400 |
| C | -2.70025400 | -0.68253200 | 2.32275600 |
| H | -3.39975500 | 0.10166800 | 2.02409600 |
| H | -2.05839300 | -0.28326300 | 3.11300700 |
| H | -3.26388100 | -1.54298700 | 2.70225700 |
| C | -0.87203400 | -2.71058300 | 1.48975300 |
| H | -0.22346500 | -2.44385400 | 2.33086900 |
| H | -0.25010500 | -3.16342000 | 0.71107900 |
| H | -1.61026100 | -3.44072400 | 1.84058600 |
| C | 1.11372000 | 1.44228000 | 2.94453100 |
| C | 0.53631600 | 0.61846200 | 3.92818900 |
| H | 0.40522500 | -0.44337600 | 3.73634900 |
| C | 0.11582800 | 1.14466200 | 5.14967100 |
| H | -0.32522300 | 0.48829200 | 5.89577000 |
| C | 0.25727100 | 2.51138300 | 5.41235600 |
| H | -0.07455400 | 2.92376700 | 6.36148800 |
| C | 0.82641000 | 3.34120800 | 4.44380000 |
| H | 0.94099900 | 4.40488700 | 4.63696800 |
| C | 1.25049800 | 2.81289800 | 3.22032500 |
| H | 1.68555700 | 3.47993300 | 2.48255200 |
| C | 2.50053500 | -0.76197700 | 1.70263900 |
| H | 1.88374500 | -1.51390100 | 2.20244000 |
| H | 3.35860300 | -0.51858300 | 2.33993300 |
| H | 2.86142000 | -1.19586000 | 0.76296700 |
| C | 2.81951200 | 1.84452200 | 0.63293900 |
| H | 2.39899400 | 2.81219400 | 0.34492300 |
| H | 3.22601500 | 1.37704100 | -0.26962700 |
| H | 3.62897800 | 1.99818000 | 1.35641100 |
| I | -1.41107600 | 2.77213000 | -0.03817500 |
| C | -3.14736900 | 2.83441200 | 1.65270000 |
| C | -2.73549400 | 2.83192300 | 2.98302400 |

| | | | |
|---|---|---|---|
| C | -4.47000300 | 2.58088400 | 1.29879100 |
| C | -3.67916900 | 2.57981000 | 3.98680900 |
| H | -1.69349800 | 3.00022500 | 3.24416500 |
| C | -5.40920000 | 2.32666900 | 2.30906200 |
| H | -4.77373500 | 2.56384700 | 0.25395200 |
| C | -5.01291900 | 2.32344900 | 3.65047000 |
| H | -3.36740300 | 2.57338800 | 5.02938900 |
| H | -6.44596700 | 2.12573300 | 2.04633000 |
| H | -5.74104200 | 2.11813200 | 4.43117300 |

**TS-4-5-I-PMePh$_2$**

E (OSS) = -2946.81831720

E (T) = -2946.80534049

$S^2$(OSS) = 0.5464

$G_{corr}$ = 0.670258

ν = -273.91 cm$^{-1}$

| | | | |
|---|---|---|---|
| Ni | -1.08585700 | 0.77954500 | -0.00087100 |
| P | -0.89894500 | 0.78396000 | -2.22754300 |
| C | -2.43407500 | 0.14098400 | -3.02770700 |
| C | -3.53982500 | 0.98642500 | -3.21420900 |
| H | -3.45687800 | 2.04703700 | -2.99927300 |
| C | -4.75937800 | 0.47823700 | -3.66745400 |
| H | -5.60297700 | 1.14969800 | -3.80619300 |
| C | -4.89487400 | -0.88585800 | -3.94075100 |
| H | -5.84457600 | -1.28213400 | -4.29003300 |
| C | -3.79847700 | -1.73504700 | -3.76570600 |
| H | -3.89127800 | -2.79687300 | -3.97921000 |
| C | -2.57902400 | -1.22717600 | -3.31416300 |
| H | -1.73932400 | -1.90094300 | -3.17644900 |
| C | 0.40267400 | -0.29059800 | -2.97945700 |
| C | 1.20097500 | -1.08318600 | -2.14430900 |
| H | 1.04585900 | -1.04417700 | -1.07155400 |
| C | 2.17970300 | -1.92406800 | -2.68236000 |
| H | 2.78199500 | -2.53818800 | -2.01901700 |
| C | 2.36918800 | -1.98016700 | -4.06411900 |
| H | 3.12973400 | -2.63264000 | -4.48548200 |
| C | 1.57186100 | -1.19868600 | -4.90807100 |
| H | 1.71150600 | -1.24369100 | -5.98525100 |
| C | 0.59264900 | -0.36314700 | -4.37036900 |
| H | -0.03313400 | 0.22202600 | -5.03910800 |
| C | -0.62840100 | 2.38742000 | -3.11998100 |
| H | -0.67104700 | 2.27944600 | -4.20838800 |
| H | 0.35653100 | 2.77114000 | -2.83443300 |
| H | -1.38128500 | 3.11459700 | -2.80242500 |
| P | -2.67353700 | 2.23587300 | 0.72406700 |
| C | -3.11322700 | 1.88510200 | 2.47752300 |
| C | -2.21973400 | 2.24641600 | 3.50106100 |
| H | -1.32534500 | 2.81299500 | 3.26524500 |
| C | -2.47052200 | 1.87956200 | 4.82320400 |
| H | -1.76781200 | 2.17209100 | 5.59838500 |
| C | -3.60967100 | 1.13367300 | 5.14391600 |
| H | -3.80105500 | 0.84261000 | 6.17352300 |
| C | -4.49879500 | 0.76362400 | 4.13225400 |
| H | -5.38415500 | 0.17874700 | 4.36796000 |
| C | -4.25479800 | 1.13757100 | 2.80788100 |
| H | -4.95185500 | 0.82572200 | 2.03685600 |
| C | -2.09985600 | 3.98965800 | 0.77852800 |
| C | -2.72667300 | 4.96273000 | 1.57352100 |
| H | -3.55357600 | 4.68485200 | 2.22156000 |
| C | -2.28185000 | 6.28538300 | 1.55264100 |
| H | -2.76711900 | 7.02814000 | 2.18068800 |
| C | -1.21577900 | 6.65540100 | 0.72538800 |
| H | -0.87236000 | 7.68647600 | 0.70919500 |
| C | -0.59072900 | 5.69514400 | -0.07349400 |
| H | 0.24556000 | 5.97250600 | -0.71010100 |
| C | -1.02456000 | 4.36687700 | -0.03739600 |
| H | -0.51328500 | 3.60838200 | -0.62044900 |
| C | -4.33449600 | 2.45143700 | -0.07246600 |
| H | -4.98680200 | 3.08116600 | 0.54205500 |
| H | -4.81192500 | 1.48232600 | -0.23595800 |
| H | -4.18997400 | 2.93453200 | -1.04267800 |
| P | -1.55764100 | -1.15549600 | 1.09171900 |
| C | -0.70577800 | -2.65071600 | 0.43225700 |
| C | 0.57095100 | -3.00738400 | 0.89911800 |
| H | 1.02328200 | -2.46501900 | 1.72464200 |
| C | 1.27912000 | -4.05282300 | 0.30073200 |
| H | 2.26491500 | -4.31687800 | 0.67556600 |
| C | 0.72699500 | -4.75528700 | -0.77374800 |
| H | 1.28120000 | -5.56490700 | -1.24096400 |
| C | -0.54308900 | -4.40877500 | -1.24305800 |
| H | -0.98377400 | -4.95023000 | -2.07653300 |
| C | -1.25248000 | -3.36565400 | -0.64643700 |
| H | -2.23593300 | -3.10396000 | -1.02536000 |
| C | -3.33459400 | -1.64616500 | 1.06729500 |
| C | -4.11956000 | -1.20700300 | -0.01002400 |
| H | -3.66062700 | -0.61475600 | -0.79432500 |
| C | -5.48230300 | -1.50852900 | -0.07148100 |
| H | -6.07039900 | -1.16386500 | -0.91781800 |
| C | -6.08132900 | -2.24070700 | 0.95675700 |
| H | -7.14391000 | -2.46665300 | 0.91975300 |
| C | -5.30806400 | -2.68306600 | 2.03501400 |
| H | -5.76913700 | -3.25350300 | 2.83740500 |
| C | -3.94254600 | -2.39482200 | 2.08716900 |
| H | -3.35921000 | -2.74596400 | 2.93303600 |
| C | -1.12660600 | -1.19721700 | 2.88892000 |
| H | -1.12375800 | -2.20798000 | 3.30885500 |
| H | -1.84582000 | -0.57654500 | 3.42959900 |
| H | -0.13714400 | -0.74818100 | 3.01481300 |
| I | 1.24983500 | 1.32001300 | 0.98080100 |
| C | 1.34745100 | 2.74765900 | 2.96125300 |
| C | 1.34790700 | 2.13156500 | 4.21011500 |
| C | 1.11494500 | 4.11433700 | 2.82747100 |
| C | 1.12129100 | 2.90714900 | 5.35552100 |
| H | 1.50390100 | 1.05837800 | 4.30013000 |
| C | 0.87746600 | 4.88298400 | 3.97525900 |
| H | 1.09039900 | 4.58077500 | 1.84725700 |
| C | 0.87918400 | 4.28010800 | 5.23733800 |
| H | 1.12352900 | 2.43644100 | 6.33667900 |

| | | | |
|---|---|---|---|
| H | 0.68245100 | 5.94900000 | 3.87800100 |
| H | 0.68797000 | 4.87649300 | 6.12584200 |

**TS-4-5-I-$PPh_3$**

E (OSS) = -3522.19654393

E (T) = -3522.18625046

$S^2$(OSS) = 0.5756

$G_{corr}$ = 0.820685

ν = -295.63 $cm^{-1}$

| | | | |
|---|---|---|---|
| Ni | -0.29723200 | 0.75225900 | 0.25707900 |
| P | 0.00769200 | 0.44789300 | -2.00073400 |
| C | -1.30551500 | -0.54709000 | -2.83998100 |
| C | -2.61550100 | -0.48756900 | -2.34282700 |
| H | -2.82791500 | 0.10892000 | -1.46568600 |
| C | -3.64135500 | -1.21066400 | -2.95462400 |
| H | -4.65025000 | -1.14914800 | -2.55636500 |
| C | -3.36631900 | -2.01790400 | -4.06175700 |
| H | -4.16195900 | -2.59031700 | -4.53148400 |
| C | -2.06142400 | -2.09271200 | -4.55830800 |
| H | -1.83831600 | -2.72258900 | -5.41565400 |
| C | -1.03750900 | -1.36056000 | -3.95321200 |
| H | -0.02759500 | -1.42999800 | -4.34550700 |
| C | 1.51585600 | -0.52096200 | -2.43602800 |
| C | 1.66212200 | -1.78269800 | -1.83365400 |
| H | 0.90823800 | -2.14300000 | -1.14163300 |
| C | 2.76875800 | -2.57858100 | -2.12145400 |
| H | 2.86202600 | -3.55153700 | -1.64872200 |
| C | 3.76164800 | -2.11387300 | -2.99031300 |
| H | 4.63217900 | -2.72908600 | -3.20259000 |
| C | 3.63292100 | -0.85440800 | -3.57894900 |
| H | 4.40034900 | -0.48610700 | -4.25525200 |
| C | 2.51017800 | -0.06361800 | -3.31203000 |
| H | 2.41232200 | 0.90672300 | -3.78999800 |
| P | -1.73877800 | 2.51313600 | 0.53492500 |
| C | -2.50765700 | 2.73414300 | 2.21553600 |
| C | -2.46926600 | 3.95790900 | 2.90341300 |
| H | -2.02370600 | 4.83258900 | 2.44314500 |
| C | -2.99722000 | 4.06877600 | 4.19275700 |
| H | -2.94865500 | 5.02404300 | 4.70921900 |
| C | -3.58390200 | 2.96483300 | 4.81407200 |
| H | -3.99301300 | 3.05311000 | 5.81706300 |
| C | -3.63698100 | 1.74365600 | 4.13608400 |
| H | -4.08923400 | 0.87427500 | 4.60615800 |
| C | -3.09540000 | 1.62906800 | 2.85501500 |
| H | -3.11706300 | 0.66596500 | 2.35660000 |
| C | -1.02002700 | 4.19480300 | 0.25846700 |
| C | -1.73256000 | 5.27971100 | -0.27573500 |
| H | -2.76687100 | 5.15939300 | -0.58041300 |
| C | -1.11718800 | 6.52506900 | -0.42321900 |
| H | -1.67809500 | 7.35335000 | -0.84835200 |
| C | 0.20937200 | 6.70862500 | -0.02297100 |
| H | 0.68587100 | 7.67827600 | -0.14176300 |
| C | 0.91794500 | 5.64088500 | 0.53444800 |
| H | 1.94919500 | 5.76665800 | 0.85072400 |
| C | 0.30420600 | 4.39508900 | 0.67037400 |
| H | 0.86132100 | 3.56396900 | 1.08393000 |
| P | 1.75103800 | 0.68483600 | 1.35838500 |
| C | 1.61365500 | 0.78262400 | 3.19907800 |
| C | 0.37508900 | 1.12772000 | 3.75336200 |
| H | -0.46646800 | 1.29858700 | 3.09289600 |
| C | 0.21499600 | 1.22826200 | 5.13807800 |
| H | -0.75555800 | 1.49719900 | 5.54670900 |
| C | 1.29778000 | 0.97602100 | 5.98290300 |
| H | 1.17798200 | 1.04878400 | 7.06081800 |
| C | 2.53805700 | 0.62086700 | 5.43959800 |
| H | 3.38139600 | 0.41759400 | 6.09464900 |
| C | 2.69615900 | 0.52220400 | 4.05669600 |
| H | 3.66125600 | 0.24218300 | 3.64297700 |
| C | 2.91165100 | 2.06243400 | 0.93807700 |
| C | 3.36979400 | 3.00796800 | 1.86849100 |
| H | 3.08684900 | 2.93046200 | 2.91312400 |
| C | 4.18774200 | 4.06659000 | 1.46126700 |
| H | 4.53060300 | 4.79050500 | 2.19615000 |
| C | 4.56478000 | 4.19441000 | 0.12244700 |
| H | 5.19946000 | 5.01895400 | -0.19122500 |
| C | 4.11805200 | 3.25483300 | -0.81126900 |
| H | 4.40113800 | 3.34234400 | -1.85704200 |
| C | 3.29601700 | 2.20318600 | -0.40731600 |
| H | 2.95802000 | 1.47999800 | -1.14211400 |
| C | 2.84899600 | -0.80566000 | 1.20134000 |
| C | 2.31009400 | -2.04423100 | 1.59061600 |
| C | 4.17196300 | -0.76550700 | 0.73834700 |
| C | 3.07189000 | -3.20914000 | 1.51392300 |
| H | 1.28916600 | -2.09604200 | 1.95372400 |
| C | 4.93260400 | -1.93561600 | 0.65651500 |
| H | 4.62176700 | 0.17425200 | 0.43810300 |
| C | 4.38756500 | -3.16049700 | 1.04374400 |
| H | 2.63414000 | -4.15651200 | 1.81473000 |
| H | 5.95478100 | -1.88353900 | 0.29019900 |
| H | 4.97987900 | -4.06951900 | 0.97892300 |
| C | -3.19677500 | 2.50097900 | -0.60872800 |
| C | -3.00309100 | 2.86850100 | -1.95342600 |
| C | -4.44983100 | 1.99891100 | -0.22634700 |
| C | -4.03171500 | 2.73820100 | -2.88522200 |
| H | -2.04459400 | 3.25598900 | -2.27829800 |
| C | -5.47750300 | 1.86119500 | -1.16464900 |
| H | -4.63709000 | 1.71122800 | 0.80252600 |
| C | -5.27346700 | 2.22807900 | -2.49628600 |
| H | -3.85501800 | 3.02654500 | -3.91809300 |
| H | -6.44043700 | 1.46923600 | -0.84735600 |
| H | -6.07308800 | 2.11749500 | -3.22381000 |
| C | 0.13849600 | 1.95937800 | -3.04818200 |
| C | 0.71209700 | 3.09304600 | -2.45364000 |
| C | -0.34777900 | 2.05352100 | -4.36150100 |
| C | 0.80138000 | 4.29888200 | -3.14982400 |
| H | 1.06324800 | 3.03572800 | -1.43142400 |
| C | -0.26756600 | 3.26290700 | -5.05618300 |
| H | -0.80550700 | 1.19290500 | -4.83900400 |

| | | | |
|---|---|---|---|
| C | 0.30476600 | 4.38746800 | -4.45295500 |
| H | 1.23643400 | 5.16741900 | -2.66310100 |
| H | -0.65690300 | 3.32729500 | -6.06907200 |
| H | 0.35656700 | 5.32846100 | -4.99445700 |
| I | -1.49822600 | -1.37237200 | 1.16386300 |
| C | -1.01442700 | -3.53541600 | 0.12960900 |
| C | -1.39873600 | -3.75953700 | -1.18988800 |
| C | -0.17601200 | -4.41960000 | 0.80285900 |
| C | -0.92536600 | -4.89990000 | -1.85241900 |
| H | -2.04456900 | -3.05897400 | -1.70845700 |
| C | 0.29115200 | -5.56035400 | 0.13497200 |
| H | 0.12338200 | -4.22859800 | 1.83060500 |
| C | -0.07818000 | -5.79632800 | -1.19324700 |
| H | -1.21529600 | -5.07891200 | -2.88569200 |
| H | 0.94715900 | -6.25861200 | 0.65098000 |
| H | 0.29374400 | -6.67605700 | -1.71236800 |

**TS-4-5-Br-PMe$_3$**

E (OSS) = -1797.8223389
E (T) = -1797.8047425
$S^2$(OSS) = 0.5443
$G_{corr}$ = 0.372202
ν = -285.62 $cm^{-1}$

| | | | |
|---|---|---|---|
| Ni | 0.99546000 | -0.07223400 | 0.07217300 |
| P | 0.12096300 | 1.50120700 | -1.24164800 |
| C | -0.36548300 | 3.08635000 | -0.41914600 |
| C | -1.44055900 | 1.07806300 | -2.14034600 |
| C | 1.16432600 | 2.13654800 | -2.63311300 |
| H | -0.82714400 | 3.78808800 | -1.12516400 |
| H | 0.51919300 | 3.55982400 | 0.02003500 |
| H | -1.78383000 | 1.90318900 | -2.77723400 |
| H | -2.22111300 | 0.83728100 | -1.41113400 |
| H | 1.35894200 | 1.32549200 | -3.34215000 |
| H | 2.12659700 | 2.48331800 | -2.24182400 |
| P | 2.51779900 | 0.98121600 | 1.35517700 |
| P | 1.61970200 | -1.83514700 | -1.16992200 |
| C | 3.50039800 | 2.33052700 | 0.54655600 |
| C | 3.89444600 | 0.03185600 | 2.16081800 |
| C | 1.82978200 | 1.87549900 | 2.82187100 |
| C | 1.66641800 | -1.63559500 | -3.01245000 |
| C | 3.31093400 | -2.54471500 | -0.88508300 |
| C | 0.57668500 | -3.35639600 | -1.01513000 |
| H | 4.19289000 | 2.80747800 | 1.25161500 |
| H | 2.82650400 | 3.09393300 | 0.14424400 |
| H | 4.56635100 | 0.69299200 | 2.72341800 |
| H | 4.47408600 | -0.49736400 | 1.39627100 |
| H | 1.32960900 | 1.15272900 | 3.47513300 |
| H | 1.07779000 | 2.59592900 | 2.48250600 |
| H | 2.39584400 | -0.86278100 | -3.27782900 |
| H | 0.68163900 | -1.31768400 | -3.37143900 |
| H | 4.06172200 | -1.76360900 | -1.04969800 |
| H | 3.52163700 | -3.38749900 | -1.55618400 |
| H | 0.54864300 | -3.67050100 | 0.03306100 |
| H | 0.95826500 | -4.18057000 | -1.63092700 |
| H | -1.07310300 | 2.87134200 | 0.38822600 |
| H | -1.27410600 | 0.19161100 | -2.76265700 |
| H | 0.67575200 | 2.96250200 | -3.16477800 |
| H | 1.94320400 | -2.57037100 | -3.51642000 |
| H | 3.39716200 | -2.88907400 | 0.15066500 |
| H | -0.44838800 | -3.12182700 | -1.32116300 |
| H | 2.60606200 | 2.40149100 | 3.39159900 |
| H | 4.07712000 | 1.91167200 | -0.28564100 |
| H | 3.47691800 | -0.71042100 | 2.84824700 |
| Br | -0.81011600 | -0.80367700 | 1.55217200 |
| C | 0.08242600 | -2.41655400 | 2.90350700 |
| C | 1.40610800 | -2.68568300 | 2.61234000 |
| C | -0.61198000 | -3.07278000 | 3.90868300 |
| C | 2.08141000 | -3.65962200 | 3.35939800 |
| H | 1.90069700 | -2.13413400 | 1.80923600 |
| C | 0.06764700 | -4.04789800 | 4.65586600 |
| H | -1.65516500 | -2.84472100 | 4.12039700 |
| C | 1.40860300 | -4.33975800 | 4.38138500 |
| H | 3.12451900 | -3.88705500 | 3.14619100 |
| H | -0.45156500 | -4.57822200 | 5.45194900 |
| H | 1.92809400 | -5.09660700 | 4.96373300 |

**TS-4-5-Cl-PMe$_3$**

E (OSS) = -2244.86583598
E (T) = -2244.84805196
$S^2$(OSS) = 0.5347
$G_{corr}$ = 0.371917
ν = -165.37 $cm^{-1}$

| | | | |
|---|---|---|---|
| Ni | 2.29970700 | 1.34803400 | 0.02861300 |
| P | 1.34468700 | 2.88316800 | -1.26710600 |
| C | 0.84037600 | 4.46645100 | -0.45066700 |
| C | -0.22682300 | 2.42246400 | -2.13208900 |
| C | 2.34618200 | 3.52981500 | -2.68364000 |
| H | 0.35124800 | 5.15219600 | -1.15382700 |
| H | 1.72470900 | 4.96057200 | -0.03375000 |
| H | -0.59616800 | 3.23475700 | -2.77082600 |
| H | -0.99092900 | 2.17717200 | -1.38673800 |
| H | 2.54725400 | 2.71816500 | -3.38984700 |
| H | 3.30706900 | 3.90304200 | -2.31363700 |
| P | 3.83065200 | 2.41819200 | 1.28125100 |
| P | 2.92298000 | -0.40494300 | -1.22331800 |
| C | 4.77074500 | 3.77788500 | 0.44173200 |
| C | 5.23677700 | 1.48829100 | 2.05976300 |
| C | 3.17122100 | 3.30849900 | 2.76573200 |
| C | 2.99173600 | -0.18998600 | -3.06369800 |
| C | 4.59360900 | -1.16107000 | -0.93329200 |
| C | 1.84342000 | -1.90442800 | -1.09353100 |
| H | 5.46985500 | 4.27552000 | 1.12546700 |
| H | 4.07101600 | 4.52276500 | 0.04847000 |
| H | 5.91880300 | 2.15836600 | 2.59902300 |
| H | 5.80057500 | 0.95405700 | 1.28696400 |
| H | 2.68065000 | 2.58427000 | 3.42521700 |

| | | | |
|---|---|---|---|
| H | 2.41640200 | 4.03447000 | 2.44444700 |
| H | 3.73626000 | 0.57294600 | -3.31582200 |
| H | 2.01643300 | 0.14653900 | -3.43125800 |
| H | 5.36644900 | -0.39899200 | -1.08428400 |
| H | 4.78798000 | -2.00238800 | -1.61114200 |
| H | 1.80844300 | -2.23611100 | -0.05066600 |
| H | 2.20508400 | -2.72785600 | -1.72208100 |
| H | 0.15352600 | 4.24596000 | 0.37325600 |
| H | -0.05731300 | 1.53207300 | -2.74789800 |
| H | 1.82761300 | 4.33918800 | -3.21224600 |
| H | 3.25863100 | -1.12502500 | -3.57233800 |
| H | 4.66306500 | -1.51868600 | 0.09934400 |
| H | 0.82396100 | -1.64130100 | -1.39611900 |
| H | 3.95837500 | 3.82913600 | 3.32538800 |
| H | 5.33548900 | 3.36299400 | -0.40055700 |
| H | 4.84072600 | 0.75050000 | 2.76497300 |
| C | 1.34825100 | -0.93052300 | 2.76971700 |
| C | 2.67623400 | -1.21251400 | 2.53262400 |
| C | 0.58388500 | -1.58523100 | 3.71963300 |
| C | 3.29384600 | -2.21466200 | 3.29191600 |
| H | 3.21361000 | -0.65300100 | 1.76225400 |
| C | 1.20799400 | -2.58950500 | 4.47732100 |
| H | -0.46353700 | -1.33788900 | 3.88025800 |
| C | 2.55569100 | -2.90133700 | 4.26349700 |
| H | 4.34166500 | -2.45919400 | 3.12703300 |
| H | 0.63961800 | -3.12686500 | 5.23401400 |
| H | 3.03027300 | -3.68048400 | 4.85448100 |
| Cl | 0.60732300 | 0.62395700 | 1.48038700 |

**5-I-PMe$_3$**

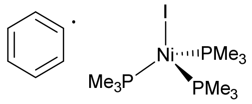

E (OSS) = -1796.05732288

E (T) = -1796.05665155

$S^2$(OSS) = 1.0067

$G_{corr}$ = 0.366149

| | | | |
|---|---|---|---|
| Ni | -0.62583000 | 0.66902000 | 0.02368500 |
| P | -1.21459500 | 2.27719400 | -1.52055600 |
| C | -1.59541400 | 3.90919700 | -0.75042900 |
| C | -2.77287800 | 1.91895600 | -2.43877600 |
| C | -0.05852400 | 2.75157000 | -2.88104000 |
| H | -1.97593900 | 4.62646200 | -1.48736700 |
| H | -0.69109300 | 4.31914200 | -0.28898500 |
| H | -3.05842200 | 2.75133400 | -3.09273100 |
| H | -3.57373000 | 1.73183400 | -1.71626900 |
| H | 0.12216000 | 1.88801300 | -3.52897800 |
| H | 0.90012900 | 3.06789400 | -2.45640200 |
| P | 0.92071400 | 1.77038500 | 1.30936900 |
| P | -0.26235200 | -1.26585900 | -1.16032400 |
| C | 2.12032600 | 2.82469800 | 0.37654700 |
| C | 2.06860700 | 0.74664000 | 2.33593400 |
| C | 0.26492700 | 2.95787400 | 2.56069700 |
| C | -0.16078800 | -1.11491800 | -2.99818900 |
| C | 1.30647700 | -2.17209800 | -0.78691700 |
| C | -1.52133500 | -2.59788800 | -0.95439900 |
| H | 2.85500800 | 3.28431000 | 1.04878900 |
| H | 1.58525200 | 3.61957000 | -0.15311000 |
| H | 2.81516600 | 1.37188400 | 2.84023000 |
| H | 2.58287300 | 0.02253400 | 1.69481000 |
| H | -0.36872100 | 2.41590900 | 3.27036800 |
| H | -0.35733100 | 3.70565100 | 2.05863600 |
| H | 0.65928700 | -0.44046000 | -3.26536700 |
| H | -1.09532300 | -0.69701800 | -3.38561700 |
| H | 2.16302400 | -1.51127900 | -0.96005400 |
| H | 1.41230300 | -3.06662600 | -1.41297600 |
| H | -1.60216400 | -2.85326500 | 0.10709600 |
| H | -1.26058900 | -3.49684900 | -1.52601400 |
| H | -2.34527600 | 3.76313300 | 0.03365500 |
| H | -2.64378500 | 1.01588800 | -3.04443300 |
| H | -0.46855800 | 3.56929700 | -3.48536100 |
| H | 0.01268700 | -2.09024600 | -3.46882800 |
| H | 1.30932100 | -2.47695500 | 0.26381400 |
| H | -2.49641600 | -2.22573200 | -1.28606900 |
| H | 1.07238800 | 3.46179400 | 3.10567800 |
| H | 2.64622400 | 2.21133000 | -0.36302000 |
| H | 1.49876700 | 0.19883500 | 3.09219700 |
| I | -2.96508700 | 0.44484800 | 1.44693400 |
| C | -1.47822700 | -2.52553600 | 3.01822400 |
| C | -0.23174800 | -2.08815200 | 2.62358400 |
| C | -1.72649500 | -3.63587100 | 3.79857000 |
| C | 0.87411600 | -2.83807600 | 3.05980500 |
| H | -0.10690000 | -1.20774200 | 1.99961100 |
| C | -0.60705200 | -4.37576700 | 4.22059300 |
| H | -2.73196900 | -3.93678500 | 4.08205200 |
| C | 0.68184100 | -3.97531400 | 3.85215200 |
| H | 1.87862600 | -2.52927800 | 2.77919000 |
| H | -0.74930700 | -5.26110300 | 4.83629900 |
| H | 1.54082800 | -4.55158800 | 4.18574000 |

**5-I-PMe$_2$Ph**

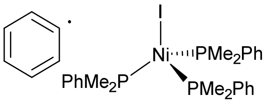

E (OSS) = -2371.44147589

E (T) = -2371.44128873

$S^2$(OSS) = 1.0117

$G_{corr}$ = 0.516916

| | | | |
|---|---|---|---|
| Ni | -0.47346700 | 0.63783000 | -0.19883400 |
| P | 0.06944800 | -0.28957200 | -2.22969100 |
| P | -1.77334800 | -1.00075600 | 0.75351400 |
| P | 1.27933300 | 1.02946800 | 1.22852300 |
| C | 1.72350300 | 0.37709700 | -2.67487600 |
| C | 1.82564600 | 1.73960900 | -3.01012100 |

| | | | |
|---|---|---|---|
| H | 0.93005200 | 2.35218100 | -3.06378600 |
| C | 3.07097700 | 2.32152000 | -3.24730600 |
| H | 3.13070500 | 3.37519000 | -3.50746700 |
| C | 4.23769300 | 1.55763100 | -3.13818400 |
| H | 5.20809300 | 2.01367500 | -3.31551800 |
| C | 4.14785300 | 0.20593500 | -2.79814800 |
| H | 5.04911400 | -0.39598300 | -2.71299700 |
| C | 2.89988100 | -0.38244800 | -2.57006600 |
| H | 2.85365000 | -1.43611700 | -2.31149000 |
| C | -0.94539100 | 0.06111500 | -3.73245500 |
| H | -1.91550900 | -0.43272800 | -3.61589000 |
| H | -0.45945200 | -0.29782600 | -4.64640100 |
| H | -1.12152700 | 1.13809800 | -3.80884900 |
| C | 0.25863000 | -2.12250800 | -2.32155000 |
| H | -0.73520000 | -2.56498100 | -2.19506700 |
| H | 0.89639600 | -2.47955500 | -1.50738600 |
| H | 0.67544200 | -2.45050900 | -3.28037800 |
| C | -3.01961200 | -1.60297100 | -0.45885600 |
| C | -3.39308700 | -2.94689600 | -0.60966400 |
| H | -2.95377100 | -3.71600300 | 0.01893300 |
| C | -4.33358400 | -3.31424100 | -1.57711600 |
| H | -4.61004800 | -4.35964500 | -1.68904100 |
| C | -4.91799600 | -2.34396300 | -2.39488000 |
| H | -5.64967200 | -2.63258200 | -3.14491300 |
| C | -4.55403400 | -1.00137000 | -2.24921500 |
| H | -4.99837400 | -0.24157800 | -2.88686300 |
| C | -3.60306800 | -0.63526300 | -1.29566500 |
| H | -3.28803500 | 0.40257300 | -1.20859800 |
| C | -2.81318200 | -0.52293600 | 2.20035500 |
| H | -3.47238600 | 0.29658100 | 1.90639500 |
| H | -2.16652000 | -0.16877200 | 3.00791400 |
| H | -3.41636800 | -1.36732800 | 2.55272500 |
| C | -0.93729100 | -2.52434900 | 1.36880800 |
| H | -0.23065100 | -2.22880900 | 2.15081100 |
| H | -0.37498900 | -2.99982600 | 0.55941100 |
| H | -1.64192300 | -3.24519900 | 1.79721200 |
| C | 0.92661400 | 1.50088400 | 2.96959000 |
| C | 0.49585100 | 0.51734300 | 3.87805100 |
| H | 0.42743200 | -0.52111000 | 3.56397900 |
| C | 0.15072600 | 0.85648600 | 5.18659900 |
| H | -0.17497200 | 0.08168400 | 5.87606800 |
| C | 0.22096500 | 2.18804400 | 5.60956100 |
| H | -0.04885400 | 2.45310900 | 6.62831600 |
| C | 0.63853800 | 3.17440700 | 4.71309500 |
| H | 0.69331900 | 4.21241900 | 5.03077600 |
| C | 0.98964300 | 2.83485900 | 3.40316900 |
| H | 1.30739100 | 3.61770500 | 2.72133600 |
| C | 2.44183800 | -0.39760800 | 1.41990400 |
| H | 1.91324400 | -1.26829200 | 1.81899800 |
| H | 3.28633600 | -0.15686200 | 2.07515000 |
| H | 2.81887100 | -0.65716800 | 0.42452800 |
| C | 2.41760000 | 2.34891400 | 0.62647300 |
| H | 1.85049200 | 3.25909100 | 0.41202400 |
| H | 2.86031500 | 2.00346000 | -0.31288500 |
| H | 3.21656100 | 2.56698000 | 1.34384900 |
| I | -1.41186000 | 3.09657400 | -0.84530200 |
| C | -3.57582800 | 3.09596400 | 1.93138800 |
| C | -2.74140000 | 2.99272600 | 3.02567700 |
| C | -4.88991200 | 2.67410500 | 1.88712300 |
| C | -3.28619800 | 2.42351800 | 4.18922500 |
| H | -1.70533300 | 3.31642400 | 2.99949200 |
| C | -5.41472100 | 2.10270700 | 3.06017200 |
| H | -5.50044700 | 2.77065100 | 0.99261800 |
| C | -4.61329000 | 1.98008800 | 4.20069500 |
| H | -2.66285400 | 2.31810200 | 5.07373300 |
| H | -6.44509100 | 1.75404200 | 3.07561000 |
| H | -5.02530300 | 1.53405600 | 5.10232000 |

**5-I-PMePh$_2$**

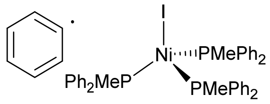


E (OSS) = -2946.82852389

E (T) = -2946.82830820

$S^2$(OSS) = 1.0113

$G_{corr}$ = 0.66871

| | | | |
|---|---|---|---|
| Ni | -0.82914300 | 0.81738700 | -0.09476500 |
| P | -0.99630500 | 0.75977400 | -2.40028600 |
| C | -2.54665700 | -0.02156100 | -3.02420600 |
| C | -3.67366400 | 0.73562300 | -3.37850400 |
| H | -3.62706100 | 1.81968900 | -3.38636900 |
| C | -4.87373800 | 0.10522400 | -3.72136900 |
| H | -5.73711400 | 0.70722500 | -3.99324100 |
| C | -4.96352400 | -1.28862200 | -3.71853900 |
| H | -5.89726000 | -1.77734800 | -3.98348900 |
| C | -3.84138200 | -2.05153800 | -3.37884000 |
| H | -3.89810000 | -3.13707200 | -3.37903600 |
| C | -2.64446000 | -1.42436600 | -3.03468300 |
| H | -1.78352200 | -2.02842700 | -2.76401600 |
| C | 0.29435000 | -0.23603600 | -3.25418400 |
| C | 1.06201600 | -1.12705100 | -2.49144600 |
| H | 0.91198300 | -1.17991300 | -1.41940300 |
| C | 2.01792000 | -1.94238600 | -3.10279300 |
| H | 2.59833200 | -2.63255500 | -2.49726500 |
| C | 2.22124900 | -1.86587700 | -4.48188100 |
| H | 2.96814900 | -2.49504600 | -4.95919700 |
| C | 1.46020200 | -0.97834800 | -5.25070600 |
| H | 1.61404100 | -0.91852700 | -6.32513600 |
| C | 0.49784400 | -0.17174000 | -4.64250700 |
| H | -0.09830400 | 0.49887500 | -5.25516100 |
| C | -0.94003000 | 2.38719900 | -3.27172900 |
| H | -1.16629000 | 2.31215900 | -4.33994700 |
| H | 0.06631700 | 2.79801600 | -3.14315200 |
| H | -1.64902800 | 3.07600300 | -2.80417400 |
| P | -2.46799400 | 2.31900000 | 0.60307100 |
| C | -3.22162100 | 1.97850000 | 2.24412000 |
| C | -2.38971900 | 1.98245400 | 3.37648900 |

| | | | |
|---|---|---|---|
| H | -1.33999600 | 2.23856300 | 3.27703900 |
| C | -2.90162100 | 1.64812100 | 4.63075400 |
| H | -2.24367700 | 1.66290300 | 5.49438800 |
| C | -4.24566200 | 1.28956500 | 4.77031100 |
| H | -4.63974300 | 1.01791800 | 5.74611500 |
| C | -5.07752500 | 1.27869500 | 3.64917000 |
| H | -6.12205800 | 0.99474000 | 3.74512500 |
| C | -4.57170600 | 1.62702700 | 2.39414300 |
| H | -5.23537700 | 1.60081700 | 1.53694300 |
| C | -1.78701500 | 4.02238300 | 0.76219600 |
| C | -2.35495300 | 4.98949200 | 1.60570300 |
| H | -3.20059600 | 4.73254300 | 2.23747000 |
| C | -1.82836800 | 6.28162100 | 1.64940800 |
| H | -2.27040600 | 7.02185900 | 2.31135200 |
| C | -0.73512000 | 6.62282400 | 0.84607100 |
| H | -0.32545700 | 7.62876400 | 0.88354700 |
| C | -0.16648300 | 5.66540600 | 0.00162700 |
| H | 0.69047000 | 5.92008000 | -0.61623700 |
| C | -0.68572200 | 4.36893300 | -0.03467000 |
| H | -0.22256500 | 3.61480300 | -0.66345500 |
| C | -3.93018600 | 2.62740100 | -0.48317700 |
| H | -4.62772900 | 3.33521400 | -0.02371100 |
| H | -4.44595500 | 1.69086400 | -0.70964700 |
| H | -3.56481600 | 3.05566600 | -1.41987200 |
| P | -1.24389700 | -1.01566300 | 1.24096100 |
| C | -0.51546300 | -2.54330400 | 0.50794500 |
| C | 0.84949900 | -2.81468600 | 0.70796000 |
| H | 1.44604200 | -2.17411100 | 1.35049300 |
| C | 1.45909500 | -3.89466800 | 0.06601500 |
| H | 2.51522700 | -4.09053700 | 0.23246400 |
| C | 0.71899300 | -4.71485200 | -0.79014000 |
| H | 1.19630400 | -5.55111000 | -1.29382100 |
| C | -0.63809400 | -4.45256900 | -0.99350300 |
| H | -1.22403400 | -5.08710200 | -1.65374900 |
| C | -1.25201600 | -3.37612200 | -0.34928300 |
| H | -2.30675500 | -3.18487100 | -0.52058100 |
| C | -3.03073200 | -1.40900300 | 1.41072000 |
| C | -3.86702800 | -1.07740700 | 0.33195700 |
| H | -3.43996300 | -0.59752100 | -0.54297800 |
| C | -5.23542800 | -1.34711200 | 0.38150100 |
| H | -5.86392000 | -1.09526200 | -0.46859000 |
| C | -5.78957000 | -1.92716800 | 1.52588100 |
| H | -6.85671600 | -2.12757600 | 1.57434800 |
| C | -4.96797800 | -2.24366500 | 2.61154600 |
| H | -5.39621200 | -2.68796700 | 3.50631700 |
| C | -3.59444000 | -1.99498900 | 2.55323900 |
| H | -2.97189300 | -2.25181800 | 3.40479000 |
| C | -0.57820800 | -1.05408700 | 2.96262700 |
| H | -0.60538400 | -2.05779600 | 3.39807100 |
| H | -1.15787000 | -0.36633900 | 3.58248600 |
| H | 0.45531300 | -0.69812000 | 2.93593200 |
| I | 1.79383400 | 1.32691500 | 0.41308500 |
| C | 1.30058500 | 2.90209500 | 3.41749700 |
| C | 1.26057200 | 2.02587700 | 4.48494000 |
| C | 0.69834600 | 4.14460000 | 3.39023300 |
| C | 0.56788000 | 2.44624600 | 5.63350900 |
| H | 1.73858200 | 1.05029100 | 4.44880300 |
| C | 0.00561900 | 4.54507700 | 4.54540200 |
| H | 0.73315100 | 4.78567100 | 2.51438100 |
| C | -0.05468600 | 3.69965900 | 5.65831000 |
| H | 0.51327300 | 1.79163800 | 6.50079000 |
| H | -0.49205900 | 5.51185300 | 4.56347700 |
| H | -0.59671000 | 4.01433800 | 6.54630900 |

**5-I-PPh$_3$**

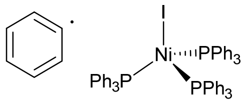


E (OSS) = -3522.20750246

E (T) = -3522.20736581

$S^2$(OSS) = 1.0130

$G_{corr}$ = 0.817883

| | | | |
|---|---|---|---|
| Ni | -0.40588100 | 0.68315600 | 0.44006400 |
| P | -0.13489200 | 0.36030300 | -1.86713900 |
| C | -1.48976200 | -0.56292200 | -2.70445400 |
| C | -2.76485500 | -0.56204000 | -2.12300600 |
| H | -2.92697900 | -0.04271400 | -1.18684000 |
| C | -3.82188700 | -1.23799200 | -2.73649600 |
| H | -4.80454200 | -1.22761900 | -2.27369400 |
| C | -3.61264800 | -1.92540000 | -3.93452300 |
| H | -4.43374700 | -2.45593700 | -4.40952400 |
| C | -2.34197800 | -1.93510500 | -4.51896700 |
| H | -2.17230400 | -2.47300100 | -5.44811700 |
| C | -1.28423400 | -1.26230500 | -3.90518400 |
| H | -0.29726200 | -1.29058900 | -4.35720600 |
| C | 1.35227300 | -0.63351900 | -2.29498000 |
| C | 1.63618300 | -1.74050100 | -1.48068500 |
| H | 0.98102200 | -1.97856100 | -0.64952300 |
| C | 2.76525300 | -2.52282800 | -1.72522300 |
| H | 2.97720100 | -3.36729800 | -1.07832000 |
| C | 3.62349400 | -2.20336000 | -2.78058400 |
| H | 4.50963000 | -2.80580400 | -2.96316100 |
| C | 3.34415400 | -1.10460600 | -3.59844600 |
| H | 4.00830200 | -0.85304800 | -4.42148200 |
| C | 2.21153300 | -0.32210300 | -3.35996100 |
| H | 2.00588100 | 0.53446700 | -3.99491000 |
| P | -1.82372700 | 2.58387300 | 0.61679400 |
| C | -2.59607900 | 2.84107100 | 2.28168700 |
| C | -2.59040300 | 4.08745400 | 2.92931300 |
| H | -2.15907900 | 4.95648100 | 2.44479600 |
| C | -3.13634300 | 4.22500600 | 4.20764900 |
| H | -3.11984300 | 5.19680200 | 4.69429100 |
| C | -3.70074100 | 3.12470400 | 4.85746100 |
| H | -4.12409700 | 3.23477800 | 5.85236700 |

| | | | |
|---|---|---|---|
| C | -3.71351700 | 1.88107100 | 4.22070900 |
| H | -4.14516200 | 1.01513300 | 4.71570400 |
| C | -3.15992400 | 1.73884000 | 2.94692700 |
| H | -3.14961600 | 0.76149100 | 2.47733500 |
| C | -1.04349600 | 4.22527500 | 0.31405700 |
| C | -1.71789100 | 5.32598900 | -0.23655300 |
| H | -2.75410000 | 5.23439300 | -0.54573200 |
| C | -1.06068800 | 6.54842900 | -0.39345700 |
| H | -1.59072600 | 7.39054600 | -0.83060400 |
| C | 0.26881700 | 6.69113200 | 0.01339900 |
| H | 0.77784500 | 7.64321700 | -0.11173900 |
| C | 0.94079800 | 5.60526000 | 0.58125500 |
| H | 1.97606000 | 5.70068600 | 0.89507100 |
| C | 0.28696800 | 4.38172800 | 0.72606900 |
| H | 0.81672400 | 3.53346000 | 1.14420000 |
| P | 1.76003800 | 0.63596600 | 1.45006800 |
| C | 1.61419700 | 1.02358300 | 3.24760000 |
| C | 0.35435700 | 1.34367800 | 3.76936000 |
| H | -0.51350900 | 1.34541700 | 3.12114900 |
| C | 0.19870900 | 1.64004200 | 5.12618600 |
| H | -0.78820000 | 1.88499300 | 5.50943500 |
| C | 1.30694900 | 1.61415300 | 5.97448200 |
| H | 1.19124300 | 1.84382800 | 7.03050000 |
| C | 2.56816500 | 1.28196000 | 5.46567000 |
| H | 3.43088700 | 1.25169800 | 6.12605700 |
| C | 2.72159100 | 0.98310000 | 4.11197300 |
| H | 3.70154100 | 0.71780700 | 3.72430100 |
| C | 2.96175900 | 1.88106700 | 0.80786600 |
| C | 3.37699600 | 3.00442200 | 1.53911300 |
| H | 3.03939100 | 3.14825800 | 2.56014100 |
| C | 4.22208700 | 3.95533900 | 0.95903900 |
| H | 4.53233300 | 4.81966700 | 1.54058500 |
| C | 4.66680600 | 3.79757800 | -0.35527400 |
| H | 5.32176700 | 4.53972500 | -0.80373600 |
| C | 4.26613000 | 2.67692500 | -1.08858500 |
| H | 4.60398700 | 2.54000200 | -2.11239200 |
| C | 3.41880200 | 1.73020000 | -0.51395700 |
| H | 3.11466300 | 0.86734000 | -1.09668000 |
| C | 2.77779900 | -0.91180800 | 1.52095900 |
| C | 2.13290200 | -2.09221900 | 1.92725200 |
| C | 4.14777000 | -0.95829400 | 1.22367600 |
| C | 2.84134000 | -3.28958300 | 2.02740700 |
| H | 1.07152700 | -2.08073300 | 2.15251900 |
| C | 4.85285700 | -2.16161200 | 1.31555100 |
| H | 4.67512500 | -0.06306000 | 0.91320800 |
| C | 4.20394300 | -3.33053200 | 1.71684200 |
| H | 2.32474000 | -4.19150300 | 2.34358300 |
| H | 5.91285600 | -2.17963000 | 1.07508700 |
| H | 4.75397400 | -4.26522200 | 1.78874500 |

| | | | |
|---|---|---|---|
| C | -3.25323200 | 2.54371700 | -0.55156200 |
| C | -3.04432200 | 2.89675500 | -1.89669900 |
| C | -4.50264500 | 2.02577000 | -0.17706500 |
| C | -4.05769700 | 2.73445300 | -2.84079300 |
| H | -2.08922800 | 3.29998400 | -2.21157500 |
| C | -5.51452500 | 1.86098900 | -1.12672400 |
| H | -4.69613800 | 1.74421200 | 0.85246800 |
| C | -5.29598100 | 2.21064700 | -2.46113300 |
| H | -3.87135100 | 3.00983400 | -3.87548800 |
| H | -6.47549900 | 1.45805200 | -0.81779800 |
| H | -6.08310800 | 2.07604900 | -3.19809800 |
| C | 0.06073000 | 1.89231200 | -2.86807900 |
| C | 0.74273000 | 2.95674200 | -2.26082200 |
| C | -0.46449100 | 2.06683700 | -4.15775400 |
| C | 0.90015700 | 4.17458100 | -2.92287600 |
| H | 1.12886400 | 2.83789600 | -1.25614000 |
| C | -0.31310600 | 3.28829200 | -4.81826900 |
| H | -1.01010500 | 1.26275100 | -4.64055100 |
| C | 0.36657100 | 4.34434100 | -4.20286700 |
| H | 1.41867400 | 4.98990600 | -2.42678400 |
| H | -0.73247500 | 3.41621700 | -5.81291700 |
| H | 0.47345400 | 5.29582100 | -4.71720700 |
| I | -1.71863700 | -1.49268900 | 1.40740900 |
| C | -0.68932000 | -4.30022600 | -0.44170500 |
| C | -1.10708800 | -4.02038000 | -1.72671900 |
| C | 0.38617900 | -5.09841400 | -0.10791500 |
| C | -0.37889700 | -4.61075900 | -2.77306200 |
| H | -1.94645400 | -3.36352900 | -1.93217900 |
| C | 1.10013200 | -5.68127800 | -1.16965500 |
| H | 0.67953800 | -5.27447800 | 0.92346500 |
| C | 0.71610100 | -5.43485000 | -2.49236000 |
| H | -0.66684400 | -4.41262100 | -3.80257800 |
| H | 1.95642700 | -6.31808400 | -0.95758600 |
| H | 1.27831100 | -5.88132500 | -3.30843500 |

**5-Br-$PMe_3$**

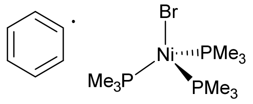


E (OSS) = -1797.83450227

E (T) = -1797.83414397

$S^2$(OSS) = 1.0115

$G_{corr}$ = 0.368688

| | | | |
|---|---|---|---|
| Ni | -0.52646100 | 0.51920300 | 0.11520900 |
| P | -1.03997200 | 2.06473100 | -1.51557600 |
| C | -1.42631300 | 3.72917300 | -0.81907400 |
| C | -2.59719800 | 1.66490900 | -2.41914000 |
| C | 0.11530300 | 2.49095400 | -2.89387400 |

| | | | |
|---|---|---|---|
| H | -1.79433000 | 4.41688200 | -1.58995200 |
| H | -0.52683600 | 4.15598100 | -0.36337400 |
| H | -2.90587200 | 2.47993200 | -3.08457900 |
| H | -3.38389700 | 1.47166000 | -1.68295500 |
| H | 0.31938200 | 1.59814900 | -3.49366700 |
| H | 1.06447500 | 2.84831300 | -2.47981100 |
| P | 1.03040700 | 1.65022300 | 1.36496800 |
| P | -0.09244600 | -1.45615200 | -0.97251200 |
| C | 2.25490800 | 2.67407800 | 0.42725800 |
| C | 2.15241700 | 0.66673300 | 2.45797200 |
| C | 0.36023400 | 2.88318800 | 2.56550700 |
| C | 0.10131400 | -1.35316700 | -2.80825500 |
| C | 1.44831400 | -2.37213500 | -0.51399800 |
| C | -1.37002500 | -2.77764800 | -0.80861200 |
| H | 2.98539300 | 3.14266300 | 1.09823000 |
| H | 1.73326900 | 3.46241400 | -0.12574400 |
| H | 2.89546400 | 1.30627100 | 2.94968800 |
| H | 2.66967600 | -0.09317300 | 1.86277500 |
| H | -0.28906500 | 2.36540100 | 3.27876600 |
| H | -0.25073800 | 3.61649700 | 2.02946700 |
| H | 0.94081200 | -0.69281900 | -3.04955300 |
| H | -0.80728900 | -0.93341200 | -3.25180000 |
| H | 2.31928000 | -1.73121600 | -0.69074700 |
| H | 1.55776000 | -3.29401600 | -1.09859300 |
| H | -1.51614200 | -3.00782400 | 0.25122300 |
| H | -1.08436900 | -3.69150900 | -1.34388000 |
| H | -2.18704700 | 3.61769000 | -0.03997200 |
| H | -2.45523800 | 0.75444900 | -3.01115800 |
| H | -0.30399200 | 3.26756600 | -3.54465100 |
| H | 0.28665300 | -2.34035100 | -3.24915300 |
| H | 1.41719600 | -2.62924600 | 0.54910800 |
| H | -2.32156600 | -2.40563500 | -1.20299500 |
| H | 1.15903400 | 3.40245500 | 3.10936300 |
| H | 2.78560500 | 2.04210200 | -0.29313400 |
| H | 1.55995300 | 0.16142200 | 3.22666500 |
| Br | -2.69413700 | 0.39703300 | 1.41336500 |
| C | -1.39611700 | -2.90546100 | 3.02771100 |
| C | -0.24156200 | -2.17690000 | 2.83695700 |
| C | -1.48129600 | -4.14069000 | 3.63498000 |
| C | 0.95034100 | -2.75368600 | 3.31042000 |
| H | -0.25430800 | -1.21121000 | 2.33860700 |
| C | -0.27637600 | -4.69855300 | 4.09947400 |
| H | -2.42400500 | -4.66812100 | 3.75781300 |
| C | 0.92768500 | -4.00582000 | 3.93544500 |
| H | 1.89044700 | -2.22209400 | 3.18669600 |
| H | -0.28666700 | -5.67107200 | 4.58643900 |
| H | 1.85407100 | -4.44311900 | 4.29842700 |

**5-Cl-$PMe_3$**

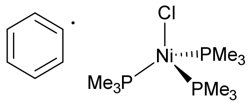


E (OSS) = -2244.88718919

E (T) = -2244.88685062

$S^2$(OSS) = 1.0118

$G_{corr}$ = 0.370729

| | | | |
|---|---|---|---|
| Ni | 1.55939600 | 1.02993200 | 0.06773100 |
| P | 1.04825600 | 2.57899500 | -1.56715400 |
| C | 0.70765900 | 4.26514800 | -0.89608100 |
| C | -0.52776500 | 2.20181400 | -2.44952300 |
| C | 2.19308300 | 2.96344700 | -2.96638200 |
| H | 0.35866800 | 4.94908200 | -1.67891300 |
| H | 1.61833100 | 4.67552900 | -0.44749100 |
| H | -0.82126400 | 3.01148300 | -3.12816800 |
| H | -1.31737300 | 2.04269800 | -1.70812500 |
| H | 2.36051000 | 2.06418000 | -3.56739000 |
| H | 3.15912300 | 3.29502900 | -2.57040900 |
| P | 3.14451200 | 2.15052800 | 1.29508500 |
| P | 2.01828400 | -0.93475900 | -1.03686400 |
| C | 4.36990300 | 3.14620100 | 0.32855300 |
| C | 4.27115900 | 1.16728500 | 2.38392700 |
| C | 2.51152200 | 3.40874100 | 2.49158400 |
| C | 2.22532100 | -0.82001700 | -2.87031200 |
| C | 3.55355400 | -1.85948900 | -0.57730800 |
| C | 0.73865200 | -2.25829400 | -0.89125900 |
| H | 5.11802800 | 3.61091800 | 0.98258200 |
| H | 3.85238500 | 3.93654800 | -0.22533100 |
| H | 5.02233300 | 1.80499100 | 2.86561100 |
| H | 4.77967600 | 0.40239300 | 1.78756600 |
| H | 1.87482100 | 2.90929700 | 3.22954500 |
| H | 1.89618400 | 4.14049600 | 1.95788100 |
| H | 3.07626400 | -0.17022700 | -3.09997100 |
| H | 1.32617200 | -0.38313200 | -3.31676500 |
| H | 4.42774900 | -1.22032100 | -0.74409600 |
| H | 3.66359800 | -2.77651900 | -1.16934400 |
| H | 0.58548900 | -2.49827600 | 0.16578600 |
| H | 1.02730800 | -3.16861300 | -1.43074800 |
| H | -0.05682800 | 4.19294300 | -0.11575800 |
| H | -0.41277400 | 1.27685600 | -3.02492000 |
| H | 1.78570500 | 3.75014000 | -3.61250100 |
| H | 2.39937500 | -1.80582900 | -3.31870400 |
| H | 3.51683300 | -2.12683800 | 0.48315200 |
| H | -0.21062200 | -1.88502100 | -1.29104400 |
| H | 3.32608200 | 3.92850700 | 3.01070900 |
| H | 4.88037700 | 2.49875300 | -0.39274700 |
| H | 3.68349800 | 0.66830000 | 3.16033600 |
| C | 0.68597800 | -2.34195600 | 2.94746600 |
| C | 1.85798100 | -1.64349800 | 2.75283900 |
| C | 0.56468800 | -3.55656800 | 3.58863100 |
| C | 3.03056700 | -2.23191100 | 3.25952600 |
| H | 1.87552500 | -0.69278400 | 2.22682300 |
| C | 1.75067100 | -4.12626300 | 4.08616800 |
| H | -0.39103800 | -4.05952800 | 3.71303700 |
| C | 2.97177800 | -3.46452800 | 3.91950700 |
| H | 3.98335100 | -1.72384500 | 3.13391400 |
| H | 1.71235800 | -5.08358100 | 4.60100600 |
| H | 3.88322600 | -3.91105500 | 4.30813000 |

| | | | |
|---|---|---|---|
| Cl | -0.47285000 | 0.85701500 | 1.30676200 |

**6-I-PMe$_3$**

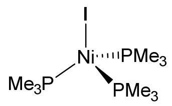


E = -1564.41378215

$G_{corr}$ = 0.285068

| | | | |
|---|---|---|---|
| Ni | 3.19335000 | 7.61491500 | 11.71328600 |
| P | 1.42114100 | 8.89806000 | 11.02938100 |
| C | 0.92926700 | 10.20501400 | 12.23542700 |
| C | -0.14454400 | 7.94826000 | 10.80984700 |
| C | 1.53713400 | 9.85467100 | 9.45469000 |
| H | 0.00722700 | 10.71403700 | 11.93061000 |
| H | 1.73419900 | 10.94368500 | 12.31335900 |
| H | -0.99097200 | 8.60345000 | 10.57235500 |
| H | -0.35757600 | 7.39969300 | 11.73337800 |
| H | 1.66951500 | 9.16654200 | 8.61384500 |
| H | 2.41126400 | 10.51382600 | 9.49478900 |
| P | 4.67452100 | 9.10374900 | 12.61927200 |
| P | 3.72089000 | 6.19142300 | 10.00155600 |
| C | 5.17192800 | 10.56633900 | 11.60707600 |
| C | 6.29539000 | 8.30412600 | 12.99476300 |
| C | 4.24443100 | 9.87664900 | 14.23909700 |
| C | 3.47263200 | 6.77897500 | 8.26781500 |
| C | 5.51298200 | 5.74015200 | 9.99938200 |
| C | 2.91216400 | 4.53276300 | 9.96311700 |
| H | 5.94598000 | 11.16362400 | 12.10392800 |
| H | 4.29733600 | 11.20149600 | 11.42812700 |
| H | 6.99713300 | 9.00042000 | 13.46914000 |
| H | 6.73882700 | 7.92256500 | 12.06883300 |
| H | 4.00335100 | 9.08339400 | 14.95427800 |
| H | 3.35683100 | 10.50649200 | 14.12017600 |
| H | 3.98225500 | 7.73855400 | 8.13028300 |
| H | 2.40375800 | 6.92744900 | 8.08120600 |
| H | 6.11448200 | 6.64307500 | 9.84946500 |
| H | 5.74919300 | 5.01732500 | 9.20899900 |
| H | 3.14119400 | 4.00121000 | 10.89240200 |
| H | 3.25166400 | 3.93374700 | 9.10942800 |
| H | 0.78299700 | 9.74791500 | 13.21948000 |
| H | -0.01831900 | 7.21718900 | 10.00393800 |
| H | 0.63923400 | 10.46021600 | 9.28337800 |
| H | 3.86141000 | 6.05808400 | 7.53849000 |
| H | 5.77644700 | 5.30789500 | 10.97047500 |
| H | 1.82558000 | 4.65920500 | 9.90808200 |
| H | 5.06726700 | 10.48533600 | 14.63304300 |
| H | 5.55019000 | 10.22549200 | 10.63698000 |
| H | 6.12182700 | 7.45502400 | 13.66458500 |
| I | 2.49872700 | 6.02825200 | 13.83966400 |

## 6-I-$PMe_2Ph$

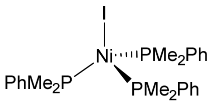


E = -2139.7985927

$G_{corr}$ = 0.434689

| | | | |
|---|---|---|---|
| Ni | -0.46362800 | 1.21841800 | -0.09111500 |
| P | 0.03894400 | 0.13975600 | -2.06019400 |
| P | -1.93893900 | -0.17262100 | 0.98647200 |
| P | 1.30022600 | 1.36634700 | 1.36474300 |
| C | 1.73705100 | 0.66483800 | -2.54471300 |
| C | 2.03141300 | 2.03737700 | -2.44873600 |
| H | 1.26480900 | 2.72544300 | -2.09743600 |
| C | 3.30133900 | 2.51779400 | -2.77160900 |
| H | 3.51084800 | 3.58162000 | -2.69537400 |
| C | 4.30389700 | 1.63001400 | -3.17500400 |
| H | 5.29639100 | 2.00072800 | -3.41734100 |
| C | 4.02606400 | 0.26374900 | -3.25997500 |
| H | 4.80234000 | -0.43143000 | -3.56966700 |
| C | 2.74963700 | -0.21745700 | -2.95155500 |
| H | 2.55443100 | -1.28303400 | -3.02847300 |
| C | -0.91300800 | 0.40014300 | -3.62396700 |
| H | -1.92519300 | 0.00436800 | -3.49237300 |
| H | -0.43513600 | -0.09777100 | -4.47535000 |
| H | -0.98541400 | 1.47388900 | -3.82410200 |
| C | 0.11997800 | -1.69945500 | -1.96995800 |
| H | -0.88360200 | -2.06743700 | -1.73433200 |
| H | 0.79409100 | -1.99752900 | -1.16076200 |
| H | 0.44883200 | -2.15868800 | -2.90864800 |
| C | -3.06485900 | -1.02538300 | -0.18939800 |
| C | -3.37660600 | -2.39189900 | -0.13477600 |
| H | -2.96891600 | -3.01912700 | 0.65274100 |
| C | -4.21194000 | -2.96474000 | -1.09948100 |
| H | -4.44108500 | -4.02627000 | -1.05077900 |
| C | -4.75161400 | -2.17831300 | -2.11983200 |
| H | -5.40136400 | -2.62574100 | -2.86736200 |
| C | -4.44970400 | -0.81369900 | -2.17889200 |
| H | -4.86207400 | -0.19631800 | -2.97274700 |
| C | -3.60417000 | -0.24352600 | -1.22659000 |
| H | -3.33774700 | 0.80923100 | -1.29436200 |
| C | -3.12167600 | 0.67428000 | 2.12405600 |
| H | -3.63549900 | 1.46960800 | 1.57583500 |
| H | -2.56304200 | 1.12996400 | 2.94744400 |
| H | -3.85875700 | -0.02914800 | 2.52687800 |
| C | -1.25313000 | -1.50644300 | 2.05520100 |
| H | -0.61949000 | -1.02932300 | 2.81014600 |
| H | -0.63339000 | -2.18297300 | 1.45792500 |
| H | -2.03328700 | -2.08257400 | 2.56450900 |
| C | 0.83291800 | 1.51040900 | 3.13717500 |
| C | 1.42179900 | 0.76664800 | 4.17103900 |
| H | 2.22347900 | 0.06583000 | 3.95828900 |
| C | 0.97962900 | 0.91500400 | 5.48958500 |
| H | 1.44043200 | 0.32852400 | 6.28045400 |
| C | -0.04558100 | 1.81404600 | 5.79150300 |
| H | -0.38593700 | 1.92848000 | 6.81729900 |
| C | -0.63738600 | 2.56121500 | 4.76757500 |
| H | -1.44175400 | 3.25663100 | 4.99298900 |
| C | -0.20922500 | 2.40174900 | 3.44913600 |
| H | -0.69712200 | 2.95188800 | 2.64746900 |
| C | 2.48677900 | -0.04467300 | 1.29934200 |
| H | 1.97514100 | -0.97590500 | 1.56313200 |
| H | 3.35185000 | 0.09365500 | 1.95762500 |
| H | 2.84201300 | -0.12808800 | 0.26660300 |
| C | 2.42416700 | 2.81434900 | 1.12461400 |
| H | 1.83768800 | 3.73674200 | 1.18115000 |
| H | 2.87084900 | 2.75110500 | 0.12724700 |
| H | 3.21752200 | 2.83730700 | 1.87984100 |
| I | -1.56805600 | 3.63130000 | -0.51879800 |

## 6-I-$PMePh_2$

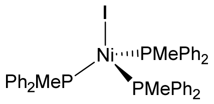


E = -2715.18416817

$G_{corr}$ = 0.58561

| | | | |
|---|---|---|---|
| Ni | 0.77183100 | 0.79556700 | 0.03949200 |
| P | 0.80753700 | 0.85591400 | -2.25606400 |
| C | -0.73225800 | 0.13153800 | -2.96427300 |
| C | -1.86958400 | 0.92691100 | -3.17833600 |
| H | -1.81996300 | 2.00293400 | -3.04201500 |
| C | -3.07996900 | 0.34641000 | -3.56649900 |
| H | -3.95010200 | 0.97711200 | -3.72994600 |
| C | -3.17188300 | -1.03620100 | -3.74701900 |
| H | -4.11407400 | -1.48696800 | -4.04679000 |
| C | -2.04229000 | -1.83481700 | -3.54444600 |
| H | -2.10170200 | -2.91071900 | -3.68719500 |
| C | -0.83281000 | -1.25731500 | -3.15659200 |
| H | 0.03346700 | -1.89137700 | -2.99336500 |
| C | 2.12623800 | -0.15772600 | -3.03843200 |
| C | 2.82698600 | -1.06694800 | -2.23298700 |
| H | 2.60346100 | -1.12769900 | -1.17319300 |
| C | 3.80774000 | -1.89444400 | -2.78586900 |
| H | 4.33557800 | -2.59776300 | -2.14857500 |
| C | 4.10164700 | -1.81383900 | -4.14826700 |
| H | 4.86726000 | -2.45330900 | -4.58002800 |
| C | 3.40761800 | -0.90906000 | -4.95924000 |
| H | 3.63246800 | -0.84615600 | -6.02089300 |
| C | 2.42187100 | -0.08853600 | -4.40998500 |
| H | 1.88017800 | 0.59721400 | -5.05552200 |
| C | 0.94198700 | 2.49319800 | -3.10153700 |
| H | 0.78656700 | 2.42615600 | -4.18275000 |
| H | 1.93987600 | 2.89549600 | -2.90102600 |
| H | 0.20800000 | 3.18389100 | -2.67726600 |
| P | -0.80083700 | 2.33419500 | 0.77010800 |
| C | -1.11560400 | 2.07575800 | 2.56197400 |
| C | -0.12738300 | 2.45055500 | 3.48903300 |

| | | | |
|---|---|---|---|
| H | 0.76617000 | 2.96655600 | 3.14873800 |
| C | -0.28338900 | 2.15943400 | 4.84405600 |
| H | 0.48635500 | 2.45921500 | 5.55024200 |
| C | -1.41985700 | 1.47653700 | 5.29084700 |
| H | -1.53697000 | 1.24391000 | 6.34598200 |
| C | -2.40106700 | 1.09200800 | 4.37444000 |
| H | -3.28401900 | 0.55462300 | 4.71065300 |
| C | -2.25246000 | 1.39024800 | 3.01699800 |
| H | -3.02015200 | 1.07053000 | 2.31977800 |
| C | -0.21380800 | 4.07512300 | 0.67898400 |
| C | -0.93476600 | 5.13050100 | 1.26172900 |
| H | -1.84861800 | 4.92615000 | 1.81372000 |
| C | -0.47645500 | 6.44279100 | 1.14607800 |
| H | -1.03777600 | 7.25394800 | 1.60258900 |
| C | 0.70524000 | 6.71440700 | 0.44663100 |
| H | 1.06064500 | 7.73780600 | 0.35901800 |
| C | 1.42880400 | 5.66993400 | -0.13206400 |
| H | 2.35084000 | 5.87389900 | -0.67008700 |
| C | 0.97243900 | 4.35398900 | -0.01306300 |
| H | 1.54197800 | 3.53571200 | -0.44170200 |
| C | -2.49890700 | 2.48286000 | 0.05334800 |
| H | -3.12140300 | 3.16284400 | 0.64422000 |
| H | -2.98224300 | 1.50498600 | 0.00030900 |
| H | -2.40790100 | 2.87820700 | -0.96193300 |
| P | 0.19163300 | -1.07276100 | 1.26852400 |
| C | 1.02094300 | -2.56613000 | 0.58144500 |
| C | 2.34977100 | -2.84887800 | 0.94144000 |
| H | 2.85660400 | -2.24075300 | 1.68467500 |
| C | 3.03911800 | -3.89985400 | 0.33203200 |
| H | 4.06602400 | -4.10688800 | 0.62192100 |
| C | 2.41603100 | -4.67713500 | -0.64809600 |
| H | 2.95553300 | -5.49117200 | -1.12471300 |
| C | 1.09574700 | -4.40028900 | -1.01287600 |
| H | 0.60206100 | -5.00019500 | -1.77325200 |
| C | 0.40268500 | -3.35217800 | -0.40422500 |
| H | -0.62092400 | -3.14537600 | -0.70148900 |
| C | -1.59326500 | -1.50545800 | 1.26658500 |
| C | -2.35936000 | -1.10658800 | 0.16031500 |
| H | -1.88049100 | -0.57110900 | -0.65303400 |
| C | -3.72869200 | -1.37774500 | 0.10706200 |
| H | -4.30371000 | -1.06682900 | -0.76078100 |
| C | -4.35057300 | -2.03580900 | 1.17119000 |
| H | -5.41800700 | -2.23791000 | 1.13912300 |
| C | -3.59556800 | -2.43462900 | 2.27894700 |
| H | -4.07553100 | -2.94713100 | 3.10864600 |
| C | -2.22364700 | -2.17842800 | 2.32475400 |
| H | -1.65253800 | -2.49597800 | 3.19181500 |
| C | 0.67114600 | -1.10269600 | 3.04895800 |
| H | 0.63805300 | -2.10899900 | 3.47753600 |
| H | 0.00065900 | -0.44258800 | 3.60485300 |
| H | 1.68550300 | -0.70314100 | 3.13386400 |
| I | 3.28360800 | 1.28260500 | 0.98221000 |

**6-I-$PPh_3$**

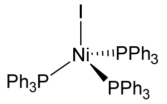


E = -3290.56151807

$G_{corr}$ = 0.735468

| | | | |
|---|---|---|---|
| Ni | 0.65210800 | 1.08207100 | 0.07069000 |
| P | 1.06762700 | 1.08895100 | -2.24545900 |
| C | -0.09917500 | 0.12626300 | -3.29572800 |
| C | -1.41722500 | -0.04978300 | -2.85304800 |
| H | -1.71419600 | 0.33553700 | -1.88643100 |
| C | -2.34323600 | -0.73336300 | -3.64338600 |
| H | -3.36210200 | -0.85646600 | -3.28732700 |
| C | -1.95764100 | -1.25910500 | -4.87865100 |
| H | -2.67699200 | -1.79609000 | -5.49158200 |
| C | -0.64096900 | -1.10029200 | -5.32188100 |
| H | -0.33289800 | -1.51307700 | -6.27902800 |
| C | 0.28480300 | -0.41250700 | -4.53520500 |
| H | 1.30587400 | -0.29664700 | -4.88680100 |
| C | 2.68730900 | 0.35110200 | -2.71217400 |
| C | 2.97978200 | -0.92370400 | -2.19944300 |
| H | 2.26755500 | -1.41771500 | -1.54437600 |
| C | 4.18325700 | -1.55101100 | -2.51858600 |
| H | 4.40116100 | -2.53221600 | -2.10836200 |
| C | 5.11651800 | -0.90629100 | -3.33611800 |
| H | 6.05982900 | -1.39156800 | -3.57230100 |
| C | 4.83524600 | 0.36390700 | -3.84371600 |
| H | 5.55606700 | 0.86969800 | -4.48101100 |
| C | 3.62204000 | 0.98939000 | -3.54015500 |
| H | 3.40824300 | 1.97326500 | -3.94719100 |
| P | -1.02854700 | 2.72145200 | 0.38293300 |
| C | -1.99148500 | 2.58561200 | 1.95748800 |
| C | -2.84358200 | 3.61406900 | 2.39506400 |
| H | -2.94762000 | 4.52399800 | 1.81175800 |
| C | -3.55882200 | 3.47903300 | 3.58467800 |
| H | -4.21280400 | 4.28247900 | 3.91367100 |
| C | -3.43420800 | 2.31626400 | 4.35333000 |
| H | -3.99047900 | 2.21529900 | 5.28159000 |
| C | -2.59275400 | 1.28885400 | 3.92425800 |
| H | -2.48569000 | 0.38229800 | 4.51386400 |
| C | -1.87539600 | 1.42355500 | 2.73195300 |
| H | -1.22038500 | 0.62402400 | 2.40452500 |
| C | -0.47535100 | 4.48437900 | 0.43464500 |
| C | -1.24807100 | 5.56489400 | -0.01858000 |
| H | -2.22889500 | 5.39409700 | -0.45000600 |
| C | -0.75782100 | 6.87023400 | 0.06658700 |
| H | -1.36509900 | 7.69549400 | -0.29594500 |
| C | 0.50499700 | 7.11522100 | 0.61213200 |
| H | 0.88566400 | 8.13139800 | 0.67183500 |
| C | 1.27725000 | 6.04661800 | 1.07644200 |
| H | 2.26535000 | 6.21956600 | 1.49328400 |
| C | 0.78860100 | 4.74318400 | 0.98560700 |
| H | 1.40466800 | 3.91796500 | 1.32333900 |

| | | | |
|---|---|---|---|
| P | 2.76321600 | 1.16772100 | 1.13998300 |
| C | 2.51953200 | 1.18296600 | 2.96962200 |
| C | 1.37166000 | 1.80951800 | 3.47578700 |
| H | 0.65546000 | 2.24586100 | 2.79050000 |
| C | 1.13661100 | 1.87047800 | 4.85002000 |
| H | 0.23310900 | 2.34763700 | 5.21967400 |
| C | 2.05259500 | 1.30113600 | 5.73766800 |
| H | 1.87040500 | 1.33859600 | 6.80843600 |
| C | 3.20253400 | 0.67727200 | 5.24403200 |
| H | 3.91849400 | 0.23327900 | 5.93072500 |
| C | 3.43796100 | 0.61846500 | 3.86898200 |
| H | 4.33456400 | 0.13080300 | 3.49910000 |
| C | 3.78498500 | 2.67723400 | 0.84518900 |
| C | 4.08707400 | 3.59904600 | 1.85947600 |
| H | 3.76279000 | 3.41577800 | 2.87864500 |
| C | 4.80103000 | 4.76462900 | 1.56692200 |
| H | 5.02401200 | 5.46961900 | 2.36364300 |
| C | 5.22952400 | 5.02125500 | 0.26244500 |
| H | 5.78278400 | 5.92914900 | 0.03752200 |
| C | 4.94593400 | 4.10117000 | -0.75086300 |
| H | 5.27620400 | 4.28716900 | -1.76938100 |
| C | 4.22616300 | 2.94166000 | -0.46283700 |
| H | 4.01535700 | 2.23512000 | -1.25868000 |
| C | 3.97233600 | -0.21212100 | 0.92805300 |
| C | 3.49718500 | -1.52124300 | 1.11757700 |
| C | 5.32639000 | -0.02064500 | 0.61940600 |
| C | 4.35738400 | -2.61218200 | 0.99936500 |
| H | 2.45072200 | -1.68756000 | 1.35148100 |
| C | 6.18367800 | -1.11695200 | 0.48928600 |
| H | 5.72271800 | 0.97872300 | 0.47677800 |
| C | 5.70438300 | -2.41401900 | 0.67987100 |
| H | 3.97244300 | -3.61734700 | 1.14996100 |
| H | 7.22929300 | -0.95150500 | 0.24274100 |
| H | 6.37395000 | -3.26430300 | 0.58029900 |
| C | -2.35542400 | 2.69637300 | -0.89541600 |
| C | -2.15345800 | 3.30042000 | -2.14826100 |
| C | -3.52257200 | 1.94400900 | -0.68532600 |
| C | -3.09895300 | 3.15732100 | -3.16419600 |
| H | -1.26045400 | 3.88542500 | -2.33450400 |
| C | -4.46411200 | 1.79892400 | -1.70689100 |
| H | -3.69688400 | 1.46403100 | 0.27279900 |
| C | -4.25551500 | 2.40351700 | -2.94908600 |
| H | -2.92376900 | 3.63123200 | -4.12640100 |
| H | -5.36135500 | 1.21218000 | -1.52825900 |
| H | -4.98794200 | 2.28701600 | -3.74334600 |
| C | 1.09151600 | 2.75940000 | -3.01563700 |
| C | 1.50748000 | 3.82219600 | -2.20117600 |
| C | 0.66929500 | 3.02977600 | -4.32644000 |
| C | 1.50174400 | 5.13364000 | -2.67742600 |
| H | 1.81019000 | 3.62105400 | -1.18158200 |
| C | 0.65644700 | 4.34280300 | -4.80205200 |
| H | 0.33190600 | 2.22372100 | -4.97022500 |
| C | 1.06867100 | 5.39660000 | -3.97929300 |
| H | 1.81161400 | 5.94268700 | -2.02210700 |
| H | 0.31835900 | 4.54385500 | -5.81528600 |
| H | 1.04612200 | 6.41759200 | -4.35111500 |
| I | -0.38680000 | -1.41962600 | 0.48720400 |

**6-Br-PMe$_3$**

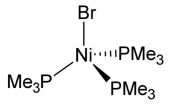


E = -1566.19226774

$G_{corr}$ = 0.287107

| | | | |
|---|---|---|---|
| Ni | 3.03250300 | 7.55801000 | 11.77588700 |
| P | 1.26707200 | 8.91609400 | 11.22903400 |
| C | 0.71294900 | 10.24606300 | 12.38905900 |
| C | -0.30556700 | 7.98865100 | 10.95138800 |
| C | 1.43712400 | 9.86729000 | 9.65241700 |
| H | -0.20052100 | 10.73924000 | 12.03408100 |
| H | 1.50308000 | 10.99816300 | 12.48689900 |
| H | -1.12900900 | 8.65216400 | 10.66040500 |
| H | -0.57256200 | 7.46031800 | 11.87237500 |
| H | 1.60521500 | 9.17079500 | 8.82402100 |
| H | 2.31162200 | 10.52378100 | 9.72105600 |
| P | 4.69473000 | 9.01283400 | 12.41539700 |
| P | 3.87791600 | 6.22789200 | 10.11080600 |
| C | 5.18975900 | 10.40823500 | 11.30993800 |
| C | 6.32322800 | 8.24707400 | 12.82857400 |
| C | 4.29040500 | 9.88914300 | 13.98952400 |
| C | 3.85611800 | 6.93305800 | 8.39969400 |
| C | 5.61548900 | 5.60194400 | 10.20979500 |
| C | 2.93378000 | 4.65832100 | 9.87645600 |
| H | 5.99141400 | 11.01175800 | 11.75214900 |
| H | 4.32400600 | 11.05260800 | 11.12227000 |
| H | 7.00328700 | 8.96836300 | 13.29741600 |
| H | 6.79269500 | 7.85599200 | 11.92056800 |
| H | 4.08020700 | 9.14482000 | 14.76482500 |
| H | 3.39004000 | 10.49700100 | 13.85278900 |
| H | 4.43130600 | 7.86548100 | 8.37817100 |
| H | 2.82389300 | 7.16564700 | 8.11611800 |
| H | 6.31260900 | 6.44377200 | 10.14364900 |
| H | 5.83757700 | 4.89632600 | 9.39968500 |
| H | 2.96854100 | 4.08178800 | 10.80615900 |
| H | 3.33826000 | 4.05644300 | 9.05377700 |
| H | 0.52646300 | 9.81292300 | 13.37745100 |
| H | -0.15283200 | 7.23965100 | 10.16647900 |
| H | 0.54932600 | 10.47422600 | 9.43620700 |
| H | 4.27697300 | 6.23664600 | 7.66368600 |
| H | 5.77052200 | 5.10305800 | 11.17251700 |
| H | 1.88442800 | 4.89440100 | 9.66881100 |
| H | 5.11394400 | 10.53360200 | 14.31962900 |
| H | 5.53176100 | 10.00999600 | 10.34822900 |
| H | 6.15886600 | 7.40893700 | 13.51435400 |
| Br | 2.16865300 | 6.00315200 | 13.55847300 |

**6-Cl-$PMe_3$**

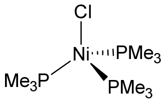


E = -2013.24570877

$G_{corr}$ = 0.288220

| | | | |
|---|---|---|---|
| Ni | 3.13193200 | 7.56706800 | 11.78039300 |
| P | 1.39241000 | 8.89649000 | 11.06173500 |
| C | 0.91569400 | 10.26053500 | 12.21291300 |
| C | -0.20563000 | 7.99951200 | 10.83466200 |
| C | 1.56338700 | 9.80056800 | 9.46056100 |
| H | 0.01767500 | 10.78848200 | 11.87044400 |
| H | 1.74179900 | 10.97619500 | 12.28550900 |
| H | -1.02067300 | 8.67595700 | 10.55071700 |
| H | -0.46854600 | 7.49247600 | 11.76949400 |
| H | 1.63355700 | 9.08155800 | 8.63873500 |
| H | 2.48697200 | 10.38948600 | 9.47418800 |
| P | 4.64697400 | 9.06403100 | 12.63199100 |
| P | 3.69287900 | 6.21567100 | 10.00838200 |
| C | 5.13628800 | 10.51214700 | 11.59046400 |
| C | 6.27965600 | 8.28483100 | 13.00422700 |
| C | 4.25553800 | 9.88272600 | 14.24367100 |
| C | 3.47508900 | 6.80296500 | 8.26818500 |
| C | 5.49320600 | 5.79245600 | 10.03306000 |
| C | 2.92526000 | 4.53645200 | 9.92720000 |
| H | 5.92939100 | 11.10670100 | 12.06012000 |
| H | 4.26603900 | 11.15702500 | 11.42490400 |
| H | 6.97713600 | 8.99130500 | 13.47024000 |
| H | 6.72423600 | 7.90224600 | 12.07950100 |
| H | 4.04560800 | 9.11184800 | 14.99270000 |
| H | 3.35726500 | 10.49937500 | 14.13232600 |
| H | 3.94183800 | 7.78698300 | 8.14992400 |
| H | 2.40666000 | 6.90161000 | 8.04714800 |
| H | 6.08352800 | 6.70420400 | 9.89196600 |
| H | 5.75566500 | 5.07245500 | 9.24812500 |
| H | 3.09691300 | 4.02097700 | 10.87752200 |
| H | 3.33908600 | 3.93654300 | 9.10751300 |
| H | 0.73204800 | 9.84483800 | 13.20932400 |
| H | -0.08544600 | 7.23548700 | 10.05867600 |
| H | 0.71301400 | 10.46817600 | 9.27756300 |
| H | 3.91887800 | 6.10725300 | 7.54562600 |
| H | 5.74667400 | 5.36740100 | 11.00989900 |
| H | 1.84292100 | 4.63718300 | 9.78877000 |
| H | 5.08143400 | 10.51256400 | 14.59650200 |
| H | 5.48712100 | 10.15781700 | 10.61465400 |
| H | 6.11816500 | 7.43771600 | 13.67900700 |
| Cl | 3.04333600 | 5.96742800 | 13.55335800 |

**7-I-$PMe_3$**

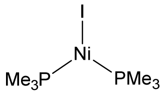


E = -1103.2070108

$G_{corr}$ = 0.179439

| | | | |
|---|---|---|---|
| Ni | 2.97694000 | 7.32161000 | 11.59716800 |
| P | 1.73021300 | 9.18358400 | 11.27788600 |
| P | 2.70143200 | 6.70177000 | 13.75815800 |
| C | 1.70482100 | 10.41968700 | 12.64600500 |
| C | -0.06337500 | 8.85994600 | 10.99769100 |
| C | 2.19514000 | 10.20294200 | 9.81453500 |
| C | 3.65263600 | 7.70893400 | 14.97502300 |
| C | 0.98472800 | 6.78048000 | 14.42808800 |
| C | 3.21436900 | 4.97799400 | 14.16182700 |
| H | 1.07966700 | 11.28397700 | 12.39281700 |
| H | 2.72509900 | 10.76340100 | 12.84689300 |
| H | -0.62405900 | 9.79195600 | 10.86137400 |
| H | -0.47347300 | 8.31492500 | 11.85440400 |
| H | 2.13865400 | 9.58357700 | 8.91309500 |
| H | 3.22875700 | 10.54772200 | 9.92509800 |
| H | 3.32940300 | 8.75349700 | 14.91538100 |
| H | 4.71855300 | 7.66652200 | 14.72699200 |
| H | 0.61255400 | 7.80871700 | 14.36719700 |
| H | 0.95020100 | 6.44977000 | 15.47257900 |
| H | 2.62461700 | 4.27620500 | 13.56246900 |
| H | 3.07226000 | 4.75373200 | 15.22547000 |
| H | 1.31206900 | 9.95248300 | 13.55527300 |
| H | -0.18205900 | 8.23511300 | 10.10619900 |
| H | 1.53561000 | 11.07105500 | 9.69924500 |
| H | 4.26945600 | 4.84342600 | 13.90105200 |
| H | 0.32944100 | 6.14002000 | 13.82804800 |
| H | 3.50701300 | 7.34509900 | 15.99878000 |
| I | 4.39144600 | 6.13458900 | 9.80203900 |

**7-Br-$PMe_3$**

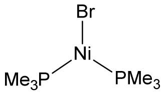


E = -1104.992075

$G_{corr}$ = 0.179718

| | | | |
|---|---|---|---|
| Ni | 3.06750900 | 7.33061100 | 11.59571600 |
| P | 1.74691600 | 9.13568400 | 11.25518400 |
| P | 2.71524600 | 6.74593800 | 13.75887300 |
| C | 1.74732400 | 10.42266800 | 12.57785300 |
| C | -0.04991700 | 8.75641800 | 11.07634700 |
| C | 2.09770600 | 10.12367900 | 9.73841800 |
| C | 3.61912600 | 7.78251000 | 14.98855800 |
| C | 0.97869700 | 6.82627000 | 14.37854000 |
| C | 3.22139600 | 5.03455200 | 14.22185100 |
| H | 1.09164700 | 11.26209700 | 12.31807200 |
| H | 2.76615200 | 10.79705000 | 12.72400900 |

| | | | |
|---|---|---|---|
| H | -0.63894700 | 9.66859100 | 10.92555600 |
| H | -0.40746900 | 8.24202200 | 11.97458600 |
| H | 2.00818700 | 9.47756100 | 8.85860400 |
| H | 3.12519500 | 10.50112900 | 9.78008200 |
| H | 3.30674200 | 8.82693100 | 14.88426500 |
| H | 4.69483900 | 7.72734000 | 14.78979100 |
| H | 0.60383200 | 7.85200200 | 14.29823900 |
| H | 0.91473000 | 6.50504100 | 15.42473400 |
| H | 2.65338700 | 4.31490100 | 13.62245200 |
| H | 3.04688500 | 4.83554700 | 15.28569700 |
| H | 1.40338200 | 9.98270000 | 13.51978600 |
| H | -0.19563400 | 8.08955500 | 10.21980800 |
| H | 1.40658800 | 10.96868500 | 9.63695700 |
| H | 4.28453600 | 4.89796700 | 13.99716200 |
| H | 0.34253200 | 6.17763100 | 13.76685600 |
| H | 3.42509900 | 7.45088900 | 16.01528300 |
| Br | 4.53599100 | 6.21555900 | 10.09098800 |

### 7-Cl-PMe$_3$

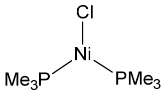


E = -1552.04429788

$G_{corr}$ = 0.182396

| | | | |
|---|---|---|---|
| Ni | 3.06690600 | 7.31937300 | 11.59599400 |
| P | 1.74836800 | 9.13061000 | 11.26473000 |
| P | 2.71674300 | 6.73966600 | 13.75863800 |
| C | 1.75110000 | 10.42370600 | 12.58137900 |
| C | -0.04942500 | 8.75621200 | 11.08430600 |
| C | 2.10425400 | 10.11132300 | 9.74422700 |
| C | 3.61372800 | 7.78534000 | 14.98584800 |
| C | 0.97752200 | 6.82259700 | 14.37053800 |
| C | 3.22257200 | 5.03196700 | 14.23742400 |
| H | 1.09763500 | 11.26343500 | 12.31708900 |
| H | 2.77060800 | 10.79661300 | 12.72617400 |
| H | -0.63554900 | 9.66943900 | 10.92877800 |
| H | -0.41092700 | 8.24613200 | 11.98340600 |
| H | 2.01797900 | 9.46003300 | 8.86785500 |
| H | 3.13183700 | 10.48837100 | 9.78745900 |
| H | 3.30995100 | 8.83052900 | 14.86563400 |
| H | 4.69117300 | 7.72105900 | 14.79995800 |
| H | 0.60416200 | 7.84876200 | 14.28846800 |
| H | 0.90943800 | 6.50226200 | 15.41673000 |
| H | 2.65683600 | 4.30686900 | 13.64236600 |
| H | 3.04474700 | 4.84114800 | 15.30220000 |
| H | 1.40565700 | 9.98978900 | 13.52562700 |
| H | -0.19540500 | 8.08665500 | 10.22995400 |
| H | 1.41414600 | 10.95618100 | 9.63525300 |
| H | 4.28681600 | 4.89428500 | 14.01789200 |
| H | 0.34264000 | 6.17451400 | 13.75709700 |
| H | 3.40581100 | 7.46753900 | 16.01420800 |
| Cl | 4.40937900 | 6.34932800 | 10.08462500 |

### 8-Br-PMe$_3$

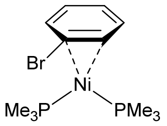


E = -1336.64535174

$G_{corr}$ = 0.267601

| | | | |
|---|---|---|---|
| Ni | -2.51527500 | 1.80090200 | -0.03276900 |
| P | -4.38851900 | 1.48763400 | -1.18364500 |
| C | -5.42814700 | 0.19073800 | -0.37829600 |
| C | -4.42043900 | 0.95404800 | -2.95305400 |
| C | -5.53034900 | 2.93934200 | -1.21686000 |
| H | -6.41317600 | 0.10190500 | -0.85156400 |
| H | -5.55218000 | 0.44260700 | 0.68028500 |
| H | -5.44824200 | 0.86884900 | -3.32584000 |
| H | -3.92640700 | -0.01728000 | -3.05528200 |
| H | -5.04366800 | 3.77575200 | -1.73080100 |
| H | -5.74682800 | 3.24839400 | -0.18913800 |
| P | -0.74211000 | 1.01423100 | -1.07728500 |
| C | 0.28074000 | 2.30221200 | -1.91782500 |
| C | 0.50677000 | 0.16478500 | -0.01508200 |
| C | -0.97523200 | -0.22175500 | -2.43164100 |
| H | 0.59936000 | 3.04152600 | -1.17650700 |
| H | -0.32606200 | 2.81174100 | -2.67435900 |
| H | 0.84549400 | 0.85788600 | 0.76098000 |
| H | 1.37125500 | -0.16925600 | -0.60104700 |
| H | -1.55022300 | 0.23092300 | -3.24509000 |
| H | -1.53560100 | -1.08148600 | -2.04845000 |
| H | -4.91174100 | -0.77349200 | -0.43968200 |
| H | -3.88047200 | 1.68260200 | -3.56781700 |
| H | -6.47074300 | 2.70394900 | -1.72917700 |
| H | 1.16291800 | 1.86504700 | -2.40074200 |
| H | 0.04842400 | -0.70315200 | 0.47080400 |
| H | -0.01359900 | -0.57019400 | -2.82716000 |
| C | -1.89536400 | 2.30945900 | 1.70060600 |
| C | -3.20549600 | 2.91896000 | 1.56358300 |
| C | -4.27868400 | 2.46159500 | 2.39853800 |
| C | -4.11752400 | 1.38229200 | 3.23408300 |
| C | -2.84792000 | 0.73501200 | 3.31437900 |
| C | -1.76043100 | 1.20286900 | 2.61272000 |
| H | -3.29064200 | 3.91571200 | 1.13276200 |
| H | -5.22225000 | 3.00345500 | 2.37495700 |
| H | -4.93930000 | 1.03241300 | 3.85335300 |
| H | -2.72962000 | -0.13192800 | 3.96092500 |
| H | -0.78725900 | 0.73589600 | 2.72885000 |
| Br | -0.24806100 | 3.61851900 | 1.78727200 |

**8-Cl-$PMe_3$**

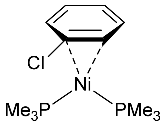


E = -1783.69729748

$G_{corr}$ = 0.268563

| | | | |
|---|---|---|---|
| Ni | -0.77264000 | 0.67024800 | -0.11353400 |
| P | -2.62002100 | 0.41448600 | -1.30633200 |
| C | -3.85748200 | -0.68813000 | -0.48957900 |
| C | -2.60606000 | -0.27437400 | -3.02303900 |
| C | -3.59831700 | 1.96390000 | -1.55118500 |
| H | -4.81212500 | -0.69266500 | -1.02898500 |
| H | -4.01944000 | -0.34904800 | 0.53838900 |
| H | -3.61853000 | -0.32332700 | -3.44209400 |
| H | -2.18007700 | -1.28272300 | -3.01082600 |
| H | -2.99298100 | 2.69954900 | -2.09219100 |
| H | -3.85641200 | 2.38761000 | -0.57498400 |
| P | 1.03005200 | -0.04754100 | -1.15728700 |
| C | 1.98296500 | 1.30675800 | -1.97944100 |
| C | 2.33378800 | -0.84511100 | -0.11922200 |
| C | 0.86900000 | -1.26924700 | -2.53617800 |
| H | 2.25451000 | 2.05616400 | -1.22951300 |
| H | 1.35156300 | 1.78860200 | -2.73419800 |
| H | 2.65430300 | -0.14217600 | 0.65568100 |
| H | 3.20288000 | -1.13792300 | -0.72039500 |
| H | 0.25954100 | -0.84058000 | -3.33749500 |
| H | 0.36964100 | -2.17283900 | -2.16964600 |
| H | -3.46427800 | -1.71004700 | -0.45014100 |
| H | -1.98564300 | 0.35603700 | -3.66933000 |
| H | -4.51969000 | 1.77559000 | -2.11529400 |
| H | 2.89234300 | 0.92832900 | -2.46195400 |
| H | 1.92253600 | -1.73473500 | 0.36951400 |
| H | 1.84848600 | -1.54580500 | -2.94432900 |
| C | -0.11382200 | 1.21953900 | 1.61916100 |
| C | -1.46586700 | 1.74168200 | 1.49147700 |
| C | -2.49964500 | 1.19115500 | 2.32480200 |
| C | -2.26171000 | 0.10997300 | 3.13673200 |
| C | -0.94586700 | -0.44362300 | 3.21152500 |
| C | 0.10363200 | 0.11840500 | 2.52555900 |
| H | -1.61106800 | 2.74967900 | 1.10419300 |
| H | -3.47989100 | 1.66371800 | 2.31171400 |
| H | -3.05641900 | -0.31055300 | 3.74741200 |
| H | -0.76369300 | -1.30599900 | 3.84910900 |
| H | 1.11229600 | -0.26592100 | 2.64325800 |
| Cl | 1.29374000 | 2.45807500 | 1.59130600 |

**TS-8-9-Br-$PMe_3$**

E = -1336.64193985

$G_{corr}$ = 0.268325

ν = -85.30 $cm^{-1}$

| | | | |
|---|---|---|---|
| Ni | -2.60106300 | 1.65679100 | 0.00721300 |
| P | -4.43035800 | 1.38936200 | -1.25685000 |
| C | -5.67221000 | 0.32762000 | -0.40282800 |
| C | -4.41498800 | 0.68646800 | -2.96083100 |
| C | -5.34476000 | 2.97279200 | -1.49828700 |
| H | -6.62787000 | 0.31574300 | -0.93969400 |
| H | -5.82724300 | 0.70560800 | 0.61310700 |
| H | -5.42976200 | 0.64071500 | -3.37322800 |
| H | -3.99472300 | -0.32379600 | -2.94164500 |
| H | -4.72600500 | 3.66909800 | -2.07467900 |
| H | -5.55127000 | 3.42468200 | -0.52272900 |
| P | -0.79349100 | 1.05740800 | -1.08144400 |
| C | 0.09006700 | 2.48410400 | -1.83706700 |
| C | 0.49552800 | 0.26730800 | -0.03497900 |
| C | -0.94246200 | -0.12575500 | -2.48744000 |
| H | 0.32035600 | 3.20423200 | -1.04622900 |
| H | -0.55868300 | 2.96591800 | -2.57671800 |
| H | 0.75004800 | 0.95431600 | 0.77722100 |
| H | 1.39427800 | 0.04030800 | -0.61995700 |
| H | -1.55800300 | 0.31103100 | -3.27890800 |
| H | -1.42172200 | -1.04826700 | -2.14277300 |
| H | -5.28819200 | -0.69581500 | -0.33083700 |
| H | -3.79535600 | 1.31120700 | -3.61263500 |
| H | -6.29011900 | 2.81036300 | -2.02875400 |
| H | 1.01741600 | 2.16198800 | -2.32530800 |
| H | 0.10302600 | -0.66114000 | 0.39283400 |
| H | 0.04413900 | -0.37003500 | -2.89799200 |
| C | -2.05804200 | 2.02438900 | 1.71394600 |
| C | -3.29554500 | 2.74234100 | 1.69264100 |
| C | -4.30822000 | 2.40404500 | 2.63487700 |
| C | -4.12055200 | 1.35653700 | 3.51744900 |
| C | -2.89924300 | 0.63829800 | 3.51313500 |
| C | -1.86780200 | 0.98055500 | 2.65368600 |
| H | -3.37806200 | 3.69102400 | 1.16416100 |
| H | -5.21167500 | 3.00854600 | 2.68189900 |
| H | -4.89704300 | 1.09177600 | 4.23012300 |
| H | -2.76978600 | -0.20198800 | 4.19198100 |
| H | -0.92739600 | 0.43926400 | 2.67196600 |
| Br | -0.07981500 | 3.56567100 | 1.89055600 |

**TS-8-9-Cl-$PMe_3$**

E = -1783.69289962

$G_{corr}$ = 0.269087

ν = -104.13 $cm^{-1}$

| | | | |
|---|---|---|---|
| Ni | -0.80924900 | 0.56031400 | -0.12550700 |
| P | -2.62462800 | 0.31269600 | -1.41950500 |
| C | -3.87651500 | -0.74462300 | -0.57466800 |
| C | -2.59668600 | -0.38744400 | -3.12368600 |
| C | -3.52644800 | 1.90271600 | -1.66166200 |
| H | -4.82870800 | -0.75306200 | -1.11777200 |
| H | -4.03689400 | -0.36607800 | 0.44028600 |
| H | -3.60905400 | -0.43552300 | -3.54164000 |
| H | -2.17372500 | -1.39652500 | -3.10276600 |
| H | -2.89955300 | 2.59644500 | -2.23223200 |

| | | | |
|---|---|---|---|
| H | -3.73523500 | 2.35284500 | -0.68570900 |
| P | 1.01236900 | -0.01240000 | -1.19417700 |
| C | 1.88322300 | 1.43784500 | -1.91600300 |
| C | 2.29460600 | -0.80464900 | -0.14344800 |
| C | 0.88123500 | -1.17463300 | -2.61848000 |
| H | 2.09870700 | 2.14004200 | -1.10542100 |
| H | 1.23283200 | 1.92717600 | -2.64912600 |
| H | 2.54061600 | -0.11599400 | 0.66969900 |
| H | 3.19637700 | -1.02920100 | -0.72468500 |
| H | 0.26698400 | -0.73054600 | -3.40681800 |
| H | 0.40886000 | -2.10731100 | -2.29229400 |
| H | -3.49610100 | -1.76919200 | -0.50014600 |
| H | -1.97534700 | 0.23979600 | -3.77135500 |
| H | -4.46998900 | 1.74813900 | -2.19765000 |
| H | 2.81651300 | 1.13513000 | -2.40511100 |
| H | 1.90088700 | -1.73443700 | 0.28017100 |
| H | 1.87296300 | -1.40251600 | -3.02621100 |
| C | -0.26389700 | 0.89777900 | 1.57388900 |
| C | -1.48043000 | 1.64497900 | 1.58429900 |
| C | -2.46202600 | 1.35060100 | 2.57063200 |
| C | -2.25290000 | 0.32877100 | 3.47975100 |
| C | -1.04186200 | -0.40352200 | 3.45485700 |
| C | -0.04191400 | -0.10999700 | 2.54017200 |
| H | -1.57265600 | 2.57271800 | 1.02021100 |
| H | -3.35556200 | 1.96828700 | 2.63128500 |
| H | -3.00507000 | 0.09778900 | 4.22923400 |
| H | -0.89647800 | -1.22104300 | 4.15783900 |
| H | 0.88930700 | -0.66726300 | 2.53491200 |
| Cl | 1.67504400 | 2.36902100 | 1.66149600 |

## 9-I-$PMe_3$

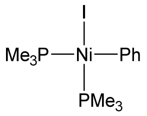


E = -1334.70742817

$G_{corr}$ = 0.268779

| | | | |
|---|---|---|---|
| Ni | 2.37440000 | 3.95863200 | 5.57007600 |
| P | 1.01244700 | 5.02022200 | 4.03544600 |
| C | 0.13548000 | 6.39191800 | 4.90358800 |
| C | -0.34992600 | 4.06672700 | 3.24739600 |
| C | 1.71661700 | 5.93965900 | 2.59506000 |
| H | -0.47430500 | 6.96501300 | 4.19612800 |
| H | 0.87311500 | 7.05837400 | 5.36408400 |
| H | -0.98519700 | 4.72020300 | 2.63856400 |
| H | -0.95502500 | 3.58966100 | 4.02361100 |
| H | 2.00331700 | 5.25135900 | 1.79738800 |
| H | 2.59675300 | 6.51204500 | 2.90619900 |
| P | 4.10747000 | 3.35992700 | 4.35495700 |
| C | 3.95981600 | 3.41180600 | 2.51789900 |
| C | 5.72806700 | 4.18411300 | 4.62391500 |
| C | 4.46099000 | 1.57603100 | 4.63198100 |
| H | 4.02651100 | 4.44136800 | 2.16033500 |
| H | 3.00530800 | 2.97906600 | 2.20368500 |
| H | 5.63172100 | 5.25719500 | 4.42790800 |
| H | 6.47646000 | 3.76275800 | 3.94347900 |
| H | 4.82627900 | 1.42293400 | 5.64899000 |
| H | 5.21243100 | 1.22333900 | 3.91729300 |
| H | -0.50247200 | 5.98773800 | 5.69272000 |
| H | 0.07494800 | 3.28474900 | 2.60871700 |
| H | 0.96615400 | 6.63378100 | 2.20016500 |
| H | 4.77631600 | 2.83345100 | 2.07112500 |
| H | 6.05335200 | 4.04509100 | 5.65695400 |
| H | 3.53901100 | 1.00006200 | 4.50172000 |
| C | 3.54282700 | 3.67092200 | 7.05768900 |
| C | 3.74568300 | 2.49242100 | 7.79311500 |
| C | 4.53579300 | 2.48287000 | 8.94715900 |
| C | 5.13508200 | 3.66278200 | 9.40098800 |
| C | 4.94125100 | 4.84782500 | 8.68616900 |
| C | 4.16416300 | 4.84416500 | 7.52089500 |
| H | 3.26352900 | 1.57227600 | 7.47281100 |
| H | 4.67414000 | 1.55561800 | 9.49972900 |
| H | 5.74449000 | 3.65805600 | 10.30149400 |
| H | 5.40176900 | 5.77283800 | 9.02759100 |
| H | 4.04627800 | 5.77341900 | 6.96355900 |
| I | 0.27331600 | 3.31344500 | 7.08271300 |

## 9-Br-$PMe_3$

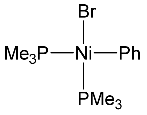


E = -1336.69800763

$G_{corr}$ = 0.271175

| | | | |
|---|---|---|---|
| Ni | -3.52707500 | 1.70041900 | 0.12845700 |
| P | -4.53274900 | 1.68518200 | -1.96134900 |
| C | -6.32780600 | 1.27132300 | -1.93998200 |
| C | -3.96597700 | 0.51692500 | -3.27871500 |
| C | -4.49898100 | 3.32033400 | -2.80758700 |
| H | -6.72635800 | 1.26545000 | -2.96082100 |
| H | -6.86607800 | 2.00207000 | -1.33354800 |
| H | -4.55048300 | 0.67072000 | -4.19293900 |
| H | -4.12263800 | -0.51060100 | -2.93353700 |
| H | -3.46455100 | 3.60310800 | -3.02845100 |
| H | -4.92536800 | 4.07113300 | -2.13466300 |
| P | -1.48573000 | 1.23291500 | -0.55457100 |
| C | -0.92567800 | 2.24031900 | -1.99401000 |
| C | -0.07656800 | 1.49765000 | 0.59744200 |
| C | -1.24035400 | -0.51828400 | -1.05826300 |
| H | -0.92212700 | 3.29479100 | -1.69811700 |
| H | -1.58704500 | 2.12698500 | -2.85424500 |
| H | -0.08743300 | 2.52704600 | 0.96616000 |
| H | 0.86494700 | 1.31035100 | 0.06883000 |
| H | -1.94179400 | -0.79260700 | -1.84966400 |
| H | -1.42887200 | -1.15790900 | -0.18974300 |
| H | -6.46977700 | 0.28261300 | -1.49088100 |
| H | -2.90695000 | 0.64543500 | -3.51482900 |
| H | -5.07397000 | 3.30019100 | -3.74038400 |

| | | | |
|---|---|---|---|
| H | 0.09047200 | 1.95098500 | -2.28487300 |
| H | -0.15631500 | 0.82236800 | 1.45203100 |
| H | -0.21468400 | -0.68257500 | -1.40693500 |
| C | -2.86027200 | 1.38547500 | 1.88710500 |
| C | -2.42448100 | 2.38335800 | 2.77399600 |
| C | -1.99047800 | 2.06959500 | 4.06501500 |
| C | -1.98751600 | 0.74050000 | 4.50530300 |
| C | -2.42208600 | -0.26661800 | 3.63999300 |
| C | -2.84608400 | 0.05664800 | 2.34376700 |
| H | -2.43524700 | 3.42245900 | 2.45338300 |
| H | -1.65832100 | 2.86267500 | 4.73269400 |
| H | -1.65129800 | 0.49487100 | 5.50991700 |
| H | -2.42715300 | -1.30415300 | 3.96929700 |
| H | -3.16539300 | -0.74504200 | 1.67834600 |
| Br | -5.46833100 | 2.83660700 | 1.08135400 |

## 9-Cl-$PMe_3$

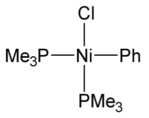


| | | |
|---|---|---|
| E | = | -1783.75121852 |
| $G_{corr}$ | = | 0.270617 |

| | | | |
|---|---|---|---|
| Ni | -1.66158000 | 0.51749800 | -0.01575800 |
| P | -2.30701100 | 1.54735800 | -2.00573100 |
| C | -4.08047500 | 1.31200400 | -2.44171000 |
| C | -1.52814700 | 1.35978300 | -3.67301600 |
| C | -2.15312400 | 3.36519500 | -1.74458700 |
| H | -4.36201600 | 1.95000900 | -3.28712100 |
| H | -4.69830600 | 1.55097700 | -1.57331700 |
| H | -1.93007300 | 2.12187500 | -4.35053200 |
| H | -1.76065200 | 0.37627600 | -4.09193800 |
| H | -1.09764900 | 3.62182300 | -1.60012600 |
| H | -2.70278700 | 3.64094400 | -0.84116300 |
| P | 0.06814000 | -0.59646800 | -0.78501100 |
| C | -0.25808500 | -1.67144600 | -2.24476100 |
| C | 1.41503500 | 0.53898200 | -1.31709500 |
| C | 0.97016900 | -1.77457600 | 0.30184400 |
| H | -0.64348800 | -1.09464500 | -3.08592800 |
| H | -1.00441400 | -2.42349000 | -1.96741100 |
| H | 1.04172500 | 1.25541700 | -2.05261600 |
| H | 2.25247900 | -0.02224600 | -1.74669400 |
| H | 0.28983000 | -2.55781000 | 0.64690900 |
| H | 1.36588700 | -1.25486600 | 1.17708300 |
| H | -4.25032800 | 0.26394300 | -2.71238400 |
| H | -0.44204300 | 1.47641000 | -3.62466000 |
| H | -2.54500700 | 3.92261200 | -2.60290600 |
| H | 0.66335200 | -2.17943800 | -2.55081500 |
| H | 1.76743800 | 1.09601800 | -0.44257900 |
| H | 1.79359300 | -2.22822100 | -0.26072600 |
| C | -1.11421900 | -0.05512300 | 1.71575000 |
| C | -1.64229100 | -1.20161900 | 2.33062500 |
| C | -1.25877000 | -1.57575700 | 3.62297900 |
| C | -0.33041100 | -0.80674200 | 4.33251900 |
| C | 0.20438500 | 0.33980800 | 3.73776700 |
| C | -0.18425200 | 0.70618000 | 2.44326900 |
| H | -2.36473700 | -1.81380500 | 1.79358500 |
| H | -1.68410200 | -2.46893400 | 4.07731500 |
| H | -0.02854500 | -1.09726400 | 5.33606100 |
| H | 0.92565100 | 0.94858100 | 4.28021200 |
| H | 0.25068000 | 1.59778400 | 1.99309300 |
| Cl | -3.47625700 | 1.53010200 | 0.95223600 |

## TS-9-3-I-$PMe_3$

| | | |
|---|---|---|
| E | = | -1334.89533628 |
| $G_{corr}$ | = | 0.265005 |
| ν | = | -88.03 $cm^{-1}$ |

| | | | |
|---|---|---|---|
| Ni | -1.30498100 | 0.12127600 | 0.00974500 |
| P | -2.55981600 | 1.85759200 | -0.96668600 |
| C | -3.50474300 | 2.75682700 | 0.33863900 |
| C | -3.86857500 | 1.45652700 | -2.20150000 |
| C | -1.63704600 | 3.22647900 | -1.79412800 |
| H | -4.08081900 | 3.58892600 | -0.08302700 |
| H | -2.80818100 | 3.14409900 | 1.09010700 |
| H | -4.41774000 | 2.35269400 | -2.51393200 |
| H | -4.56679700 | 0.73834600 | -1.75962400 |
| H | -1.12426000 | 2.84186300 | -2.68257900 |
| H | -0.88343200 | 3.62882400 | -1.10828700 |
| P | 0.49890900 | -0.48730700 | -0.96608300 |
| C | -0.01624600 | -0.95849900 | -2.66636700 |
| C | 1.81787600 | 0.76136100 | -1.21863300 |
| C | 1.37052500 | -1.96769200 | -0.33022300 |
| H | -0.44193000 | -0.08849800 | -3.17569900 |
| H | -0.78242100 | -1.73703700 | -2.60445200 |
| H | 1.38624400 | 1.65414800 | -1.68237200 |
| H | 2.60357900 | 0.35953800 | -1.86796200 |
| H | 1.81275500 | -1.74305300 | 0.64412900 |
| H | 2.15689200 | -2.27781800 | -1.02707000 |
| H | -4.18707400 | 2.05746200 | 0.83295700 |
| H | -3.41409300 | 0.98891500 | -3.08171300 |
| H | -2.30803800 | 4.03867100 | -2.09876700 |
| H | 0.84195100 | -1.33018600 | -3.23813900 |
| H | 2.25033000 | 1.04067700 | -0.25413800 |
| H | 0.64828300 | -2.78085300 | -0.20896900 |
| C | -0.21023300 | 0.38033000 | 1.48376800 |
| C | -0.12258300 | -0.59811600 | 2.48617700 |
| C | 0.37255900 | -0.26586100 | 3.75185500 |
| C | 0.81192100 | 1.03529500 | 4.01787100 |
| C | 0.75835100 | 2.00583300 | 3.01269400 |
| C | 0.26516000 | 1.67560600 | 1.74506500 |
| H | -0.46111800 | -1.60940900 | 2.28111500 |
| H | 0.41706700 | -1.02564300 | 4.52901300 |
| H | 1.20103100 | 1.28940300 | 5.00058300 |
| H | 1.10710000 | 3.01703300 | 3.21063800 |
| H | 0.23973600 | 2.42962100 | 0.96181300 |
| I | -3.06747900 | -1.97892200 | 0.15291800 |

**TS-9-3-Br-PMe$_3$**

E = -1336.67631292

$G_{corr}$ = 0.267521

ν = -84.67 $cm^{-1}$

| | | | |
|---|---|---|---|
| Ni | 0.71481000 | 1.22553000 | -0.03001400 |
| P | -0.48274500 | 3.04627000 | -0.97831300 |
| C | -1.43605900 | 3.86803000 | 0.37238000 |
| C | -1.79203400 | 2.73018900 | -2.23942500 |
| C | 0.43314200 | 4.47372200 | -1.71463700 |
| H | -1.99258300 | 4.73828700 | 0.00432300 |
| H | -0.74611400 | 4.18705700 | 1.16113300 |
| H | -2.36664500 | 3.63650700 | -2.46558400 |
| H | -2.46880000 | 1.95688300 | -1.86152800 |
| H | 0.95310000 | 4.15027800 | -2.62328100 |
| H | 1.18002400 | 4.83812400 | -1.00043600 |
| P | 2.55352300 | 0.68320500 | -0.99142800 |
| C | 2.07818300 | 0.17636500 | -2.69552300 |
| C | 3.85228400 | 1.95522600 | -1.24535800 |
| C | 3.46790400 | -0.76434000 | -0.33444100 |
| H | 1.63524700 | 1.02841400 | -3.22081700 |
| H | 1.33202600 | -0.62227400 | -2.63956000 |
| H | 3.41084000 | 2.83675000 | -1.72116700 |
| H | 4.65372200 | 1.56320600 | -1.88157800 |
| H | 3.86903200 | -0.52168200 | 0.65353600 |
| H | 4.28885400 | -1.04096500 | -1.00519500 |
| H | -2.13681500 | 3.14760800 | 0.80730900 |
| H | -1.33223900 | 2.35883500 | -3.16211700 |
| H | -0.24207700 | 5.29949800 | -1.97049200 |
| H | 2.95228900 | -0.17970200 | -3.25290600 |
| H | 4.26977000 | 2.25072600 | -0.27888100 |
| H | 2.77825400 | -1.60817000 | -0.23344800 |
| C | 1.78582400 | 1.56724300 | 1.44928400 |
| C | 1.89417200 | 0.62190200 | 2.48113600 |
| C | 2.41474600 | 0.99301400 | 3.72550100 |
| C | 2.85479000 | 2.30303000 | 3.94479800 |
| C | 2.77585200 | 3.24220600 | 2.91225300 |
| C | 2.25860000 | 2.87139400 | 1.66522300 |
| H | 1.54544100 | -0.39262500 | 2.31388300 |
| H | 2.47598300 | 0.25731700 | 4.52466200 |
| H | 3.26216600 | 2.58759100 | 4.91169100 |
| H | 3.12375300 | 4.26058900 | 3.07196300 |
| H | 2.21411400 | 3.60036800 | 0.85929000 |
| Br | -1.02463200 | -0.51900700 | 0.31646300 |

**TS-9-3-Cl-PMe$_3$**

E = -1783.72865959

$G_{corr}$ = 0.268487

ν = -88.55 $cm^{-1}$

| | | | |
|---|---|---|---|
| Ni | -0.46345900 | 1.07262600 | -0.11445600 |
| P | -1.64277900 | 2.96437400 | -0.98048100 |
| C | -2.43743500 | 3.79902700 | 0.46244500 |
| C | -3.06930000 | 2.73735600 | -2.13063400 |
| C | -0.73396700 | 4.36403600 | -1.77616600 |
| H | -2.99353200 | 4.69559700 | 0.16325900 |
| H | -1.66705800 | 4.07639600 | 1.18972200 |
| H | -3.61427400 | 3.67491400 | -2.29368500 |
| H | -3.75377800 | 1.99244000 | -1.71155100 |
| H | -0.30145700 | 4.02943400 | -2.72578300 |
| H | 0.08218000 | 4.69493800 | -1.12454700 |
| P | 1.42783300 | 0.58471500 | -1.01476000 |
| C | 1.01142800 | 0.02274900 | -2.71781500 |
| C | 2.67583000 | 1.90731600 | -1.26916000 |
| C | 2.39569400 | -0.80108000 | -0.30271100 |
| H | 0.52911500 | 0.84145300 | -3.26176600 |
| H | 0.30915100 | -0.81494400 | -2.65949700 |
| H | 2.20776100 | 2.76421100 | -1.76405500 |
| H | 3.50144200 | 1.53853400 | -1.88815600 |
| H | 2.76119500 | -0.51452100 | 0.68758400 |
| H | 3.24529300 | -1.05633600 | -0.94580400 |
| H | -3.12120500 | 3.09414500 | 0.94730000 |
| H | -2.70771400 | 2.36253400 | -3.09479200 |
| H | -1.39559900 | 5.21675400 | -1.97225700 |
| H | 1.91154300 | -0.29267700 | -3.25791200 |
| H | 3.06925900 | 2.23205800 | -0.30192300 |
| H | 1.74445100 | -1.67410300 | -0.19336700 |
| C | 0.53808000 | 1.47616000 | 1.39532500 |
| C | 0.61168700 | 0.54703000 | 2.44488500 |
| C | 1.09468400 | 0.93620000 | 3.69919300 |
| C | 1.53292000 | 2.24747900 | 3.91203700 |
| C | 1.49146500 | 3.16910300 | 2.86180400 |
| C | 1.00994900 | 2.78106400 | 1.60599400 |
| H | 0.27003800 | -0.47133900 | 2.28502000 |
| H | 1.12998800 | 0.21166100 | 4.50999600 |
| H | 1.91073800 | 2.54603500 | 4.88664400 |
| H | 1.83802400 | 4.18884900 | 3.01542500 |
| H | 0.99451800 | 3.49789200 | 0.78876300 |
| Cl | -2.15901000 | -0.48711900 | 0.26577700 |

## In Toluene

## $Ni(PMe_3)_4$

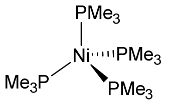


E = -2014.13708291

$G_{corr}$ = 0.403970

| | | | |
|---|---|---|---|
| Ni | 2.97381100 | 7.50344000 | 11.85409900 |
| P | 1.37483900 | 8.84210700 | 11.13366300 |
| P | 2.20722400 | 6.27725200 | 13.51149600 |
| C | 0.88536800 | 10.24856200 | 12.25337300 |
| C | -0.31697100 | 8.16396700 | 10.75098000 |
| C | 1.64143500 | 9.81555100 | 9.56776600 |
| C | 2.55556300 | 6.82503300 | 15.25803600 |
| C | 0.36860200 | 6.01592500 | 13.67682000 |
| C | 2.74379500 | 4.49927900 | 13.67361100 |
| H | 0.05074000 | 10.83875800 | 11.85235300 |
| H | 1.74639700 | 10.91117200 | 12.39388100 |
| H | -0.99515300 | 8.92925400 | 10.35015700 |
| H | -0.76077600 | 7.74282600 | 11.65808900 |
| H | 1.69439100 | 9.13211500 | 8.71510100 |
| H | 2.59567600 | 10.35007200 | 9.62535100 |
| H | 2.18758700 | 7.84642100 | 15.40103200 |
| H | 3.63692500 | 6.82627000 | 15.43416100 |
| H | -0.12338400 | 6.97858500 | 13.85513400 |
| H | 0.11669700 | 5.33787300 | 14.50319500 |
| H | 2.36144500 | 3.92489400 | 12.82404700 |
| H | 2.37823100 | 4.03591700 | 14.59981200 |
| P | 4.57278300 | 8.84210700 | 12.57453600 |
| P | 3.74039800 | 6.27725100 | 10.19670300 |
| C | 5.06225400 | 10.24856200 | 11.45482600 |
| C | 6.26459200 | 8.16396600 | 12.95721900 |
| C | 4.30618600 | 9.81555100 | 14.14043300 |
| C | 3.39205800 | 6.82503300 | 8.45016300 |
| C | 5.57901900 | 6.01592500 | 10.03137800 |
| C | 3.20382600 | 4.49927900 | 10.03458800 |
| H | 5.89688200 | 10.83875700 | 11.85584600 |
| H | 4.20122600 | 10.91117200 | 11.31431800 |
| H | 6.94277500 | 8.92925400 | 13.35804300 |
| H | 6.70839800 | 7.74282500 | 12.05011000 |
| H | 4.25323000 | 9.13211500 | 14.99309800 |
| H | 3.35194600 | 10.35007200 | 14.08284800 |
| H | 3.76003400 | 7.84642100 | 8.30716700 |
| H | 2.31069700 | 6.82627000 | 8.27403800 |
| H | 6.07100500 | 6.97858500 | 9.85306500 |
| H | 5.83092400 | 5.33787300 | 9.20500400 |
| H | 3.58617600 | 3.92489400 | 10.88415200 |
| H | 3.56939100 | 4.03591700 | 9.10838700 |
| H | 0.60305900 | 9.85393000 | 13.23550800 |
| H | -0.22469800 | 7.35334000 | 10.01912300 |
| H | 0.83650600 | 10.53989700 | 9.38505300 |
| H | 3.86490100 | 6.17035700 | 7.70594000 |
| H | 5.97358000 | 5.60061000 | 10.96487800 |
| H | 2.10969600 | 4.44342100 | 10.05054800 |
| H | 3.83792600 | 4.44342100 | 13.65765100 |
| H | -0.02595900 | 5.60061000 | 12.74332100 |
| H | 2.08272000 | 6.17035800 | 16.00225900 |
| H | 5.11111600 | 10.53989700 | 14.32314600 |
| H | 5.34456300 | 9.85392900 | 10.47269100 |
| H | 6.17231900 | 7.35333900 | 13.68907600 |

## $Ni(PMe_3)_3$

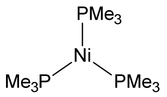


E = -1552.92426336

$G_{corr}$ = 0.294185

| | | | |
|---|---|---|---|
| Ni | -0.37837600 | 1.29416200 | -0.03109900 |
| P | -1.59146500 | 2.98753900 | -0.59201400 |
| P | -0.61818900 | 0.48827300 | 1.95583800 |
| C | -1.65730000 | 4.43136800 | 0.58038000 |
| C | -3.40670900 | 2.62804300 | -0.77096400 |
| C | -1.29768300 | 3.90778500 | -2.18441500 |
| C | 0.44962100 | 1.34553100 | 3.21291700 |
| C | -2.25996400 | 0.56294700 | 2.83011800 |
| C | -0.18076400 | -1.28545500 | 2.32129700 |
| H | -2.32478500 | 5.22823500 | 0.22713300 |
| H | -0.65035900 | 4.84505700 | 0.70753100 |
| H | -3.98395600 | 3.52092500 | -1.04389700 |
| H | -3.78933400 | 2.22797500 | 0.17437000 |
| H | -1.37319200 | 3.20596000 | -3.02228600 |
| H | -0.28166700 | 4.31859600 | -2.18123800 |
| H | 0.18761500 | 2.40858700 | 3.24441100 |
| H | 1.49972900 | 1.27013200 | 2.91047300 |
| H | -2.59566300 | 1.60479000 | 2.88108800 |
| H | -2.21362400 | 0.15511200 | 3.84879900 |
| H | -0.81105800 | -1.94614300 | 1.71516300 |
| H | -0.31398100 | -1.54403800 | 3.38037000 |
| P | 1.12325800 | 0.41662900 | -1.30785200 |
| C | 1.30644200 | 0.96266900 | -3.07865600 |
| C | 2.87662000 | 0.64088600 | -0.73043800 |
| C | 1.09510100 | -1.42544500 | -1.56013100 |
| H | 1.50425300 | 2.04006300 | -3.09967900 |
| H | 0.36415100 | 0.78646600 | -3.61020900 |
| H | 3.09499200 | 1.71042600 | -0.63615900 |
| H | 3.60360400 | 0.18967800 | -1.41805000 |
| H | 1.15910700 | -1.92528600 | -0.58757800 |
| H | 1.91848100 | -1.77372800 | -2.19737400 |
| H | -2.00274300 | 4.08627900 | 1.56132700 |
| H | -3.55380400 | 1.86238100 | -1.54075200 |
| H | -2.00997800 | 4.72896700 | -2.34330400 |
| H | 2.11528200 | 0.44143500 | -3.60851400 |
| H | 2.99325500 | 0.18688600 | 0.25968000 |
| H | 0.14457600 | -1.71537200 | -2.02228400 |

| | | | |
|---|---|---|---|
| H | 0.86298900 | -1.46747800 | 2.04174300 |
| H | -3.00486100 | -0.00272300 | 2.25874800 |
| H | 0.33526600 | 0.91787100 | 4.21737200 |

**$Ni(PMe_3)_2$**

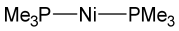

E = -1091.70878163

$G_{corr}$ = 0.186059

| | | | |
|---|---|---|---|
| Ni | 2.97653100 | 7.45427600 | 11.84232000 |
| P | 1.82795000 | 9.14246000 | 11.22109500 |
| P | 4.14393200 | 5.80034500 | 12.51666300 |
| C | 1.88573500 | 10.63545200 | 12.32156800 |
| C | -0.00517300 | 8.91423100 | 11.04646100 |
| C | 2.23965600 | 9.90648800 | 9.58127300 |
| C | 5.41142300 | 6.14879300 | 13.82612300 |
| C | 3.24381700 | 4.37090300 | 13.28343600 |
| C | 5.18372900 | 4.91534300 | 11.26115800 |
| H | 1.26234000 | 11.45165600 | 11.93504300 |
| H | 2.92012100 | 10.98512800 | 12.40776900 |
| H | -0.50279800 | 9.84149100 | 10.73504100 |
| H | -0.42457300 | 8.58604800 | 12.00350800 |
| H | 2.14014300 | 9.15418500 | 8.79142000 |
| H | 3.28041500 | 10.24787900 | 9.59046600 |
| H | 4.91633300 | 6.55807700 | 14.71323900 |
| H | 6.12009100 | 6.89925000 | 13.45983000 |
| H | 2.66092700 | 4.72481400 | 14.14044200 |
| H | 3.93479200 | 3.58754600 | 13.61952600 |
| H | 4.54196200 | 4.52317100 | 10.46499000 |
| H | 5.74499000 | 4.08580600 | 11.70958700 |
| H | 1.53836800 | 10.36244300 | 13.32377500 |
| H | -0.20799900 | 8.13331600 | 10.30567500 |
| H | 1.58494200 | 10.75684500 | 9.35259500 |
| H | 5.88904600 | 5.62001800 | 10.80771500 |
| H | 2.54610100 | 3.94431300 | 12.55477600 |
| H | 5.96277700 | 5.24313800 | 14.10964700 |

**$PMe_3$**

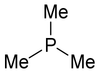

E = -461.172525307

$G_{corr}$ = 0.084469

| | | | |
|---|---|---|---|
| P | 1.67484700 | -0.11566400 | -0.01330200 |
| C | 2.55783100 | 0.74151800 | -1.41288500 |
| H | 2.39837900 | 0.18879300 | -2.34593100 |
| H | 2.14845100 | 1.74901800 | -1.54919800 |
| H | 3.63817000 | 0.82334900 | -1.23581400 |
| C | 2.37136300 | 0.85366700 | 1.41672300 |
| H | 1.97765200 | 1.87654400 | 1.39773100 |
| H | 2.05965700 | 0.39537400 | 2.36235600 |
| H | 3.46792000 | 0.90020800 | 1.39517500 |
| C | 2.73439300 | -1.64250600 | 0.11665500 |

| | | | |
|---|---|---|---|
| H | 2.46666600 | -2.20518100 | 1.01842200 |
| H | 2.55297500 | -2.29344900 | -0.74633300 |
| H | 3.80560600 | -1.40598700 | 0.15666900 |

**PhI**

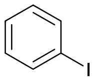

E = -243.107411301

$G_{corr}$ = 0.058655

| | | | |
|---|---|---|---|
| C | 2.37157500 | -0.21732900 | 0.00004300 |
| C | 3.76822800 | -0.23265400 | 0.00051000 |
| C | 4.45944700 | 0.98171500 | -0.00005600 |
| C | 3.76393600 | 2.19238200 | -0.00103400 |
| C | 2.36761900 | 2.19011800 | -0.00148000 |
| C | 1.66000600 | 0.98489800 | -0.00094800 |
| H | 4.31110600 | -1.17155600 | 0.00129100 |
| H | 5.54623300 | 0.97381700 | 0.00029700 |
| H | 4.30742100 | 3.13298200 | -0.00147600 |
| H | 1.81887400 | 3.12816300 | -0.00226200 |
| H | 0.57508200 | 0.98662000 | -0.00129100 |
| I | 1.31136300 | -2.06288500 | 0.00090300 |

**Ph•**

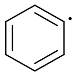

E = -231.634469524

$G_{corr}$ = 0.059636

| | | | |
|---|---|---|---|
| C | 1.08903600 | -0.03726000 | 0.00006800 |
| C | 2.48710300 | -0.02944900 | 0.00046800 |
| C | 3.19309100 | 1.17730400 | -0.00006300 |
| C | 2.50138000 | 2.40050800 | -0.00093800 |
| C | 1.12511000 | 2.33023600 | -0.00131100 |
| C | 0.37584200 | 1.17352000 | -0.00088100 |
| H | 0.54732900 | -0.98048800 | 0.00048500 |
| H | 3.03023100 | -0.97063200 | 0.00119900 |
| H | 4.28086300 | 1.17485100 | 0.00024600 |
| H | 3.03752200 | 3.34612200 | -0.00135700 |
| H | -0.71121200 | 1.18196800 | -0.00119500 |

**Toluene**

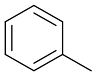

E = -271.655265649

$G_{corr}$ = 0.098610

| | | | |
|---|---|---|---|
| C | 5.36852300 | -0.42567700 | 0.02178100 |
| H | 4.47301500 | -0.85535400 | 0.49141000 |
| H | 5.87686300 | 0.17269100 | 0.78652500 |
| H | 6.02627100 | -1.25953500 | -0.24677800 |

| | | | |
|---|---|---|---|
| C | 5.00528800 | 0.40794500 | -1.18421600 |
| C | 4.60796100 | 1.74478800 | -1.03725900 |
| C | 5.03057800 | -0.13799500 | -2.47461600 |
| C | 4.24480600 | 2.51280100 | -2.14452200 |
| H | 4.58602300 | 2.18782400 | -0.04364900 |
| C | 4.66816900 | 0.62597900 | -3.58604900 |
| H | 5.33961500 | -1.17241400 | -2.60986700 |
| C | 4.27322300 | 1.95542100 | -3.42513600 |
| H | 3.94332800 | 3.54841800 | -2.00750700 |
| H | 4.69804800 | 0.18236500 | -4.57820700 |
| H | 3.99451800 | 2.55299600 | -4.28916600 |

**Toluene radical**

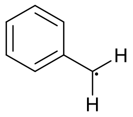


E = -271.002248804

$G_{corr}$ = 0.085412

| | | | |
|---|---|---|---|
| C | 6.50908800 | 1.27618600 | -0.21000700 |
| H | 6.67673200 | 1.81105700 | 0.71994700 |
| H | 6.69715200 | 0.20676400 | -0.21611400 |
| C | 6.06368900 | 1.94410500 | -1.36490400 |
| C | 5.81901300 | 3.35063500 | -1.36239100 |
| C | 5.83781300 | 1.24266900 | -2.58778000 |
| C | 5.37988800 | 4.00260500 | -2.50438500 |
| H | 5.98439800 | 3.90922400 | -0.44413800 |
| C | 5.39869600 | 1.90613500 | -3.72307600 |
| H | 6.01733000 | 0.17054000 | -2.61621900 |
| C | 5.16527600 | 3.28938000 | -3.69350100 |
| H | 5.20012900 | 5.07449300 | -2.47747600 |
| H | 5.23410300 | 1.34969400 | -4.64248800 |
| H | 4.82056800 | 3.80490100 | -4.58553300 |

**H Abstraction TS: Ph• + Toluene**

E = -503.285594423

$G_{corr}$ = 0.170965

ν = -1107.17 $cm^{-1}$

| | | | |
|---|---|---|---|
| C | 0.48657400 | -0.67541400 | 0.05715700 |
| C | 1.87801500 | -0.62389000 | 0.22491400 |
| C | 2.62063300 | 0.06446100 | -0.72007300 |
| C | 2.06170500 | 0.70079100 | -1.81565800 |
| C | 0.66967500 | 0.64194600 | -1.97270200 |
| C | -0.11121200 | -0.04442600 | -1.03795100 |
| H | -0.12625600 | -1.20779900 | 0.78132700 |
| H | 2.35048400 | -1.11237100 | 1.07427100 |
| H | 2.68330000 | 1.23079800 | -2.53388300 |
| H | 0.19919300 | 1.13087700 | -2.82283500 |
| H | -1.19007900 | -0.08773700 | -1.16298900 |
| C | 5.34497900 | 0.34502300 | -0.68351400 |
| H | 4.12462700 | 0.17578200 | -0.63411700 |
| H | 5.57819400 | 0.84416900 | 0.26109900 |
| H | 5.75058500 | -0.67043500 | -0.70122800 |
| C | 5.65257400 | 1.14709700 | -1.89141800 |
| C | 5.63552400 | 2.55332100 | -1.84745900 |
| C | 5.89697600 | 0.52463700 | -3.12910900 |
| C | 5.86516100 | 3.30919600 | -2.99612700 |
| H | 5.44017300 | 3.05209700 | -0.90071900 |
| C | 6.12706200 | 1.27939000 | -4.27842200 |
| H | 5.90665200 | -0.56165800 | -3.18358100 |
| C | 6.11232800 | 2.67569400 | -4.21740000 |
| H | 5.85249700 | 4.39477700 | -2.93889600 |
| H | 6.31910800 | 0.77746900 | -5.22345500 |
| H | 6.29291200 | 3.26445000 | -5.11287900 |

**TS-1-2-I-$PMe_3$**

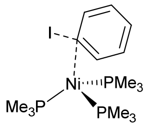


E = -1796.04472876

$G_{corr}$ = 0.377611

ν = -45.52 $cm^{-1}$

| | | | |
|---|---|---|---|
| Ni | -1.22766800 | -0.66595800 | 0.31022100 |
| P | -0.47472200 | 0.44258700 | -1.45468100 |
| P | -0.40115100 | -2.73170400 | 0.26434200 |
| P | -3.45091400 | -0.79752000 | 0.16192500 |
| C | -0.71802400 | 0.67184700 | 1.97539500 |
| C | -1.50084000 | -0.12509200 | 2.85331200 |
| C | 0.68906900 | 0.47397000 | 1.95915700 |
| C | -0.88127800 | -1.04110400 | 3.71839800 |
| H | -2.56354100 | 0.06567900 | 2.94323100 |
| C | 1.28291200 | -0.44784400 | 2.82156700 |
| H | 1.30783200 | 1.09218600 | 1.32082900 |
| C | 0.50441700 | -1.18979600 | 3.72128100 |
| H | -1.49831700 | -1.61883400 | 4.40218300 |
| H | 2.36310100 | -0.57000800 | 2.80122800 |
| H | 0.97917800 | -1.88261200 | 4.41100100 |
| C | -4.37843200 | -1.98627000 | 1.25045300 |
| H | -4.18701500 | -1.74348200 | 2.30142400 |
| H | -5.46151500 | -1.96581900 | 1.07230200 |
| H | -4.01237000 | -3.00380100 | 1.07441100 |
| C | -4.51571300 | 0.70895000 | 0.37071900 |
| H | -5.57470200 | 0.49466800 | 0.17592900 |
| H | -4.41527000 | 1.09855600 | 1.38922100 |
| H | -4.17561500 | 1.49190000 | -0.31434900 |
| C | -4.04610300 | -1.37437100 | -1.50097300 |
| H | -3.72014300 | -0.66715000 | -2.27068000 |
| H | -3.60295100 | -2.34834000 | -1.73363900 |
| H | -5.13929400 | -1.46184700 | -1.54502900 |
| C | -0.16525900 | -0.60209000 | -2.95954300 |
| H | 0.13981500 | 0.00089100 | -3.82426500 |
| H | 0.62301100 | -1.33242500 | -2.74812300 |

| | | | |
|---|---|---|---|
| H | -1.07907800 | -1.14842200 | -3.21718200 |
| C | -1.58327900 | 1.72918500 | -2.21069300 |
| H | -2.55308800 | 1.28478000 | -2.45789700 |
| H | -1.75385600 | 2.53182100 | -1.48716200 |
| H | -1.15156000 | 2.15870700 | -3.12399100 |
| C | 1.10859500 | 1.41180000 | -1.41396100 |
| H | 1.93124100 | 0.76377600 | -1.09313300 |
| H | 1.35249700 | 1.83686700 | -2.39577000 |
| H | 1.01419100 | 2.23274900 | -0.69533800 |
| C | 1.43623300 | -2.94819300 | 0.09344800 |
| H | 1.93439800 | -2.46371100 | 0.93915200 |
| H | 1.73335600 | -4.00446900 | 0.05888000 |
| H | 1.77569000 | -2.45389800 | -0.82404200 |
| C | -0.97329800 | -3.81515600 | -1.13499200 |
| H | -0.80027600 | -3.31943000 | -2.09495000 |
| H | -0.45420400 | -4.78211900 | -1.13931100 |
| H | -2.04858900 | -4.00076800 | -1.04013200 |
| C | -0.72683400 | -3.88963700 | 1.67755700 |
| H | -0.24516300 | -3.50857800 | 2.58141700 |
| H | -1.80498400 | -3.93183600 | 1.86601300 |
| H | -0.35507000 | -4.90178600 | 1.47294700 |
| I | -1.41225000 | 2.76794000 | 1.66203000 |

**4-I-$PMe_3$**

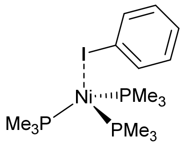


E = -1796.05331975

$G_{corr}$ = 0.374202

| | | | |
|---|---|---|---|
| Ni | 2.49049200 | 7.76494000 | 11.86972100 |
| P | 1.46795900 | 9.29256500 | 10.69778800 |
| C | 1.23004600 | 10.98173200 | 11.44135700 |
| C | -0.26941500 | 8.98593600 | 10.10796100 |
| C | 2.26339900 | 9.77627500 | 9.08974300 |
| H | 0.71601800 | 11.67288700 | 10.76046300 |
| H | 2.20467600 | 11.40681300 | 11.70368600 |
| H | -0.65036100 | 9.79746700 | 9.47359200 |
| H | -0.93252400 | 8.87671300 | 10.97421500 |
| H | 2.24374600 | 8.92718800 | 8.39963000 |
| H | 3.31173000 | 10.03838600 | 9.26806600 |
| P | 4.03338300 | 8.70826300 | 13.10119900 |
| P | 3.06058600 | 6.00247100 | 10.71197300 |
| C | 5.09107700 | 9.95087300 | 12.20812800 |
| C | 5.38654800 | 7.74459800 | 13.94588800 |
| C | 3.47063200 | 9.73357100 | 14.54534400 |
| C | 3.17482300 | 6.18102000 | 8.86327100 |
| C | 4.72276900 | 5.21689300 | 11.01566600 |
| C | 1.98720300 | 4.48897900 | 10.81096500 |
| H | 5.85312900 | 10.39602800 | 12.86130700 |
| H | 4.46386700 | 10.75333700 | 11.80533800 |
| H | 6.10153700 | 8.40062200 | 14.46018000 |
| H | 5.92868200 | 7.14340300 | 13.20744600 |
| H | 2.95672900 | 9.08490900 | 15.26386500 |
| H | 2.74859000 | 10.48094100 | 14.19931400 |
| H | 3.88197400 | 6.98150100 | 8.61922200 |
| H | 2.19394100 | 6.45953900 | 8.46356800 |
| H | 5.50704200 | 5.96923500 | 10.87452100 |
| H | 4.91693700 | 4.37303300 | 10.34013400 |
| H | 1.94903500 | 4.13711200 | 11.84752800 |
| H | 2.35241600 | 3.67435300 | 10.17203500 |
| H | 0.64554800 | 10.89092200 | 12.36357700 |
| H | -0.30258100 | 8.04654900 | 9.54503100 |
| H | 1.75577600 | 10.62692100 | 8.61665300 |
| H | 3.50436100 | 5.25456000 | 8.37465100 |
| H | 4.78181200 | 4.85623200 | 12.04789700 |
| H | 0.96640800 | 4.74911200 | 10.51028600 |
| H | 4.29982300 | 10.24140200 | 15.05539300 |
| H | 5.58894700 | 9.45884100 | 11.36488800 |
| H | 4.94959700 | 7.06209400 | 14.68234200 |
| I | 0.59453300 | 6.98089800 | 13.52685400 |
| C | 1.45960400 | 5.42557000 | 14.72272300 |
| C | 2.79055200 | 5.09202400 | 14.47767900 |
| C | 0.71528100 | 4.76832200 | 15.70376700 |
| C | 3.39016200 | 4.07971400 | 15.23186900 |
| H | 3.33811700 | 5.62536400 | 13.70451000 |
| C | 1.32683100 | 3.75690800 | 16.45108800 |
| H | -0.32175800 | 5.03495800 | 15.88610100 |
| C | 2.66091900 | 3.41105300 | 16.21827100 |
| H | 4.42855300 | 3.81632700 | 15.04511600 |
| H | 0.75494300 | 3.23962200 | 17.21731800 |
| H | 3.12889900 | 2.62401500 | 16.80328400 |

**TS-4-5-I-$PMe_3$**

E (OSS) = -1796.04305854

E (T) = -1796.02642847

$S^2$(OSS) = 0.6013

$G_{corr}$ = 0.371398

ν = -188.75 $cm^{-1}$

| | | | |
|---|---|---|---|
| Ni | -1.68677000 | 1.86665100 | 0.02369100 |
| P | -2.46821200 | 3.47045700 | -1.33098600 |
| C | -2.90535100 | 5.07052300 | -0.51227800 |
| C | -4.04720400 | 3.07810500 | -2.21132200 |
| C | -1.41106300 | 4.05967500 | -2.73263100 |
| H | -3.35449100 | 5.78567100 | -1.21276600 |
| H | -2.00617800 | 5.52123800 | -0.07845000 |
| H | -4.37313500 | 3.89673900 | -2.86532600 |
| H | -4.82580700 | 2.87387400 | -1.46938800 |
| H | -1.22267500 | 3.22874000 | -3.41973500 |
| H | -0.44546900 | 4.40303100 | -2.34672300 |
| P | -0.07963000 | 2.83781900 | 1.27305500 |
| P | -1.22264000 | 0.05003200 | -1.22720200 |
| C | 0.94934800 | 4.13485700 | 0.43559100 |
| C | 1.26296400 | 1.80468200 | 2.02669300 |
| C | -0.67492000 | 3.75738700 | 2.76343800 |
| C | -1.20260400 | 0.23833100 | -3.07170800 |

| | | | |
|---|---|---|---|
| C | 0.41963400 | -0.77736700 | -0.97731300 |
| C | -2.37039300 | -1.38483300 | -1.02731600 |
| H | 1.69157200 | 4.57156700 | 1.11560300 |
| H | 0.30690900 | 4.93831700 | 0.06014500 |
| H | 2.00658300 | 2.42106800 | 2.54787000 |
| H | 1.76656200 | 1.22770700 | 1.24335500 |
| H | -1.19030600 | 3.05496000 | 3.42672400 |
| H | -1.40437900 | 4.51318400 | 2.45334900 |
| H | -0.42017000 | 0.94911800 | -3.35864600 |
| H | -2.16555100 | 0.63131600 | -3.41492100 |
| H | 1.22355300 | -0.05301800 | -1.14907300 |
| H | 0.56035000 | -1.62996900 | -1.65421900 |
| H | -2.37013500 | -1.69977000 | 0.02112900 |
| H | -2.08954000 | -2.23479400 | -1.66189200 |
| H | -3.60897100 | 4.87046700 | 0.30204900 |
| H | -3.91471100 | 2.17167900 | -2.81193300 |
| H | -1.88385200 | 4.87845600 | -3.28893000 |
| H | -1.01364300 | -0.71619100 | -3.57880100 |
| H | 0.49753300 | -1.13509700 | 0.05408200 |
| H | -3.38861100 | -1.06841100 | -1.27609600 |
| H | 0.14072400 | 4.24407600 | 3.31301300 |
| H | 1.47246600 | 3.68961900 | -0.41810200 |
| H | 0.82721000 | 1.10117000 | 2.74235500 |
| I | -3.62365500 | 1.24820000 | 1.63132900 |
| C | -2.57836600 | -0.54034600 | 2.98269400 |
| C | -1.27683000 | -0.82185300 | 2.60465500 |
| C | -3.20369700 | -1.20546300 | 4.02838600 |
| C | -0.56117000 | -1.80824500 | 3.29568400 |
| H | -0.82701800 | -0.26730500 | 1.77817100 |
| C | -2.48529900 | -2.19255400 | 4.71931400 |
| H | -4.22802500 | -0.97094200 | 4.31212500 |
| C | -1.16858400 | -2.49223600 | 4.35396300 |
| H | 0.46423300 | -2.04138400 | 3.01287800 |
| H | -2.95471500 | -2.72598700 | 5.54380000 |
| H | -0.61716200 | -3.25769100 | 4.89431700 |

**5-I-$PMe_3$**

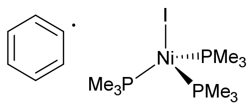


E (OSS) = -1796.04908611

E (T) = -1796.04880792

$S^2$(OSS) = 1.0098

$G_{corr}$ = 0.369947

| | | | |
|---|---|---|---|
| Ni | -1.01348900 | 1.33721100 | 0.10126100 |
| P | -1.84396800 | 2.74744200 | -1.52362800 |
| C | -2.33544900 | 4.38923200 | -0.83349200 |
| C | -3.40414500 | 2.20609300 | -2.34909300 |
| C | -0.79847400 | 3.24576700 | -2.96855300 |
| H | -2.82310400 | 5.02208300 | -1.58497700 |
| H | -1.44924900 | 4.90885900 | -0.45251300 |
| H | -3.80187500 | 2.96734300 | -3.03134500 |
| H | -4.14553400 | 1.98576200 | -1.57438100 |
| H | -0.55882500 | 2.36754600 | -3.57638600 |
| H | 0.14376400 | 3.67306700 | -2.60742700 |
| P | 0.78266800 | 2.07515300 | 1.31078900 |
| P | -0.48629200 | -0.58500000 | -1.04271400 |
| C | 2.03733200 | 2.99424600 | 0.30419700 |
| C | 1.88259800 | 0.96208000 | 2.29957800 |
| C | 0.30580400 | 3.33260500 | 2.57283600 |
| C | -0.30324700 | -0.49879900 | -2.88041900 |
| C | 1.02325600 | -1.54509900 | -0.58284900 |
| C | -1.83063400 | -1.83041000 | -0.84432000 |
| H | 2.84907800 | 3.39997700 | 0.92088600 |
| H | 1.54675200 | 3.82077900 | -0.22116400 |
| H | 2.69803900 | 1.52275100 | 2.77288200 |
| H | 2.31496100 | 0.19395300 | 1.64982700 |
| H | -0.33503000 | 2.85446300 | 3.32035200 |
| H | -0.28343900 | 4.12084900 | 2.09248200 |
| H | 0.54863600 | 0.14054700 | -3.13607400 |
| H | -1.20589700 | -0.06309000 | -3.32103200 |
| H | 1.91387300 | -0.92876300 | -0.74762500 |
| H | 1.11549600 | -2.46502200 | -1.17291300 |
| H | -1.95150100 | -2.06263700 | 0.21774800 |
| H | -1.62111000 | -2.75232200 | -1.40000000 |
| H | -3.01981700 | 4.22531400 | 0.00490000 |
| H | -3.22226400 | 1.28341100 | -2.91140800 |
| H | -1.30259000 | 3.98501800 | -3.60293900 |
| H | -0.14507600 | -1.49159900 | -3.31879500 |
| H | 0.97346300 | -1.81391500 | 0.47586500 |
| H | -2.77265900 | -1.39647600 | -1.19490900 |
| H | 1.17756100 | 3.77834900 | 3.06699200 |
| H | 2.46570400 | 2.32401600 | -0.44988400 |
| H | 1.29744900 | 0.46964000 | 3.08133700 |
| I | -3.13896800 | 1.25303300 | 1.74196900 |
| C | -1.99922700 | -2.02430900 | 3.07063500 |
| C | -0.72747500 | -1.58913100 | 2.76067300 |
| C | -2.30309000 | -3.16866300 | 3.77721800 |
| C | 0.34124900 | -2.38440700 | 3.20763200 |
| H | -0.56856600 | -0.67710000 | 2.19192900 |
| C | -1.21929500 | -3.95225900 | 4.21133700 |
| H | -3.32675600 | -3.46181100 | 3.99610900 |
| C | 0.09198000 | -3.55824400 | 3.92736000 |
| H | 1.36475400 | -2.08214400 | 2.99598800 |
| H | -1.40565800 | -4.86553500 | 4.77227300 |
| H | 0.92380500 | -4.16761000 | 4.27109300 |

**6-I-$PMe_3$**

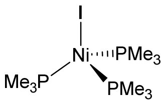


E = -1564.40705784

$G_{corr}$ = 0.287279

| | | | |
|---|---|---|---|
| Ni | -1.84324800 | -0.47041800 | 0.07921600 |
| P | -3.67235000 | 0.83299800 | -0.33312000 |

| | | | |
|---|---|---|---|
| C | -4.17073000 | 2.07806700 | 0.93713600 |
| C | -5.20502000 | -0.18462800 | -0.49073500 |
| C | -3.71537500 | 1.84136500 | -1.88501700 |
| H | -5.13735100 | 2.54125700 | 0.70425000 |
| H | -3.40975300 | 2.86365600 | 0.99811800 |
| H | -6.09094100 | 0.42899800 | -0.69384000 |
| H | -5.35087600 | -0.74269800 | 0.43972200 |
| H | -3.55483700 | 1.18376100 | -2.74671500 |
| H | -2.90261900 | 2.57640500 | -1.86908300 |
| P | -0.14197700 | 0.93142700 | 0.69769200 |
| P | -0.96025700 | -1.86388100 | -1.49890000 |
| C | 0.36832000 | 2.34285100 | -0.38093900 |
| C | 1.45901800 | 0.06443300 | 1.00569400 |
| C | -0.45114800 | 1.75441900 | 2.31875600 |
| C | 0.06553300 | -1.13883000 | -2.85632700 |
| C | 0.10925200 | -3.24578600 | -0.90411300 |
| C | -2.26680500 | -2.77092200 | -2.43936500 |
| H | 1.20553300 | 2.91013500 | 0.04293100 |
| H | -0.48304700 | 3.01876100 | -0.51857700 |
| H | 2.21298200 | 0.72914500 | 1.44426200 |
| H | 1.85020500 | -0.34044000 | 0.06586400 |
| H | -0.70886200 | 0.98519200 | 3.05354400 |
| H | -1.30629200 | 2.43263400 | 2.23085400 |
| H | 0.97406800 | -0.69586400 | -2.43361700 |
| H | -0.49920900 | -0.34275900 | -3.35401200 |
| H | 0.99547200 | -2.83431100 | -0.40903800 |
| H | 0.42646300 | -3.91106000 | -1.71673700 |
| H | -2.91041000 | -3.29548200 | -1.72511300 |
| H | -1.84768200 | -3.49675300 | -3.14646500 |
| H | -4.23258200 | 1.58340400 | 1.91170300 |
| H | -5.07727700 | -0.91280700 | -1.29921900 |
| H | -4.66769300 | 2.37070500 | -2.01253700 |
| H | 0.35840700 | -1.88907400 | -3.60093100 |
| H | -0.44854300 | -3.82098000 | -0.15809500 |
| H | -2.88296300 | -2.05157500 | -2.99025600 |
| H | 0.42104200 | 2.31937900 | 2.66880900 |
| H | 0.65791700 | 1.96145200 | -1.36634900 |
| H | 1.27410900 | -0.77149300 | 1.68817600 |
| I | -2.49900300 | -2.01070500 | 2.17983600 |

**7-I-$PMe_3$**

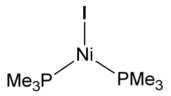


E = -1103.20076166

$G_{corr}$ = 0.179104

| | | | |
|---|---|---|---|
| Ni | 3.02107300 | 7.39600400 | 11.66721000 |
| P | 1.70436400 | 9.17505000 | 11.23412200 |
| P | 2.68797800 | 6.74339800 | 13.81325500 |
| C | 1.67778300 | 10.51280700 | 12.50681500 |
| C | -0.09120800 | 8.81947700 | 10.99845700 |
| C | 2.13905100 | 10.09063600 | 9.69413400 |
| C | 3.66405900 | 7.70987000 | 15.04595400 |
| C | 0.98561800 | 6.78770700 | 14.52758800 |
| C | 3.21612900 | 5.00879800 | 14.14315600 |
| H | 1.06731200 | 11.36551300 | 12.18679200 |
| H | 2.70003000 | 10.85700500 | 12.69592800 |
| H | -0.66755400 | 9.73261700 | 10.80900900 |
| H | -0.48658800 | 8.32496400 | 11.89205900 |
| H | 2.09272000 | 9.40044900 | 8.84533200 |
| H | 3.16784900 | 10.45810900 | 9.76951400 |
| H | 3.34566500 | 8.75787800 | 15.02748100 |
| H | 4.72298100 | 7.67434800 | 14.76902600 |
| H | 0.59966400 | 7.81254300 | 14.50468800 |
| H | 0.97072900 | 6.42970200 | 15.56371600 |
| H | 2.61246500 | 4.32647600 | 13.53548700 |
| H | 3.11262400 | 4.74087500 | 15.20133100 |
| H | 1.27001500 | 10.12157700 | 13.44506400 |
| H | -0.21515500 | 8.13603900 | 10.15179000 |
| H | 1.46406800 | 10.93554000 | 9.51324400 |
| H | 4.26021300 | 4.88926600 | 13.83718800 |
| H | 0.32163600 | 6.15787600 | 13.92568000 |
| H | 3.54516700 | 7.31616200 | 16.06226700 |
| I | 4.75342400 | 6.19204700 | 10.24449700 |

## In *n*-Hexane

### PhH

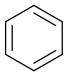

E = -232.323983605

$G_{corr}$ = 0.073465

| | | | |
|---|---|---|---|
| C | 3.70589900 | 1.91299500 | 0.09731400 |
| C | 5.02855700 | 2.16828700 | -0.27308500 |
| C | 5.30158700 | 2.86508100 | -1.45295300 |
| C | 4.25182000 | 3.30556600 | -2.26273300 |
| C | 2.92907100 | 3.05022800 | -1.89226100 |
| C | 2.65602400 | 2.35345000 | -0.71257900 |
| H | 3.49371700 | 1.37081000 | 1.01535700 |
| H | 5.84529900 | 1.82523200 | 0.35707100 |
| H | 4.46380500 | 3.84788300 | -3.18074700 |
| H | 2.11242000 | 3.39344200 | -2.52243700 |
| H | 1.62680800 | 2.15474600 | -0.42445400 |
| H | 6.33077500 | 3.06381900 | -1.74109800 |

### Ph•

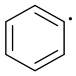

E = -231.634151943

$G_{corr}$ = 0.059624

| | | | |
|---|---|---|---|
| C | 1.08911600 | -0.03717900 | 0.00006800 |
| C | 2.48709500 | -0.02944000 | 0.00046700 |
| C | 3.19297100 | 1.17727300 | -0.00006400 |
| C | 2.50128200 | 2.40032400 | -0.00093700 |
| C | 1.12504800 | 2.33036600 | -0.00131100 |
| C | 0.37606800 | 1.17352000 | -0.00088000 |
| H | 0.54753700 | -0.98047900 | 0.00048500 |
| H | 3.03019000 | -0.97061800 | 0.00119900 |
| H | 4.28073200 | 1.17470000 | 0.00024600 |
| H | 3.03719100 | 3.34602400 | -0.00135700 |
| H | -0.71093500 | 1.18219000 | -0.00119600 |

### Hexane

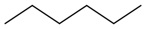

E = -237.171989133

$G_{corr}$ = 0.158283

| | | | |
|---|---|---|---|
| C | 4.30635300 | 1.65426000 | -0.70126800 |
| H | 4.96429200 | 2.26450200 | -1.33697500 |
| H | 3.30432200 | 1.72035900 | -1.14707800 |
| C | 4.77290600 | 0.19029800 | -0.75004600 |
| H | 4.10385100 | -0.42259700 | -0.12725000 |
| H | 4.66001800 | -0.18425700 | -1.77768600 |
| C | 6.22335100 | -0.03257900 | -0.30206500 |
| H | 6.34756100 | 0.28332200 | 0.74293000 |
| H | 6.88905900 | 0.61119400 | -0.89710500 |
| C | 6.67864400 | -1.49139000 | -0.43553600 |
| H | 6.56112500 | -1.81377000 | -1.48005700 |
| H | 6.01260300 | -2.13369600 | 0.15855000 |
| C | 8.12800200 | -1.71011000 | 0.00700500 |
| H | 8.26850600 | -1.42712900 | 1.05812900 |
| H | 8.82056400 | -1.10520200 | -0.59220700 |
| H | 8.42765400 | -2.75989700 | -0.09717400 |
| C | 4.26433400 | 2.25193400 | 0.70936300 |
| H | 3.62672900 | 1.65261400 | 1.37246800 |
| H | 5.26111700 | 2.29885900 | 1.16216400 |
| H | 3.86304900 | 3.27239900 | 0.69494500 |

### Hexane radical

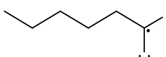


E = -236.505625587

$G_{corr}$ = 0.141932

| | | | |
|---|---|---|---|
| C | 4.14198600 | 2.86583800 | -0.96927300 |
| H | 4.23676800 | 3.61890500 | -1.75033400 |
| C | 4.75210000 | 1.52116800 | -1.20420800 |
| H | 4.19636700 | 0.75627200 | -0.63878900 |
| H | 4.67249900 | 1.24756100 | -2.26589200 |
| C | 6.24224800 | 1.43280300 | -0.78679900 |
| H | 6.33332700 | 1.68981200 | 0.27804700 |
| H | 6.81666200 | 2.19115900 | -1.33755900 |
| C | 6.85294300 | 0.04756400 | -1.03109700 |
| H | 6.75786500 | -0.20720300 | -2.09633400 |
| H | 6.26968800 | -0.70652800 | -0.48329700 |
| C | 8.32334400 | -0.03860800 | -0.61293100 |
| H | 8.44486700 | 0.18218900 | 0.45520600 |
| H | 8.93659900 | 0.68085000 | -1.17024700 |
| H | 8.73559700 | -1.03841200 | -0.79466400 |
| C | 3.77452800 | 3.32159200 | 0.40386400 |
| H | 3.36364600 | 2.49827000 | 1.00472000 |
| H | 4.64357400 | 3.71218500 | 0.96547700 |
| H | 3.03197300 | 4.12949100 | 0.38295500 |

### H Abstraction TS: Ph• + Hexane

E = -468.799635792

$G_{corr}$ = 0.228793

ν = -1304.15 $cm^{-1}$

| | | | |
|---|---|---|---|
| C | 0.55681800 | -0.96172200 | 0.05857600 |
| C | 1.92526500 | -0.67751400 | 0.15994000 |
| C | 2.49884800 | 0.16891700 | -0.77780100 |
| C | 1.76974000 | 0.74242900 | -1.80926800 |
| C | 0.40163500 | 0.45395200 | -1.90396200 |
| C | -0.20007000 | -0.39613100 | -0.97151900 |
| H | 0.08461400 | -1.62301700 | 0.78183600 |
| H | 2.51910700 | -1.11513500 | 0.95975500 |

| | | | |
|---|---|---|---|
| H | 2.24418000 | 1.40278500 | -2.53235200 |
| H | -0.19135800 | 0.89186500 | -2.70395800 |
| H | -1.26129000 | -0.61873800 | -1.04778100 |
| C | 5.15691000 | 0.66655100 | -0.54524300 |
| H | 5.39731900 | 1.24462000 | -1.44718000 |
| H | 3.92261800 | 0.45474300 | -0.67194100 |
| C | 5.82140000 | -0.70265900 | -0.55585400 |
| H | 5.48939900 | -1.27445800 | 0.32354600 |
| H | 5.47745400 | -1.26359200 | -1.43635800 |
| C | 7.35937700 | -0.65076200 | -0.56795900 |
| H | 7.71936500 | -0.12366500 | 0.32654300 |
| H | 7.69465200 | -0.05641300 | -1.43097500 |
| C | 8.00628800 | -2.04019400 | -0.62570700 |
| H | 7.65072900 | -2.56801500 | -1.52205000 |
| H | 7.66481200 | -2.63414000 | 0.23423000 |
| C | 9.53651900 | -1.98552100 | -0.63559200 |
| H | 9.92103100 | -1.49168100 | 0.26597300 |
| H | 9.90679100 | -1.42357600 | -1.50253200 |
| H | 9.97536600 | -2.98950200 | -0.67811700 |
| C | 5.31896300 | 1.48871800 | 0.72097900 |
| H | 5.00364400 | 0.91841000 | 1.60424500 |
| H | 6.36411000 | 1.78915300 | 0.87951600 |
| H | 4.71734900 | 2.40425700 | 0.68133600 |

# REFERENCES


[1] Gaussian 09, Revision D.01, M. J. Frisch, G. W. Trucks, H. B. Schlegel, G. E. Scuseria, M. A. Robb, J. R. Cheeseman, G. Scalmani, V. Barone, G. A. Petersson, H. Nakatsuji, X. Li, M. Caricato, A. Marenich, J. Bloino, B. G. Janesko, R. Gomperts, B. Mennucci, H. P. Hratchian, J. V. Ortiz, A. F. Izmaylov, J. L. Sonnenberg, D. Williams-Young, F. Ding, F. Lipparini, F. Egidi, J. Goings, B. Peng, A. Petrone, T. Henderson, D. Ranasinghe, V. G. Zakrzewski, J. Gao, N. Rega, G. Zheng, W. Liang, M. Hada, M. Ehara, K. Toyota, R. Fukuda, J. Hasegawa, M. Ishida, T. Nakajima, Y. Honda, O. Kitao, H. Nakai, T. Vreven, K. Throssell, J. A. Montgomery, Jr., J. E. Peralta, F. Ogliaro, M. Bearpark, J. J. Heyd, E. Brothers, K. N. Kudin, V. N. Staroverov, T. Keith, R. Kobayashi, J. Normand, K. Raghavachari, A. Rendell, J. C. Burant, S. S. Iyengar, J. Tomasi, M. Cossi, J. M. Millam, M. Klene, C. Adamo, R. Cammi, J. W. Ochterski, R. L. Martin, K. Morokuma, O. Farkas, J. B. Foresman, and D. J. Fox, Gaussian, Inc., Wallingford CT, 2016.

[2] Becke, A. D. *J. Chem. Phys.* **1993**, *98*, 5648-5652.

[3] C. Lee, W. Yang, R.G. Parr *Phys. Rev. B. Condens. Matter*. **1988**, *37*, 785-789.

[4] P. J. Stephens, F. J. Devlin, C. F. Chabalowski, M. J. Frisch, *J. Phys. Chem.* **1994**, *98*, 11623-11627.

[5] S. Grimme, J. Antony, S. Ehrlich, H. Krieg, *J. Chem. Phys.* **2010**, *132*, 154104-19.

[6] N. Fey, B. M. Ridgway, J. Jover, C. L. McMullin, J. N. Harvey, J. N. *Dalton Trans.* **2011**, *40*, 11184-11191.

[7] M. M. Francl, W. J. Pietro, W. J. Hehre, J. S. Binkley, M. S. Gordon, D. J. DeFrees, J. A. Pople, *J. Chem. Phys.* **1982**, *77*, 3654-3665.

[8] P. C. Hariharan, J. A. Pople, *Theoret. Chim. Acta* **1973**, *28*, 213-222.

[9] W. J. Hehre, R. Ditchfield, J. A. Pople, *J. Chem. Phys.* **1972**, *56*, 2257-2261.

[10] W. R. Wadt, P. J. Hay, *J. Chem. Phys.* **1985**, *82*, 284-298.

[11] P. J. Hay, W. R. Wadt, *J. Chem. Phys.* **1985**, *82*, 270-284.

[12] L. E. Roy, P. J. Hay, R. L. Martin, *J. Chem. Theor. Comput.* **2008**, *4*, 1029-1031.

[13] A. W. Ehlers, M. Böhme, S. Dapprich, A. Gobbi, A. Höllwarth, V. Jonas, K. F. Köhler, R. Stegmann, A. Veldkamp, G. Frenking, *Chem. Phys. Lett.* **1993**, *208*, 111-114.

[14] R. Krishnan, J.S. Binkley, R. Seeger, J.A. Pople, *J. Chem. Phys.* **1980**, *72*, 650-654.

[15] K. Yamaguchi, F. Jensen, A. Dorigo, K. N. Houk, *Chem. Phys. Lett.* **1988**, *149*, 537-542.

[16] S. Yamanaka, T. Kawakami, H. Nagao, K. Yamaguchi, *Chem. Phys. Lett.* **1994** , *231*, 25-33.

[17] M. H. Lim, S. E. Worthington, F. J. Dulles, C. J. Cramer, in *Chemical Applications of Density Functional Theory*, Vol. 629 (Eds. B. B. Laird, R. B. Ross, T. Ziegler), American Chemical Society, Washington DC, **1996**; p 402.

[18] H. Isobe, Y. Takano, Y. Kitagawa, T. Kawakami, S. Yamanaka, K. Yamaguchi, K. N. Houk, *Mol. Phys.* **2002**, *100*, 717-727.

[19] H. Yu, Y. Fu, Q. Guo, Z. Lin, *Organometallics* **2009**, *28*, 4443-4451.

[20] C. A. Tolman, *Chem. Rev.* **1977**, *77*, 313-348.

[21] C. A. Tolman, W. C. Seidel, L. W. Gosser, *J. Am. Chem. Soc.* **1974**, *96*, 53-60.

[22] J. A. Murphy, S.-Z. Zhou, D. W. Thomson, F. Schoenebeck, M. Mahesh, S. R. Park, T. Tuttle, L. E. A. Berlouis, *Angew. Chem. Int. Ed.* **2007**, *46*, 5178-5183.

[23] a) L. E. Rush, P. G. Pringle, J. N. Harvey, Angew. Chem. Int. Ed. **2014**, *53*, 8672-8676. b) C. H. Bamford, C. F. H. Tipper, R. G. Compon, *Comprehensive Chemical Kinetics*, *Vol. 25*, Elsevier, B.V., Amsterdam, **1985**.

[24] Taken from Sigma-Aldrich database: http://www.sigmaaldrich.com/chemistry/solvents/toluene-center.html

[25] Taken from Sigma-Aldrich database: http://www.sigmaaldrich.com/chemistry/solvents/tetrahydrofuran-center.html
